# Enhancing Temperature Sensitivity of Redox Potential via Redox-Induced Change in Copper Coordination Number by an External Ligand

**DOI:** 10.1021/jacs.5c21444

**Published:** 2026-03-31

**Authors:** Daniela Carmona-Pérez, William W. Brennessel, Agnes E. Thorarinsdottir

**Affiliations:** Department of Chemistry, 6927 University of Rochester, Rochester, New York 14627, United States

## Abstract

We report a new design strategy for enhancing the temperature sensitivity of molecular redox potentials that is based on a redox-induced change in the metal coordination number facilitated by an external ligand. The Cu complex bis­(2,9-dimethyl-1,10-phenanthroline)­copper­(II) binds coordinating ligands, including MeCN, whereas the Cu^I^ analog remains tetracoordinate. Upon introduction of a stronger donor, 4-*tert*-butylpyridine (^*t*^Bu-py), the MeCN ligand is displaced while retaining penta- and tetracoordination at the Cu^II^ and Cu^I^ centers, respectively. Variable-temperature electrochemical analysis reveals the temperature sensitivity of the apparent half-wave potential (*E*_1/2_) of the Cu^II^/Cu^I^ couple to increase from α = 1.70(6) mV °C^–1^ to α = 2.3(2) mV °C^–1^ upon addition of 15.0 equiv of ^*t*^Bu-py to the MeCN-bound Cu^II^ complex in MeCN solution. Similar analysis starting with the Cu^I^ complex affords α = 2.6(2) mV °C^–1^ in the presence of ^*t*^Bu-py, providing a record-high temperature sensitivity of *E*_1/2_ of the Cu^II^/Cu^I^ couple in MeCN solution.

## Graphical abstract

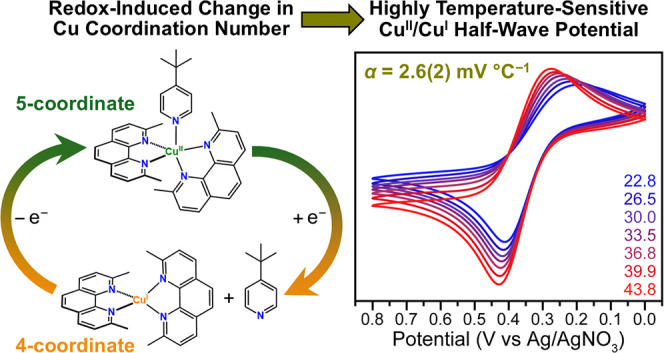

Over the past decade, variable-temperature electrochemistry has emerged as a powerful technique to study the electro- and thermochemical properties of molecules and materials.^1^ These studies are attractive for electrocatalysis,^2–6^ electrochemical sensing,^7–9^ the design of thermoelectrochemical devices for waste heat harvesting^10–16^ and electrochemical cooling,^17,18^ and providing valuable thermodynamic parameters of electron-transfer reactions.^19–26^ One key limitation for the implementation of compounds toward these applications is the lack of a complete understanding of the factors that dictate the temperature dependence of the electrochemical properties of molecules and materials. The temperature coefficient (α) of the formal potential (*E* ^0^′) is a critical parameter to consider when designing molecular compounds for targeted applications in electrochemistry, as it describes the change in a redox couple’s formal potential with respect to temperature and is linearly related to the redox reaction entropy (Δ*S*_redox_; eq 1).^1,10,11,27^

$$\Delta S_{\mathrm{redox}}=nF\left(\frac{\partial E^{0\prime }}{\partial T}\right)=nF\alpha$$ 1

The impact of charge,^26,28,29^ pH,^30,31^ solvent and solvation environment,^13,20,28,32–38^ spin state,^9,28,39–42^ and metal coordination environment^27,28,43,44^ on α for a series of molecular compounds has been reported. While these works have provided significant knowledge of structural and physical factors that influence α, further insights are needed to facilitate the design of compounds with optimal temperature coefficients for desired applications, such as large absolute temperature coefficients for effective heat-to-electricity conversions and electrochemical refrigeration.

Considering the linear correlation between Δ*S*_redox_ and α (eq 1), we envisioned that systems displaying large structural changes between redox states would afford large absolute temperature coefficients. One attractive system toward this end comprises a redox-active metal center that changes coordination number during the electron-transfer process, resulting in simultaneous changes in geometry and number of molecular components.^45^ As copper complexes are known to prefer hexa/pentacoordination and tetracoordination for the Cu^II^ and Cu^I^ redox states, respectively,^46,47^ we postulated that a redox-induced change in copper coordination number could afford large Δ*S*_redox_. Herein, we demonstrate notable enhancement in the temperature sensitivity of *E*_1/2_ of the Cu^II^/Cu^I^ redox couple by leveraging the redox-induced change in the coordination number approach through the use of an external ligand (Scheme 1), thereby providing a new design strategy toward increasing the temperature sensitivity of molecular redox potentials.

**Scheme 1:**
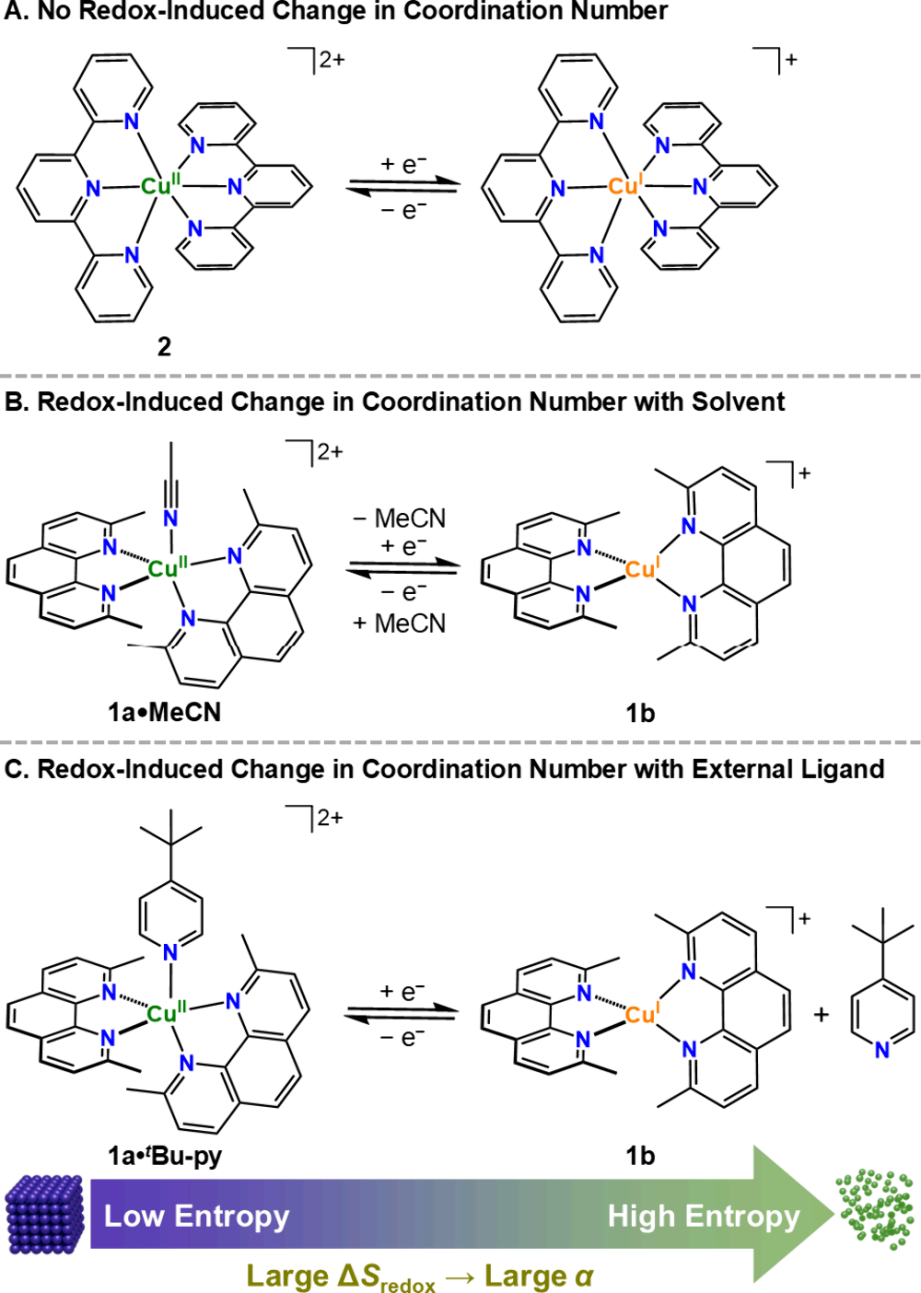
Overview of Cu^II^/Cu^I^ Redox Processes with No Change in Copper Coordination Number (**A**) and with Change Involving a Solvent Molecule (**B**) or an External Ligand (**C**) As Discussed in This Work

Copper complexes supported by 2,9-dimethyl-1,10-phenanthroline (Me_2_-phen) ligands were selected for these proof-of-concept studies, as they have previously been shown to favor pentacoordinate Cu^II^ and tetracoordinate Cu^I^ complexes.^48–50^ The mononuclear complexes [Cu­(Me_2_-phen)_2_(MeCN)]­(PF_6_)_2_ (**1a·MeCN**) and [Cu­(Me_2_-phen)_2_]­(PF_6_) (**1b**) were synthesized as green and orange-red solids, respectively (Experimental Section, Scheme S1, Figure S1).^48,51^ Slow diffusion of Et_2_O vapor into a concentrated MeCN solution of **1a·MeCN** gave green block-shaped crystals of [Cu­(Me_2_-phen)_2_(MeCN)]­(PF_6_)_2_·MeCN (**1a′**; Figures 1 and S2, Table S1). Application of vacuum and/or heat to these crystals afforded a purple polycrystalline solid corresponding to the desolvated tetracoordinate Cu^II^ complex **1a**.^48,50^ This solvation–desolvation behavior is reversible. Slow evaporation of a CH_2_Cl_2_ solution of **1b** afforded orange-red needle-shaped crystals matching the reported structure.^52^ The Cu^II^ center in **1a′** and Cu^I^ center in **1b** reside in distorted square pyramidal and tetrahedral coordination environments, respectively, as indicated by τ_5_ ^53^ and τ_4_ ^54^ parameters of 0.19(2) and 0.71(1), respectively (Table S2). While these structural metrics suggest that a redox-induced change in copper coordination number could be realized in MeCN solutions of **1a** and/or **1b** (Scheme 1B), we postulated that employing an external ligand would be more effective due to greater structural changes and less transient interactions (Scheme 1C).^50,55,56^ The external ligand 4-*tert*-butylpyridine (^*t*^Bu-py) was selected, as it has been shown to replace MeCN in **1a·MeCN** and display no coordination to the Cu^I^ center in **1b**.^49,50^ Reaction of **1a** with ^*t*^Bu-py in CH_2_Cl_2_ afforded [Cu­(Me_2_-phen)_2_(^*t*^Bu-py)]­(PF_6_)_2_·CH_2_Cl_2_ (**1a·** ^***t***^ **Bu-py**). Slow diffusion of Et_2_O vapor into a concentrated CH_2_Cl_2_ solution of **1a·** ^***t***^ **Bu-py** gave blue-green plate-shaped crystals (Figures 1 and S3, Table S1). The Cu^II^ center in **1a·** ^***t***^ **Bu-py** exhibits similar structural metrics to those in **1a′**, albeit displaying closer to ideal square pyramidal geometry (τ_5_ = 0.039(2); Table S2). To provide a related coordinatively saturated hexacoordinate Cu^II^ complex for comparison to pentacoordinate **1a·MeCN** and **1a·** ^***t***^ **Bu-py**, the pseudo-octahedral [Cu­(terpy)_2_]­(PF_6_)_2_ (**2**) was synthesized (Figure 1, Table S2).^57,58^

**Figure 1:**
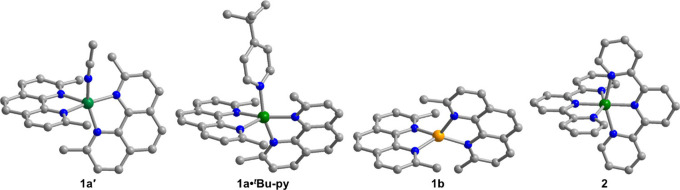
Crystal structures of the cationic complexes [Cu­(Me_2_-phen)_2_(MeCN)]^2+^, [Cu­(Me_2_-phen)_2_(^*t*^Bu-py)]^2+^, [Cu­(Me_2_-phen)_2_]^+^, and [Cu­(terpy)_2_]^2+^, as observed in **1a′**, **1a·** ^***t***^ **Bu-py**, **1b**,^52^ and **2**,^57^ respectively. Green, orange, blue, and gray spheres represent Cu^II^, Cu^I^, N, and C atoms, respectively; H atoms are omitted for clarity.

To investigate interactions between ^*t*^Bu-py and copper complexes **1a**, **1b**, and **2**, UV–visible–NIR spectrophotometric titrations were carried out in MeCN. The spectrum of **1a** is unaffected by air exposure (Figure S4) but shows clear changes upon addition of 0.2–15.0 equiv of ^*t*^Bu-py, with the d–d transition band^49,50,59^ shift from 725 to 717 nm and increase in intensity (Figures 2 and S5–S7). Insignificant spectral changes were observed upon further addition of up to 40.0 equiv of ^*t*^Bu-py (Figures S5–S7), indicating complete exchange of MeCN for ^*t*^Bu-py.^49,50^ As ^*t*^Bu-py does not absorb visible light (Figure S8), an effective *K*_eq_ = 8(2) × 10^2^ M^–1^ for the ligand-exchange reaction can be obtained from monitoring the absorbance at 725 nm (Experimental Section). This value aligns with prior studies^49^ and corroborates complete coordination of ^*t*^Bu-py to **1a** in the presence of 15.0 equiv of ^*t*^Bu-py (Table S3). The UV–visible–NIR spectra of **1a·** ^***t***^ **Bu-py** in the presence and absence of ^*t*^Bu-py align with these results (Figures S9–S13). Notably, dissociation of Me_2_-phen (Figures S14 and S15) occurs with >100 equiv of ^*t*^Bu-py, as the spectrum of [Cu­(^*t*^Bu-py)_4_(MeCN)]­(PF_6_)_2_ (**3**), which was synthesized as violet-blue plates of [*trans*-Cu­(^*t*^Bu-py)_4_(MeCN)_2_]­(PF_6_)_2_·Et_2_O·solvent (**3′**; Figures S16 and S17, Tables S1 and S4), shows similar features (Figures S18 and S19).^49,50^ Similar analysis of MeCN solutions of **1b** and **2** reveals no spectral changes (Figures 2, S20, and S21), supporting no interactions between ^*t*^Bu-py and the Cu^I^ and Cu^II^ centers in **1b** and **2**, respectively.

**Figure 2:**
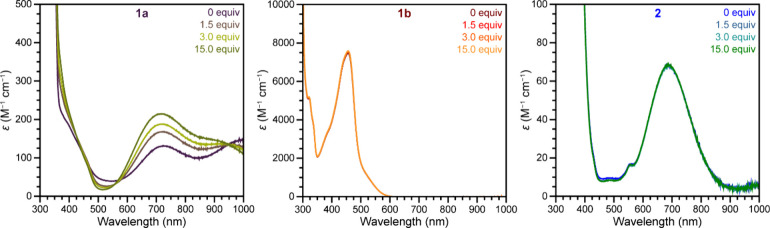
UV–visible–NIR spectra of **1a**, **1b**, and **2** in the presence of 0, 1.5, 3.0, and 15.0 equiv of ^*t*^Bu-py collected at room temperature in MeCN.

In anticipation of variable-temperature electrochemical analysis, we first sought to investigate the influence of ^*t*^Bu-py on the electrochemical properties of the copper complexes at room temperature (∼23 °C). The cyclic voltammograms (CVs) of **1a** and **1b** in MeCN containing 0.1 M (^*n*^Bu_4_N)­(PF_6_) supporting electrolyte exhibit a quasi-reversible Cu^II^/Cu^I^ redox wave with *E*_1/2_ = 0.382–0.384 V vs Ag/AgNO_3_ (Figures S22 and S23). Upon addition of ^*t*^Bu-py to a MeCN solution of **1a**, *E*_1/2_ shifts negatively, reaching a value of 0.306 V vs Ag/AgNO_3_ in the presence of 15.0 equiv, and the voltammogram becomes less reversible (Figures 3, S24, and S25), likely owing to the slower rate of [Cu­(Me_2_-phen)_2_(^*t*^Bu-py)]^2+^/[Cu­(Me_2_-phen)_2_]^+^ reduction than [Cu­(Me_2_-phen)_2_(MeCN)]^2+^/[Cu­(Me_2_-phen)_2_]^+^. The CV of **1a·** ^***t***^ **Bu-py** shows similar behavior to the CV of **1a** in the presence of 1.0 equiv of ^*t*^Bu-py (Figures S26–S28). Open-circuit potential (*E*_OCP_) analysis of an equimolar MeCN solution of **1a** and **1b** (here, *E*_OCP_ ≈ *E*_1/2_)^60^ shows similar changes in *E*_OCP_ upon addition of ^*t*^Bu-py (Figures S29 and S30). Complex **2** displays a quasi-reversible Cu^II^/Cu^I^ redox wave with *E*_1/2_ = −0.560 V vs Ag/AgNO_3_ that is minimally affected in the presence of low concentrations of ^*t*^Bu-py but shifts slightly positively at higher concentrations (Figures S31–S34). In contrast, the CV of **3** shows a largely irreversible Cu^II^/Cu^I^ redox wave (Figures S35 and S36). Together, room-temperature electrochemical analysis corroborates UV–visible–NIR analysis and underscores the feasibility of using ^*t*^Bu-py as an external ligand to influence the redox behavior of **1a**. The employment of 1.5–15.0 equiv of ^*t*^Bu-py allows us to probe the impact of MeCN ligand displacement efficiency on α while retaining favorable quasi-reversible electrochemical behavior.

**Figure 3:**
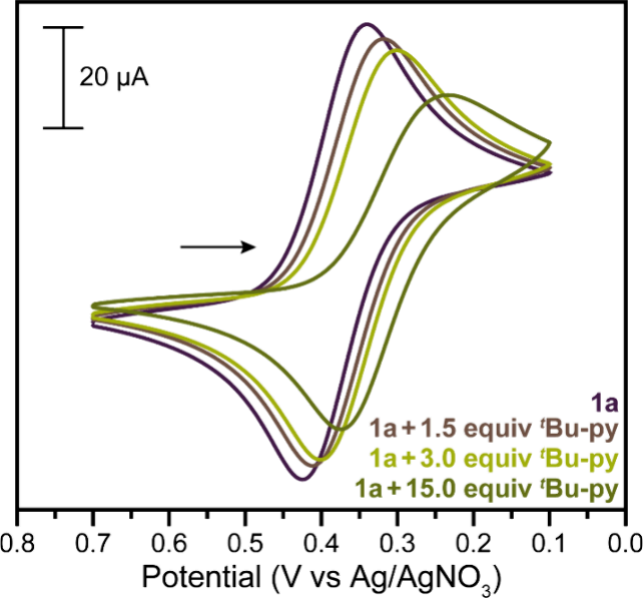
Cyclic voltammograms of 3 mM **1a** in the presence of 0, 1.5, 3.0, and 15.0 equiv of ^*t*^Bu-py collected at room temperature in MeCN containing 0.1 M (^*n*^Bu_4_N)­(PF_6_) supporting electrolyte using a 100 mV s^–1^ scan rate.

To probe the impact of redox-induced change in metal coordination number on the electrochemical properties of copper complexes and gain insights into the influence of such a mechanism stemming from a solvent molecule vs an external ligand, variable-temperature cyclic voltammetry measurements were undertaken for MeCN solutions of copper complexes in the absence and presence of ^*t*^Bu-py using an isothermal setup.^1^ The voltammograms collected in the temperature range 23–44 °C for **1a** with 0, 1.5, 3.0, and 15.0 equiv of ^*t*^Bu-py shift positively with increasing temperature (Figures 4 and S37), consistent with cationic redox couples.^11,20,27,28,32,34–36,40,42–44^ Temperature coefficients ranging from α = 1.70(6) mV °C^–1^ in the absence of ^*t*^Bu-py to α = 2.3(2) mV °C^–1^ with 15.0 equiv of ^*t*^Bu-py were obtained from plots of apparent half-wave potential vs temperature (Table 1, Figures S38–S41) and consideration of the temperature dependence of the Ag/AgNO_3_ reference electrode potential.^1,26,44,61^ Variable-temperature cyclic voltammetry analysis of **1a·** ^***t***^ **Bu-py** aligns with these results of increasing α with an increasing amount of ^*t*^Bu-py (Table 1, Figures S42–S45). In contrast, a similar analysis of **2** with 0, 3.0, and 15.0 equiv of ^*t*^Bu-py provides temperature coefficients that fall within error of one another (Table 1, Figures S46–S51). We note that the temperature sensitivity of *E*_1/2_ arises from a collection of processes that influence the geometric and electronic structure and/or solvation of the oxidized and/or reduced copper species^1,11,15,23,30^ and is minimally affected by changes in molecular diffusion and solution viscosity, as similar room-temperature diffusion coefficients^62,63^ were observed in the absence and presence of ^*t*^Bu-py across all compounds (Figures S52–S71, Tables S5–S7). These results underscore the critical role that change in coordination number during electron-transfer reactions plays in boosting α. Furthermore, the similar temperature coefficients obtained for **1a** and **2** in the absence of ^*t*^Bu-py suggest that the use of an external ligand as opposed to a (de)­coordinated solvent molecule is important for observing the enhancement, although influence from the different coordination at Cu^II^ in **1a** and **2** cannot be ruled out. Statistically identical temperature coefficients were obtained for **1a** and **1b** (Figures S72 and S73) in the absence of ^*t*^Bu-py, as well as for an equimolar solution with and without 15.0 equiv of ^*t*^Bu-py assessed by variable-temperature *E*_OCP_ analysis (Figures S74–S77), demonstrating reversible electrochemical behavior. The reversibility of the investigated Cu^II^/Cu^I^ redox systems is further evident from the good agreement between *E*_1/2_ of MeCN solutions before heating and after cooling back down (Figures S38–S41, S43, S47, S49, S51, S73, S75, and S77).

**Figure 4:**
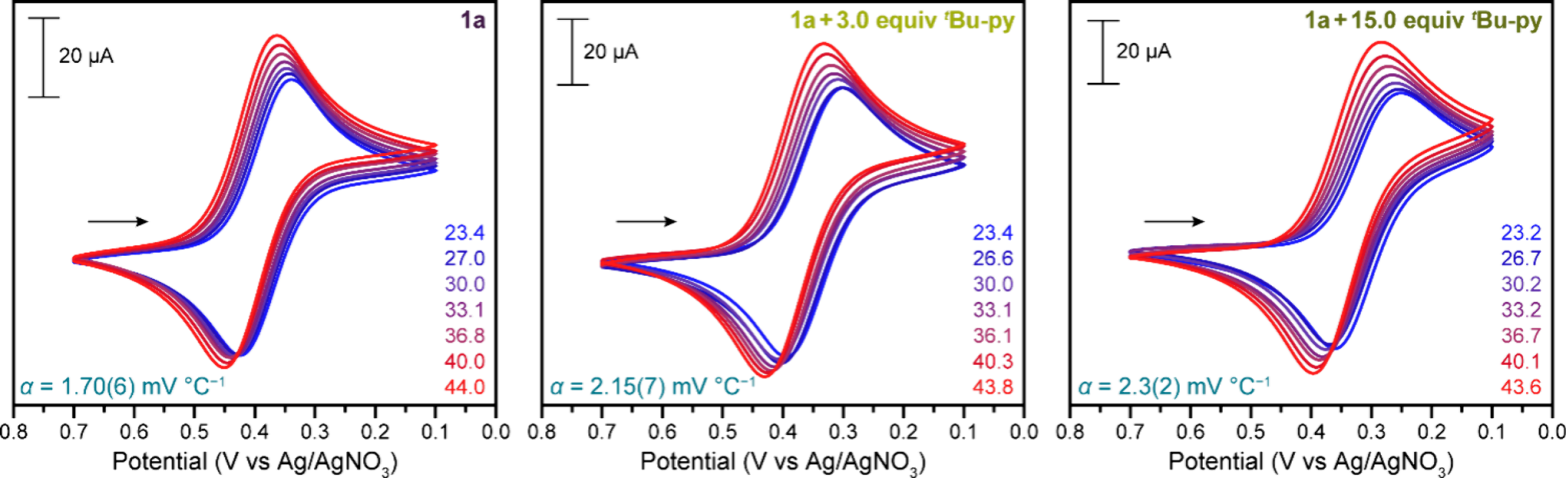
Cyclic voltammograms of 3 mM **1a** in the presence of 0, 3.0, and 15.0 equiv of ^*t*^Bu-py collected at variable temperatures (∼23–44 °C) in MeCN containing 0.1 M (^*n*^Bu_4_N)­(PF_6_) supporting electrolyte using a 100 mV s^–1^ scan rate. Average temperature coefficients are displayed in teal.

**Table 1:** Selected Electrochemical and Thermodynamic Parameters for Copper Complexes in MeCN Solutions^a^

Compound	Equiv of ^*t*^Bu-py	*E*_1/2_ ^b^ (V)	Δ*E*_p_ ^c^ (V)	α^d^ (mV °C^–1^)	Δ*S*_redox_ ^e^ (J K^–1^ mol^–1^)
**1a**	0	0.382	0.086	1.70(6)	164(6)
	1.5	0.361	0.096	2.02(7)	195(7)
	3.0	0.347	0.096	2.15(7)	207(7)
	15.0	0.306	0.110	2.3(2)	222(20)
**1b**	0	0.384	0.093	1.80(6)	174(6)
**1a·** ^***t***^ **Bu-py**	0	0.365	0.091	1.91(7)	184(7)
**2**	0	–0.560	0.091	1.66(7)	160(7)
	3.0	–0.559	0.094	1.67(6)	161(6)
	15.0	–0.550	0.103	1.6(1)	154(10)

a Solutions contained 3 mM copper complex and 0.1 M (^*n*^Bu_4_N)­(PF_6_) supporting electrolyte.

b Apparent half-wave potential vs Ag/AgNO_3_ at room temperature.

c Difference in apparent anodic and cathodic peak potentials at room temperature.

d Temperature coefficient calculated using eqs S9 and S10.

e Redox reaction entropy calculated using eq 1.

The temperature coefficient and corresponding Δ*S*_redox_ obtained for **1a** in the presence of 15.0 equiv of ^*t*^Bu-py are higher than values reported for other metal complexes with overall 2+/1+ charge-state change in homogeneous MeCN solution^28,44^ and surmount Cu^II^/Cu^0^ systems harnessing thermosensitive crystallization (Table S8).^64–66^ Moreover, a MeCN solution of **1b** with 15.0 equiv of ^*t*^Bu-py provides (after CV cycling to a stable *E*_1/2_) an even higher temperature coefficient of α = 2.6(2) mV °C^–1^ (Figures S78–S80), affording a record-high temperature sensitivity of *E*_1/2_ of the Cu^II^/Cu^I^ couple. The different results observed when starting from a solution containing predominantly Cu^II^ vs Cu^I^ species likely arise from influence of the Cu^II^/Cu^I^ concentration ratio on α.^66–69^

The foregoing results demonstrate that the temperature sensitivity of metal-based redox potentials may be enhanced using a redox-induced change in the coordination number strategy when facilitated by an external ligand. Efforts are underway to extend this initial proof-of-concept investigation to other metal and ligand systems and understand the effect of the concentrations of individual redox species.

## Supplementary Material



## Notes

Experimental details and additional characterization data, including NMR data, X-ray crystallographic data, UV–visible–NIR absorption spectra, and electrochemical data (PDF)
