# Supplementary material for: Enhancing Temperature Sensitivity of Redox Potential via Redox-Induced Change in Copper Coordination Number by an External Ligand
Source: J Am Chem Soc. 2026 Mar 31;148(13):13500–6. doi: 10.1021/jacs.5c21444 (PMC13067261; doi:10.1021/jacs.5c21444)
Supplement: Supplementary file 1 [file ja5c21444_si_001.pdf]

Supporting Information for:

Enhancing Temperature Sensitivity of Redox Potential via Redox-Induced Change in Copper Coordination Number by an External Ligand

Daniela Carmona-Pérez, William W. Brennessel, and *Agnes E. Thorarinsdottir**
Department of Chemistry, University of Rochester, Rochester, New York 14627, USA

*Correspondence to: agnes.thorarinsdottir@rochester.edu (A.E.T.)

Table of Contents

A. Experimental Section

General Considerations S5
Syntheses of Copper Complexes S5
Synthesis of $[Cu(Me_2\text{-phen})_2](PF_6)_2$ (**1a**) S5
Synthesis of $[Cu(Me_2\text{-phen})_2(^t\text{Bu-py})](PF_6)_2\cdot CH_2Cl_2$ (**1a•*t*Bu-py**) S6
Synthesis of $[Cu(Me_2\text{-phen})_2](PF_6)$ (**1b**) S6
Synthesis of $[Cu(terpy)_2](PF_6)_2$ (**2**) S7
Synthesis of $[Cu(^t\text{Bu-py})_4(MeCN)](PF_6)_2$ (**3**) S8
X-ray Structure Determination S8
Nomenclature of Copper Complexes S10
UV–Visible–NIR Absorption Spectroscopy S10
General Electrochemical Considerations S12
Diffusion Coefficient Determination S15
Temperature Coefficient Determination S15
Redox Reaction Entropy Determination S18
Other Physical Measurements S18

B. Supplementary Scheme

Scheme S1. Overview of the syntheses of copper complexes S19

C. Supplementary Figures

Figure S1. ^1H NMR spectrum of **1b** in CD_3CN S20
Figure S2. Crystal structure of **1a′** S21

Figure S3. Crystal structure of **1a•^{t}Bu-py** S22
Figure S4. UV–visible–NIR spectra of **1a** in MeCN (time dependence) S23
Figure S5. UV–visible–NIR spectra of **1a** w/wo ^{t}Bu-py in MeCN S24
Figure S6. Plot of λ_{max} for d–d band vs equiv of ^{t}Bu-py for data of **1a** S25
Figure S7. Plot of ε_{max} for d–d band vs equiv of ^{t}Bu-py for data of **1a** S26
Figure S8. UV–visible–NIR spectrum of ^{t}Bu-py in MeCN S27
Figure S9. UV–visible–NIR spectra of **1a•^{t}Bu-py** in MeCN (time dependence) S28
Figure S10. UV–visible–NIR spectra of **1a•^{t}Bu-py** w/wo ^{t}Bu-py in MeCN S29
Figure S11. Plot of λ_{max} for d–d band vs equiv of ^{t}Bu-py for data of **1a•^{t}Bu-py** S30
Figure S12. Plot of ε_{max} for d–d band vs equiv of ^{t}Bu-py for data of **1a•^{t}Bu-py** S31
Figure S13. UV–visible–NIR spectra of **1a** and **1a•^{t}Bu-py** w/wo ^{t}Bu-py in MeCN S32
Figure S14. UV–visible–NIR spectrum of Me_2-phen in MeCN S33
Figure S15. UV–visible–NIR spectra of Me_2-phen, **1a**, and **1b** in MeCN S34
Figure S16. Crystal structure of **3′** S35
Figure S17. Crystal structure of the cationic complex in **3′** S36
Figure S18. UV–visible–NIR spectra of **1a** w/wo ^{t}Bu-py, **1a•^{t}Bu-py**, and **3** in MeCN S37
Figure S19. UV–visible–NIR spectra of **1a** w/wo >50 equiv of ^{t}Bu-py and **3** in MeCN S38
Figure S20. UV–visible–NIR spectra of **1b** w/wo ^{t}Bu-py in MeCN S39
Figure S21. UV–visible–NIR spectra of **2** w/wo ^{t}Bu-py in MeCN S40
Figure S22. CVs of **1a** and **1b** in MeCN showing extended potential window S41
Figure S23. CVs of **1a** and **1b** in MeCN w/wo iR_u compensation S42
Figure S24. CVs of **1a** w/wo ^{t}Bu-py in MeCN S43
Figure S25. CVs of **1a** w/wo ^{t}Bu-py in MeCN showing extended potential window S44
Figure S26. CV of **1a•^{t}Bu-py** in MeCN showing extended potential window S45
Figure S27. CVs of **1a•^{t}Bu-py** in MeCN w/wo iR_u compensation S46
Figure S28. CVs of **1a** w/wo ^{t}Bu-py and **1a•^{t}Bu-py** in MeCN at room temperature S47
Figure S29. Plot of E_{OCP} vs time for 1:1 solution of **1a** and **1b** w/wo ^{t}Bu-py in MeCN S48
Figure S30. Plot of E_{OCP} vs equiv of ^{t}Bu-py in 1:1 solution of **1a** and **1b** in MeCN S49
Figure S31. CV of **2** in MeCN showing extended potential window S50
Figure S32. CVs of **2** in MeCN w/wo iR_u compensation S51
Figure S33. CVs of **2** w/wo ^{t}Bu-py in MeCN S52
Figure S34. CVs of **2** with 0, 1.5, 3.0, and 15.0 equiv of ^{t}Bu-py in MeCN S53
Figure S35. CV of **3** in MeCN showing extended potential window S54
Figure S36. CVs of **3** in MeCN w/wo iR_u compensation S55
Figure S37. Variable-temperature CVs of **1a** with 1.5 equiv of ^{t}Bu-py in MeCN S56
Figure S38. Plot of $E_{1/2}$ vs T for **1a** in MeCN S57
Figure S39. Plot of $E_{1/2}$ vs T for **1a** with 1.5 equiv of ^{t}Bu-py in MeCN S58
Figure S40. Plot of $E_{1/2}$ vs T for **1a** with 3.0 equiv of ^{t}Bu-py in MeCN S59
Figure S41. Plot of $E_{1/2}$ vs T for **1a** with 15.0 equiv of ^{t}Bu-py in MeCN S60
Figure S42. Variable-temperature CVs of **1a•^{t}Bu-py** in MeCN S61

Figure S43. Plot of $E_{1/2}$ vs T for **1a•^{t}Bu-py** in MeCN S62
Figure S44. CVs of **1a** w/wo ^{t}Bu-py and **1a•^{t}Bu-py** in MeCN at lowest temperature S63
Figure S45. CVs of **1a** w/wo ^{t}Bu-py and **1a•^{t}Bu-py** in MeCN at highest temperature S64
Figure S46. Variable-temperature CVs of **2** in MeCN S65
Figure S47. Plot of $E_{1/2}$ vs T for **2** in MeCN S66
Figure S48. Variable-temperature CVs of **2** with 3.0 equiv of ^{t}Bu-py in MeCN S67
Figure S49. Plot of $E_{1/2}$ vs T for **2** with 3.0 equiv of ^{t}Bu-py in MeCN S68
Figure S50. Variable-temperature CVs of **2** with 15.0 equiv of ^{t}Bu-py in MeCN S69
Figure S51. Plot of $E_{1/2}$ vs T for **2** with 15.0 equiv of ^{t}Bu-py in MeCN S70
Figure S52. Variable-scan-rate CV of **1a** in MeCN S71
Figure S53. Randles–Ševčík plot for **1a** in MeCN S72
Figure S54. Variable-scan-rate CV of **1a** with 3.0 equiv of ^{t}Bu-py in MeCN S73
Figure S55. Randles–Ševčík plot for **1a** with 3.0 equiv of ^{t}Bu-py in MeCN S74
Figure S56. Variable-scan-rate CV of **1a** with 15.0 equiv of ^{t}Bu-py in MeCN S75
Figure S57. Randles–Ševčík plot for **1a** with 15.0 equiv of ^{t}Bu-py in MeCN S76
Figure S58. Variable-scan-rate CV of **1b** in MeCN S77
Figure S59. Randles–Ševčík plot for **1b** in MeCN S78
Figure S60. Variable-scan-rate CV of **1b** with 3.0 equiv of ^{t}Bu-py in MeCN S79
Figure S61. Randles–Ševčík plot for **1b** with 3.0 equiv of ^{t}Bu-py in MeCN S80
Figure S62. Variable-scan-rate CV of **1b** with 15.0 equiv of ^{t}Bu-py in MeCN S81
Figure S63. Randles–Ševčík plot for **1b** with 15.0 equiv of ^{t}Bu-py in MeCN S82
Figure S64. Variable-scan-rate CV of **2** in MeCN S83
Figure S65. Randles–Ševčík plot for **2** in MeCN S84
Figure S66. Variable-scan-rate CV of **2** with 3.0 equiv of ^{t}Bu-py in MeCN S85
Figure S67. Randles–Ševčík plot for **2** with 3.0 equiv of ^{t}Bu-py in MeCN S86
Figure S68. Variable-scan-rate CV of **2** with 15.0 equiv of ^{t}Bu-py in MeCN S87
Figure S69. Randles–Ševčík plot for **2** with 15.0 equiv of ^{t}Bu-py in MeCN S88
Figure S70. Variable-scan-rate CV of **1a•^{t}Bu-py** in MeCN S89
Figure S71. Randles–Ševčík plot for **1a•^{t}Bu-py** in MeCN S90
Figure S72. Variable-temperature CVs of **1b** in MeCN S91
Figure S73. Plot of $E_{1/2}$ vs T for **1b** in MeCN S92
Figure S74. Plot of E_{OCP} vs time for **1a**:**1b** (1:1) in MeCN at different T S93
Figure S75. Plot of E_{OCP} vs T for **1a**:**1b** (1:1) in MeCN S94
Figure S76. Plot of E_{OCP} vs time for **1a**:**1b** (1:1) with ^{t}Bu-py in MeCN at different T S95
Figure S77. Plot of E_{OCP} vs T for **1a**:**1b** (1:1) with ^{t}Bu-py in MeCN S96
Figure S78. Temperature coefficients of copper complexes w/wo ^{t}Bu-py in MeCN S97
Figure S79. Variable-temperature CVs of **1b** with 15.0 equiv of ^{t}Bu-py in MeCN S98
Figure S80. Plot of $E_{1/2}$ vs T for **1b** with 15.0 equiv of ^{t}Bu-py in MeCN S99

D. Supplementary Tables

Table S1. Crystallographic data for **1a′**, **1a•^{t}Bu-py**, and **3′** S100
Table S2. Selected structural metrics for **1a′**, **1a•^{t}Bu-py**, **1b**, and **2** S101
Table S3. Ratio of ^{t}Bu-py binding to **1a** in MeCN S102
Table S4. Selected structural metrics for **3′** S103
Table S5. Diffusion coefficients for Cu complexes in MeCN (reversible couple) S104
Table S6. Diffusion coefficients for Cu complexes in MeCN (irreversible couple) S105
Table S7. Diffusion coefficients for **1b** S106
Table S8. Summary of selected temperature coefficients from the literature S107

E. References S108

A. Experimental Section

General Considerations

Unless otherwise specified, the manipulations described below were carried out under ambient atmosphere and temperature. Air- and water-free manipulations were performed under a dry dinitrogen atmosphere in MBraun MB 200B and MBraun Labmaster SP gloveboxes or using standard Schlenk line techniques. Glassware was oven-dried at 125 °C for at least 4 h and allowed to cool in an evacuated antechamber prior to use in gloveboxes. Acetonitrile (MeCN), dichloromethane (CH_2Cl_2), diethyl ether (Et_2O), and methanol (MeOH) used inside gloveboxes and on the Schlenk line were dried using a commercial solvent purification system from Pure Process Technology and stored over 3 Å molecular sieves prior to use. MeCN, CH_2Cl_2, Et_2O, ethanol (EtOH), and MeOH solvents used outside gloveboxes were purchased from Sigma–Aldrich and Fisher Scientific. Ultrapure water (18.2 MΩ cm) was obtained from an Arium Mini water purification system from Sartorius. Deuterated solvents were purchased from Cambridge Isotope Laboratories. (^{n}Bu_4N)(PF_6) used for electrochemical measurements was purchased from A2B Chem LLC and recrystallized twice from ethanol and dried under vacuum for at least 12 h prior to use. 4-*tert*-Butylpyridine (^{t}Bu-py) was purchased from Sigma–Aldrich and deoxygenated using the freeze-pump-thaw method on the Schlenk line prior to use inside gloveboxes. All other reagents were purchased from commercial vendors and used without further purification.

Syntheses of Copper Complexes

An overview of the syntheses of the copper complexes used in this study is provided in Scheme S1. Below are detailed experimental procedures and characterization data.

Synthesis of [Cu(Me_2-phen)$_2$](PF_6)$_2$ (1a)

This compound was synthesized following a modified literature procedure.[1] A colorless solution of 2,9-dimethyl-1,10-phenanthroline (Me_2-phen) (266 mg, 1.3 mmol) in EtOH (10 mL) was added to a blue solution of $Cu(NO_3)_2 \cdot 3H_2O$ (140 mg, 0.58 mmol) in EtOH (20 mL) to yield a green solution. This solution was stirred at 20 °C for 1 h, during which time a green precipitate formed. To redissolve the solid, 10 mL of MeCN was added to the mixture, producing a green solution. Subsequently, a colorless solution of KPF_6 (1.07 g, 5.8 mmol) in ultrapure water (20 mL) was added, resulting in a green solution that was stirred at 20 °C for 1 h. Afterwards, the volume of the reaction mixture was reduced to 10 mL under reduced pressure, resulting in the formation of a pale green solid. The solid was collected by vacuum filtration, washed with ultrapure water (2 × 10 mL) and Et_2O (2 × 20 mL), and dried in air. According to single-crystal X-ray diffraction analysis,

this solid corresponds to a complex with one nitrate ion coordinated to the Cu^{II} center. To remove the coordinated nitrate and complete the desired anion exchange of $(NO_3)^-$ for $(PF_6)^-$, the green solid was redissolved in MeCN (20 mL), and a colorless solution of KPF_6 (1.07 g, 5.8 mmol) in ultrapure water (20 mL) was added. The resulting green solution was stirred at 20 °C for 1 h. After this time, the MeCN solvent was removed under reduced pressure, yielding a green aqueous suspension. The green solid was collected by vacuum filtration, washed with ultrapure water (2 × 10 mL) and Et_2O (2 × 20 mL), and dried in air. Diffusion of Et_2O vapor into a concentrated solution of the green solid in MeCN (5 mL) afforded $[Cu(Me_2\text{-phen})_2(MeCN)](PF_6)_2$ (**1a•MeCN**) as a green crystalline solid. This solid was washed with Et_2O (20 mL) and dried under reduced pressure for 6 h to give **1a** (390 mg, 87%) as a purple crystalline solid. Note that the change in the color of the compound after drying is due to the decoordination of a MeCN molecule from the Cu^{II} center, and thus a reduction in the Cu coordination number from five to four. Anal. Calcd for $C_{28}H_{24}CuF_{12}N_4P_2$: C, 43.67; H, 3.14; N, 7.28%. Found: C, 43.81; H, 3.12; N, 7.23%. UV–visible–NIR absorption spectrum (1.0 mM; MeCN, 20 °C): ~400 nm (ε = 170 M^{-1} cm^{-1}), ~725 nm (ε = 130 M^{-1} cm^{-1}), ~990 nm (ε = 145 M^{-1} cm^{-1}). Slow diffusion of Et_2O vapor into a concentrated solution of **1a** in MeCN afforded green block-shaped crystals of $[Cu(Me_2\text{-phen})_2(MeCN)](PF_6)_2\cdot MeCN$ (**1a′**) suitable for single-crystal X-ray diffraction analysis (Figures 1 and S2).

Synthesis of $[Cu(Me_2\text{-phen})_2(^t\text{Bu-py})](PF_6)_2\cdot CH_2Cl_2$ (1a•^{t}Bu-py)

A colorless solution of 4-*tert*-butylpyridine (^{t}Bu-py) (56 μL, 0.38 mmol) in CH_2Cl_2 (5 mL) was added to a purple suspension of **1a** (98 mg, 0.13 mmol) in CH_2Cl_2 (5 mL), yielding a green solution. This solution was stirred at 20 °C for 30 min, then the volume of the green solution was reduced to half of its original volume under reduced pressure. Diffusion of Et_2O vapor into the resulting concentrated green CH_2Cl_2 solution afforded a green crystalline solid, which was washed with Et_2O (20 mL) and dried under reduced pressure for 6 h to give **1a•^{t}Bu-py** (94 mg, 73%) as a green crystalline solid. Anal. Calcd for $C_{38}H_{39}Cl_2CuF_{12}N_5P_2$: C, 46.09; H, 3.97; N, 7.07%. Found: C, 45.88; H, 3.88; N, 6.88%. UV–visible–NIR absorption spectrum (1.2 mM; MeCN, 20 °C): ~450 nm (ε = 135 M^{-1} cm^{-1}), ~723 nm (ε = 160 M^{-1} cm^{-1}), ~990 nm (ε = 135 M^{-1} cm^{-1}). Slow diffusion of Et_2O vapor into a concentrated solution of **1a•^{t}Bu-py** in CH_2Cl_2 afforded blue-green plate-shaped crystals of **1a•^{t}Bu-py** suitable for single-crystal X-ray diffraction analysis (Figures 1 and S3).

Synthesis of $[Cu(Me_2\text{-phen})_2](PF_6)$ (1b)

This compound was synthesized following a three-step literature procedure.[2]

Step 1. Synthesis of $[Cu(Me_2\text{-phen})_2(Cl)]Cl$

To a green solution of $CuCl_2$ (250 mg, 1.9 mmol) in EtOH (120 mL), Me_2-phen (852 mg, 4.1 mmol) was added as a solid to yield a green solution. A green precipitate formed in the solution after stirring at 20 °C for 1 h. The green solid was collected by vacuum filtration, washed with cold EtOH (2 × 10 mL) and Et_2O (2 × 20 mL), and dried in air to give [Cu(Me_2-phen)$_2$(Cl)]Cl (814 mg, 78%) as a green solid.

Step 2. Synthesis of [Cu(Me_2-phen)$_2$(Cl)](PF_6)

To a green solution of [Cu(Me_2-phen)$_2$(Cl)]Cl (1.30 g, 2.4 mmol) in a 1:2 EtOH/H_2O mixture (volume ratio, 350 mL), (NH_4)(PF_6) (7.50 g, 46.0 mmol) was added as a white solid to yield a green suspension. This suspension was stirred at 20 °C for 1 h. Afterwards, the green solid was collected by vacuum filtration, washed thoroughly with ultrapure water (>50 mL), followed by Et_2O (2 × 20 mL), and dried in air to give [Cu(Me_2-phen)$_2$(Cl)](PF_6) (1.20 g, 76%) as a green solid.

*Step 3. Synthesis of [Cu(Me_2-phen)$_2$](PF_6) (**1b**)*

To a green solution of [Cu(Me_2-phen)$_2$(Cl)](PF_6) (1.20 g, 1.8 mmol) in MeCN, L-ascorbic acid (3.2 g, 18.2 mmol) was added as a white solid to yield an orange-red suspension. This suspension was stirred at 20 °C for 1 h. Afterwards, the excess of L-ascorbic acid was removed by gravimetric filtration. The orange-red filtrate was collected, and the MeCN solvent was removed under reduced pressure, yielding an orange-red solid. The solid was suspended in 100 mL of ultrapure water and sonicated to remove traces of L-ascorbic acid. The resulting orange-red solid was collected by vacuum filtration, washed thoroughly with ultrapure water (>50 mL), followed by Et_2O (2 × 20 mL), and dried under reduced pressure for >4 h to give an orange-red solid. Slow evaporation of a concentrated solution of the orange-red solid in CH_2Cl_2 yielded an orange-red crystalline solid, which was dried under reduced pressure for 6 h to give **1b** (934 mg, 83%). Anal. Calcd for $C_{28}H_{24}CuF_6N_4P$: C, 53.80; H, 3.87; N, 8.96%. Found: C, 53.53; H, 3.75; N, 8.96%. ^1H NMR (400 MHz, CD_3CN, 22 °C): δ 8.57 (d, 4H), 8.09 (s, 4H), 7.81 (d, 4H), 2.40 (s, 12H) (Figure S1). UV–visible–NIR absorption spectrum (0.1 mM; MeCN, 20 °C): ~320 nm (ε = 5120 M^{-1} cm^{-1}), ~455 nm (ε = 7470 M^{-1} cm^{-1}). Slow evaporation of a concentrated solution of **1b** in CH_2Cl_2 afforded orange-red needle-shaped crystals of **1b** suitable for single-crystal X-ray diffraction analysis. The crystals matched a previously reported crystal structure, CCDC 228944 (Figure 1).[3]

Synthesis of [Cu(terpy)$_2$](PF_6)$_2$ (2)

This compound was synthesized following a modified literature procedure.[4] A blue solution of $Cu(NO_3)_2·3H_2O$ (44 mg, 0.18 mmol) in dry MeOH (10 mL) was added under a positive flow of N_2 gas to a 50-mL Schlenk flask containing a yellow solution of 2,2′:6′,2″-terpyridine (terpy) (90 mg, 0.39 mmol) in dry MeOH (10 mL), yielding a green solution. This solution was stirred at 20 °C for 3 h, during which time the color of the solution turned from green to teal. To this teal solution,

$(NH_4)(PF_6)$ (303 mg, 1.9 mmol) was added as a white solid, resulting in the formation of a blue precipitate. The obtained blue suspension was stirred at 20 °C for 30 min. After this time, the blue solid was collected by vacuum filtration, washed with cold MeOH (2 × 10 mL) and Et_2O (2 × 20 mL), and dried in air. Diffusion of Et_2O vapor into a concentrated solution of the blue solid in MeCN (5 mL) afforded a blue crystalline solid, which was washed with Et_2O (20 mL) and dried under reduced pressure for 6 h to give **2** (106 mg, 72%) as a blue crystalline solid. Anal. Calcd for $C_{30}H_{22}CuF_{12}N_6P_2$: C, 43.94; H, 2.70; N, 10.25%. Found: C, 44.10; H, 2.82; N, 10.27%. UV–visible–NIR absorption spectrum (2.0 mM; MeCN, 20 °C): ~486 nm (ε = 10 M^{-1} cm^{-1}), ~556 nm (ε = 15 M^{-1} cm^{-1}), ~687 nm (ε = 70 M^{-1} cm^{-1}). Slow diffusion of Et_2O vapor into a concentrated solution of **2** in MeCN afforded green crystals of **2** suitable for single-crystal X-ray diffraction analysis. The crystals matched a previously reported crystal structure, CCDC 1108163 (Figure 1).[5]

Synthesis of [Cu(^{t}Bu-py)$_4$(MeCN)](PF_6)$_2$ (3)

This compound was synthesized following a modified literature procedure.[1] A colorless solution of 4-*tert*-butylpyridine (^{t}Bu-py) (372 µL, 2.5 mmol) in EtOH (15 mL) was added to a blue solution of $Cu(NO_3)_2{\cdot}3H_2O$ (123 mg, 0.51 mmol) in EtOH (15 mL), yielding a violet solution. This solution was stirred at 20 °C for 30 min, then a colorless solution of KPF_6 (1.88 g, 10.2 mmol) in ultrapure water (30 mL) was added, resulting in the formation of a violet precipitate. The solid was collected by vacuum filtration, washed thoroughly with ultrapure water (>50 mL) and EtOH (>50 mL), and dried in air. Slow evaporation of a concentrated solution of the violet solid in MeCN afforded a violet-blue powder that was dried under reduced pressure for 6 h to give **3** (245 mg, 51%) as a violet-blue crystalline solid. Anal. Calcd for $C_{38}H_{55}CuF_{12}N_5P_2$: C, 48.79; H, 5.93; N, 7.49%. Found: C, 48.74; H, 5.44; N, 7.39%. UV–visible–NIR absorption spectrum (5.0 mM; MeCN, 20 °C): ~418 nm (ε = 15 M^{-1} cm^{-1}), ~580 nm (ε = 80 M^{-1} cm^{-1}). Slow evaporation of a concentrated solution of **3** in a 1:1:1 EtOH/Et_2O/MeCN mixture (volume ratio) at 4 °C afforded violet-blue plate-shaped crystals of [*trans*-Cu(^{t}Bu-py)$_4$(MeCN)$_2$](PF_6)$_2{\cdot}Et_2O{\cdot}$solvent (**3′**) suitable for single-crystal X-ray diffraction analysis (Figures S16 and S17). Note that the term "solvent" in the formula above denotes a combination of crystallographically disordered EtOH, Et_2O, and MeCN molecules (vide infra). Further note that upon dissolution of **3** in a MeCN-containing solution, a second molecule of MeCN binds to the Cu^{II} center, as indicated in the crystal structure of **3′**, thus changing the Cu coordination number from five to six.

X-ray Structure Determination

Single crystals of **1a′**, **1a•^{t}Bu-py**, **1b**, **2**, and **3′** were placed onto a nylon loop and mounted on a Rigaku XtaLAB Synergy-S Dualflex diffractometer equipped with a HyPix-6000HE HPC area

detector for data collection at 100 K for **1a′**, **1b**, **2**, and **3′**, and 173 K for **1a•*t*Bu-py**. A preliminary set of cell constants and an orientation matrix were calculated from a small sampling of reflections.[6] For each crystal, a short pre-experiment was run, from which an optimal data collection strategy was determined. Note that the pre-experiment runs for crystals of **1b** and **2** matched previously reported crystallographic data (CCDC 228944[3] and 1108163[5] for **1b** and **2**, respectively). The full data collections were carried out using a PhotonJet Cu X-ray source. Raw data were integrated and corrected for Lorentz and polarization effects with CrysAlis^{Pro}.[6] Absorption corrections were applied using the multiscan method within CrysAlis^{Pro}.[6] Space group assignments were determined based on systematic absences (**1a′**) or just intensity statistics (**1a•*t*Bu-py** and **3′**). Structures were solved using the dual-space algorithm of *SHELXT*[7] and refined using *SHELXL*[8] operated within the *Olex2* interface.[9] Most or all non-hydrogen atoms were assigned directly from the structure solution. Full-matrix least squares and difference Fourier cycles were then performed, which located any remaining non-hydrogen atoms based on electron density and proposed molecular formula. All hydrogen atoms were placed at calculated positions using suitable riding models and refined using isotropic displacement parameters derived from their parent atoms. Thermal parameters for all non-hydrogen atoms were refined anisotropically.

In the crystal structure of **1a′**, one of the $(PF_6)^-$ anions is modeled as disordered over two positions in a ratio of 0.86:0.14. In the crystal structure of **1a•*t*Bu-py**, the two $(PF_6)^-$ anions are modeled as disordered over two positions each in ratios of 0.51:0.49 and 0.73:0.27. In the crystal structure of **3′**, reflection contributions from highly disordered solvent molecules were fixed and added to the calculated structure factors using the SQUEEZE routine of the program *PLATON*.[10] A void volume of 725 Å^3, with 166 electrons, was estimated per unit cell and ascribed to a combination of EtOH, Et_2O, and MeCN molecules. Due to this disorder, the nomenclature of the single crystals of **3′** is noted as [*trans*-Cu(^{t}Bu-py)$_4$(MeCN)$_2$]$(PF_6)_2$·Et_2O·solvent. Thus, all calculated quantities that derive from the molecular formula are known to be inaccurate. Three ^{t}Bu groups are modeled as disordered over two positions each in ratios of 0.54:0.46, 0.50:0.50, and 0.77:0.23. One of the $(PF_6)^-$ anions is modeled as disordered over two positions in a ratio of 0.59:0.41. Crystallographic data for **1a′**, **1a•*t*Bu-py**, and **3′**, and the details of data collection are listed in Table S1. These structures have been deposited in the CCDC under deposition numbers 2505012, 2505013, and 2505014 for **1a′**, **1a•*t*Bu-py**, and **3′**, respectively. Selected mean interatomic distances and angles and structural parameters for **1a′**, **1a•*t*Bu-py**, **1b**, **2**, and **3′** are further provided in Tables S2 and S4.

Nomenclature of Copper Complexes

As outlined in the sections above and Scheme S1, copper compounds discussed in this work are given labels of "**1**", "**2**", or "**3**" depending on their coordination environment, and are further separated into "**a**" or "**b**" based on the oxidation state of Cu. Coordination of MeCN solvent or external *ᵗ*Bu-py ligand is indicated by "•". To illustrate, compounds bearing two Me_2-phen ligands are labelled "**1**", those with two terpy ligands as "**2**", and those with four ^{t}Bu-py ligands as "**3**". To further distinguish between compounds bearing two Me_2-phen ligands, "**1a**" and "**1b**" denote Cu^{II} and Cu^{I} complexes, respectively. Coordination of a fifth ligand to the Cu^{II} complexes is indicated by "•", providing the compounds **1a•MeCN** and **1a•^{t}Bu-py**. For instances where single-crystal structures of the copper complexes contain additional solvent molecules compared to the chemical formulas obtained from elemental analysis on bulk samples, the single-crystal compounds are given a slightly different label that contains the prime symbol to simultaneously acknowledge the similarities and differences between the bulk and single-crystal samples. As such, compound $[Cu(Me_2\text{-phen})_2(MeCN)](PF_6)_2$ is labelled **1a•MeCN** and the single-crystal compound $[Cu(Me_2\text{-phen})_2(MeCN)](PF_6)_2 \cdot MeCN$ as **1a′**. Similarly, $[Cu(^t\text{Bu-py})_4(MeCN)](PF_6)_2$ is labelled **3** and the single-crystal compound $[\textit{trans}\text{-}Cu(^t\text{Bu-py})_4(MeCN)_2](PF_6)_2 \cdot Et_2O \cdot$solvent as **3′**.

UV–Visible–NIR Absorption Spectroscopy

Solution UV–visible–NIR absorption spectra were collected at 20 °C on an Agilent Cary 60 UV–visible–NIR spectrophotometer. Spectra were collected in the 300–1000 nm range using 2400 nm s^{-1} scan rate for 0.1–500 mM samples of complexes **1a**, **1a•^{t}Bu-py**, **1b**, **2**, and **3**, and ligands Me_2-phen, terpy, and ^{t}Bu-py in dry MeCN. All data were collected in a quartz cuvette and treated with a background correction of the MeCN solvent. Samples of complexes **1a**, **1a•^{t}Bu-py**, **1b**, and **2** were collected in the absence and presence of variable amount of ^{t}Bu-py (equiv of ^{t}Bu-py is based on the total copper concentration in the sample solution). For the ^{t}Bu-py titration experiments, a concentrated stock solution of ^{t}Bu-py was prepared in dry MeCN and added to a solution of the given copper complex in dry MeCN using a pre-calibrated micropipette under ambient conditions. After each addition, the sample solution was mixed in the cuvette sample holder using the micropipette prior to data collection.

The UV–visible–NIR absorption data obtained from titration of ^{t}Bu-py into a solution of **1a** in dry MeCN was used to estimate an effective equilibrium constant (K_{eq}) for binding of ^{t}Bu-py to the Cu^{II} center in **1a**, i.e., the exchange of MeCN solvent molecule for ^{t}Bu-py ligand (eq S1).

$$\mathbf{1a{\bullet}MeCN} + {}^t\text{Bu-py} \rightleftharpoons \mathbf{1a{\bullet}{}^t Bu\text{-}py} + \text{MeCN} \qquad \text{(S1)}$$

Note that in eq S1 and the following discussion in this subsection, we use the notation **1a•^{t}Bu-py** to represent **1a** with ^{t}Bu-py bound while ignoring co-crystallization with CH_2Cl_2. As MeCN is present in large excess as the reaction solvent and isosbestic points are observed (vide infra), suggesting that only two Cu-containing species are involved in the dissociation–association equilibrium, K_{eq} may be written as follows:

$$K_{eq} = \frac{[\mathbf{1a{\bullet}}^t\mathbf{Bu\text{-}py}]}{[\mathbf{1a{\bullet}MeCN}]\cdot[^t\mathbf{Bu\text{-}py}]} \quad \text{(S2)}$$

Furthermore, as ^{t}Bu-py does not absorb light above 400 nm (vide infra), the observed absorbance is a sum of the contribution from **1a•MeCN** and **1a•^{t}Bu-py**. For a fixed wavelength (λ) at which addition of ^{t}Bu-py results in significant changes in absorbance (and molar absorptivity), and a fixed pathlength of 1 cm, the following equation may thus be derived:

$$A_\lambda = \varepsilon_{\mathbf{1a{\bullet}MeCN}}\cdot[\mathbf{1a{\bullet}MeCN}] + \varepsilon_{\mathbf{1a{\bullet}}t\mathbf{Bu\text{-}py}}\cdot[\mathbf{1a{\bullet}}^t\mathbf{Bu\text{-}py}] \quad \text{(S3)}$$

The total concentrations of Cu^{II} and ^{t}Bu-py in solution are given by:

$$[Cu^{II}]_{total} = [\mathbf{1a{\bullet}MeCN}] + [\mathbf{1a{\bullet}}^t\mathbf{Bu\text{-}py}] \quad \text{(S4)}$$

$$[^t\text{Bu-py}]_{total} = [^t\text{Bu-py}] + [\mathbf{1a{\bullet}}^t\mathbf{Bu\text{-}py}] \quad \text{(S5)}$$

Combining equations S3 and S4 gives:[11]

$$A_\lambda = \varepsilon_{\mathbf{1a{\bullet}MeCN}}\cdot[Cu^{II}]_{total} + (\varepsilon_{\mathbf{1a{\bullet}}t\mathbf{Bu\text{-}py}} - \varepsilon_{\mathbf{1a{\bullet}MeCN}})\cdot[\mathbf{1a{\bullet}}^t\mathbf{Bu\text{-}py}] \quad \text{(S6)}$$

Assuming that complete exchange of MeCN for ^{t}Bu-py is achieved upon addition of 15–40 equiv of ^{t}Bu-py to an MeCN solution of **1a** (vide infra), values of $\varepsilon_{\mathbf{1a{\bullet}MeCN}}$ and $\varepsilon_{\mathbf{1a{\bullet}}t\mathbf{Bu\text{-}py}}$ may be obtained for a given wavelength from the measured molar absorptivity values at 0 equiv and 15–40 equiv (average) of ^{t}Bu-py, respectively. At 725 nm, which is the wavelength of maximum absorbance for the d–d transition band of **1a•MeCN** in the absence of ^{t}Bu-py (vide infra), values of $\varepsilon_{\mathbf{1a{\bullet}MeCN}}$ = 131 M^{-1} cm^{-1} and $\varepsilon_{\mathbf{1a{\bullet}}t\mathbf{Bu\text{-}py}}$ = 214 M^{-1} cm^{-1} were obtained. With these values in hand, along with a known total concentration of Cu^{II} (as defined by the mass of **1a** used to prepare the initial sample solution), values of [**1a•^{t}Bu-py**], and subsequently K_{eq}, may be derived as a function of added equiv of ^{t}Bu-py. Considering the range of 0.6–4.0 equiv of ^{t}Bu-py, which provides a ^{t}Bu-py binding ratio ([**1a•^{t}Bu-py**]:$[Cu^{II}]_{total}$) of 0.2–0.8, and thus the highest reliability of the calculated K_{eq} value,[11] an average value of K_{eq} = 8(2) × 10^2 M^{-1} was obtained. Similar analysis at a wavelength of 847 nm provided K_{eq} = 9(2) × 10^2 M^{-1}. These values of K_{eq} align with prior reports.[12] A summary of the

binding ratio as a function of equiv of ^{t}Bu-py added is provided for data collected at both wavelengths (725 nm and 847 nm) in Table S3.

General Electrochemical Considerations

All electrochemical experiments were carried out under a dry dinitrogen atmosphere in a MBraun Labmaster SP or a MBraun MB 200B glovebox using a CH Instruments 760E electrochemical workstation. Sample solutions of complexes **1a**, **1a•^{t}Bu-py**, **1b**, **2**, and **3** were prepared in dry MeCN containing 0.1 M (^{n}Bu$_4$N)(PF$_6$) supporting electrolyte. For sample solutions containing additional ^{t}Bu-py, the equiv of ^{t}Bu-py is based on the total copper concentration in the sample solution. For the ^{t}Bu-py titration experiments, a concentrated stock solution of ^{t}Bu-py was prepared in dry MeCN and added to a solution of the given copper complex in dry MeCN using a pre-calibrated micropipette. Sample solutions were stirred for at least one minute prior to data collection. Unless otherwise specified, experiments were carried out using a 10-mL glass beaker or a 20-mL scintillation vial, a 3-mm diameter glassy carbon working electrode (CH Instruments, Inc.), a non-aqueous Ag/AgNO$_3$ reference electrode (CH Instruments, Inc.) filled with a MeCN solution containing 0.01 M AgNO$_3$ and 0.1 M (^{n}Bu$_4$N)(PF$_6$), and a counter electrode composed of a platinum mesh (99.9%, 52 mesh woven from 0.1-mm diameter wire, Fisher Scientific) attached to a platinum wire (99.95%, 0.5-mm diameter, Fisher Scientific). Before use, the glassy carbon working electrode was polished with alumina powder (0.05 μm, Allied High Tech Products, Inc.) on a microfiber polishing cloth, and the platinum counter electrode was cleaned by soaking in concentrated nitric acid (ACS Plus, 15.8 N, Fisher Scientific) followed by drying with a butane flame. All electrochemical glassware was washed with concentrated nitric acid (ACS Plus, 15.8 N, Fisher Scientific) and ultrapure water before experiments, and oven-dried for at least 4 h prior to use. Cyclic voltammetry (CV) measurements were conducted using a scan rate of 25–400 mV s^{-1}. The second CV cycle is displayed in all cases. All recorded electrode potentials are referenced to the Ag/AgNO$_3$ electrode. Drifts in the reference electrode potential were checked routinely using the ferrocenium/ferrocene (Fc^{+}/Fc) redox couple ($E_{1/2}$ = 0.091(1) V vs Ag/AgNO$_3$). Uncompensated solution resistance (R_u) was determined to be ~20–60 Ω using the potential step method (amplitude of 50 mV) around the open-circuit potential (E_{OCP}). Note, however, that all electrode potentials are reported without applying iR_u compensation as identical CV traces were obtained with and without applying such compensation in manual mode (Figures S23, S27, S32, and S36). All electrochemical data are plotted according to the polarographic convention, where negative currents correspond to anodic reactions (oxidation) and positive currents correspond to

cathodic reactions (reduction). Independently prepared samples of each type were measured to ensure reproducibility. The provided data are representative examples.

Our definitions of several terms used to describe "potential" in the manuscript and Supporting Information along with examples of use are provided below for clarity.

- *Redox potential*: While the term "redox potential" does not have a universally accepted or standardized definition in the literature, we use it herein for instances that equally apply to reduction potential and oxidation potential. For example, "the temperature sensitivity of molecular redox potentials" refers to the temperature sensitivity of both the reduction potential and the oxidation potential of a molecule, as the temperature sensitivity values should be identical for an ideal, reversible redox couple.

- *Electrode potential*: The free-energy change divided by the electron charge associated with moving an electron and any associated species from a reference electrode to the working electrode.[13] This term is used in the experimental section above to describe the recorded potential values in CV measurements against the Ag/$AgNO_3$ reference electrode potential (vide supra).

- *Reference electrode potential*: The potential of an electrode that has a stable and well-known electrode potential that provides a constant reference point to measure the potential of a working electrode against.[14] In our studies, a non-aqueous Ag/$AgNO_3$ reference electrode was used for all experiments (vide supra), and it is denoted as E_{ref} (vide infra).

- *Open-circuit potential*: The electrode potential when negligible net current flows between the working and reference electrodes.[13,14] The open-circuit potential is denoted as E_{OCP} and used herein to enable a study of the similarities or differences between the temperature sensitivity of the Cu^{II}/Cu^{I} redox couple assessed by CV and an equimolar solution of Cu^{II} and Cu^{I} species assessed by OCP analysis (vide infra).

- *Formal potential*: The reduction potential of a redox couple measured under a specific set of conditions when the oxidized and reduced redox species are present at concentrations such that the ratio of their concentrations in defined stoichiometry is unity and other components of the medium are present at designated concentrations. The formal potential is denoted by $E^{0\prime}$ and incorporates the standard potential (obtained under standard conditions with unit activity) and activity coefficients.[14] We use this term herein in our

discussion of the temperature coefficient (α), which is defined as the change in the formal potential of a redox couple with respect to temperature (see eq 1).[15–18]

- *Apparent peak potential*: Anodic and cathodic peak potentials represent the measured electrode potentials at the maximum anodic and cathodic currents, respectively. The difference between the anodic and cathodic peak potentials is called peak-to-peak separation and denoted with ΔE_p.[19] We include the term "apparent" as our copper system of interest involves both electron-transfer and chemical (i.e., ligand dissociation–association) processes, displays quasi-reversible electrochemical behavior, and is operated under non-standard conditions.[20,21] In our study, we use the apparent anodic and cathodic peak potentials to provide apparent half-wave potentials (vide infra) and peak-to-peak separations (see Table 1).

- *Apparent half-wave potential*: For an electrochemically reversible redox couple, the half-wave potential is the midpoint between the anodic and cathodic peak potentials.[19] Using the apparent anodic and cathodic peak potential values (vide supra), we obtain apparent half-wave potential values, denoted with $E_{1/2}$. These values are employed to estimate the temperature coefficients of Cu^{II}/Cu^{I} redox processes under different experimental conditions (vide infra).

In the absence and presence of 15 equiv of ^{t}Bu-py, the observed quasi-reversible Cu^{II}/Cu^{I} redox wave may be assigned to the $[Cu(Me_2\text{-phen})_2(MeCN)]^{2+}/[Cu(Me_2\text{-phen})_2]^{+}$ and $[Cu(Me_2\text{-phen})_2(^t\text{Bu-py})]^{2+}/[Cu(Me_2\text{-phen})_2]^{+}$ redox couples, respectively. These redox processes are postulated to represent an electron-transfer (i.e., reduction of Cu^{II} to Cu^{I}) event followed by a homogeneous chemical reaction (i.e., ligand dissociation–association), also known as an EC-type mechanism.[22] In the presence of low concentrations of ^{t}Bu-py, both redox events are likely operative. This is supported by the concentration-dependent $E_{1/2}$ value of **1a•^{t}Bu-py**, for which $E_{1/2}$ = 0.365 V vs Ag/$AgNO_3$ at 3.0 mM in MeCN solution containing 0.1 M (^{n}Bu_4N)(PF_6) supporting but shifts to 0.374 V vs Ag/$AgNO_3$ (i.e., closer to the $E_{1/2}$ value observed for **1a**) at 1.0 mM concentration of the complex. The increase in ΔE_p and decrease in reversibility of the cathodic wave upon incremental addition of ^{t}Bu-py to an MeCN solution of **1a** (see Table 1, Figure 3, and vide infra) is attributed to slower rate of reduction of $[Cu(Me_2\text{-phen})_2(^t\text{Bu-py})]^{2+}$ than of $[Cu(Me_2\text{-phen})_2(MeCN)]^{2+}$, likely arising from greater binding strength and steric bulk of the ^{t}Bu-py ligand compared to MeCN.

Diffusion Coefficient Determination

The diffusion coefficients associated with the Cu^{II}/Cu^{I} redox process for complexes **1a**, **1a•*ᵗ*Bu-py**, **1b**, and **2** (3.0 mM) in the absence and presence of variable amount of ^{t}Bu-py (equiv of ^{t}Bu-py is based on the total copper concentration in the sample solution) were determined at the ambient glovebox temperature (~23–25 °C) from variable-scan-rate (25–400 mV s^{-1}) CV measurements. The anodic and cathodic diffusion coefficients of the Cu^{II}/Cu^{I} redox couple were separately quantified from the anodic and cathodic peak currents, respectively, using Randles–Ševčík analysis.[23,24] Specifically, the diffusion coefficients were estimated using the slopes of the linear fits to the data in plots of peak current vs the square root of scan rate. As the redox couples of **1a**, **1a•*ᵗ*Bu-py**, **1b**, and **2** in the absence and presence of variable amount of ^{t}Bu-py are not strictly reversible, rather quasi-reversible, as the peak potential separation is >59.2 mV per electron transfer at 25 °C (see Table 1) and increases slightly with scan rate in the 25–400 mV s^{-1} range, the diffusion coefficients were separately estimated using the Randles–Ševčík equations for a fully reversible redox couple (eq S7) and an irreversible redox couple (eq S8) at 25 °C:[14,23–25]

$$i_p = 2.69 \times 10^5 n^{3/2} A c D_0^{1/2} v^{1/2} \tag{S7}$$

$$i_p = 2.99 \times 10^5 n^{3/2} \alpha_{CT}^{1/2} A c D_0^{1/2} v^{1/2} \tag{S8}$$

In these equations, i_p is the peak current (anodic or cathodic), n is the number of electrons transferred in the given reaction, A is the geometric surface area of the working electrode (cm^2), c is the concentration of the redox-active species in the bulk solution (mol cm^{-3}), D_0 is the diffusion coefficient (cm^2 s^{-1}), v is the scan rate (V s^{-1}), and α_{CT} is the charge-transfer coefficient. In this study, α_{CT} was assumed to be 0.5 owing to the electrochemical symmetry of the investigated redox couples.[14] Summaries of the estimated anodic, cathodic, and average diffusion coefficients obtained using eqs S7 and S8 are provided in Tables S5 and S6, respectively. The true values of the diffusion coefficients are expected to be within the ranges suggested by eqs S7 and S8.

Temperature Coefficient Determination

The temperature coefficient (α) of the Cu^{II}/Cu^{I} redox couple of **1a**, **1a•*ᵗ*Bu-py**, **1b**, and **2** in the absence and presence of variable amount of ^{t}Bu-py (equiv of ^{t}Bu-py is based on the total copper concentration in the sample solution) was estimated using variable-temperature CV measurements in an isothermal electrochemical setup.[15,26] The temperature of the solution near the working electrode was controlled using a hot plate and quantified using a thermocouple

(stainless steel) immersed in the analyte solution. The thermocouple was calibrated using an external temperature controller (Omega Engineering CS8DPT). The solution temperature was increased in ~3–5 °C intervals in the temperature range ~23–44 °C. At each temperature, the cell was allowed to equilibrate for ~3 min before three CV cycles were collected. Solutions were stirred between measurements at different temperatures but paused during CV data collection. The apparent half-wave potential ($E_{1/2}$) for the Cu^{II}/Cu^{I} redox couple of each complex under given conditions was extracted from the variable-temperature CV data and plotted against temperature. Note that CV data were also collected for sample solutions after cooling back down to near room temperature for comparative purposes. We note that the temperature dependence of $E_{1/2}$ arises from a collection of processes that influence the geometric structure, electronic properties, and/or solvation of the oxidized and/or reduced copper species in solution.[15,17,27–29] As the room-temperature diffusion coefficients for the oxidized and reduced species of individual redox couples and across different samples are of the same order of magnitude in the absence and presence of ^{t}Bu-py (Tables S5 and S6), we conclude that the temperature dependence of $E_{1/2}$ for the investigated copper systems are minimally affected by changes in molecular diffusion and solution viscosity. Thus, we estimate temperature coefficients of the Cu^{II}/Cu^{I} redox couples with respect to the reference electrode potential as the slopes of the linear fits to the data of $E_{1/2}$ vs temperature plots. We obtained linear fits with $R^2 > 0.98$ for all samples. Independently prepared samples of each type were measured to ensure reproducibility. Error bars denote the standard deviation of measurements of independently prepared samples. Notably, the $Ag/AgNO_3$ reference electrode potential is also sensitive to temperature.[30] Accordingly, the true temperature coefficient of a given redox couple is obtained after correcting for the temperature coefficient of the reference electrode potential using the following equations:[31]

$$\frac{\partial E_{1/2}}{\partial T} = \frac{\partial E_{1/2\text{-meas}}}{\partial T} + \frac{\partial E_{\text{ref}}}{\partial T} \tag{S9}$$

$$\alpha = \alpha_{\text{meas}} + \alpha_{\text{ref}} \tag{S10}$$

In these equations, $E_{1/2\text{-meas}}$ is the measured apparent half-wave potential, E_{ref} is the reference electrode potential, α is the true temperature coefficient, α_{meas} is the measured temperature coefficient, and α_{ref} is the temperature coefficient of the reference electrode potential.

The temperature coefficient of the non-aqueous $Ag/AgNO_3$ reference electrode potential was estimated using a non-isothermal open-circuit potential measurement in a two-electrode setup following a modified literature procedure where one $Ag/AgNO_3$ reference electrode serves as a working electrode and a second $Ag/AgNO_3$ reference electrode serves as a

reference/counter electrode.[15,26,31] A three-compartment custom-made glass cell (Adams & Chittenden Scientific) with fine frits separating the two side compartments from the middle compartment was used for the measurements. The cell was arranged such that one of the side compartments was placed in a heating block, while the other side compartment was held at the temperature of the glovebox. For each measurement, ~10 mL and ~3 mL of MeCN containing 0.1 M (^{n}Bu$_4$N)(PF$_6$) were added to the side and middle compartments, respectively. The temperature of the solution in the side compartment housing the Ag/AgNO$_3$ electrode serving as a working electrode was increased in ~3–4 °C intervals in the temperature range ~23–44 °C. Thermocouples (stainless steel) were placed in each side compartment at the same height as the electrodes. Temperature measurements and associated calibration were carried out as previously described. At each temperature, the cell was allowed to equilibrate for ~3 min before E_{OCP} was recorded for 120 s while stirring the solution. The average E_{OCP} values were plotted against the temperature difference between the two Ag/AgNO$_3$ electrodes, and the slope of the linear fit to the data afforded the temperature coefficient of the Ag/AgNO$_3$ reference electrode potential (α_{ref}). The value α_{ref} = 0.53(3) mV °C^{-1}, previously reported by our group,[26] was confirmed by a single measurement and aligns well with values reported in the literature for the Ag/AgNO$_3$ reference electrode potential in MeCN solutions.[30,32] We note that no correction for the thermal liquid junction potential was made because such contribution should be minimal in a three-compartment double-fritted electrochemical cell using the same electrolyte solution inside and outside of the reference electrode frit.

The temperature coefficients of the [Cu(Me$_2$-phen)$_2$(MeCN)]$^{2+}$/[Cu(Me$_2$-phen)$_2$]$^+$ and [Cu(Me$_2$-phen)$_2$(^{t}Bu-py)]$^{2+}$/[Cu(Me$_2$-phen)$_2$]$^+$ redox couples were also assessed using variable-temperature E_{OCP} measurements in an isothermal electrochemical setup.[15,32] An equimolar solution of **1a** and **1b** (3 mM total) in dry MeCN containing 0.1 M (^{n}Bu$_4$N)(PF$_6$) supporting electrolyte was prepared and either 0 or 15 equiv of ^{t}Bu-py was added. The E_{OCP} was then measured for 120 s under stirring at selected temperatures in the temperature range ~22–43 °C (Figures S74 and S76). Temperature measurements and associated calibration were carried out as previously described. As the sample solutions contain equimolar Cu^{II} and Cu^I redox species, the measured E_{OCP} values are a good estimate of $E_{1/2}$. The slopes of the linear fits to the data of E_{OCP} vs temperature plots afford temperature coefficients of the [Cu(Me$_2$-phen)$_2$(MeCN)]$^{2+}$/[Cu(Me$_2$-phen)$_2$]$^+$ (Figure S75) and [Cu(Me$_2$-phen)$_2$(^{t}Bu-py)]$^{2+}$/[Cu(Me$_2$-phen)$_2$]$^+$ (Figure S77) redox couples. Values of α = 1.76(5) mV °C^{-1} and α = 2.26(6) mV °C^{-1} were obtained for the data in the absence and presence of ^{t}Bu-py, respectively, after accounting for the temperature dependence of the Ag/AgNO$_3$ reference electrode potential. The reported errors

correspond to the standard deviation of measurements of independently prepared samples or the value obtained through error propagation of the standard error in the slope of the linear fit to individual data sets, whichever is larger. Note that these temperature coefficient values are statistically identical to the values obtained for **1a** and **1b** in the absence and presence of 15 equiv of *ᵗ*Bu-py using variable-temperature cyclic voltammetry analysis (Table 1, Figure S78), demonstrating good agreement between the two methods in assessing the temperature coefficients of this copper system under different conditions.

Redox Reaction Entropy Determination

The temperature coefficient values for the Cu^{II}/Cu^{I} redox couple of **1a**, **1a•^{t}Bu-py**, **1b**, and **2** in the absence and presence of variable amount of ^{t}Bu-py (equiv of ^{t}Bu-py is based on the total copper concentration in the sample solution) were used to estimate the associated redox reaction entropies (ΔS_{redox}) using the following equation:[17,31,33]

$$\Delta S_{redox} = S_{red} - S_{ox} = nF\alpha \qquad \text{(S11)}$$

In this equation, S_{red} and S_{ox} are the partial molar entropies of the reduced and oxidized species, n is the number of electrons transferred in the given reaction, F is Faraday's constant, and α is the corrected temperature coefficient.

Other Physical Measurements

^{1}H NMR spectra were collected at 19–22 °C at 400 MHz frequency on an automated JEOL 400 MHz (9.4 T) spectrometer. Diffusion ordered spectroscopy (DOSY) NMR experiments were collected at 19–22 °C on an automated JEOL 400 MHz (9.4 T) spectrometer using a linear array of 16 gradient field strengths from 0.03 T m^{-1} to 0.3 T m^{-1}, a diffusion time of 0.1 s, and a relaxation delay of 3 s. All chemical shift values (δ) are reported in ppm. ^{1}H NMR spectra are referenced to the residual proton signal from CD_3CN at 1.94 ppm. The MestReNova NMR data processing software (version 14.2.1) was used to analyze and process all recorded NMR spectra. The Bayesian method was used to generate DOSY NMR spectra. Error bars were determined from the width of the DOSY NMR signals. Elemental analyses of all complexes were performed at the CENTC Elemental Analysis Facility at the University of Rochester. Samples for analysis were weighed with a PerkinElmer Model AD 6000 Autobalance, and their compositions were determined with a PerkinElmer 2400 Series II Elemental Analyzer. Air-sensitive samples were handled in a VAC Atmospheres glovebox.

B. Supplementary Scheme

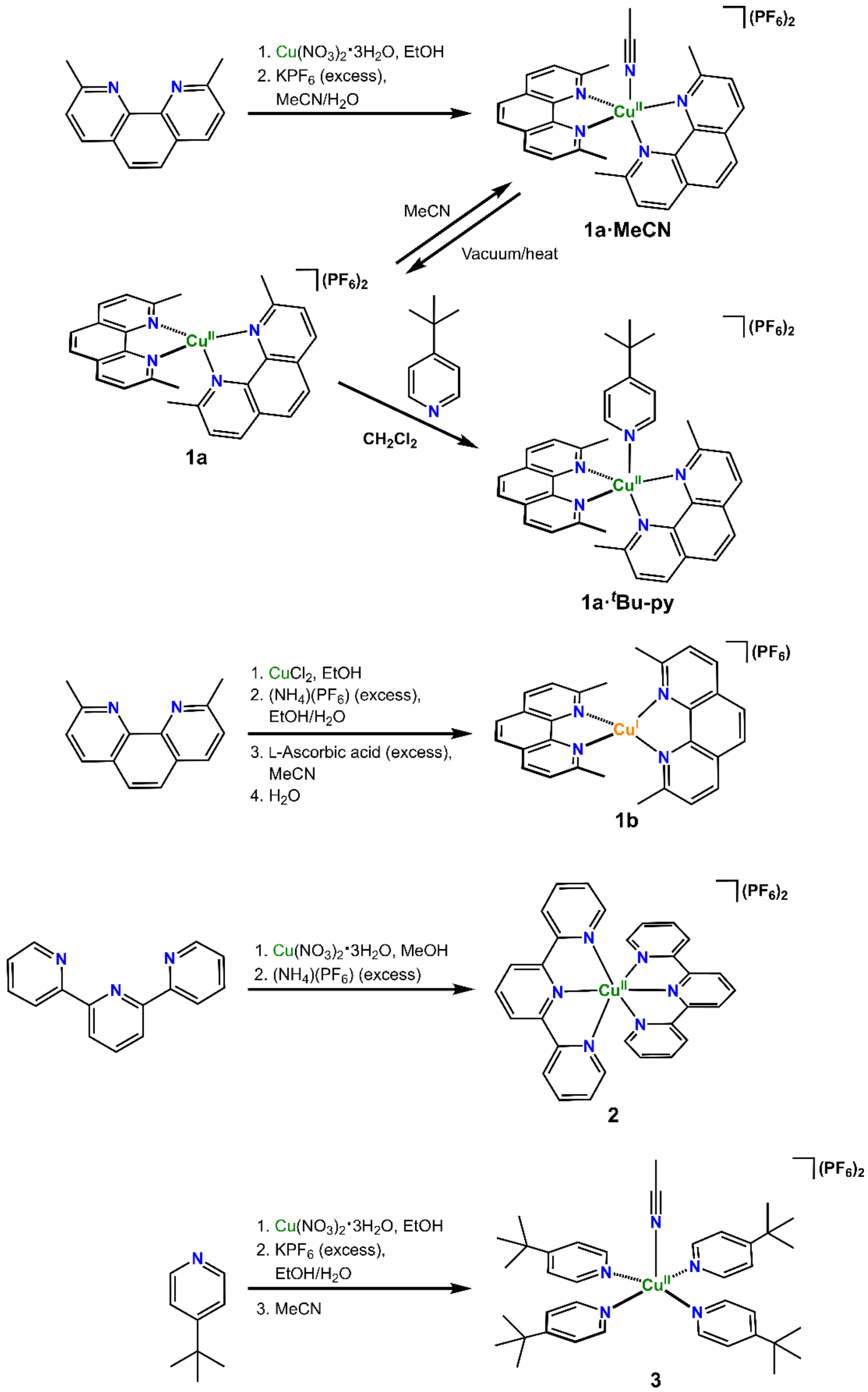

Scheme S1. Overview of the syntheses of the copper complexes studied in this work.

C. Supplementary Figures

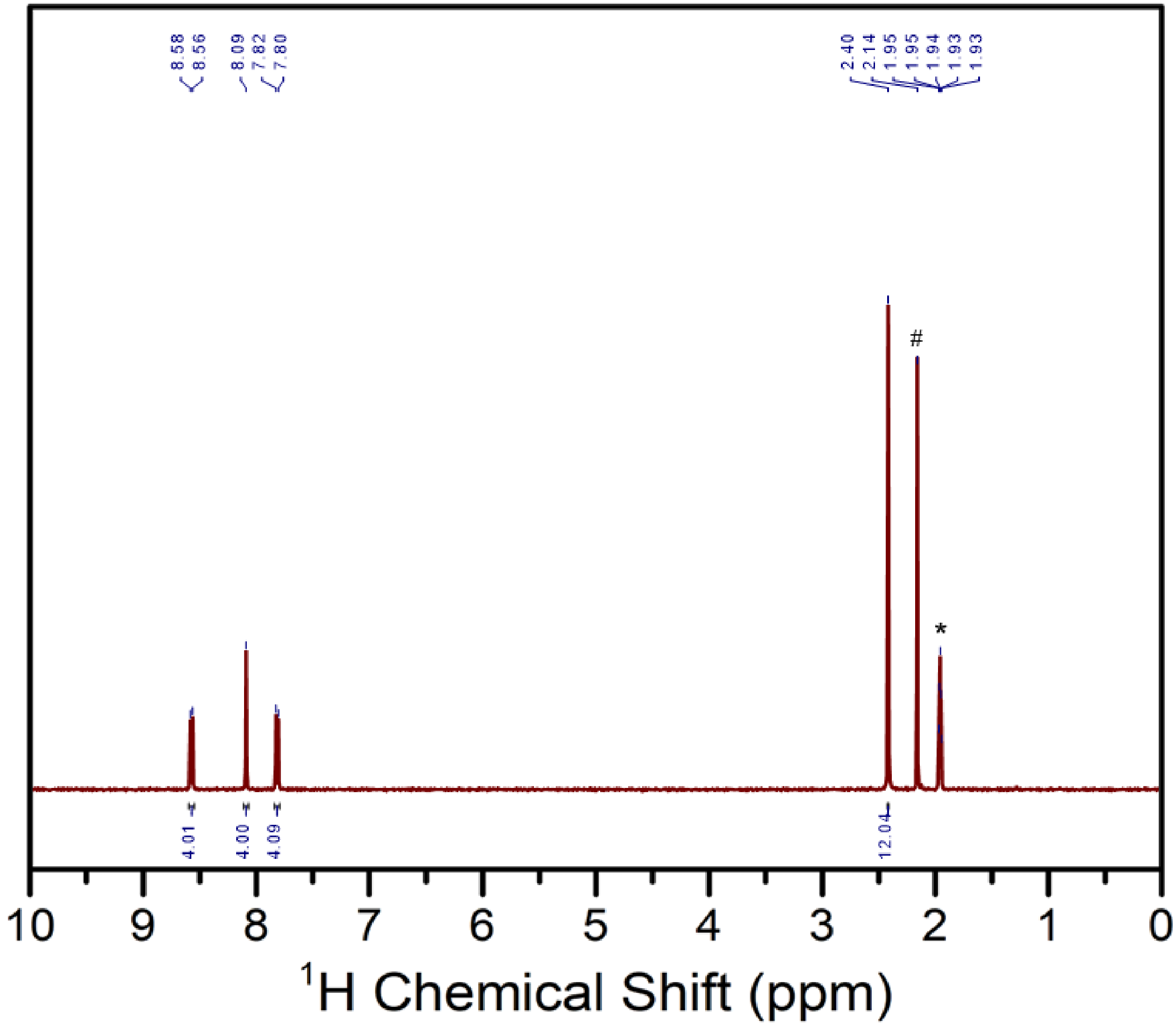

Figure S1. ^{1}H NMR spectrum of **1b** in CD_3CN. The asterisk denotes the residual proton signal from the deuterated solvent at 1.94 ppm, and the hash denotes signal from residual water at 2.14 ppm.

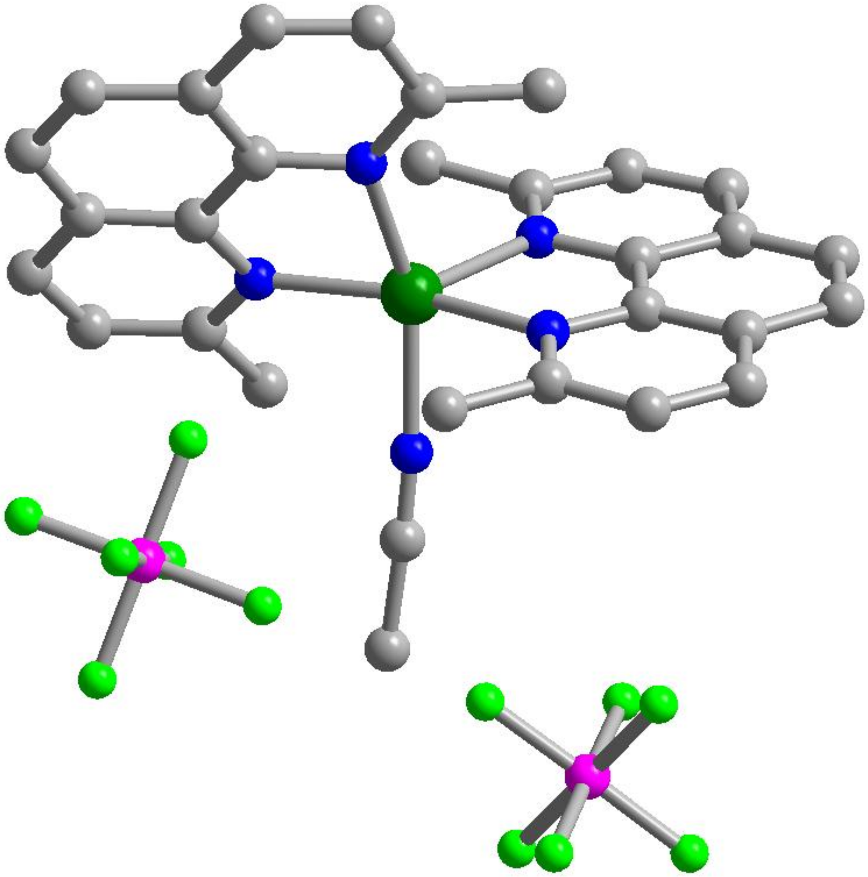

Figure S2. Crystal structure of **1a′** with MeCN solvent molecule removed for clarity. Dark green, pink, light green, blue, and gray spheres represent Cu, P, F, N, and C atoms, respectively; H atoms are omitted for clarity.

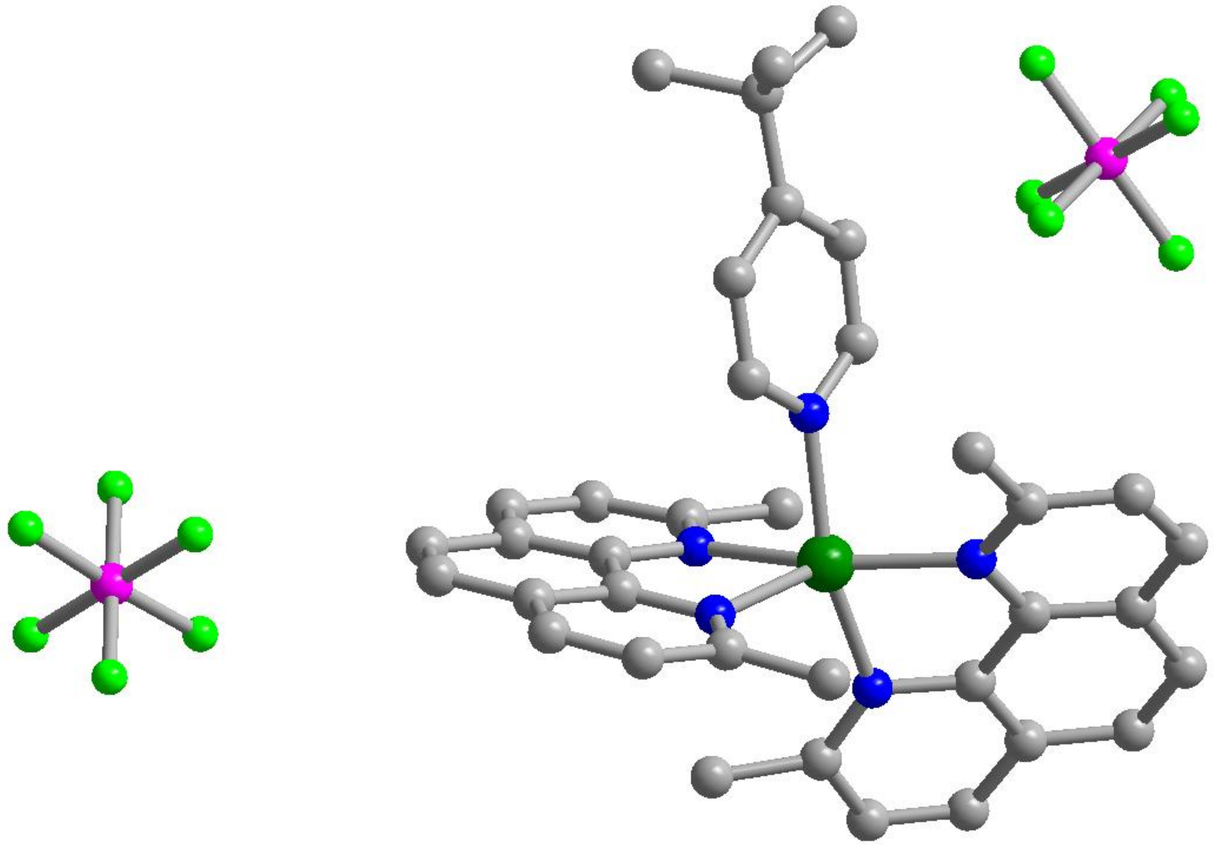

Figure S3. Crystal structure of **1a•^{t}Bu-py** with CH_2Cl_2 solvent molecule removed for clarity. Dark green, pink, light green, blue, and gray spheres represent Cu, P, F, N, and C atoms, respectively; H atoms are omitted for clarity.

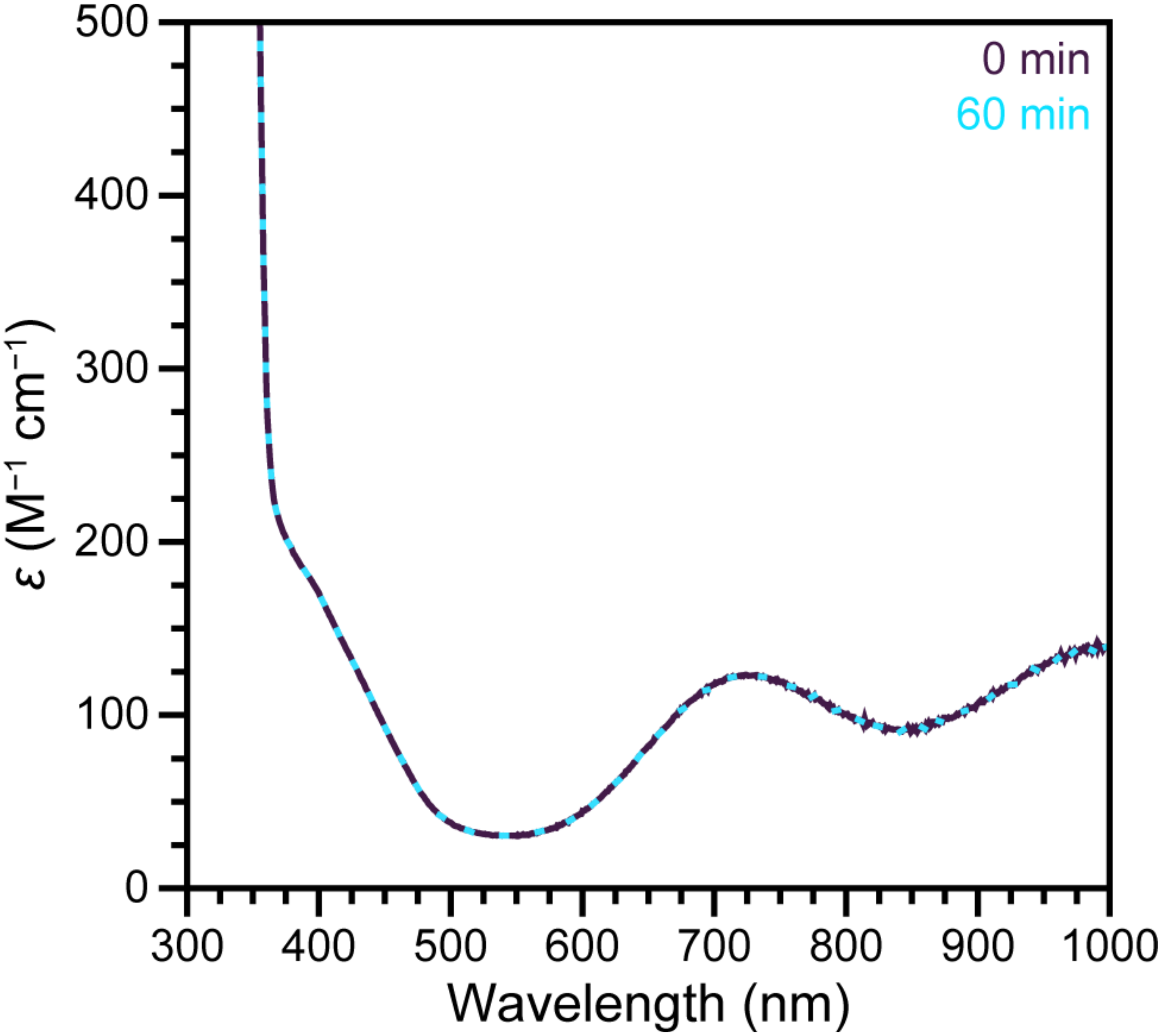

Figure S4. UV–visible–NIR absorption spectrum of 1.0 mM of **1a** in dry MeCN upon exposure to ambient air for variable time. The solid purple line corresponds to the spectrum recorded right after sample preparation. The light blue dashed line corresponds to the spectrum after 1 h of air exposure. Note that the molar absorptivity (ε) is plotted against wavelength.

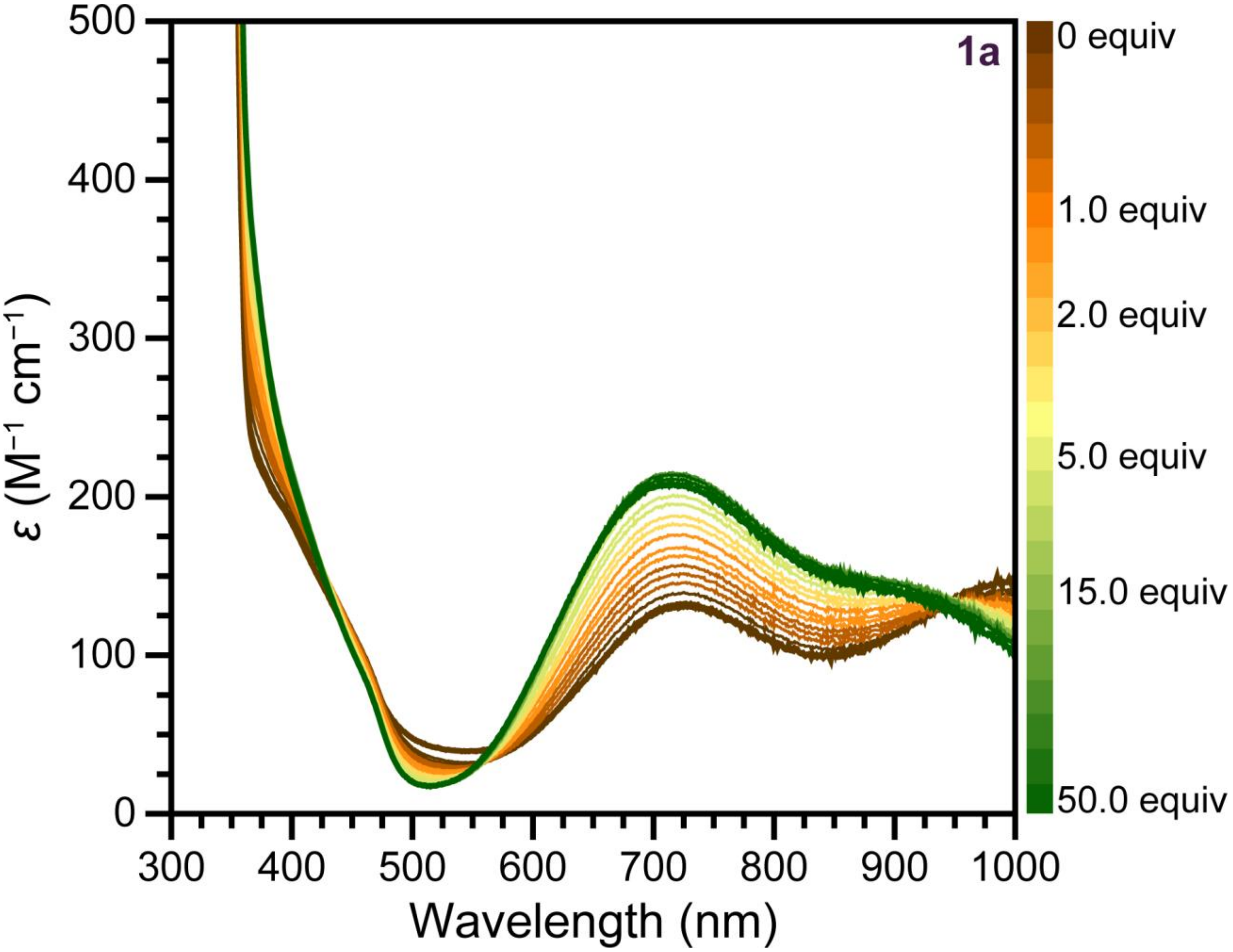

Figure S5. UV–visible–NIR absorption spectra of **1a** (initial concentration of 1.0 mM) in the absence and presence of variable amount of ^{t}Bu-py in dry MeCN. The color bar denotes the number of equiv of ^{t}Bu-py added to the sample solution. Note that the molar absorptivity (ε) is plotted against wavelength.

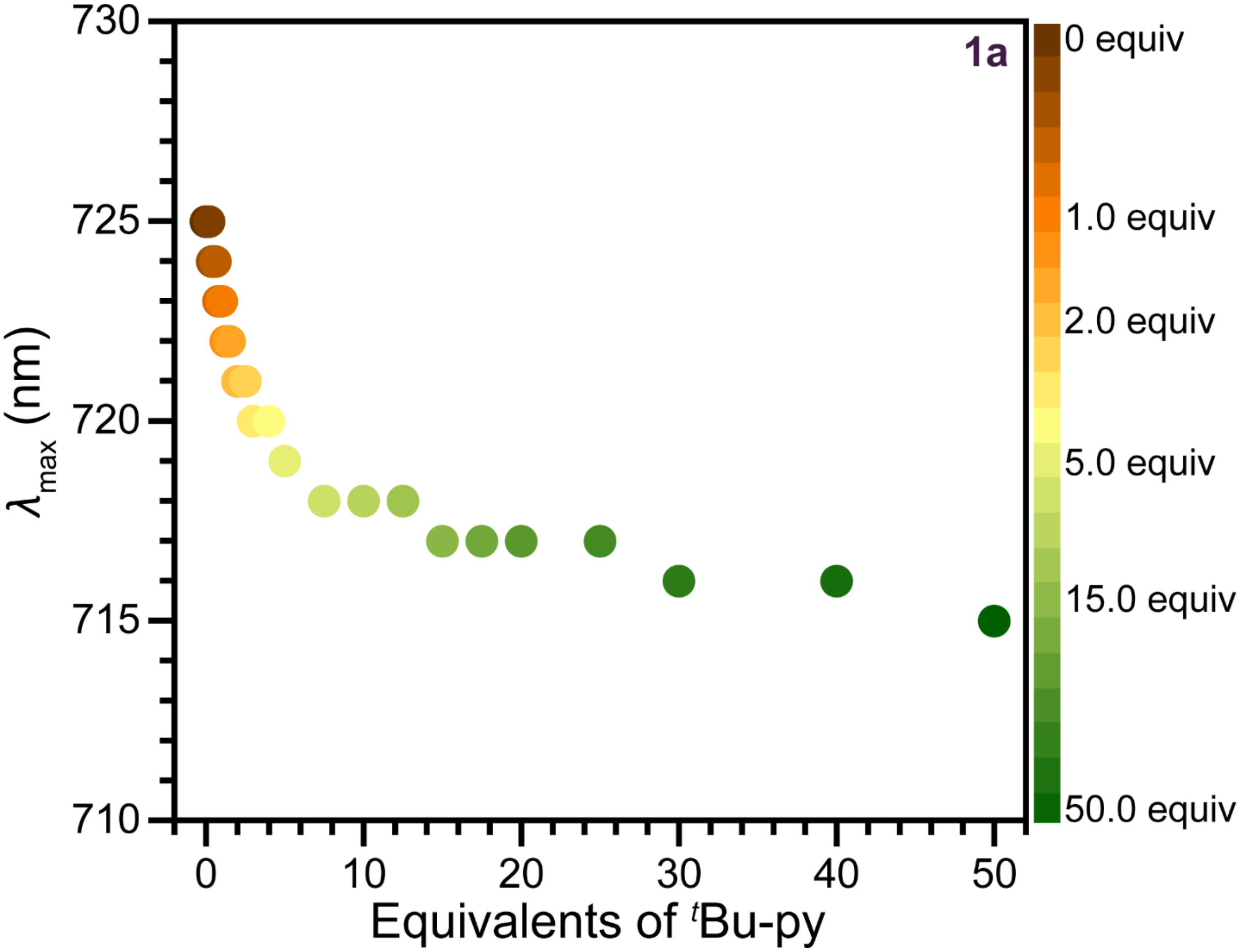

Figure S6. Plot of wavelength of maximum molar absorptivity (λ_{max}) for the d–d transition band at 715–725 nm vs equivalents of ^{t}Bu-py added for the data shown in Figure S5. The color bar denotes the number of equiv of ^{t}Bu-py added to the sample solution.

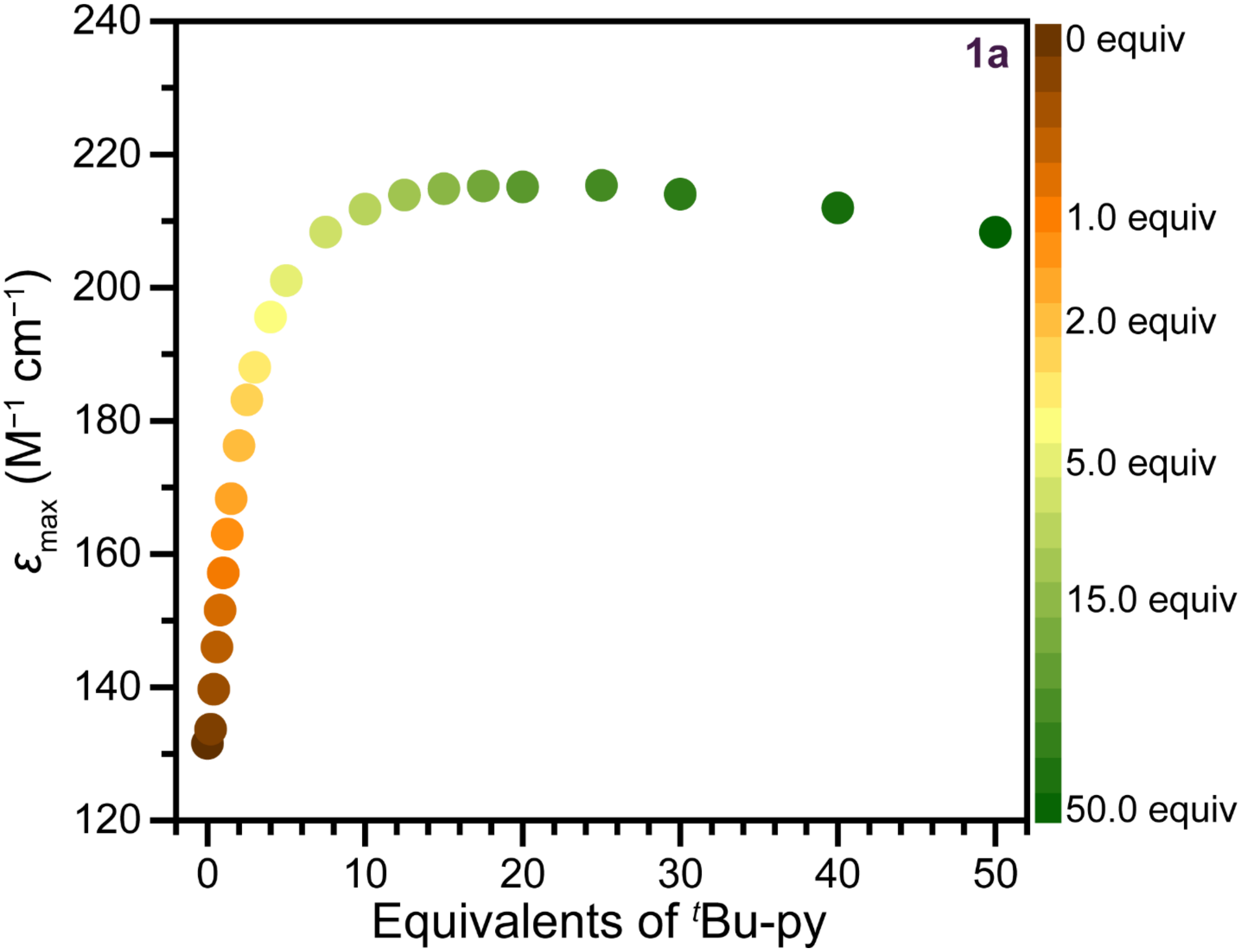

Figure S7. Plot of maximum molar absorptivity (ε_{max}) for the d–d transition band at 715–725 nm vs equivalents of ^{t}Bu-py added for the data shown in Figure S5. The color bar denotes the number of equiv of ^{t}Bu-py added to the sample solution.

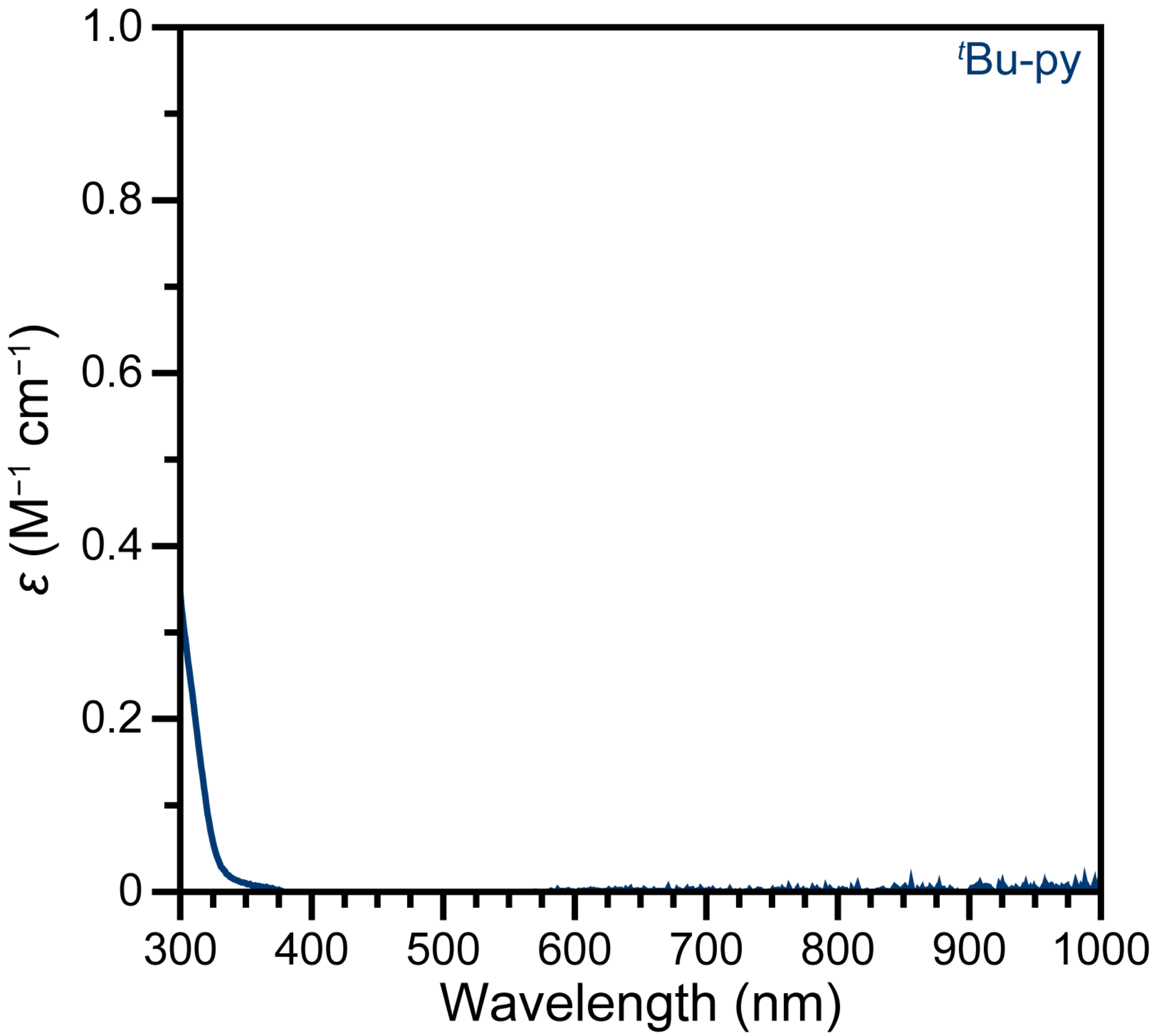

Figure S8. UV–visible–NIR absorption spectrum of 500 mM of ^{t}Bu-py in dry MeCN. Note that the molar absorptivity (ε) is plotted against wavelength.

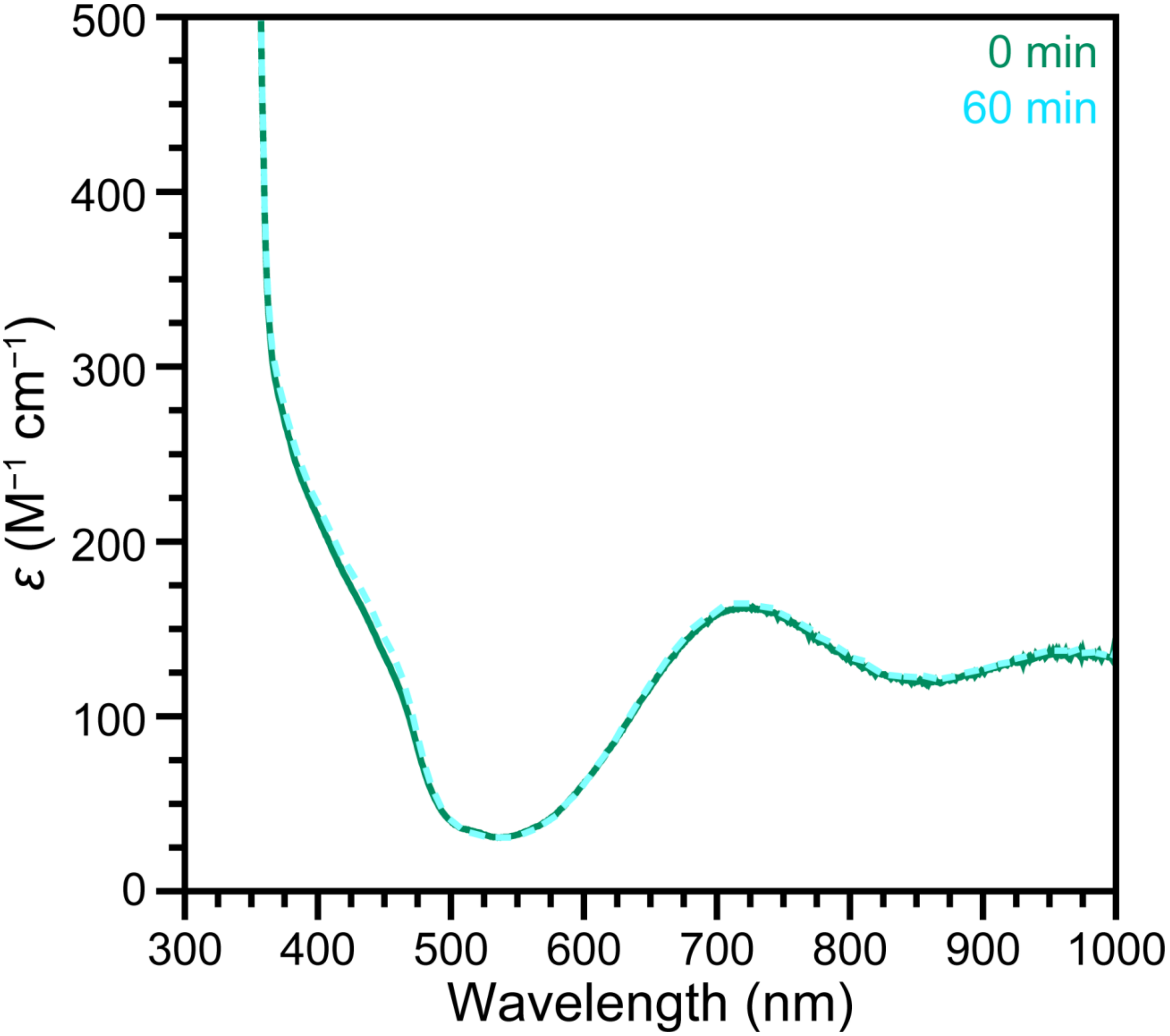

Figure S9. UV–visible–NIR absorption spectrum of 1.2 mM of **1a•^{t}Bu-py** in dry MeCN for variable time. The solid blue-green line corresponds to the spectrum recorded right after sample preparation. The light blue dashed line corresponds to the spectrum after 1 h. Note that the molar absorptivity (*ε*) is plotted against wavelength.

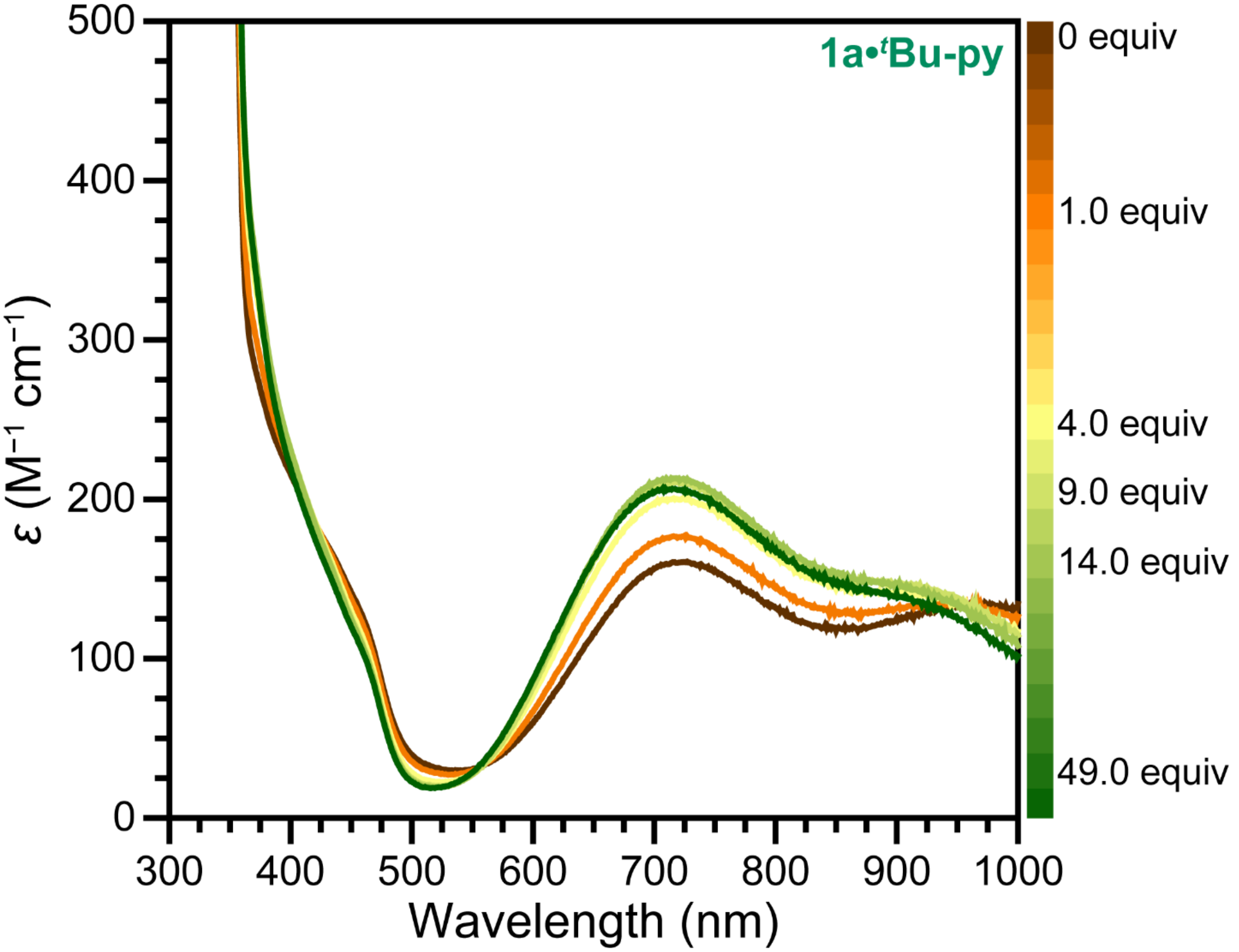

Figure S10. UV–visible–NIR absorption spectra of **1a•^{t}Bu-py** (initial concentration of 1.2 mM) in the absence and presence of variable amount of ^{t}Bu-py in dry MeCN. The color bar denotes the number of equiv of ^{t}Bu-py added to the sample solution. Note that the molar absorptivity (ε) is plotted against wavelength.

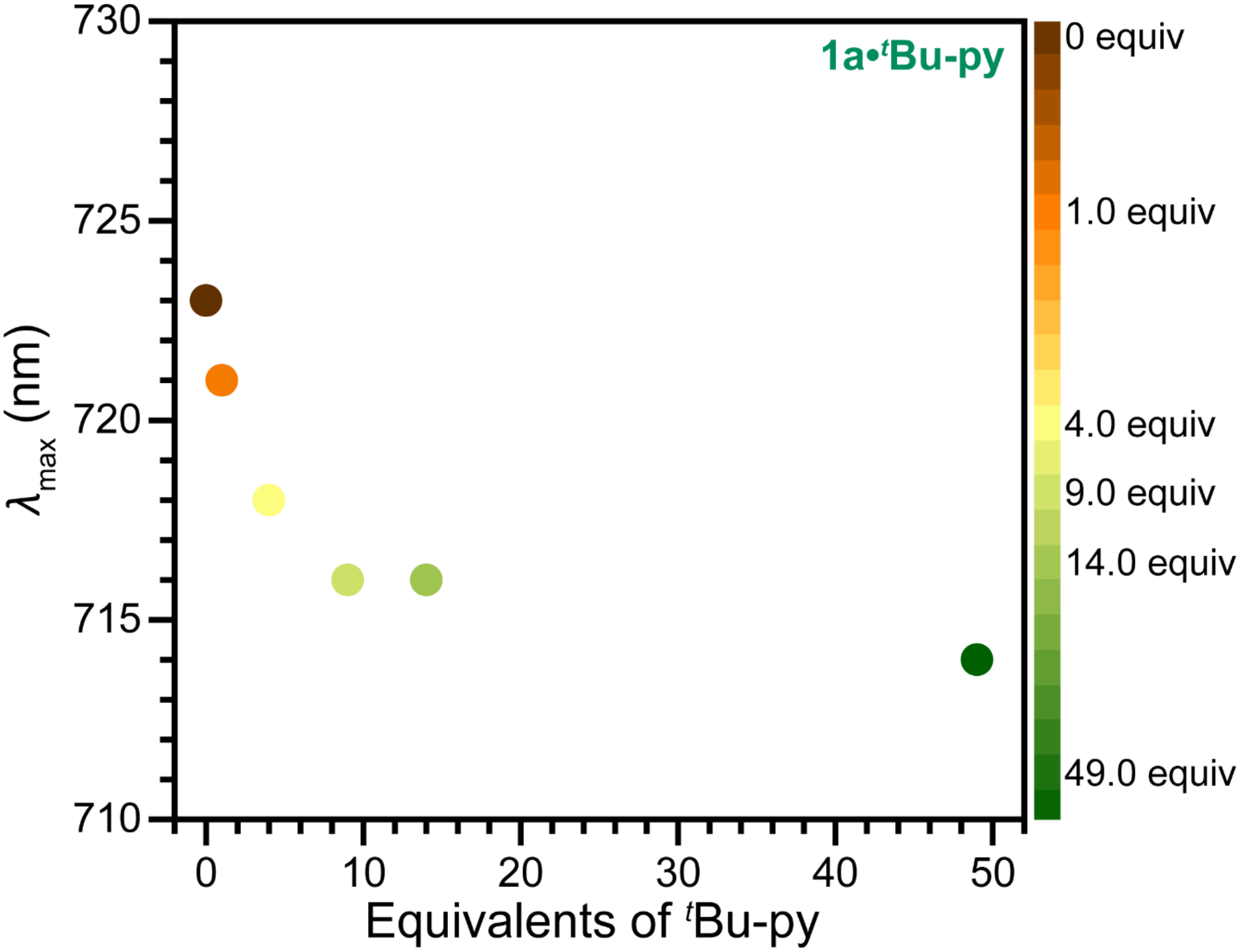

Figure S11. Plot of wavelength of maximum molar absorptivity (λ_{max}) for the d–d transition band at 714–723 nm vs equivalents of ^{t}Bu-py added for the data shown in Figure S10. The color bar denotes the number of equiv of ^{t}Bu-py added to the sample solution.

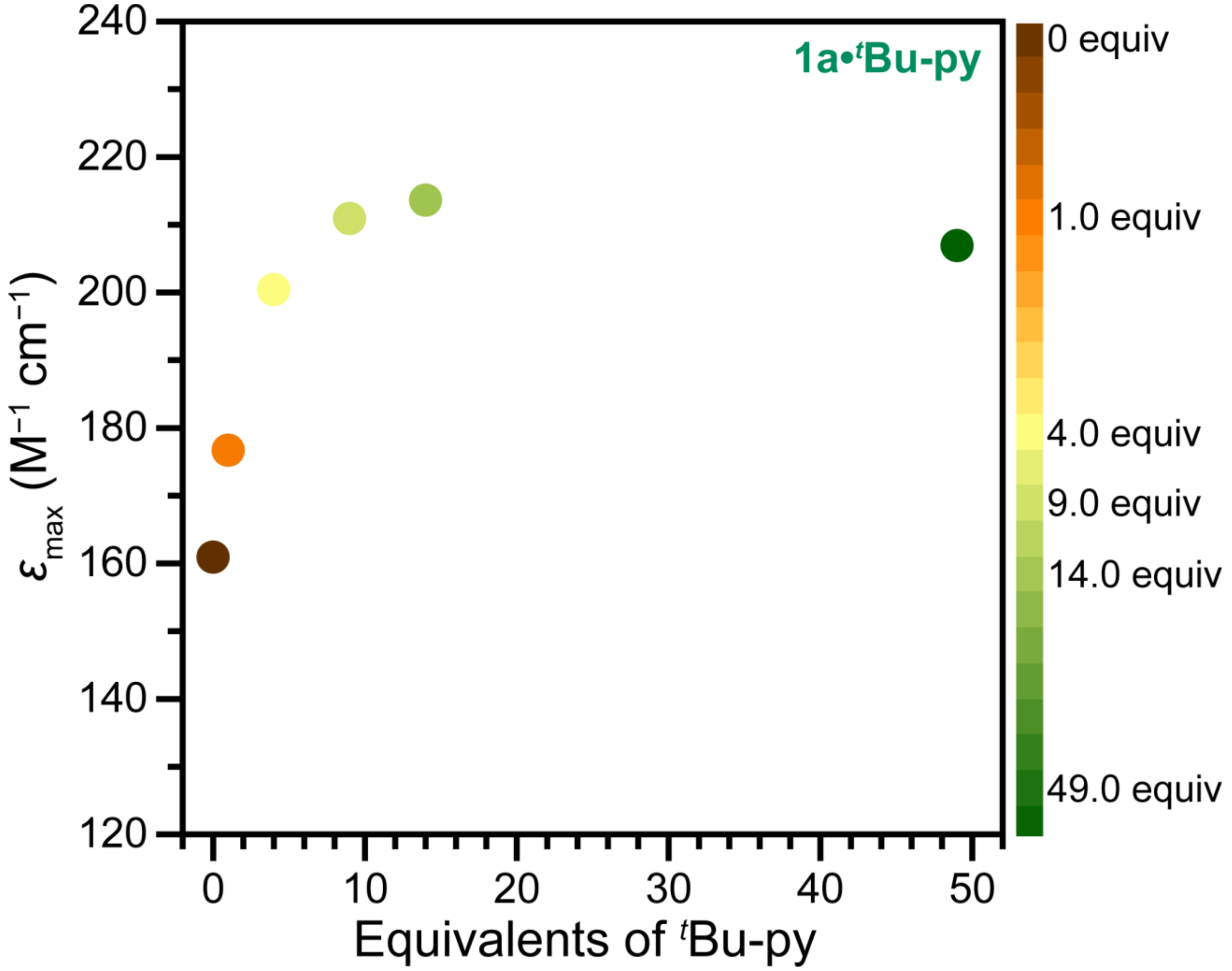

Figure S12. Plot of maximum molar absorptivity (ε_{max}) for the d–d transition band at 714–723 nm vs equivalents of ^{t}Bu-py added for the data shown in Figure S10. The color bar denotes the number of equiv of ^{t}Bu-py added to the sample solution.

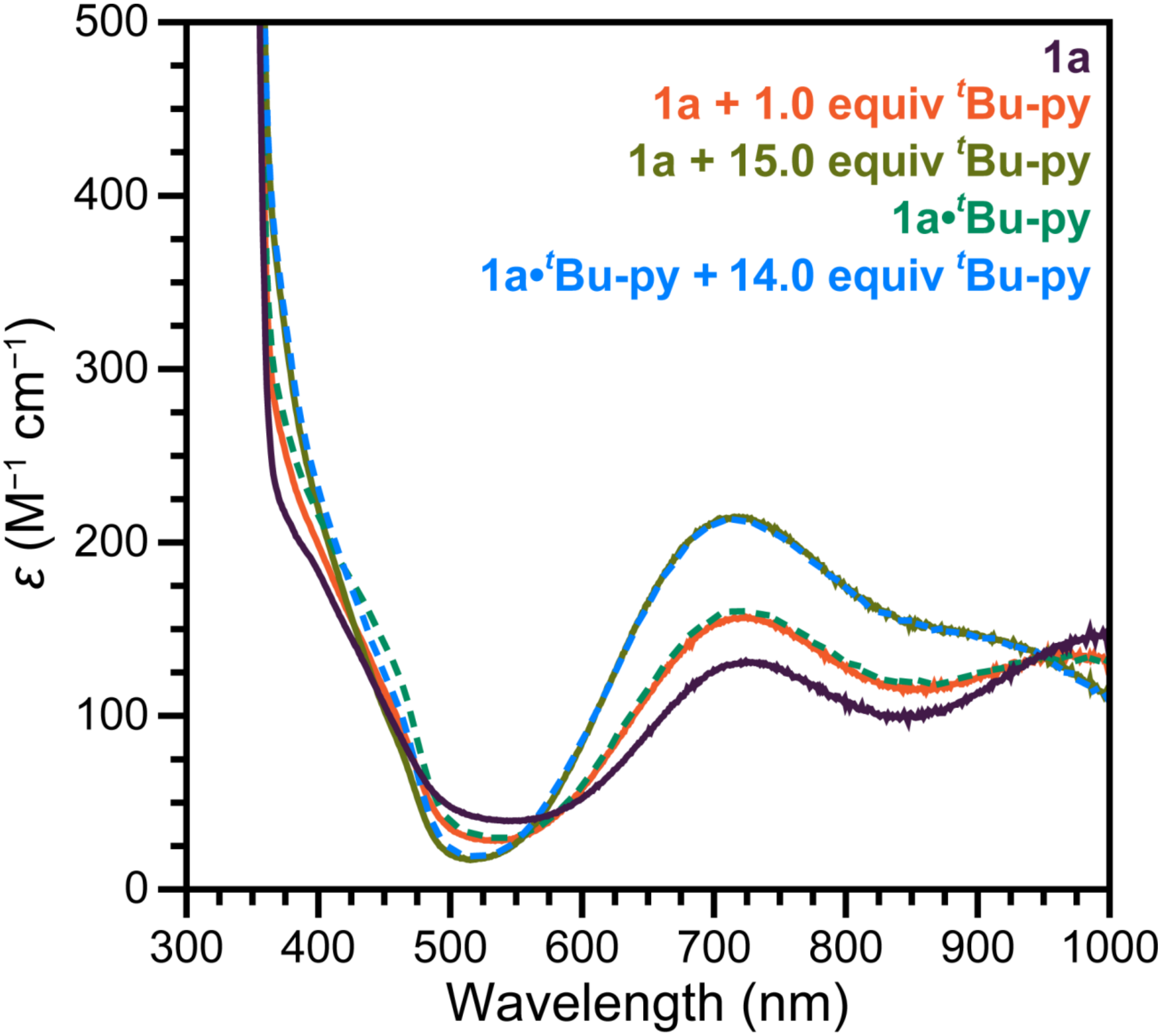

Figure S13. Comparison of the UV–visible–NIR absorption spectra of 1.0 mM of **1a** in the absence and presence of 1.0 and 15.0 equiv of ^{t}Bu-py, and 1.2 mM of **1a•^{t}Bu-py** in the absence and presence of 14.0 equiv of ^{t}Bu-py in dry MeCN. Note that the molar absorptivity (ε) is plotted against wavelength.

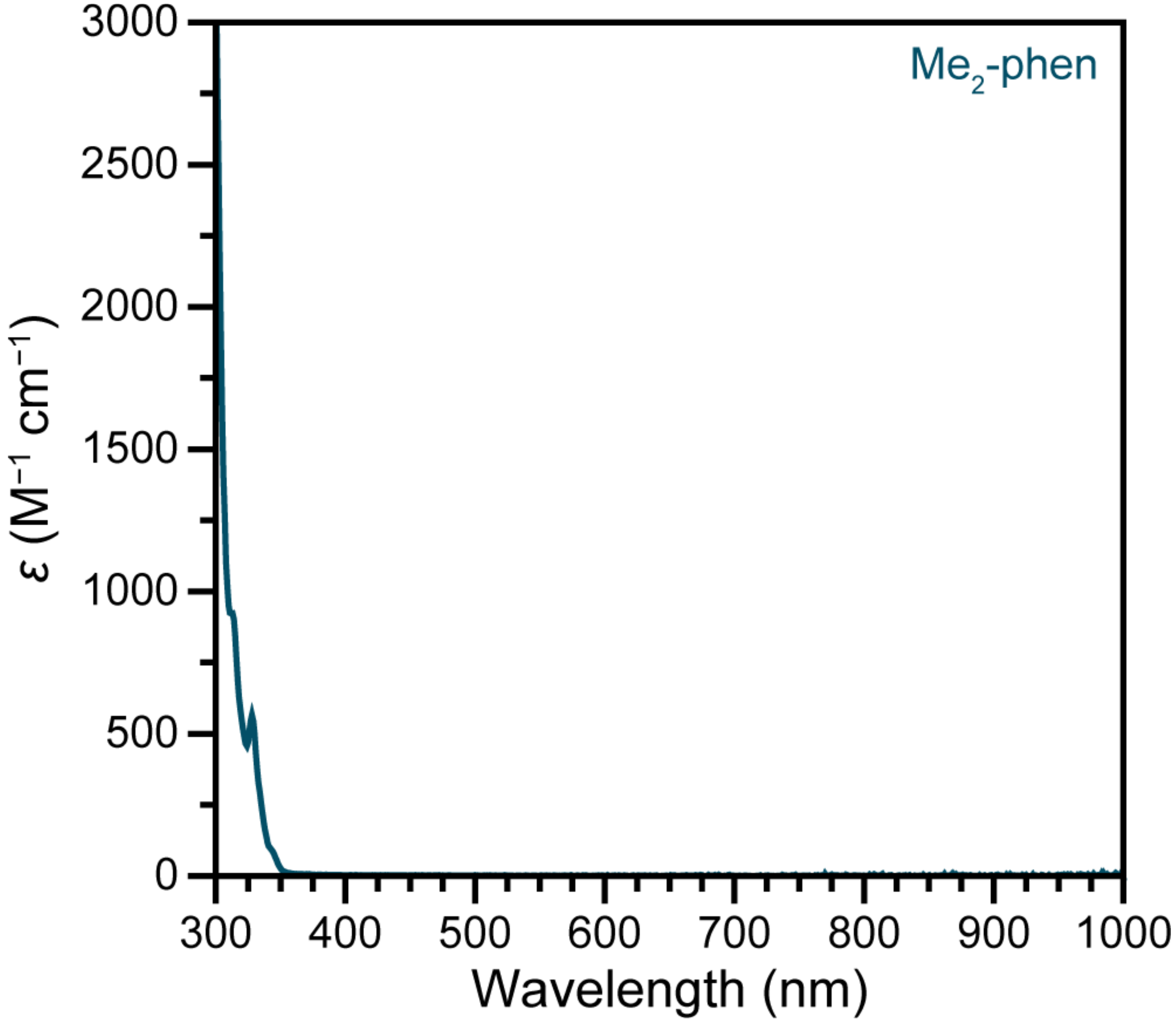

Figure S14. UV–visible–NIR absorption spectrum of 0.4 mM of Me$_2$-phen in dry MeCN. Note that the molar absorptivity (ε) is plotted against wavelength.

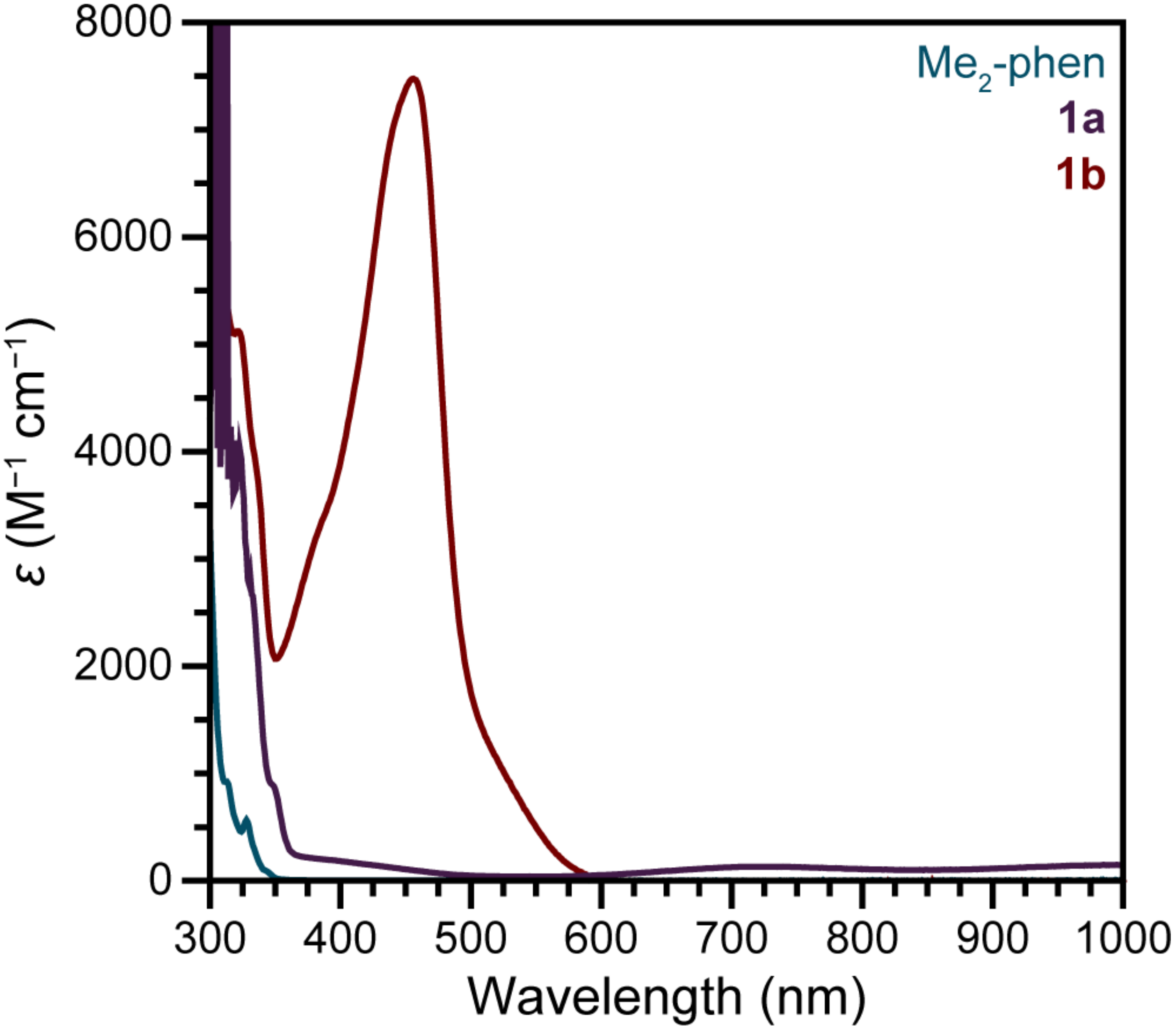

Figure S15. Comparison of the UV–visible–NIR absorption spectra of 0.4 mM of Me_2-phen, 1.0 mM of **1a**, and 0.1 mM of **1b** in dry MeCN. Note that the molar absorptivity (ε) is plotted against wavelength.

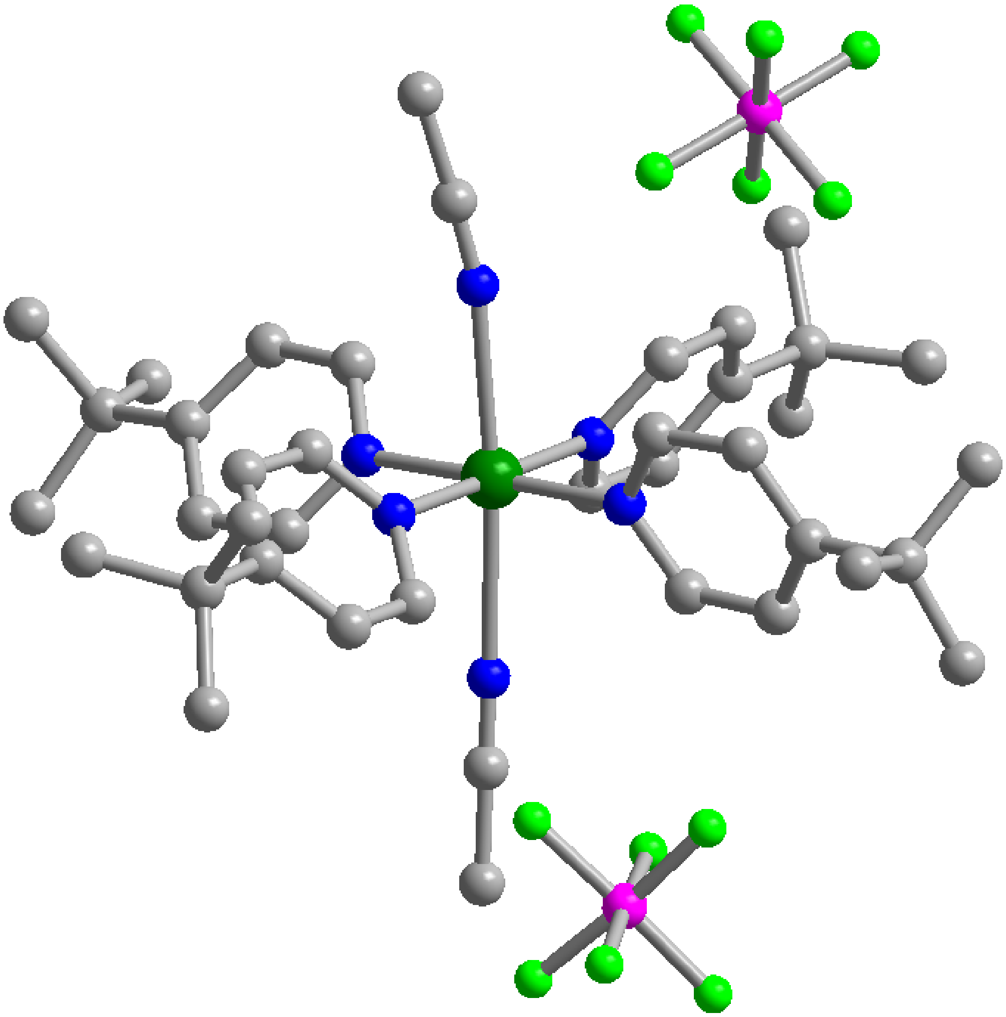

Figure S16. Crystal structure of **3′** with solvent molecules removed for clarity. Dark green, pink, light green, blue, and gray spheres represent Cu, P, F, N, and C atoms, respectively; H atoms are omitted for clarity.

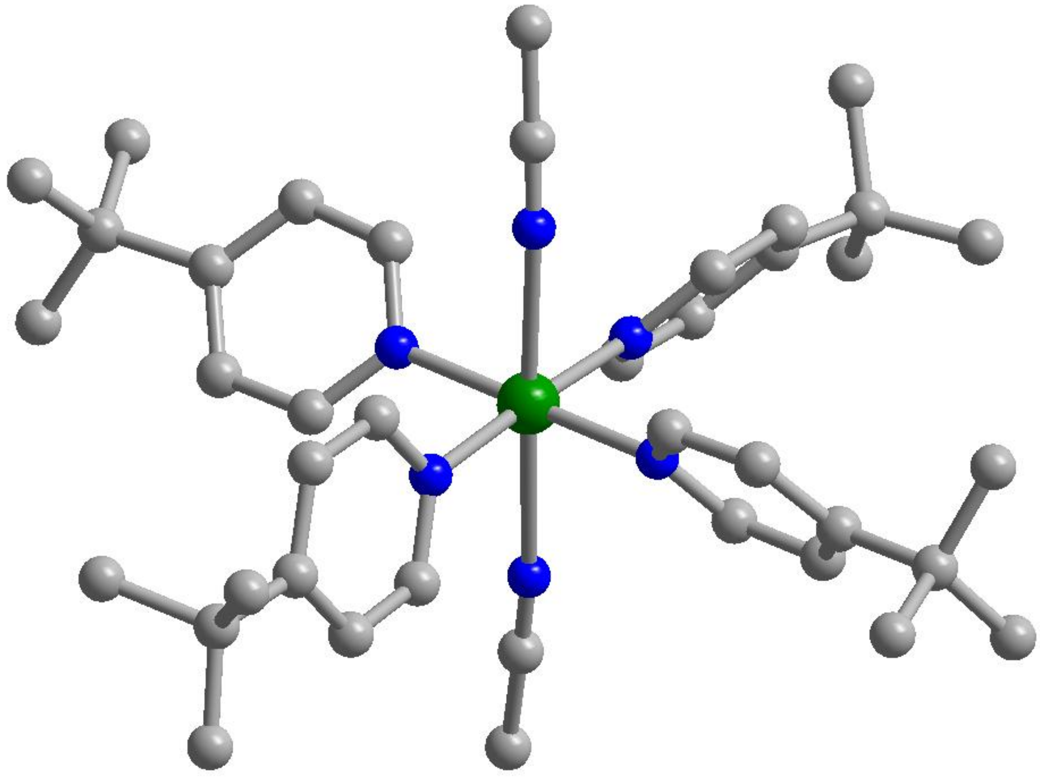

Figure S17. Crystal structure of the cationic complex [*trans*-Cu(^{t}Bu-py)$_4$(MeCN)$_2$]$^{2+}$, as observed in **3′**. Dark green, blue, and gray spheres represent Cu, N, and C atoms, respectively; H atoms are omitted for clarity.

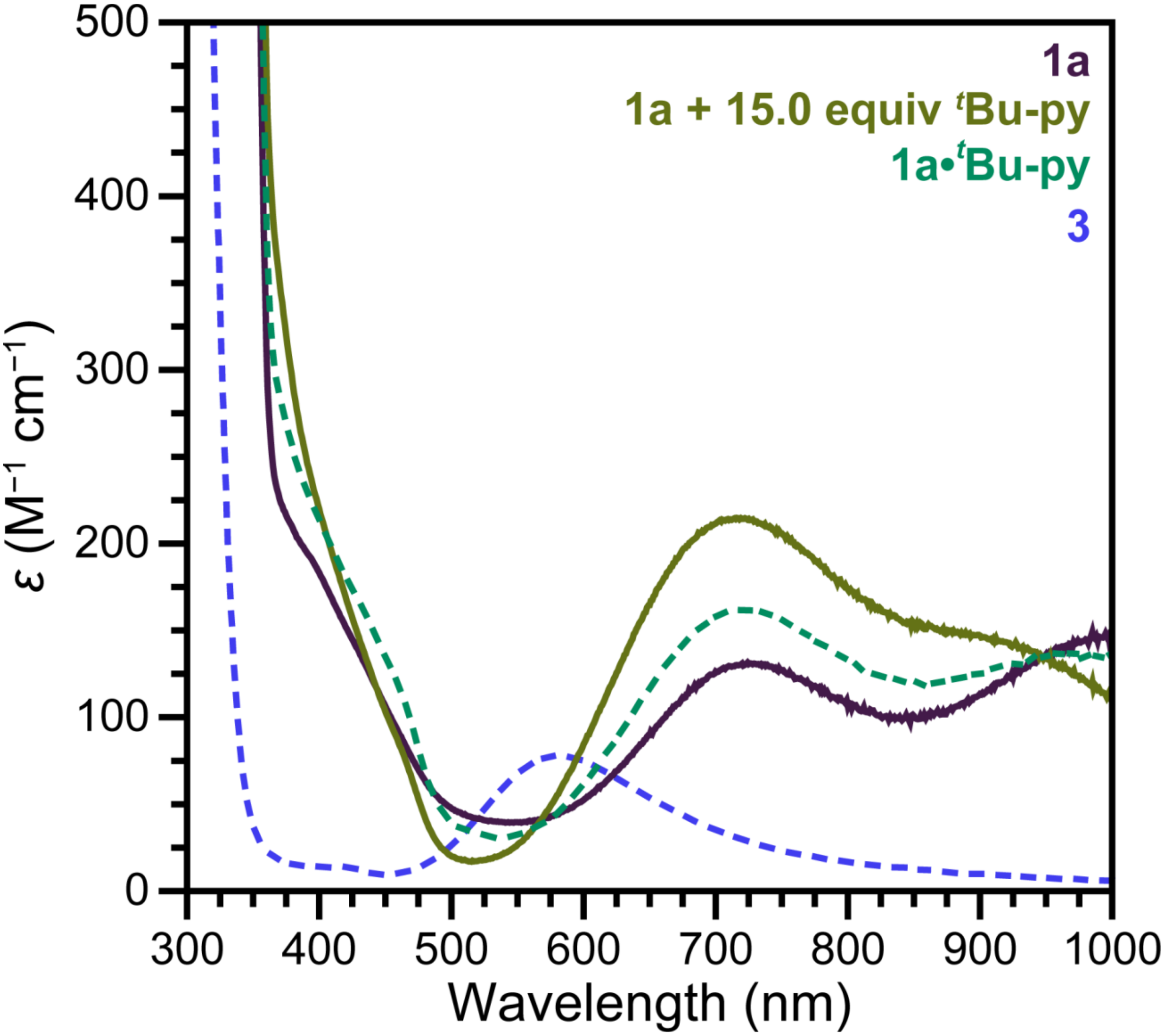

Figure S18. Comparison of the UV–visible–NIR absorption spectra of 1.0 mM of **1a** in the absence and presence of 15.0 equiv of ^{t}Bu-py, 1.2 mM of **1a•^{t}Bu-py**, and 5.0 mM of **3** in dry MeCN. Note that the molar absorptivity (ε) is plotted against wavelength.

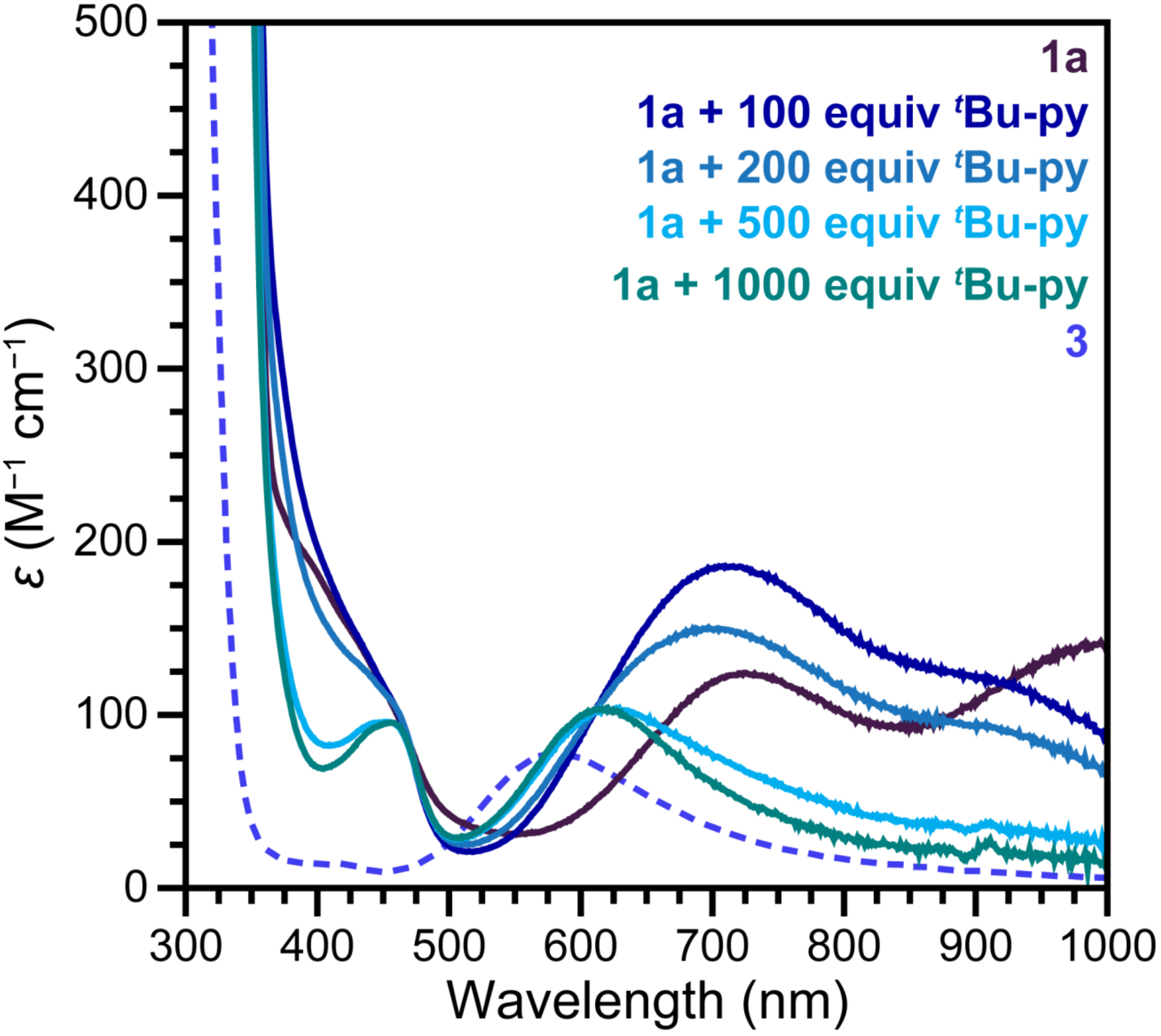

Figure S19. Comparison of the UV–visible–NIR absorption spectra of **1a** (initial concentration of 1.0 mM) in the absence and presence of 100, 200, 500, and 1000 equiv of ^{t}Bu-py, and 5.0 mM of **3** in dry MeCN. Note that the molar absorptivity (ε) is plotted against wavelength.

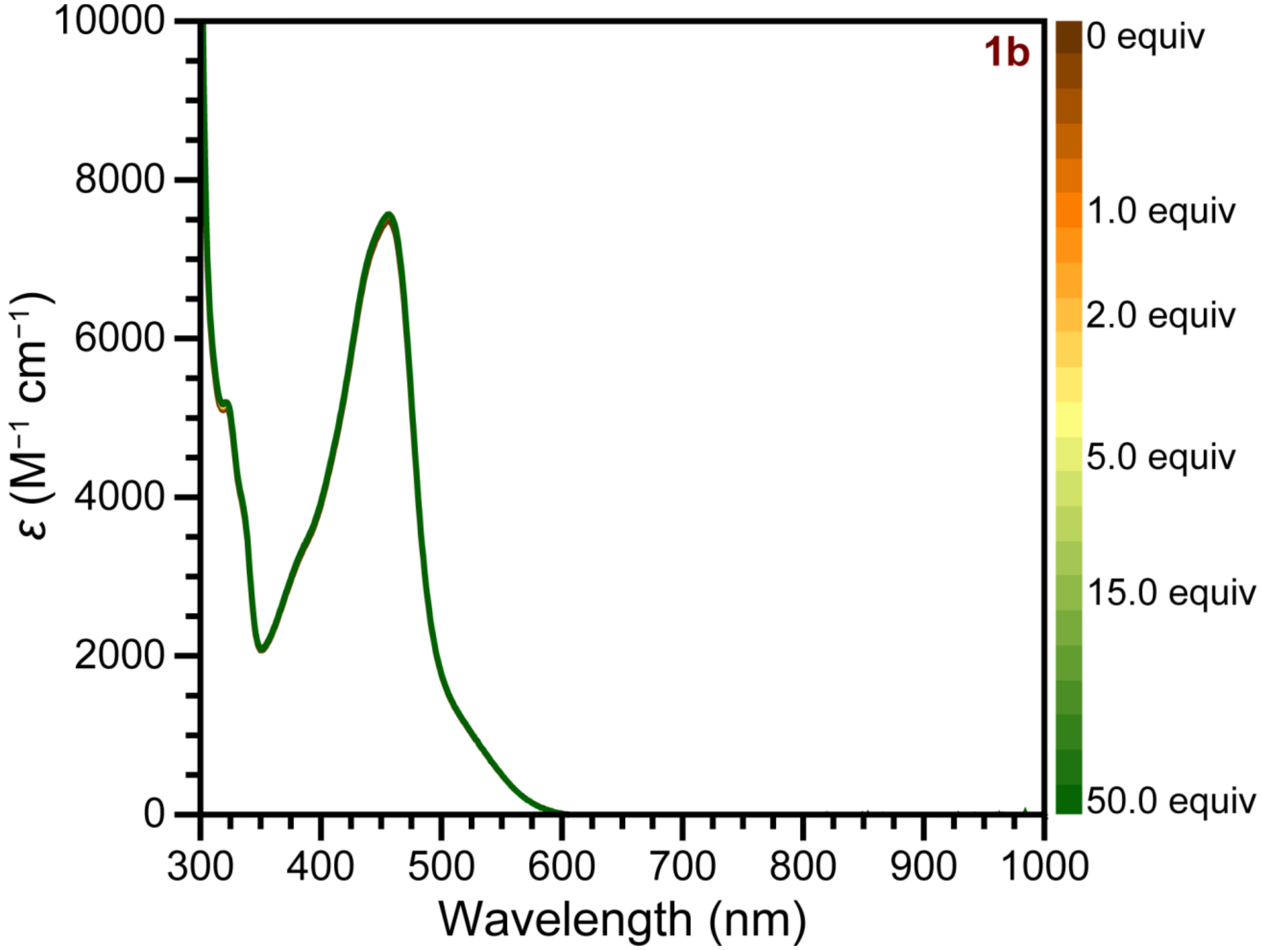

Figure S20. UV–visible–NIR absorption spectra of **1b** (initial concentration of 0.1 mM) in the absence and presence of variable amount of ^{t}Bu-py in dry MeCN. The color bar denotes the number of equiv of ^{t}Bu-py added to the sample solution. Note that the molar absorptivity (ε) is plotted against wavelength.

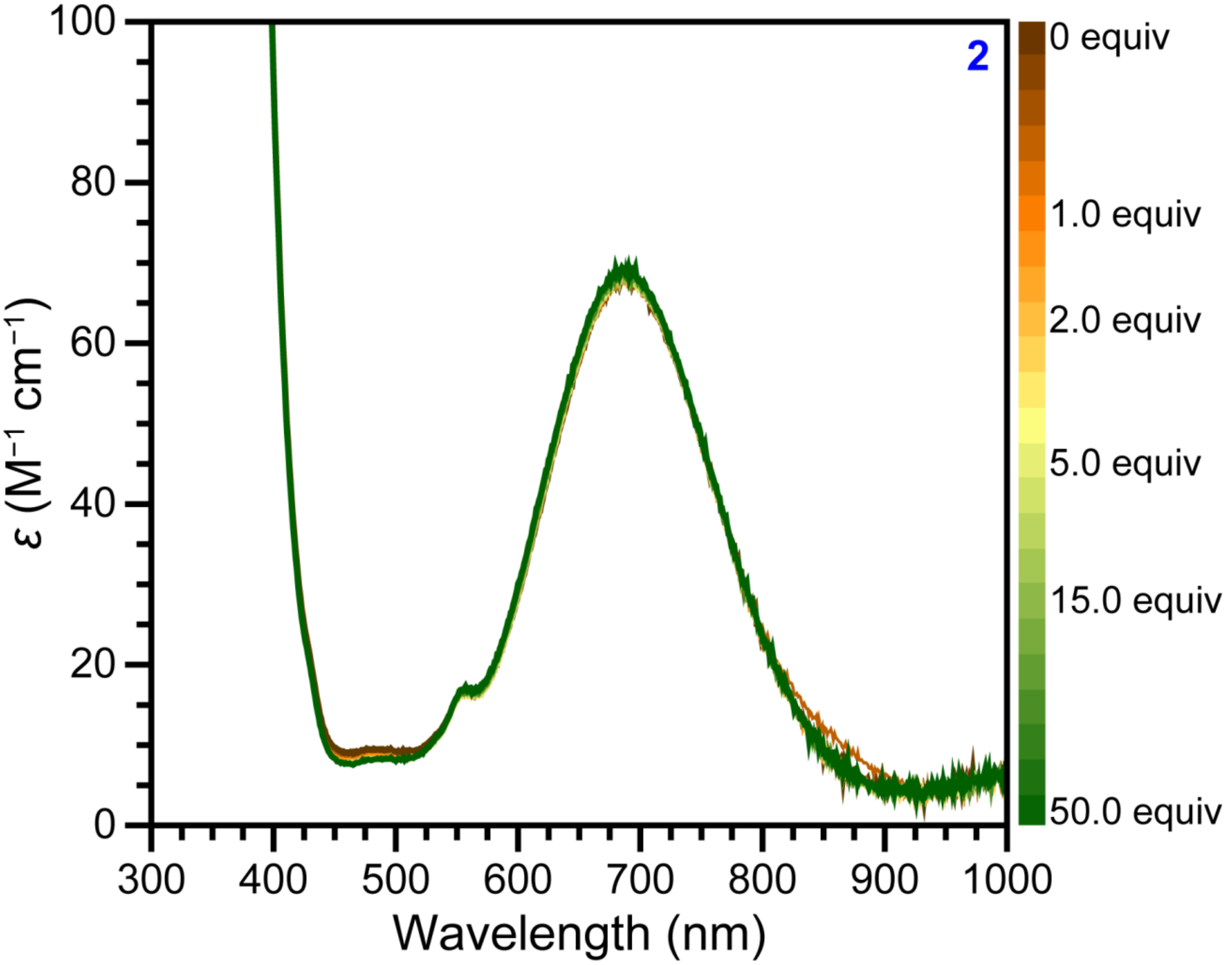

Figure S21. UV–visible–NIR absorption spectra of **2** (initial concentration of 2.0 mM) in the absence and presence of variable amount of ^{t}Bu-py in dry MeCN. The color bar denotes the number of equiv of ^{t}Bu-py added to the sample solution. Note that the molar absorptivity (*ε*) is plotted against wavelength.

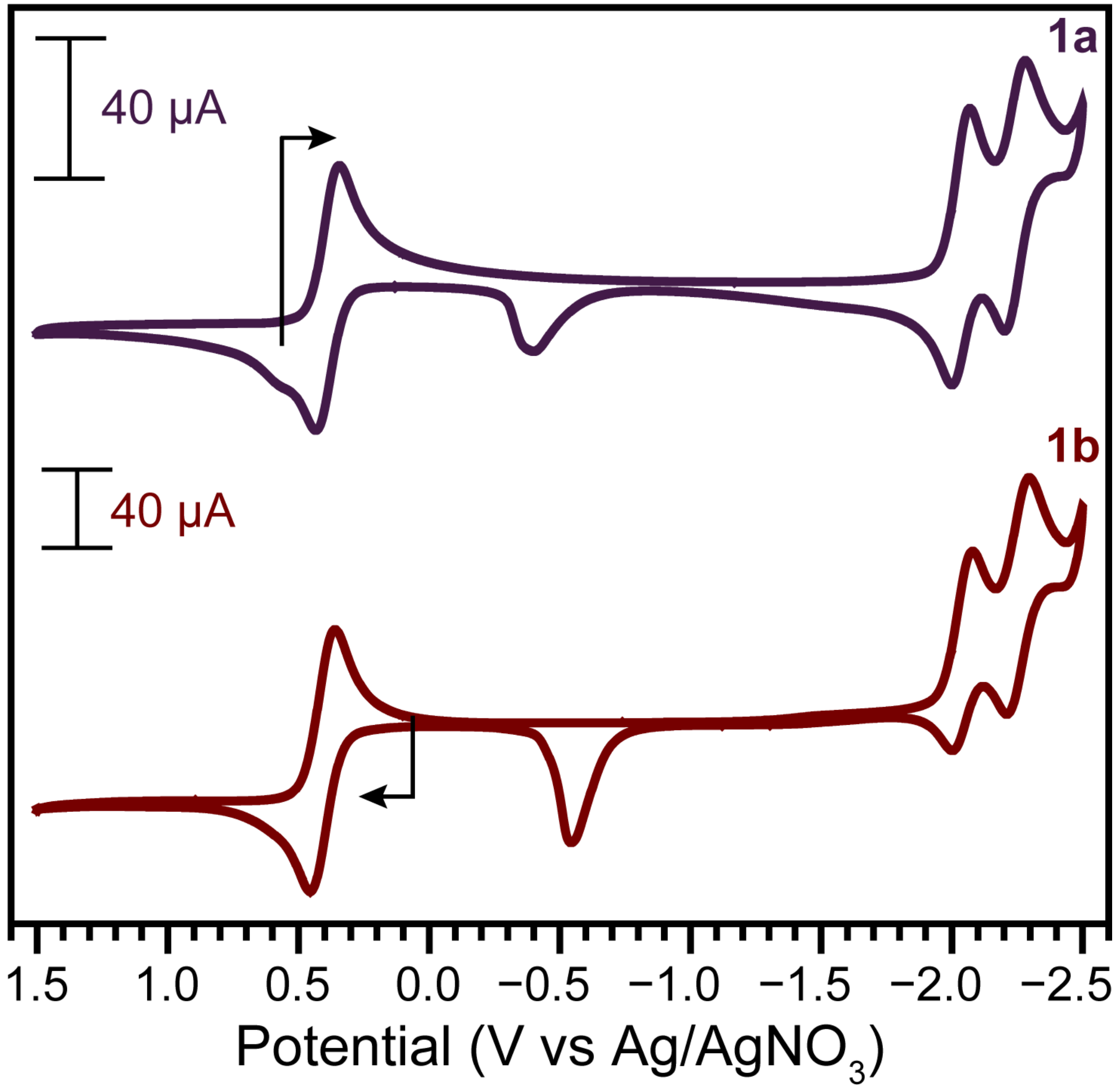

Figure S22. Cyclic voltammograms of 3.0 mM of **1a** and 3.0 mM of **1b** in dry MeCN containing 0.1 M ($^{n}Bu_4N$)(PF_6) supporting electrolyte collected at room temperature (23–25 °C) using 100 mV s^{-1} scan rate. Vertical black lines and arrows denote the open-circuit potentials and scan directions, respectively. Glassy carbon, Ag/$AgNO_3$, and Pt mesh were used as working, reference, and counter electrodes, respectively.

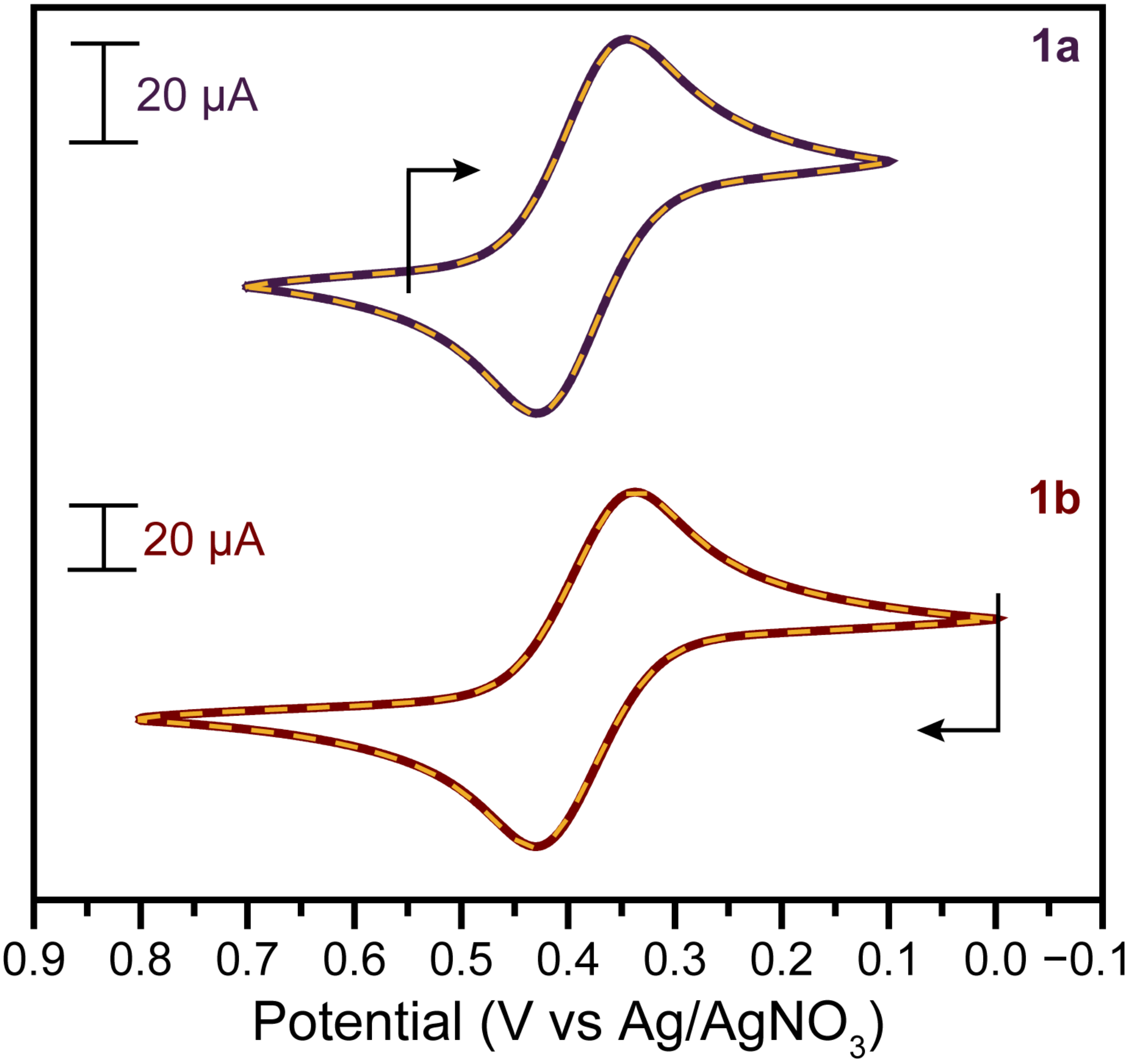

Figure S23. Comparison of cyclic voltammograms of 3.0 mM of **1a** and 3.0 mM of **1b** in dry MeCN containing 0.1 M (^{n}Bu$_4$N)(PF$_6$) supporting electrolyte collected at room temperature (23–25 °C) in the potential window containing the Cu^{II}/Cu^{I} redox couple using 100 mV s^{-1} scan rate. Vertical black lines and arrows denote the open-circuit potentials and scan directions, respectively. Purple and red voltammogram traces were collected without applying iR_u compensation, whereas the overlaid yellow dashed lines represent the voltammograms after applying 100% manual iR_u compensation as described in the Experimental Section. Glassy carbon, Ag/AgNO$_3$, and Pt mesh were used as working, reference, and counter electrodes, respectively.

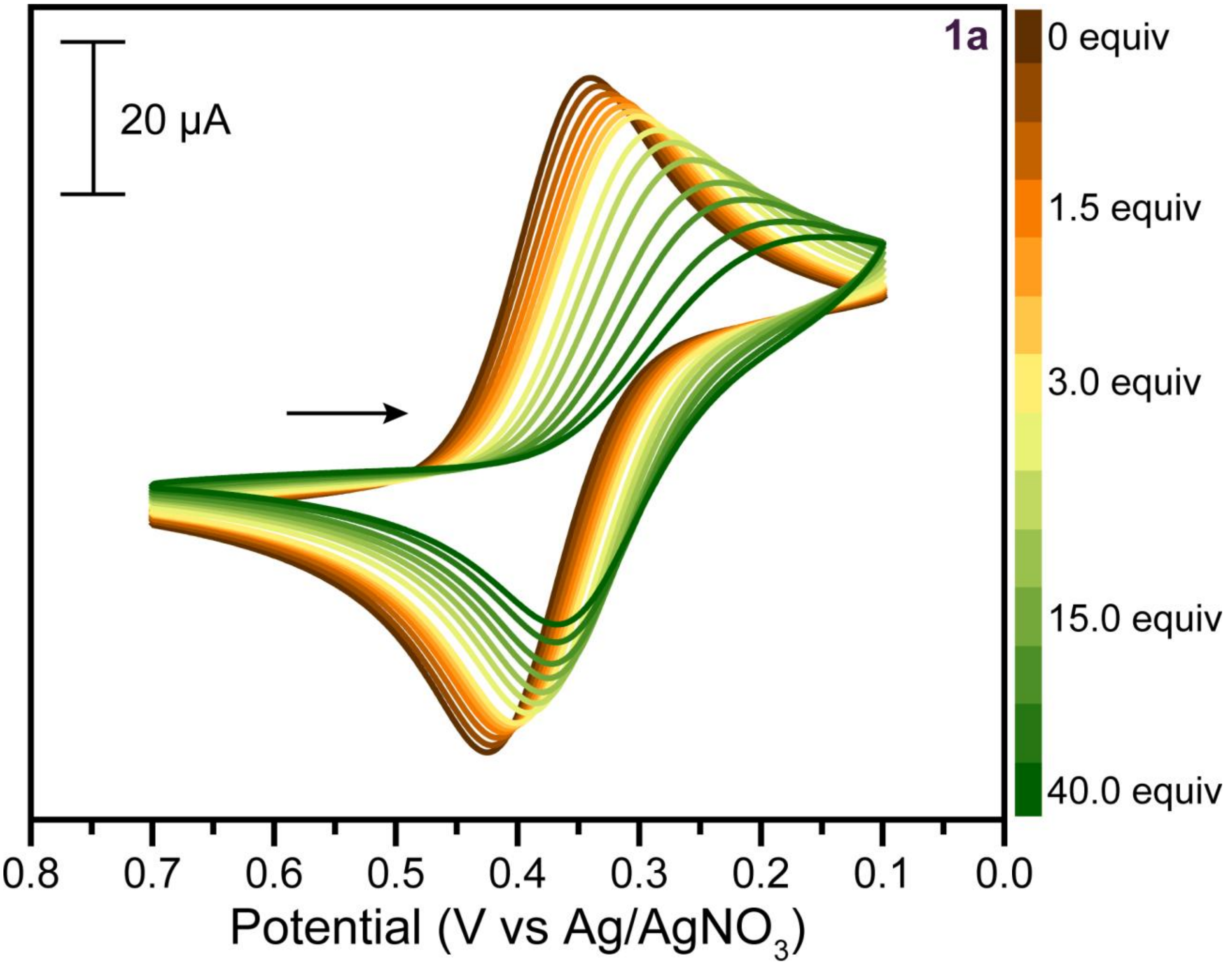

Figure S24. Cyclic voltammograms of 3 mM of **1a** in the absence and presence of variable amount of ^{t}Bu-py in dry MeCN containing 0.1 M (^{n}Bu$_4$N)(PF$_6$) supporting electrolyte collected at room temperature (23–25 °C) in the potential window containing the Cu^{II}/Cu^{I} redox couple using 100 mV s^{-1} scan rate. The color bar denotes the number of equiv of ^{t}Bu-py added to the sample solution. Note that the E_{OCP} changes with the addition of ^{t}Bu-py, thus, no vertical line denoting E_{OCP} is displayed. The black arrow indicates the scan direction. Glassy carbon, Ag/AgNO$_3$, and Pt mesh were used as working, reference, and counter electrodes, respectively.

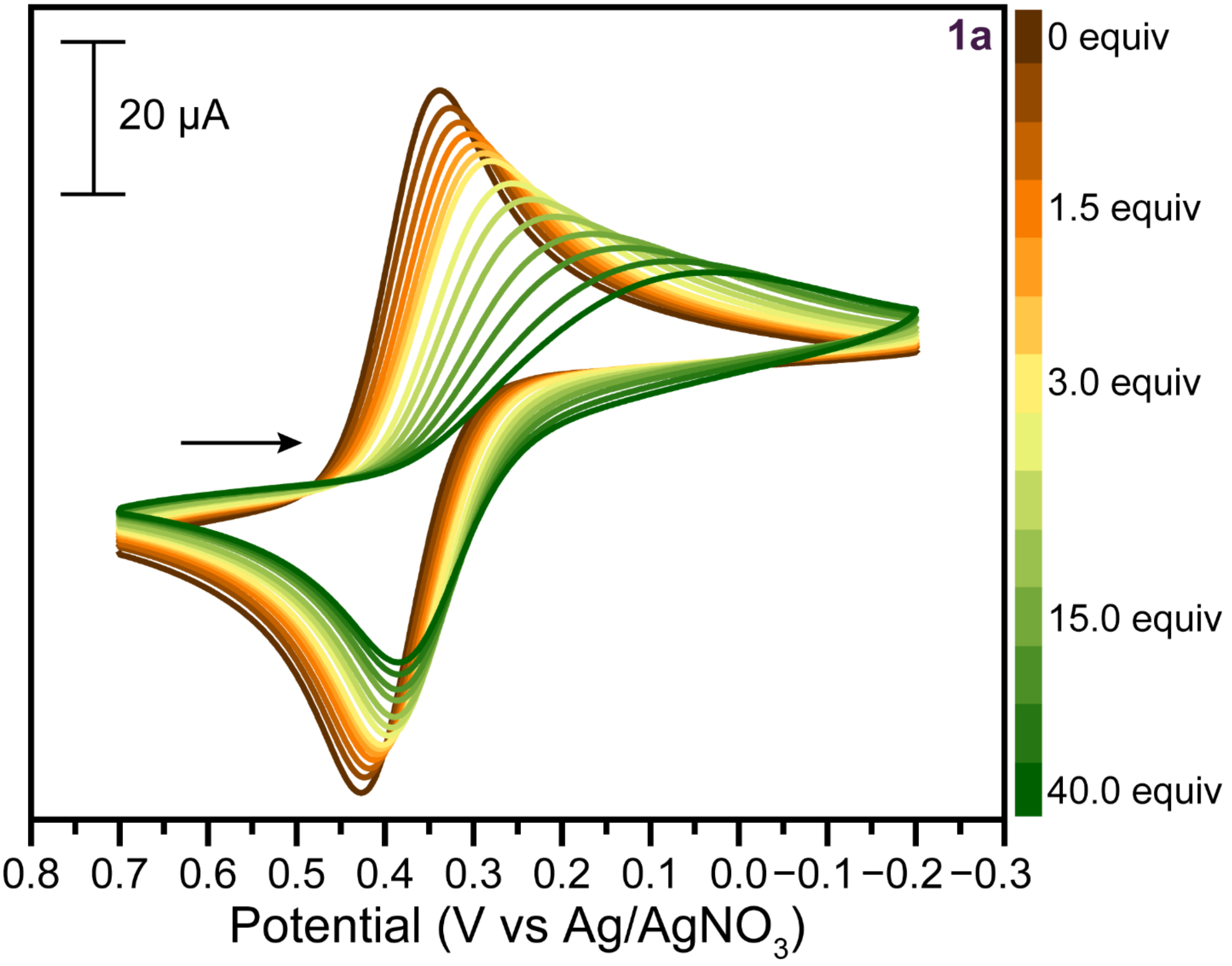

Figure S25. Cyclic voltammograms of 3 mM of **1a** in the absence and presence of variable amount of ^{t}Bu-py in dry MeCN containing 0.1 M (${}^{n}Bu_4N$)(PF_6) supporting electrolyte collected at room temperature (23–25 °C) in an extended potential window around the Cu^{II}/Cu^{I} redox couple using 100 mV s^{-1} scan rate. The color bar denotes the number of equiv of ^{t}Bu-py added to the sample solution. Note that the E_{OCP} changes with the addition of ^{t}Bu-py, thus, no vertical line denoting E_{OCP} is displayed. The black arrow indicates the scan direction. Glassy carbon, Ag/$AgNO_3$, and Pt mesh were used as working, reference, and counter electrodes, respectively.

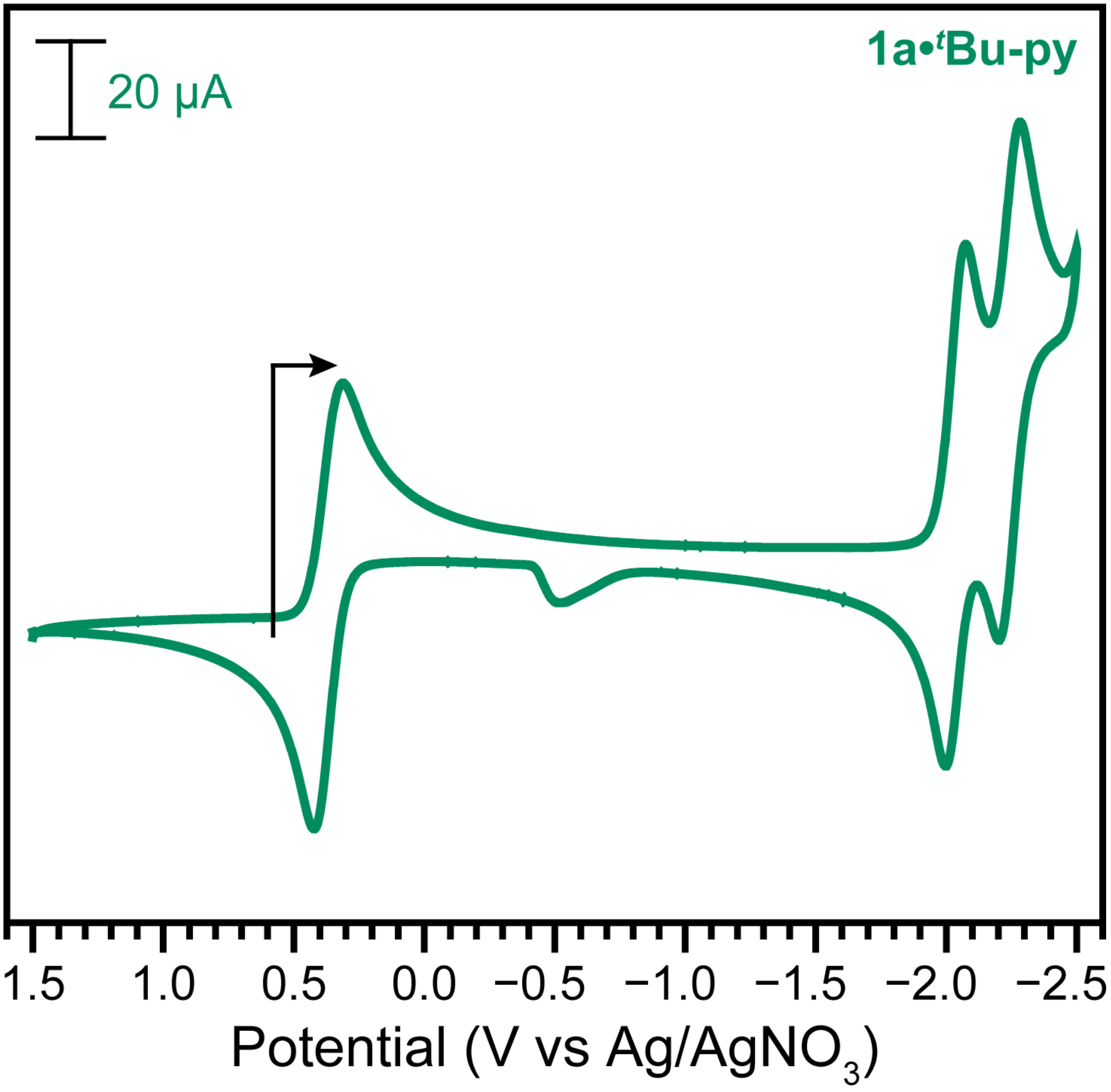

Figure S26. Cyclic voltammogram of 3.0 mM of **1a•^{t}Bu-py** in dry MeCN containing 0.1 M (^{n}Bu$_4$N)(PF$_6$) supporting electrolyte collected at room temperature (23–25 °C) using 100 mV s^{-1} scan rate. The vertical black line and arrow denote the open-circuit potential and scan direction, respectively. Glassy carbon, Ag/AgNO$_3$, and Pt mesh were used as working, reference, and counter electrodes, respectively.

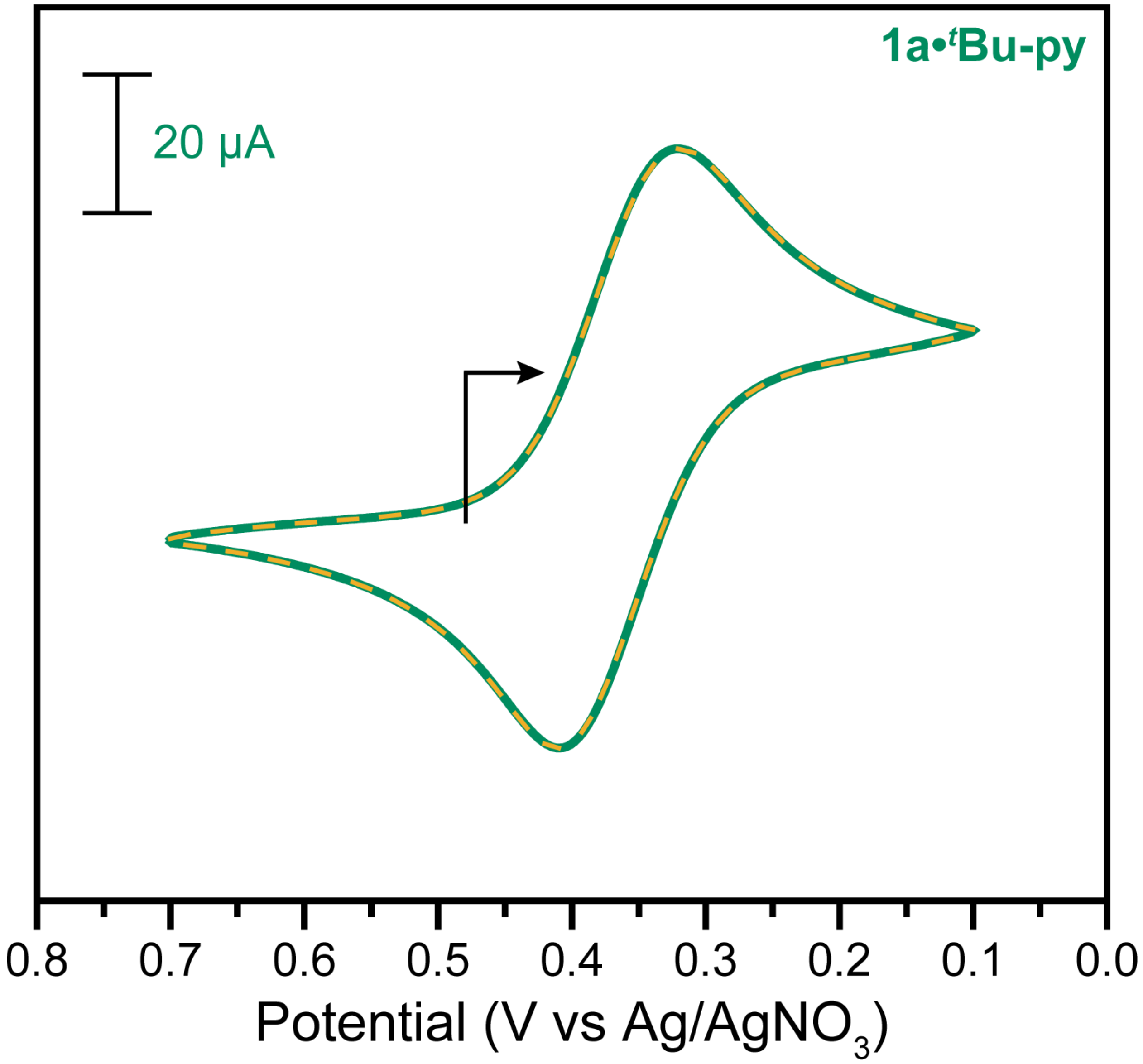

Figure S27. Cyclic voltammograms of 3.0 mM of **1a•^{t}Bu-py** in dry MeCN containing 0.1 M ($^{n}Bu_4N$)(PF_6) supporting electrolyte collected at room temperature (23–25 °C) in the potential window containing the Cu^{II}/Cu^{I} redox couple using 100 mV s^{-1} scan rate. The vertical black line and arrow denote the open-circuit potential and scan direction, respectively. The green voltammogram trace was collected without applying iR_u compensation, whereas the overlaid yellow dashed line represents the voltammogram after applying 100% manual iR_u compensation as described in the Experimental Section. Glassy carbon, Ag/$AgNO_3$, and Pt mesh were used as working, reference, and counter electrodes, respectively.

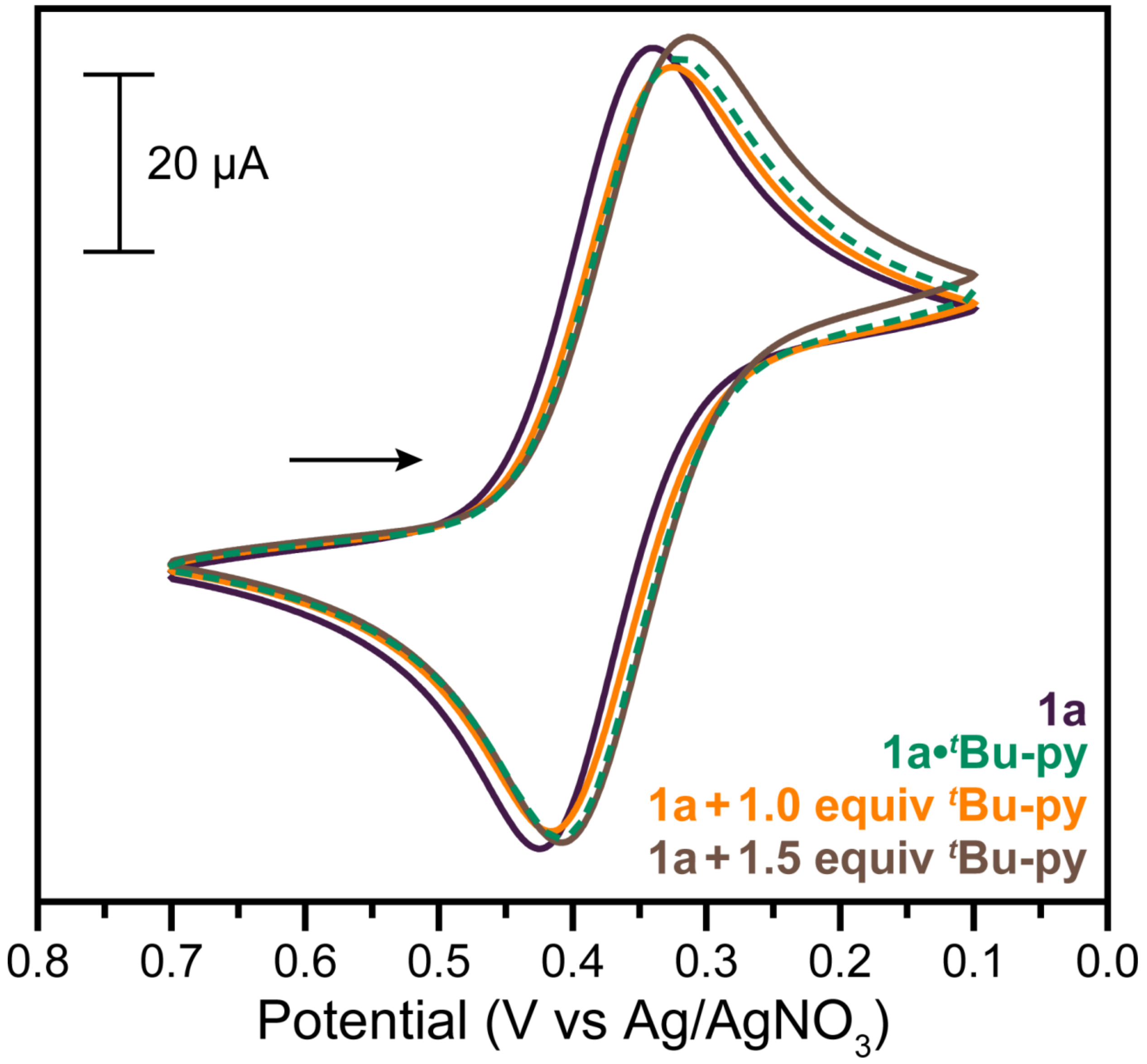

Figure S28. Comparison of cyclic voltammograms of 3.0 mM of **1a** in the absence and presence of 1.0 and 1.5 equiv of ^{t}Bu-py and 3.0 mM of **1a•^{t}Bu-py** in dry MeCN containing 0.1 M ($^{n}Bu_4N$)(PF_6) supporting electrolyte collected at ~23 °C in the potential window containing the Cu^{II}/Cu^{I} redox couple using 100 mV s^{-1} scan rate. Note that the E_{OCP} changes between the sample solutions, thus, no vertical line denoting E_{OCP} is displayed. The black arrow indicates the scan direction. Glassy carbon, Ag/$AgNO_3$, and Pt mesh were used as working, reference, and counter electrodes, respectively.

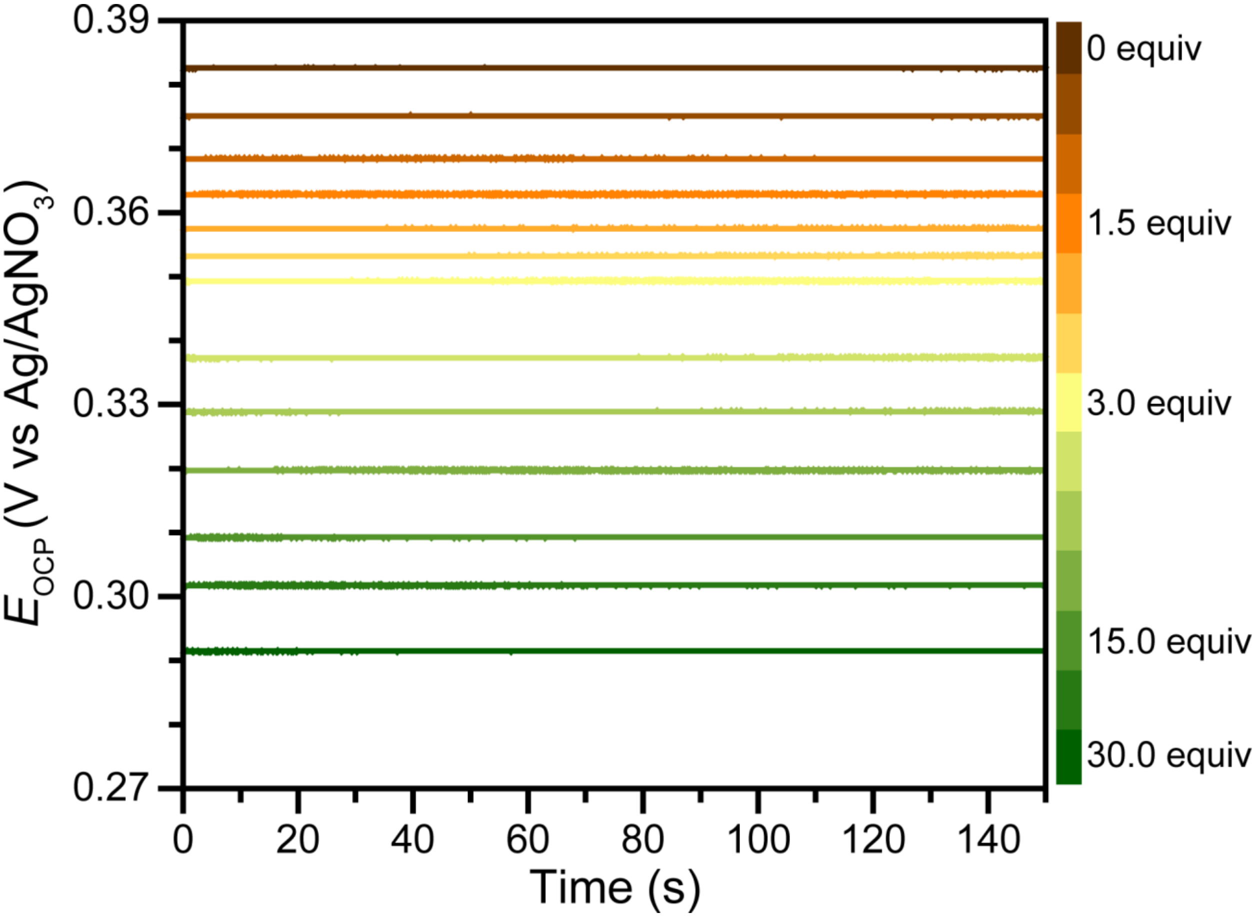

Figure S29. Plot of the time dependence of E_{OCP} for an equimolar solution of **1a** and **1b** (3 mM total) in the absence and presence of variable amount of ^{t}Bu-py in dry MeCN containing 0.1 M ($^{n}Bu_4N$)(PF_6) supporting electrolyte collected at room temperature (23–25 °C). The color bar denotes the number of equiv of ^{t}Bu-py added to the sample solution. Glassy carbon, Ag/$AgNO_3$, and Pt mesh were used as working, reference, and counter electrodes, respectively.

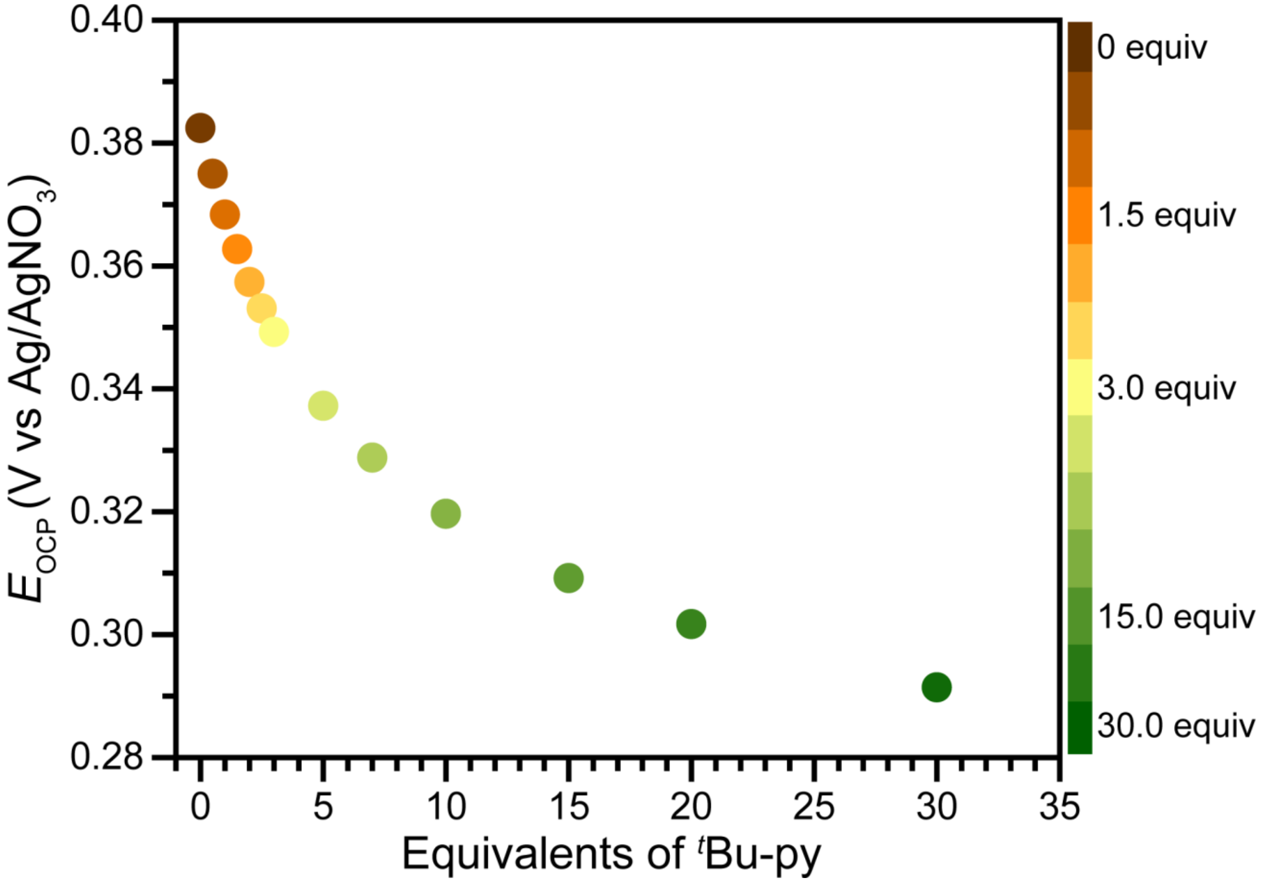

Figure S30. Plot of E_{OCP} vs equivalents of ^{t}Bu-py added for the data shown in Figure S29. The color bar denotes the number of equiv of ^{t}Bu-py added to the sample solution.

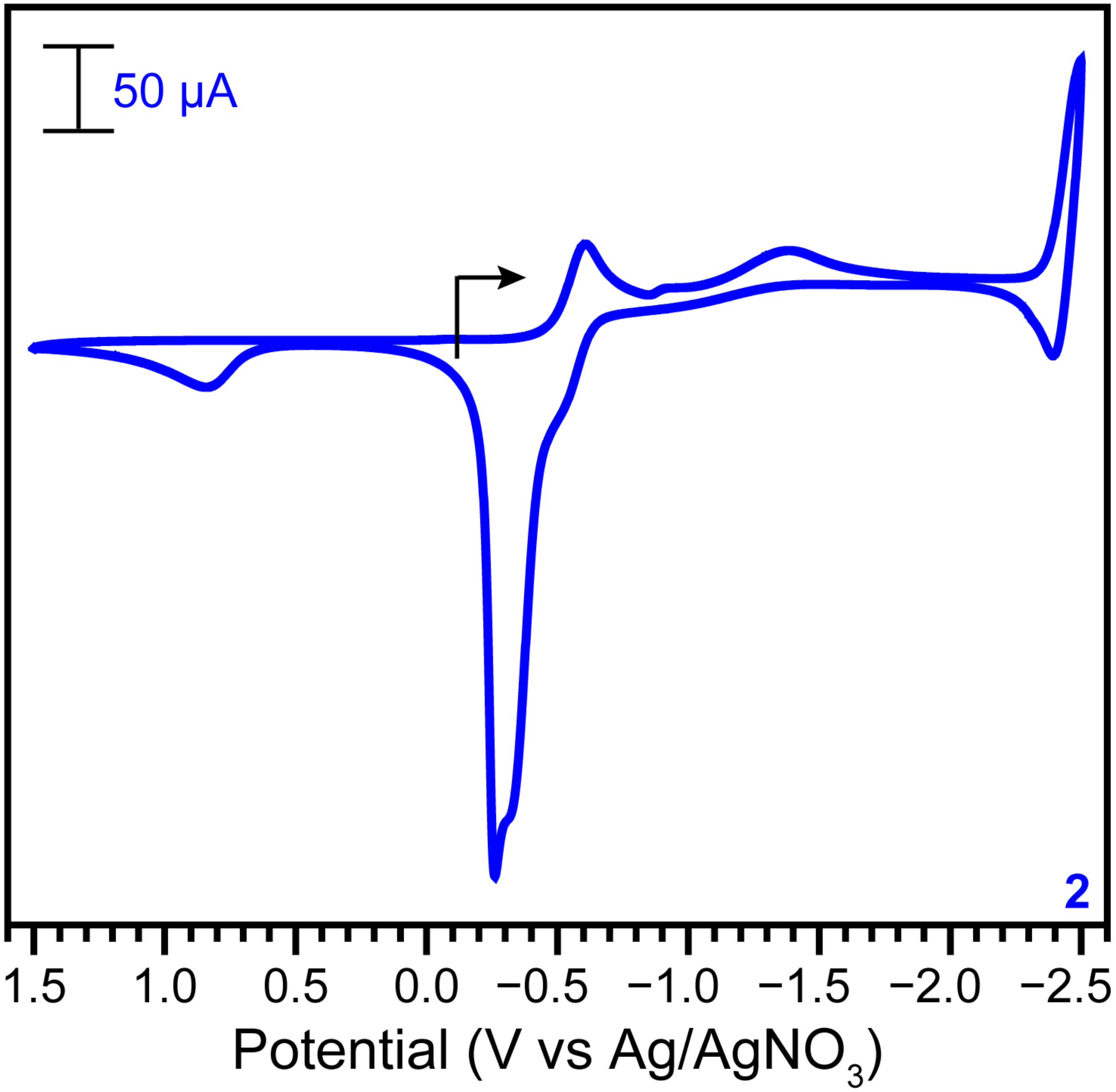

Figure S31. Cyclic voltammogram of 3.0 mM of **2** in dry MeCN containing 0.1 M (^{n}Bu$_4$N)(PF$_6$) supporting electrolyte collected at room temperature (23–25 °C) using 100 mV s^{-1} scan rate. The vertical black line and arrow denote the open-circuit potential and scan direction, respectively. Glassy carbon, Ag/AgNO$_3$, and Pt mesh were used as working, reference, and counter electrodes, respectively.

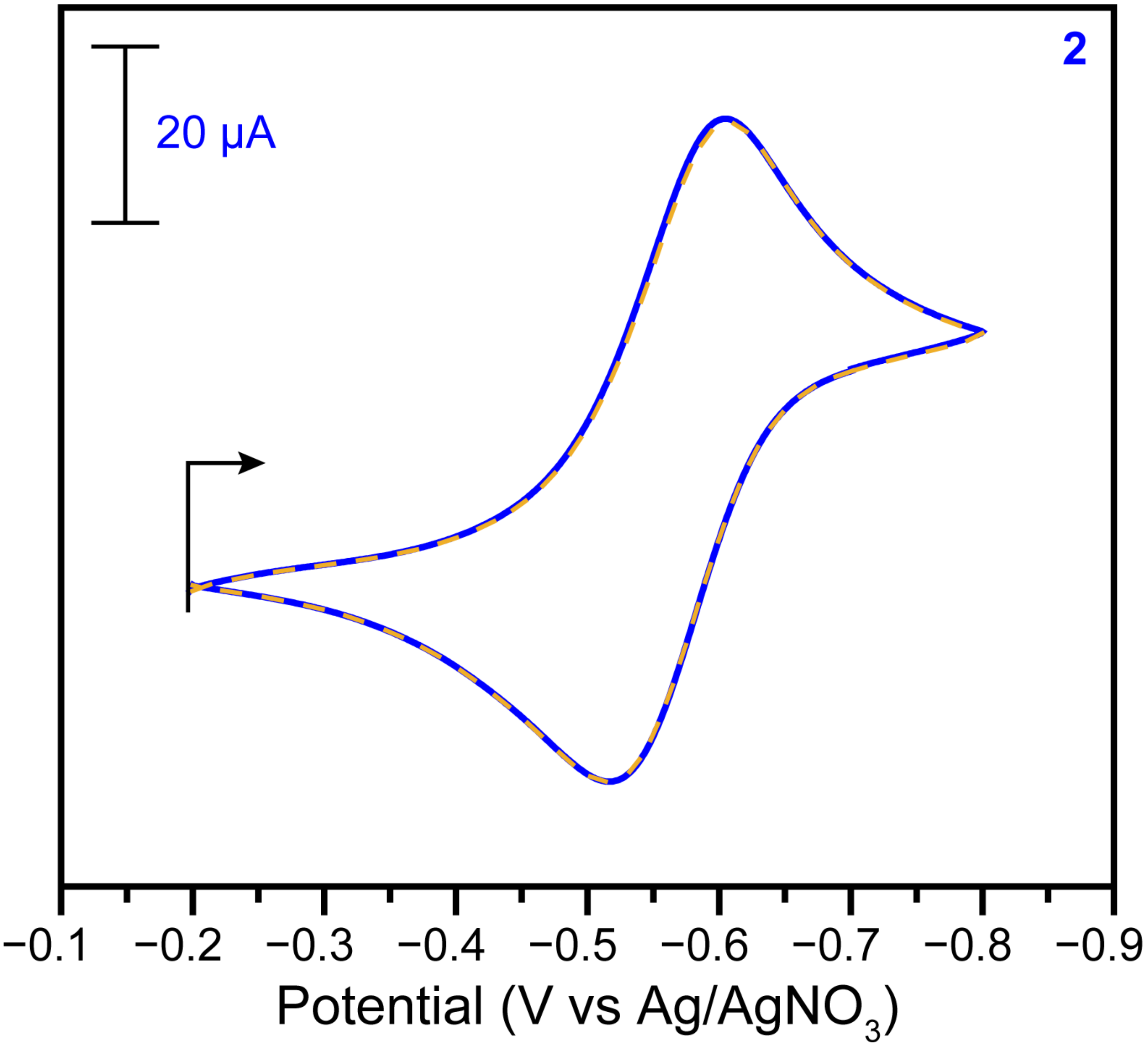

Figure S32. Cyclic voltammograms of 3.0 mM of **2** in dry MeCN containing 0.1 M ($^{n}Bu_4N$)(PF_6) supporting electrolyte collected at room temperature (23–25 °C) in the potential window containing the Cu^{II}/Cu^{I} redox couple using 100 mV s^{-1} scan rate. The vertical black line and arrow denote the open-circuit potential and scan direction, respectively. The blue voltammogram trace was collected without applying iR_u compensation, whereas the overlaid yellow dashed line represents the voltammogram after applying 100% manual iR_u compensation as described in the Experimental Section. Glassy carbon, Ag/$AgNO_3$, and Pt mesh were used as working, reference, and counter electrodes, respectively.

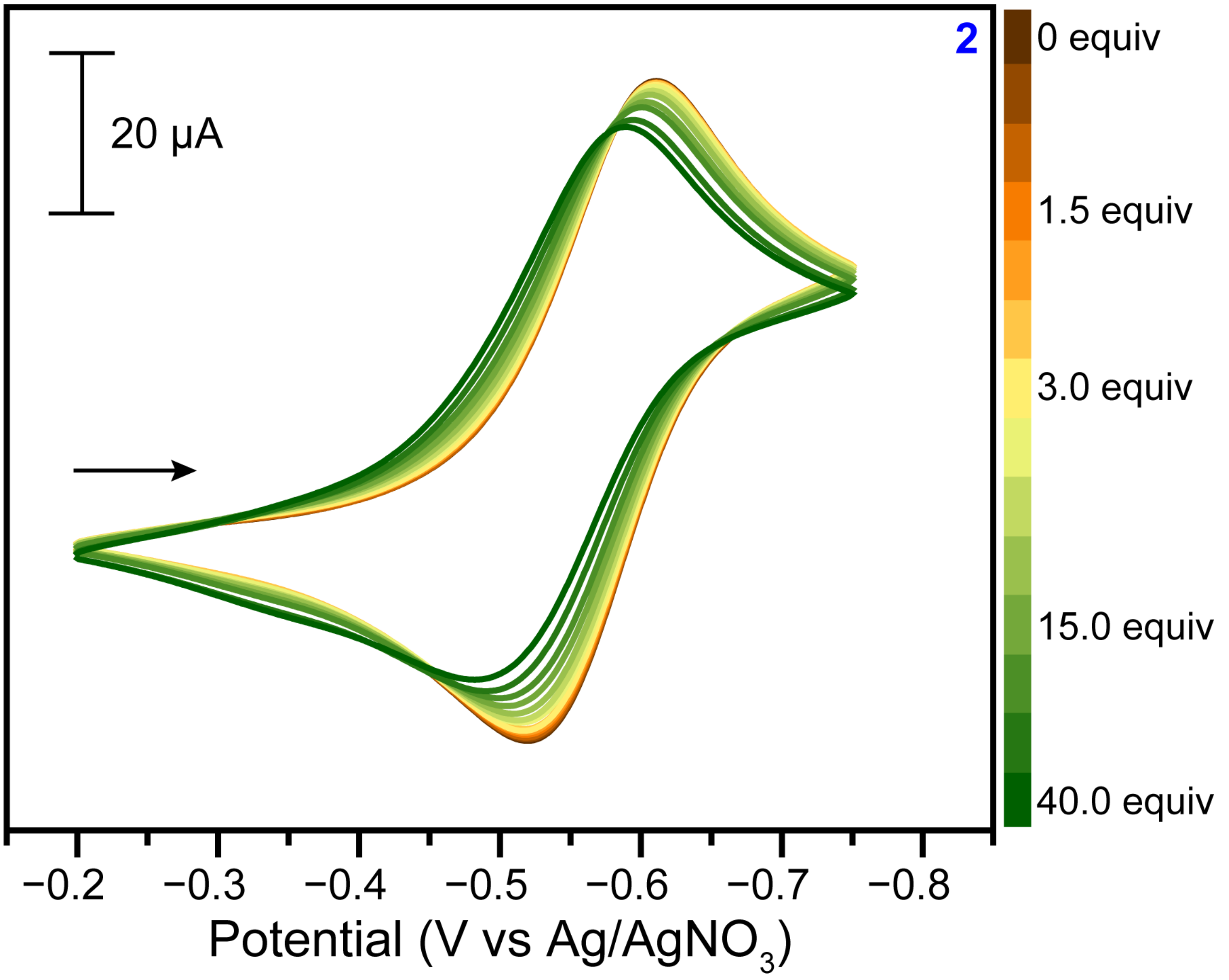

Figure S33. Cyclic voltammograms of 3 mM of **2** in the absence and presence of variable amount of ^{t}Bu-py in dry MeCN containing 0.1 M (^{n}Bu$_4$N)(PF$_6$) supporting electrolyte collected at room temperature (23–25 °C) in the potential window containing the Cu^{II}/Cu^{I} redox couple using 100 mV s^{-1} scan rate. The color bar denotes the number of equiv of ^{t}Bu-py added to the sample solution. Note that the E_{OCP} changes with the addition of ^{t}Bu-py, thus, no vertical line denoting E_{OCP} is displayed. The black arrow indicates the scan direction. Glassy carbon, Ag/AgNO$_3$, and Pt mesh were used as working, reference, and counter electrodes, respectively.

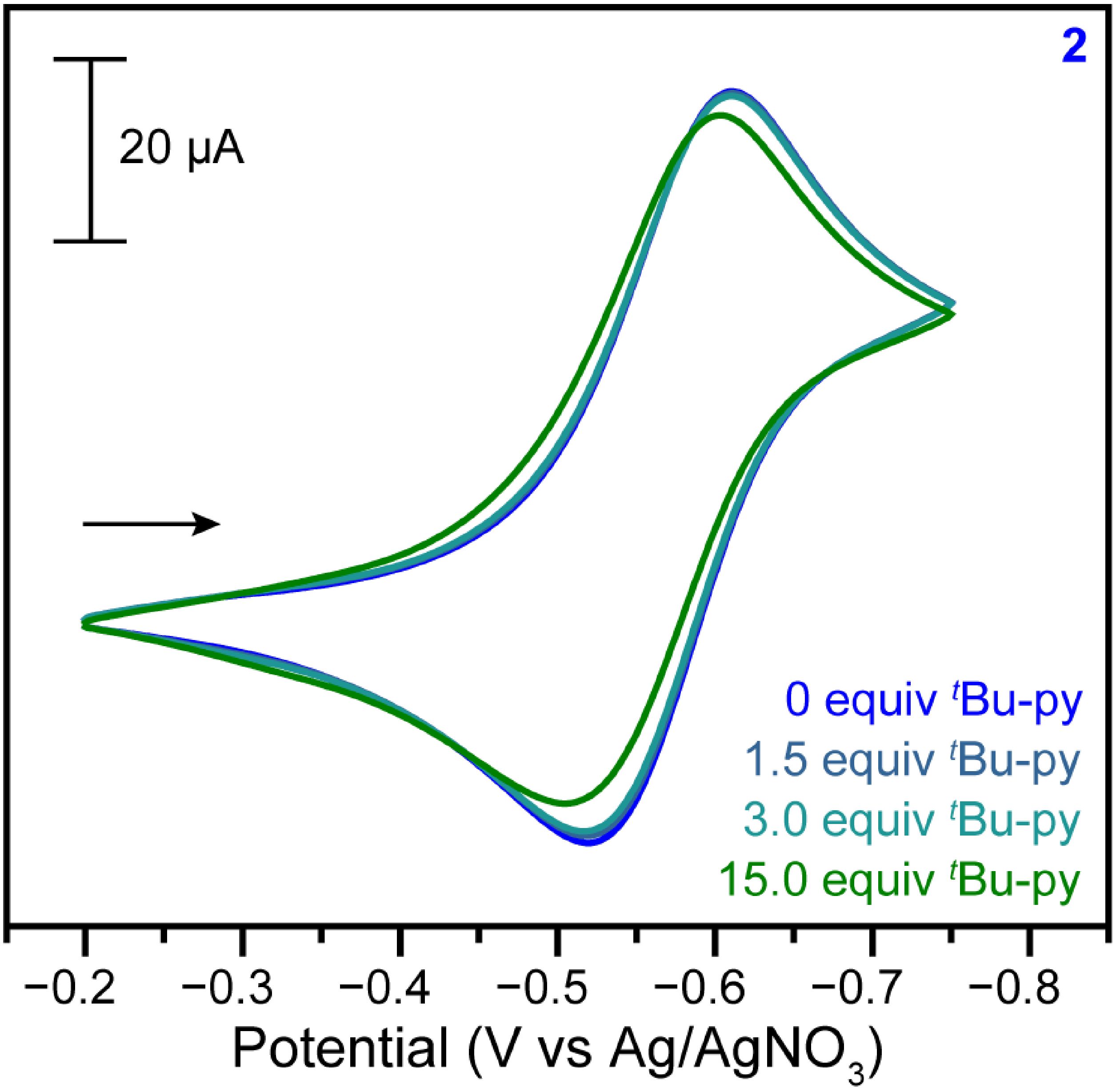

Figure S34. Cyclic voltammograms of 3.0 mM of **2** in the absence and presence of 1.5, 3.0, and 15.0 equiv of ^{t}Bu-py in dry MeCN containing 0.1 M (^{n}Bu$_4$N)(PF$_6$) supporting electrolyte collected at room temperature (23–25 °C) in the potential window containing the Cu^{II}/Cu^{I} redox couple using 100 mV s^{-1} scan rate. Note that the E_{OCP} changes with the addition of ^{t}Bu-py, thus, no vertical line denoting E_{OCP} is displayed. The black arrow indicates the scan direction. Glassy carbon, Ag/AgNO$_3$, and Pt mesh were used as working, reference, and counter electrodes, respectively.

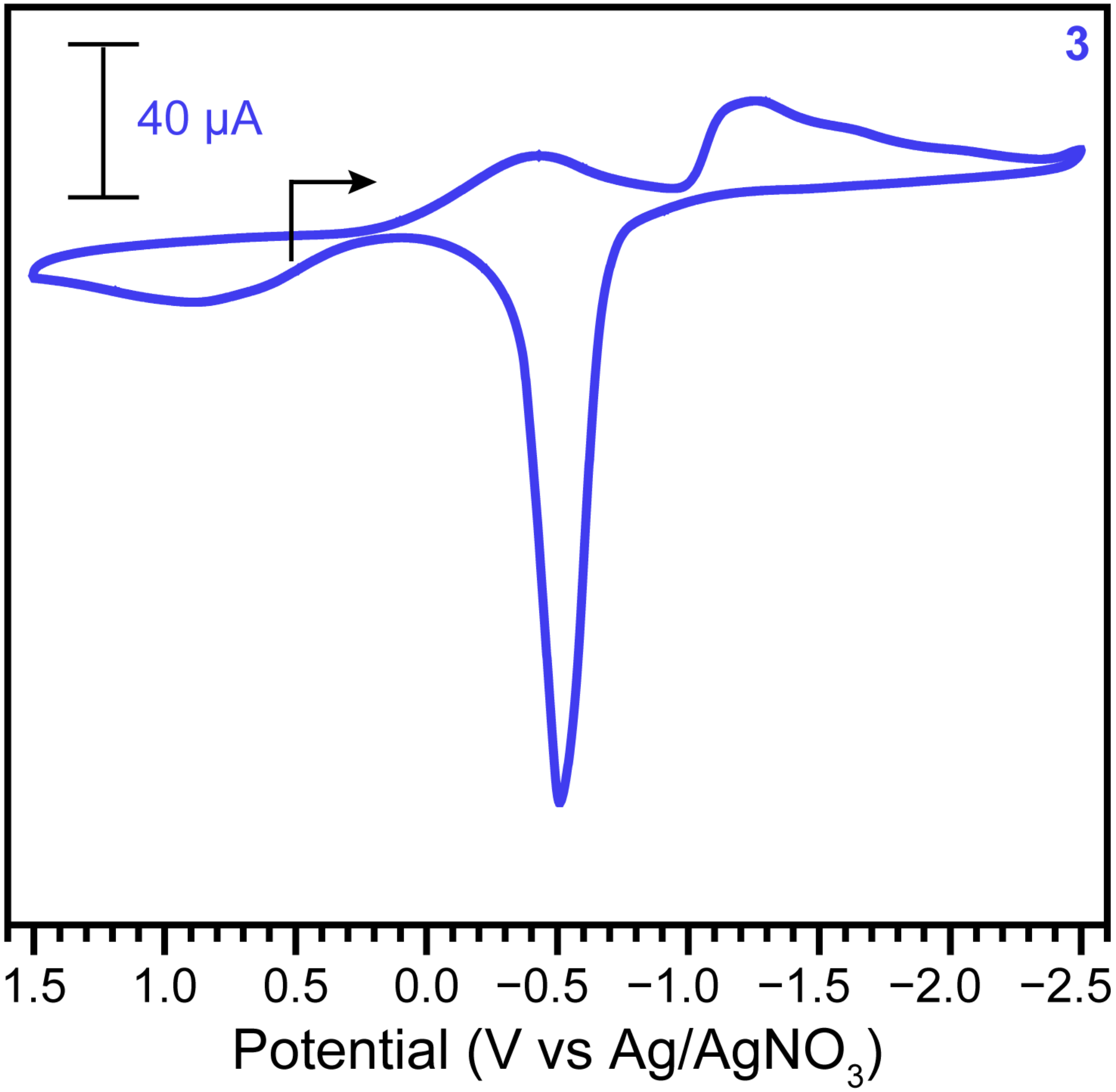

Figure S35. Cyclic voltammogram of 3.0 mM of **3** in dry MeCN containing 0.1 M (^{n}Bu$_4$N)(PF$_6$) supporting electrolyte collected at room temperature (23–25 °C) using 100 mV s^{-1} scan rate. The vertical black line and arrow denote the open-circuit potential and scan direction, respectively. Glassy carbon, Ag/AgNO$_3$, and Pt mesh were used as working, reference, and counter electrodes, respectively.

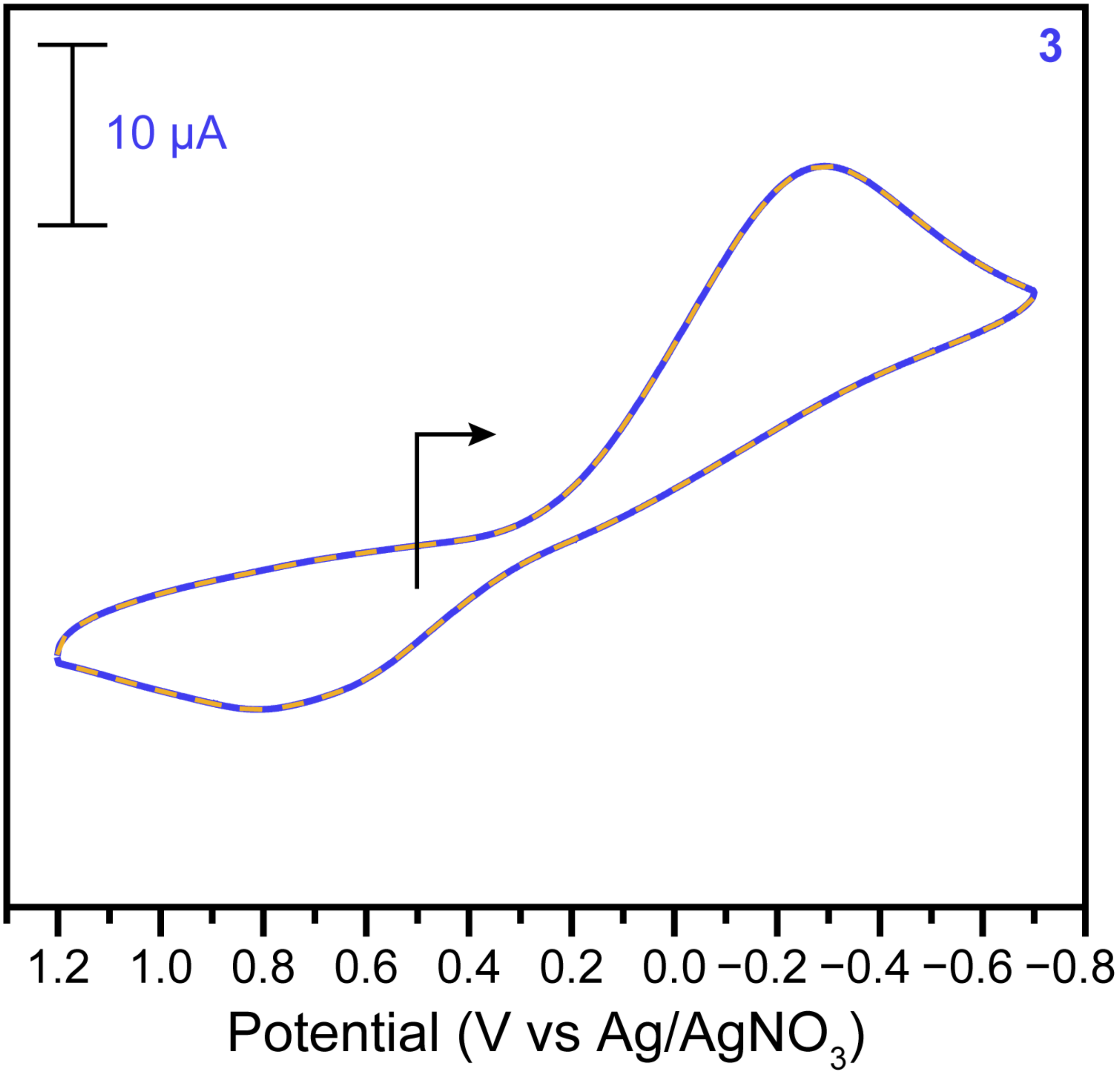

Figure S36. Cyclic voltammograms of 3.0 mM of **3** in dry MeCN containing 0.1 M (^{n}Bu$_4$N)(PF$_6$) supporting electrolyte collected at room temperature (23–25 °C) in the potential window containing the Cu^{II}/Cu^{I} redox couple using 100 mV s^{-1} scan rate. The vertical black line and arrow denote the open-circuit potential and scan direction, respectively. The violet-blue voltammogram trace was collected without applying *i*R_u compensation, whereas the overlaid yellow dashed line represents the voltammogram after applying 100% manual *i*R_u compensation as described in the Experimental Section. Glassy carbon, Ag/AgNO$_3$, and Pt mesh were used as working, reference, and counter electrodes, respectively.

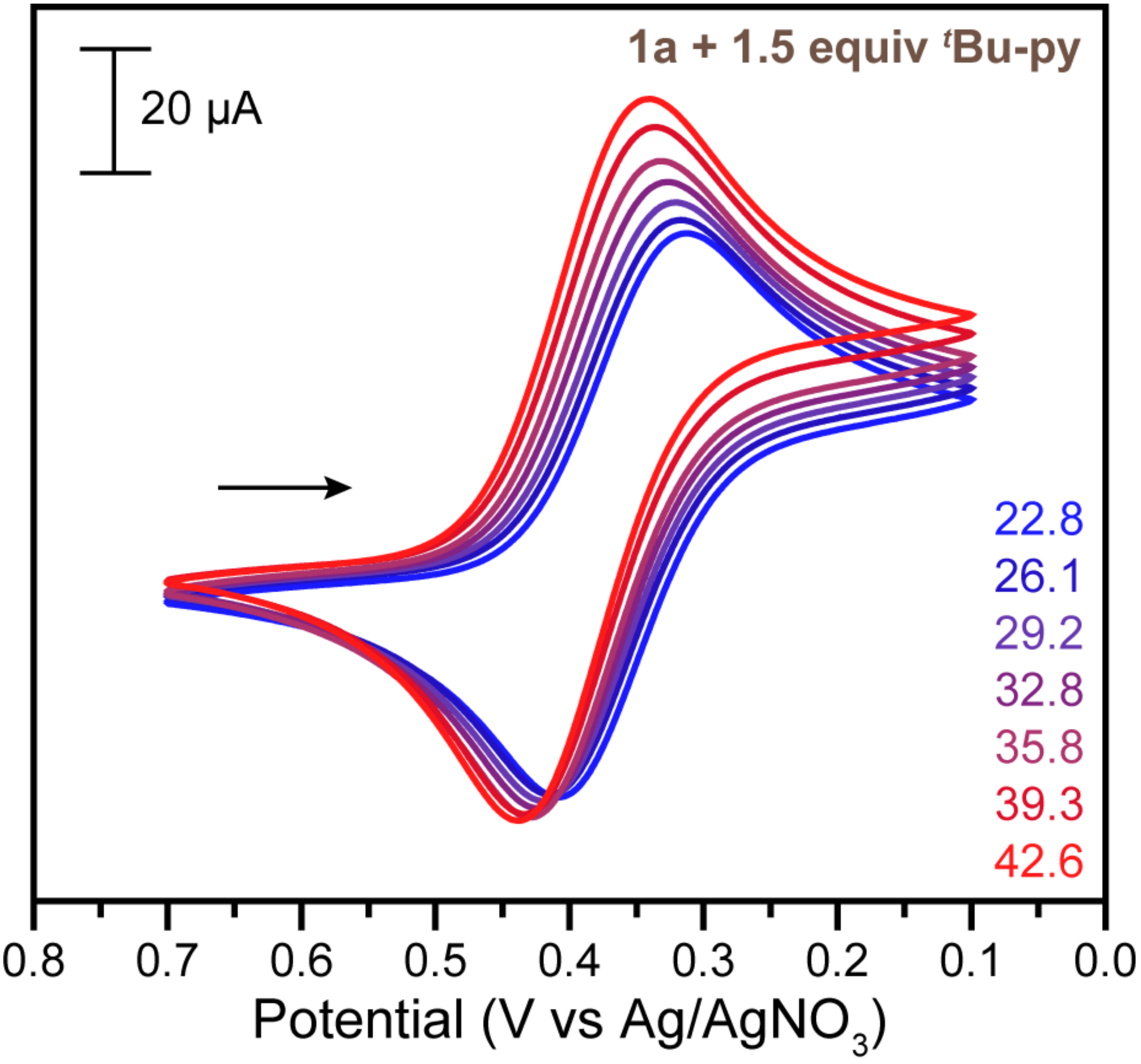

Figure S37. Cyclic voltammograms of 3 mM of **1a** in the presence of 1.5 equiv of ^{t}Bu-py in dry MeCN containing 0.1 M (^{n}Bu$_4$N)(PF$_6$) supporting electrolyte collected at variable temperatures (~23–43 °C) in the potential window containing the Cu^{II}/Cu^{I} redox couple using 100 mV s^{-1} scan rate. Colored numbers denote the temperature of the solution for each measurement in °C. Note that the E_{OCP} changes with temperature, thus, no vertical line denoting E_{OCP} is displayed. The black arrow indicates the scan direction. Glassy carbon, Ag/AgNO$_3$, and Pt mesh were used as working, reference, and counter electrodes, respectively.

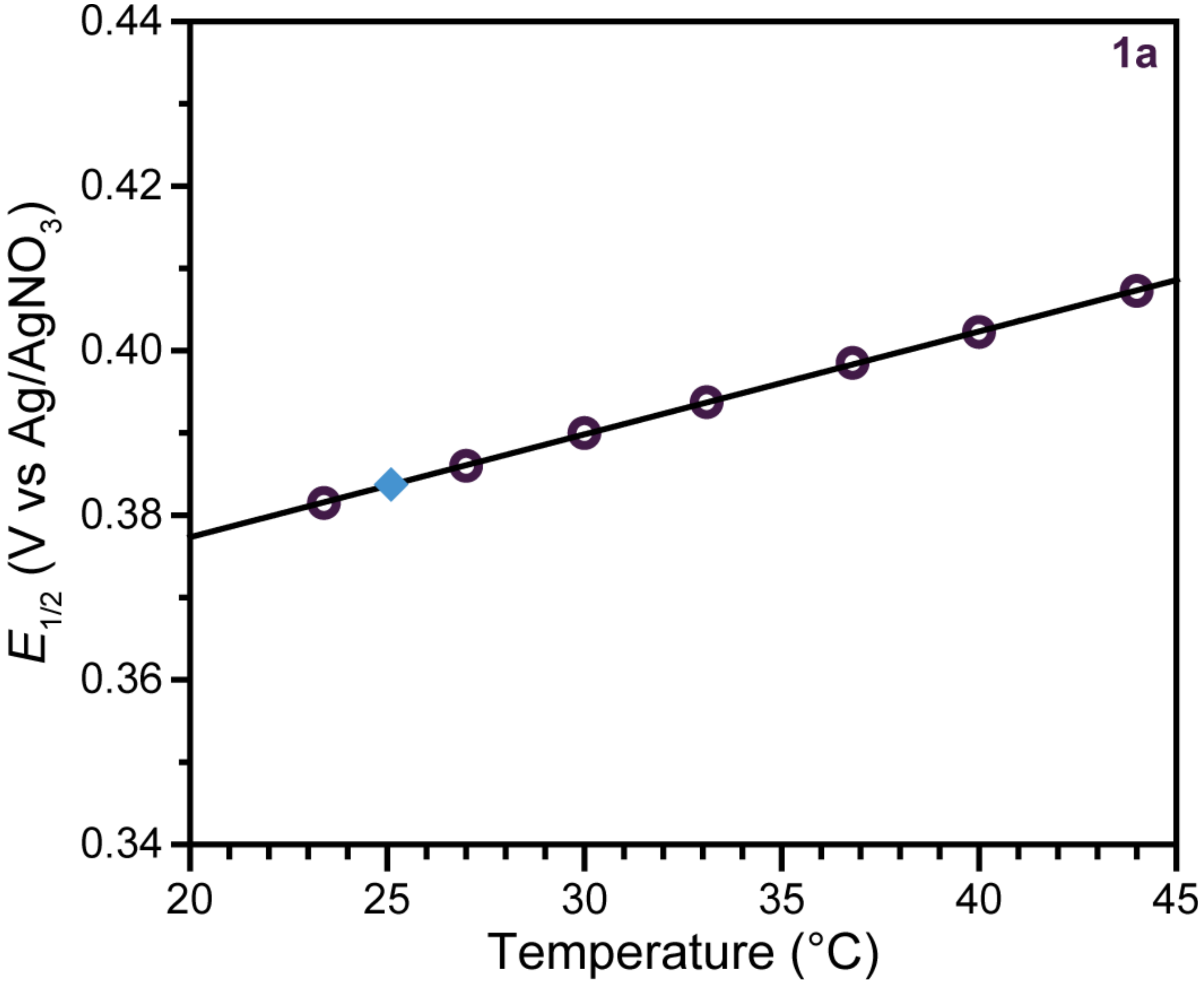

Figure S38. Plot of the temperature dependence of $E_{1/2}$ for the Cu^{II}/Cu^{I} redox couple of **1a** in dry MeCN containing 0.1 M ($^{n}Bu_4N$)(PF_6) supporting electrolyte, as obtained from the variable-temperature cyclic voltammetry data shown in Figure 4, left. Colored circles denote experimental data from the variable-temperature experiment in heating mode, and the black line corresponds to a linear fit to the data. The average slope from four independent measurements after correcting for the temperature coefficient of the reference electrode potential (eqs S9 and S10), which represents the average temperature coefficient, is α = 1.70(6) mV °C^{-1} (Table 1). The error in the average slope corresponds to the standard deviation of individual measurements. The blue diamond corresponds to a data point measured after cooling the solution back down to close to room temperature.

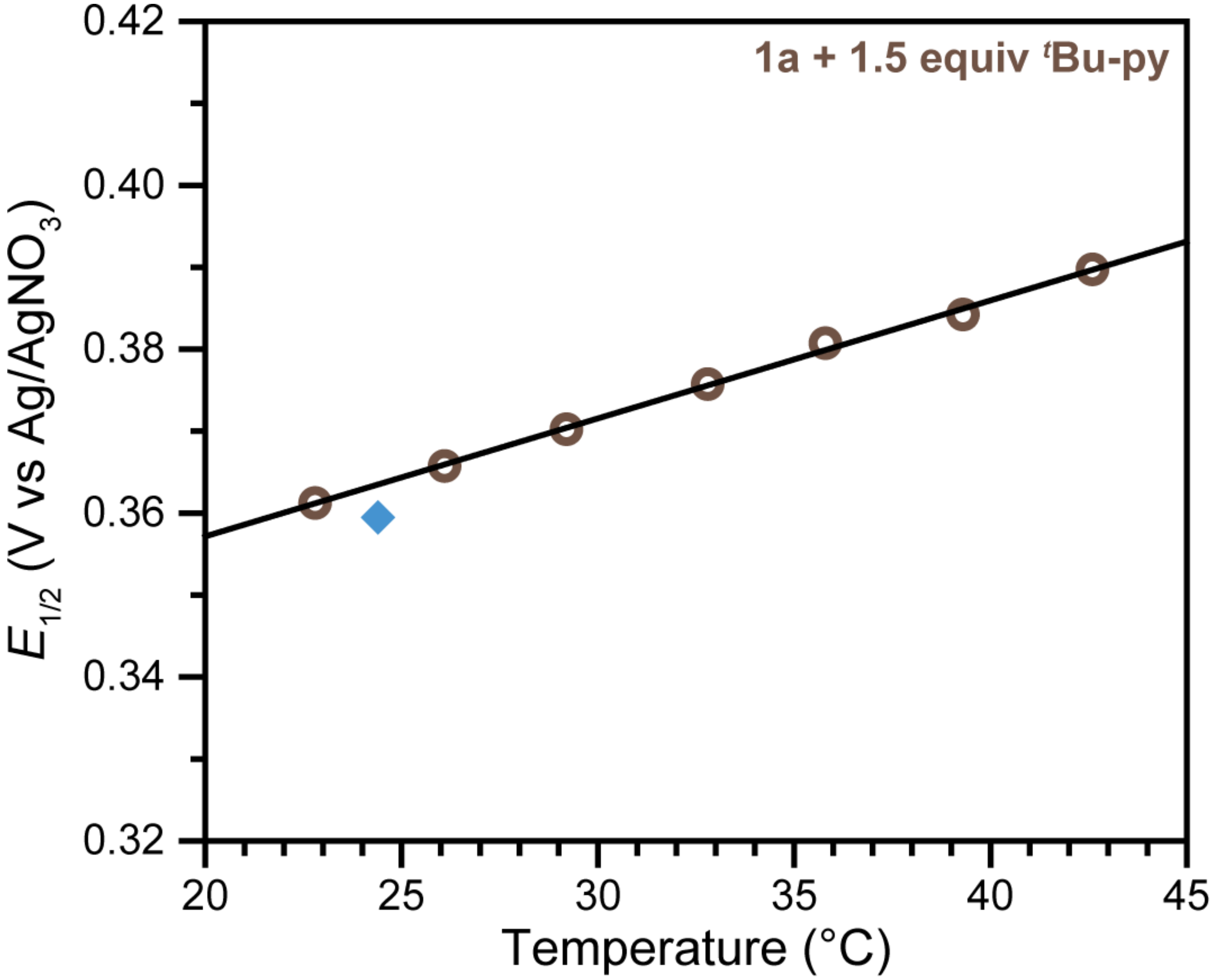

Figure S39. Plot of the temperature dependence of $E_{1/2}$ for the Cu^{II}/Cu^{I} redox couple of **1a** in the presence of 1.5 equiv of ^{t}Bu-py in dry MeCN containing 0.1 M ($^{n}Bu_4N$)(PF_6) supporting electrolyte, as obtained from the variable-temperature cyclic voltammetry data shown in Figure S37. Colored circles denote experimental data from the variable-temperature experiment in heating mode, and the black line corresponds to a linear fit to the data. The average slope from three independent measurements after correcting for the temperature coefficient of the reference electrode potential (eqs S9 and S10), which represents the average temperature coefficient, is α = 2.02(7) mV °C^{-1} (Table 1). The error in the average slope corresponds to the standard deviation of individual measurements. The blue diamond corresponds to a data point measured after cooling the solution back down to close to room temperature.

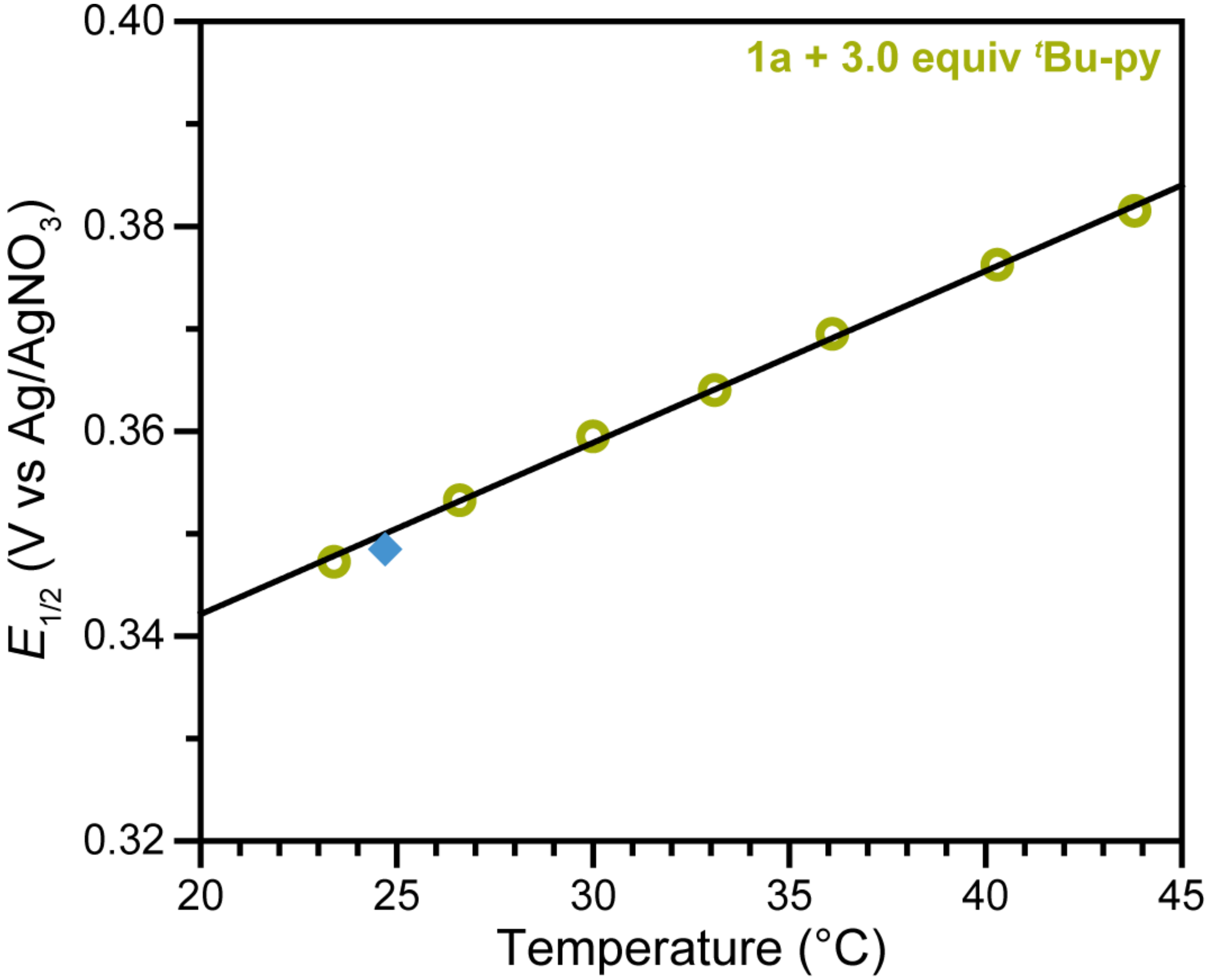

Figure S40. Plot of the temperature dependence of $E_{1/2}$ for the Cu^{II}/Cu^{I} redox couple of **1a** in the presence of 3.0 equiv of ^{t}Bu-py in dry MeCN containing 0.1 M ($^n Bu_4N$)(PF_6) supporting electrolyte, as obtained from the variable-temperature cyclic voltammetry data shown in Figure 4, center. Colored circles denote experimental data from the variable-temperature experiment in heating mode, and the black line corresponds to a linear fit to the data. The average slope from three independent measurements after correcting for the temperature coefficient of the reference electrode potential (eqs S9 and S10), which represents the average temperature coefficient, is α = 2.15(7) mV °C^{-1} (Table 1). The error in the average slope corresponds to the standard deviation of individual measurements. The blue diamond corresponds to a data point measured after cooling the solution back down to close to room temperature.

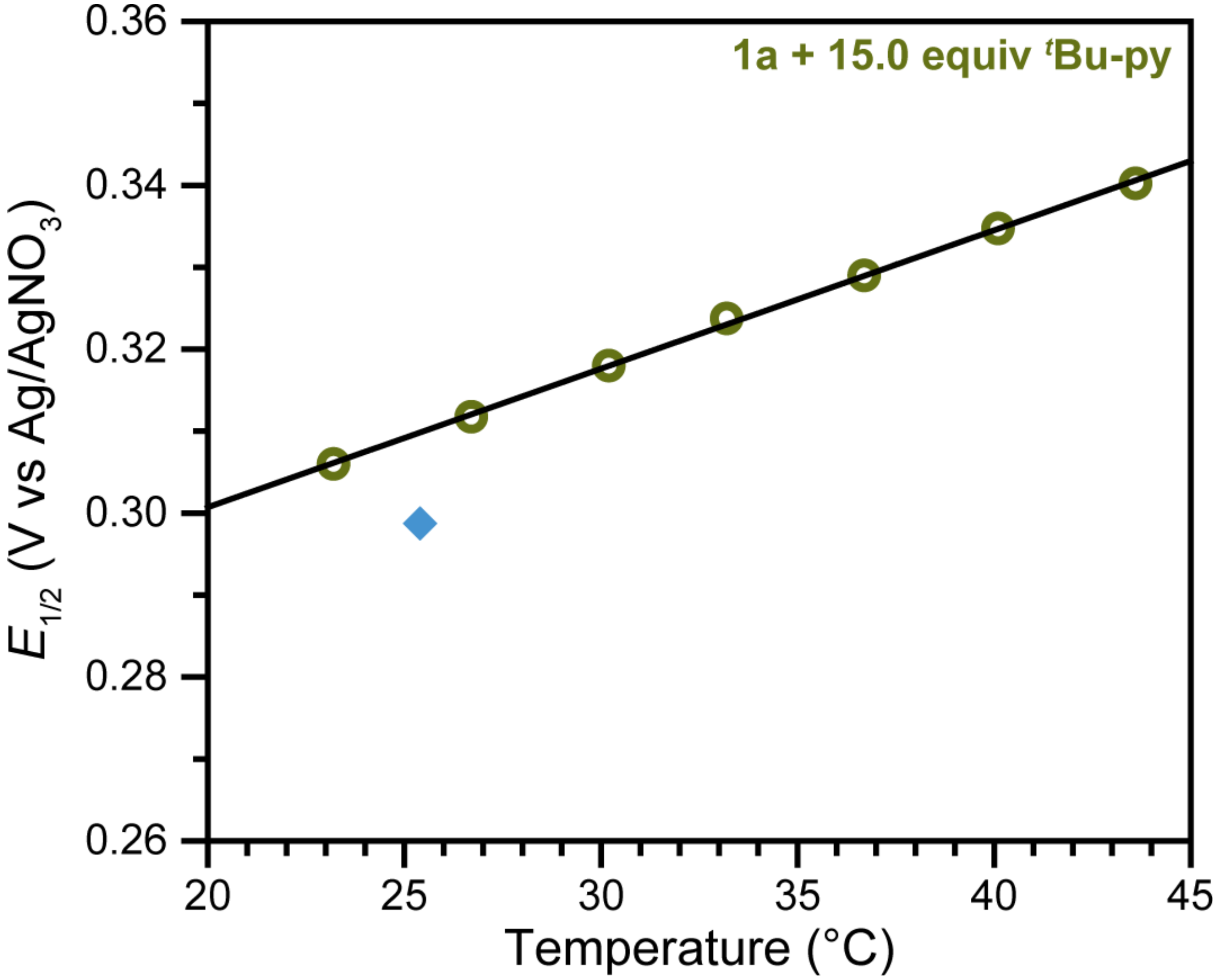

Figure S41. Plot of the temperature dependence of $E_{1/2}$ for the Cu^{II}/Cu^{I} redox couple of **1a** in the presence of 15.0 equiv of ^{t}Bu-py in dry MeCN containing 0.1 M ($^{n}Bu_4N$)(PF_6) supporting electrolyte, as obtained from the variable-temperature cyclic voltammetry data shown in Figure 4, right. Colored circles denote experimental data from the variable-temperature experiment in heating mode, and the black line corresponds to a linear fit to the data. The average slope from four independent measurements after correcting for the temperature coefficient of the reference electrode potential (eqs S9 and S10), which represents the average temperature coefficient, is α = 2.3(2) mV °C^{-1} (Table 1). The error in the average slope corresponds to the standard deviation of individual measurements. The blue diamond corresponds to a data point measured after cooling the solution back down to close to room temperature.

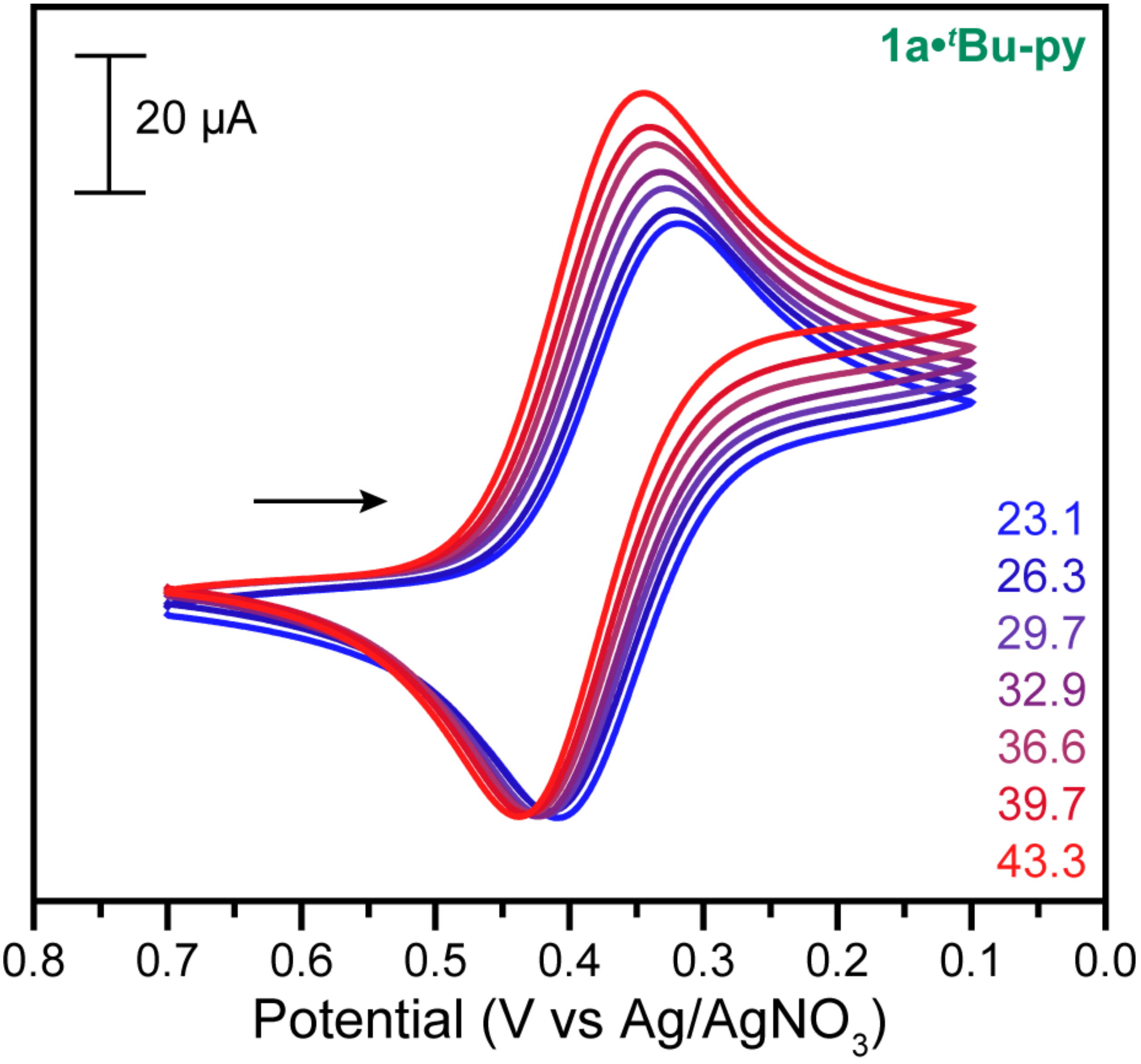

Figure S42. Cyclic voltammograms of 3 mM of **1a•^{t}Bu-py** in dry MeCN containing 0.1 M (^{n}Bu$_4$N)(PF$_6$) supporting electrolyte collected at variable temperatures (~23–43 °C) in the potential window containing the Cu^{II}/Cu^{I} redox couple using 100 mV s^{-1} scan rate. Colored numbers denote the temperature of the solution for each measurement in °C. Note that the E_{OCP} changes with temperature, thus, no vertical line denoting E_{OCP} is displayed. The black arrow indicates the scan direction. Glassy carbon, Ag/AgNO$_3$, and Pt mesh were used as working, reference, and counter electrodes, respectively.

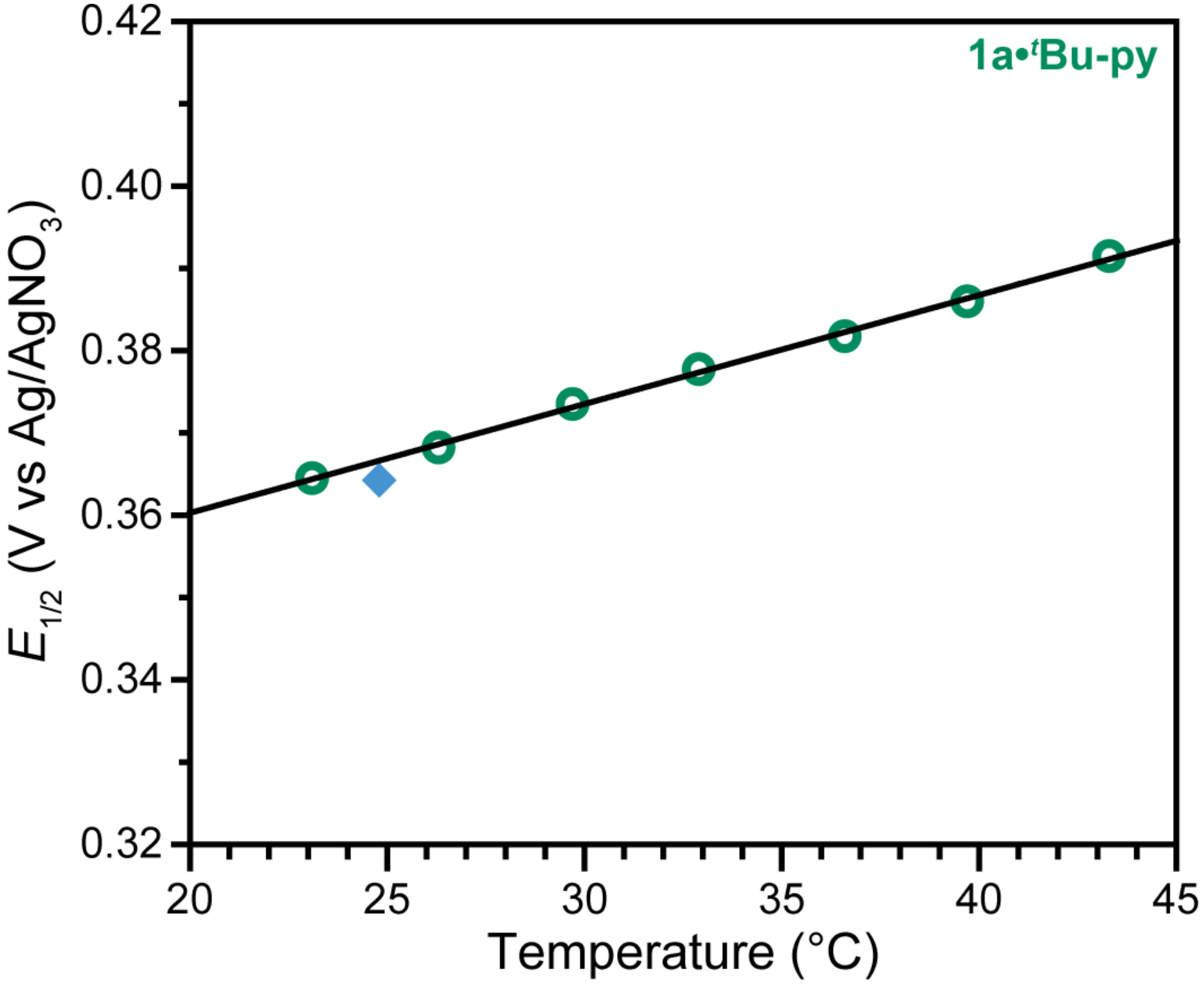

Figure S43. Plot of the temperature dependence of $E_{1/2}$ for the Cu^{II}/Cu^{I} redox couple of **1a•^{t}Bu-py** in dry MeCN containing 0.1 M (^{n}Bu$_4$N)(PF$_6$) supporting electrolyte, as obtained from the variable-temperature cyclic voltammetry data shown in Figure S42. Colored circles denote experimental data from the variable-temperature experiment in heating mode, and the black line corresponds to a linear fit to the data. The average slope from three independent measurements after correcting for the temperature coefficient of the reference electrode potential (eqs S9 and S10), which represents the average temperature coefficient, is α = 1.91(7) mV °C^{-1} (Table 1). The error in the average slope corresponds to the standard deviation of individual measurements. The blue diamond corresponds to a data point measured after cooling the solution back down to close to room temperature.

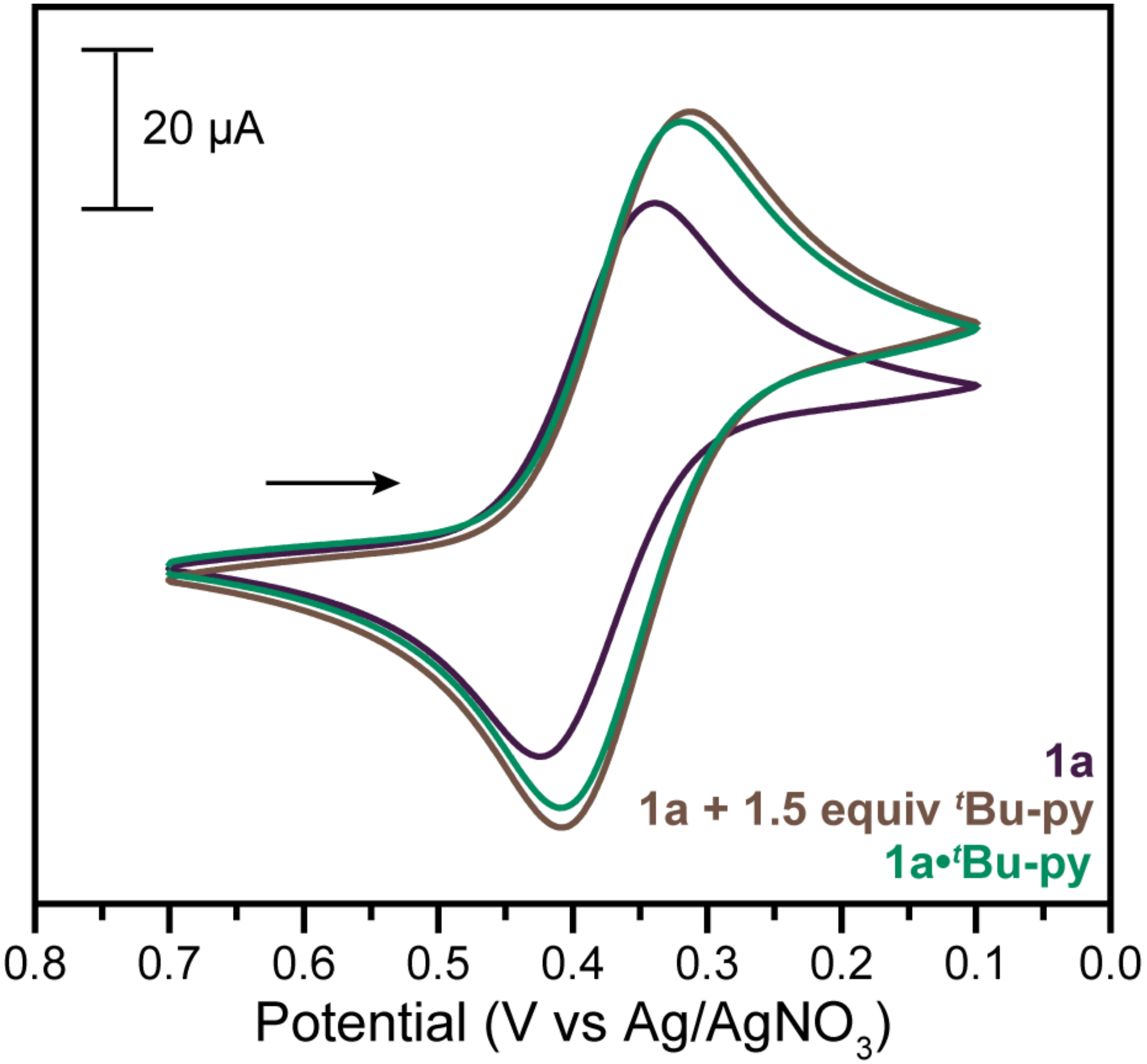

Figure S44. Comparison of cyclic voltammograms of 3 mM of **1a** in the absence and presence of 1.5 equiv of ^{t}Bu-py and 3 mM of **1a•^{t}Bu-py** in dry MeCN containing 0.1 M (^{n}Bu$_4$N)(PF$_6$) supporting electrolyte collected at the lowest temperature (~23–24 °C) of the variable-temperature cyclic voltammetry experiments (Figures 4, S37, and S42) in the potential window containing the Cu^{II}/Cu^{I} redox couple using 100 mV s^{-1} scan rate. Note that the E_{OCP} changes between the sample solutions, thus, no vertical line denoting E_{OCP} is displayed. The black arrow indicates the scan direction. Glassy carbon, Ag/AgNO$_3$, and Pt mesh were used as working, reference, and counter electrodes, respectively.

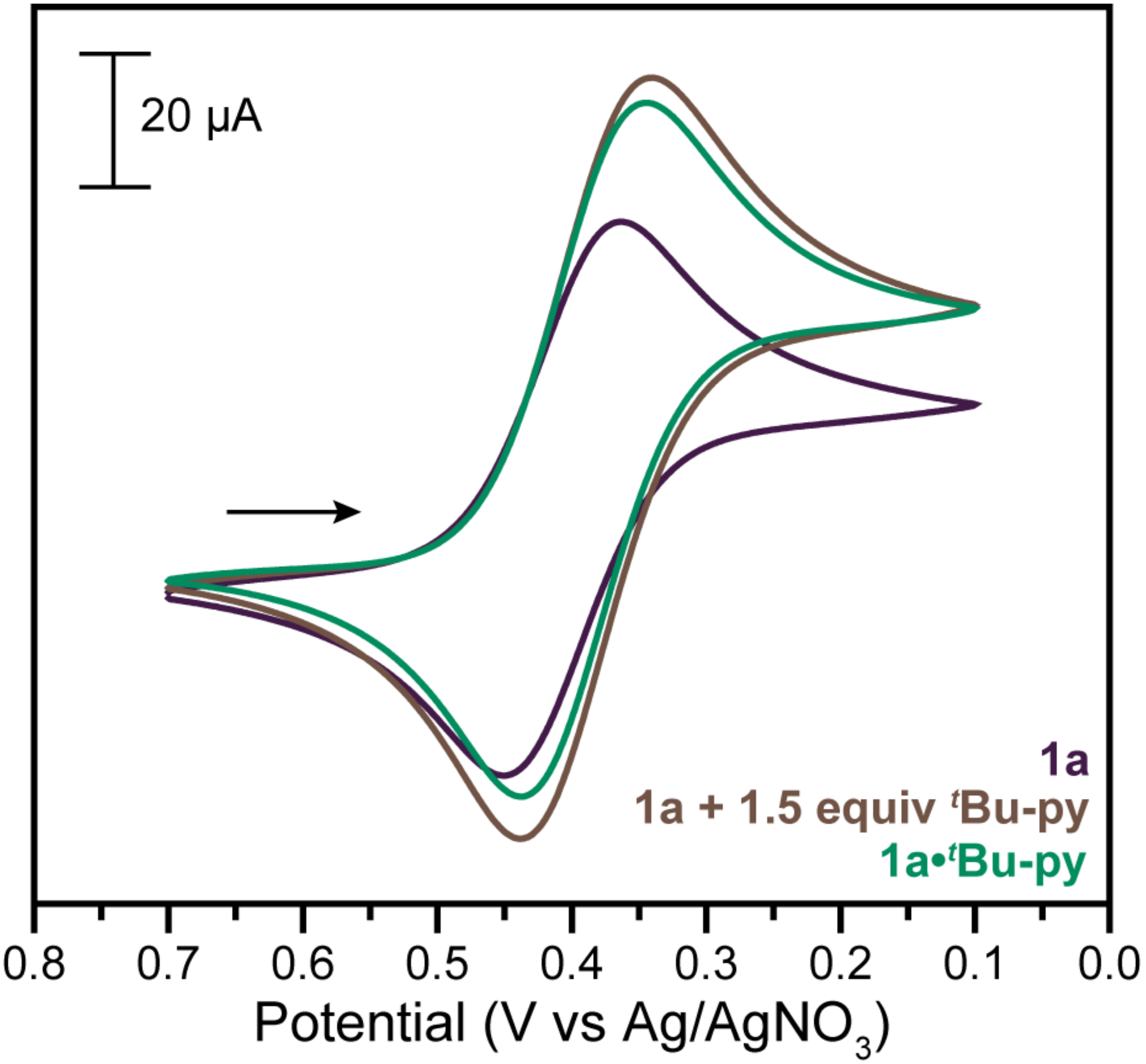

Figure S45. Comparison of cyclic voltammograms of 3 mM of **1a** in the absence and presence of 1.5 equiv of *t*Bu-py and 3 mM of **1a•*t*Bu-py** in dry MeCN containing 0.1 M ($^{n}Bu_4N$)(PF_6) supporting electrolyte collected at the highest temperature (~42–44 °C) of the variable-temperature cyclic voltammetry experiments (Figures 4, S37, and S42) in the potential window containing the Cu^{II}/Cu^{I} redox couple using 100 mV s^{-1} scan rate. Note that the E_{OCP} changes between the sample solutions, thus, no vertical line denoting E_{OCP} is displayed. The black arrow indicates the scan direction. Glassy carbon, $Ag/AgNO_3$, and Pt mesh were used as working, reference, and counter electrodes, respectively.

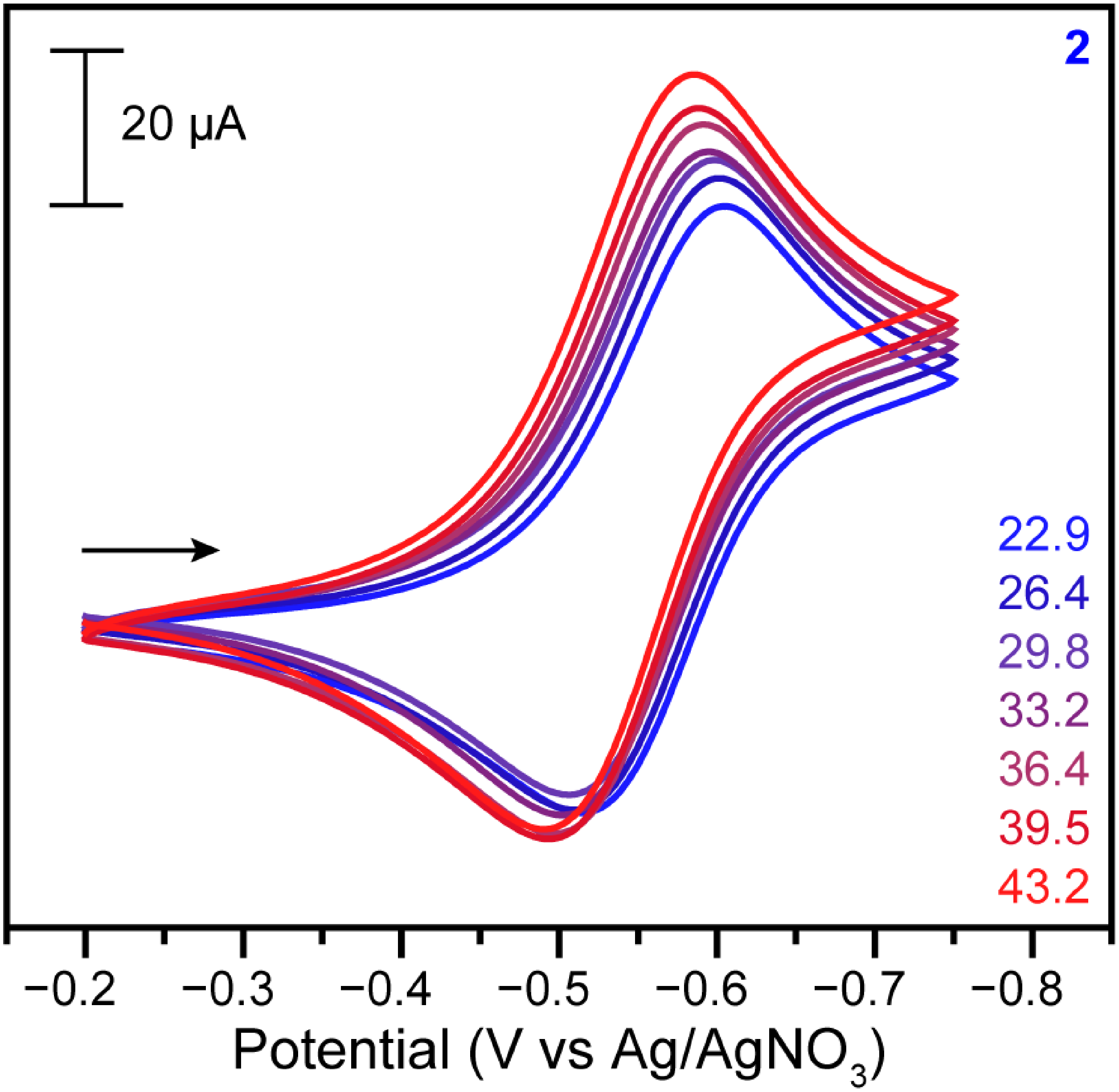

Figure S46. Cyclic voltammograms of 3 mM of **2** in dry MeCN containing 0.1 M ($^{n}Bu_4N$)(PF_6) supporting electrolyte collected at variable temperatures (~23–43 °C) in the potential window containing the Cu^{II}/Cu^{I} redox couple using 100 mV s^{-1} scan rate. Colored numbers denote the temperature of the solution for each measurement in °C. Note that the E_{OCP} changes with temperature, thus, no vertical line denoting E_{OCP} is displayed. The black arrow indicates the scan direction. Glassy carbon, Ag/$AgNO_3$, and Pt mesh were used as working, reference, and counter electrodes, respectively.

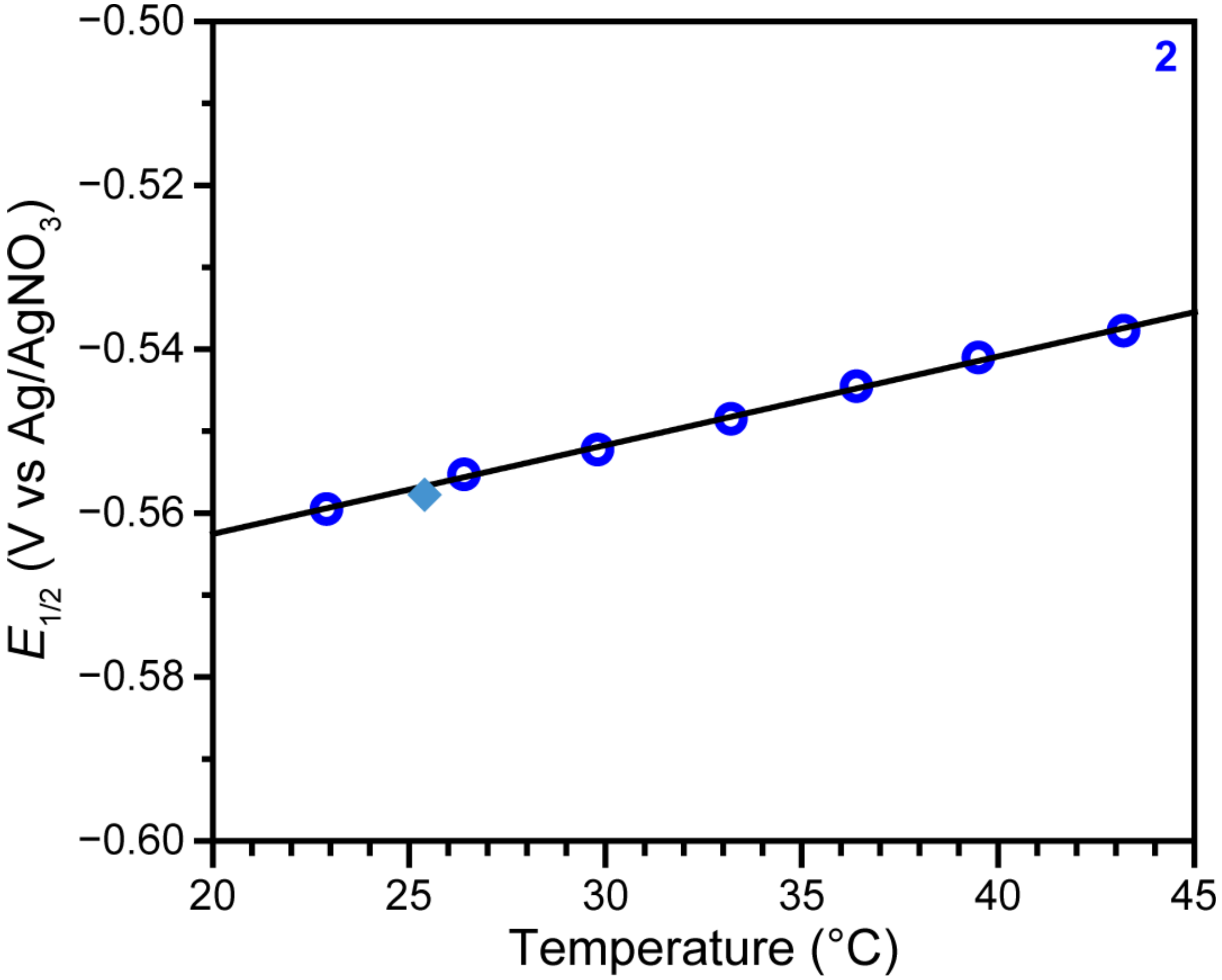

Figure S47. Plot of the temperature dependence of $E_{1/2}$ for the Cu^{II}/Cu^{I} redox couple of **2** in dry MeCN containing 0.1 M ($^{n}Bu_4N$)(PF_6) supporting electrolyte, as obtained from the variable-temperature cyclic voltammetry data shown in Figure S46. Colored circles denote experimental data from the variable-temperature experiment in heating mode, and the black line corresponds to a linear fit to the data. The average slope from four independent measurements after correcting for the temperature coefficient of the reference electrode potential (eqs S9 and S10), which represents the average temperature coefficient, is α = 1.66(7) mV °C^{-1} (Table 1). The error in the average slope corresponds to the standard deviation of individual measurements. The blue diamond corresponds to a data point measured after cooling the solution back down to close to room temperature.

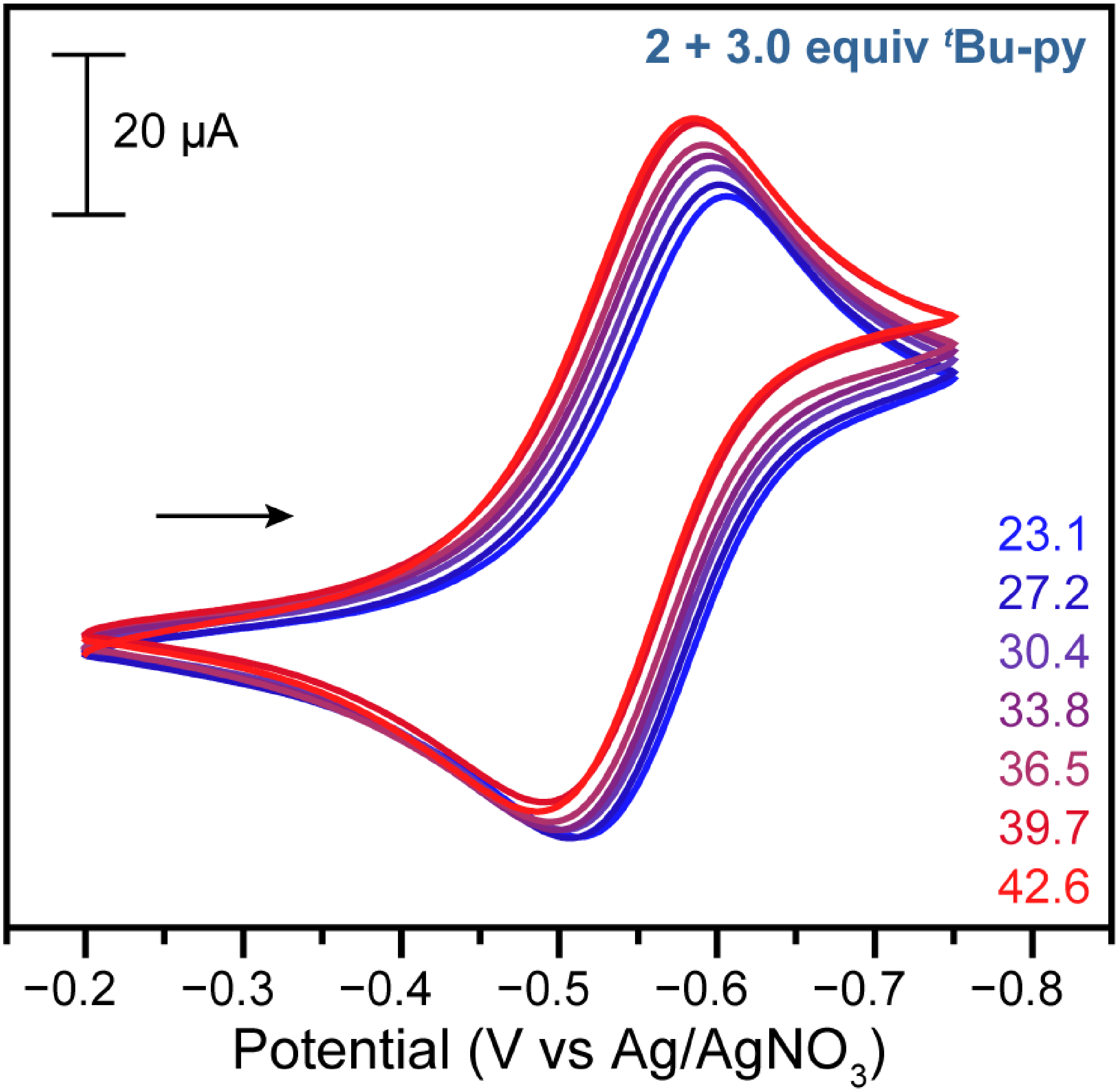

Figure S48. Cyclic voltammograms of 3 mM of **2** in the presence of 3.0 equiv of ^{t}Bu-py in dry MeCN containing 0.1 M (^{n}Bu$_4$N)(PF$_6$) supporting electrolyte collected at variable temperatures (~23–43 °C) in the potential window containing the Cu^{II}/Cu^{I} redox couple using 100 mV s^{-1} scan rate. Colored numbers denote the temperature of the solution for each measurement in °C. Note that the E_{OCP} changes with temperature, thus, no vertical line denoting E_{OCP} is displayed. The black arrow indicates the scan direction. Glassy carbon, Ag/AgNO$_3$, and Pt mesh were used as working, reference, and counter electrodes, respectively.

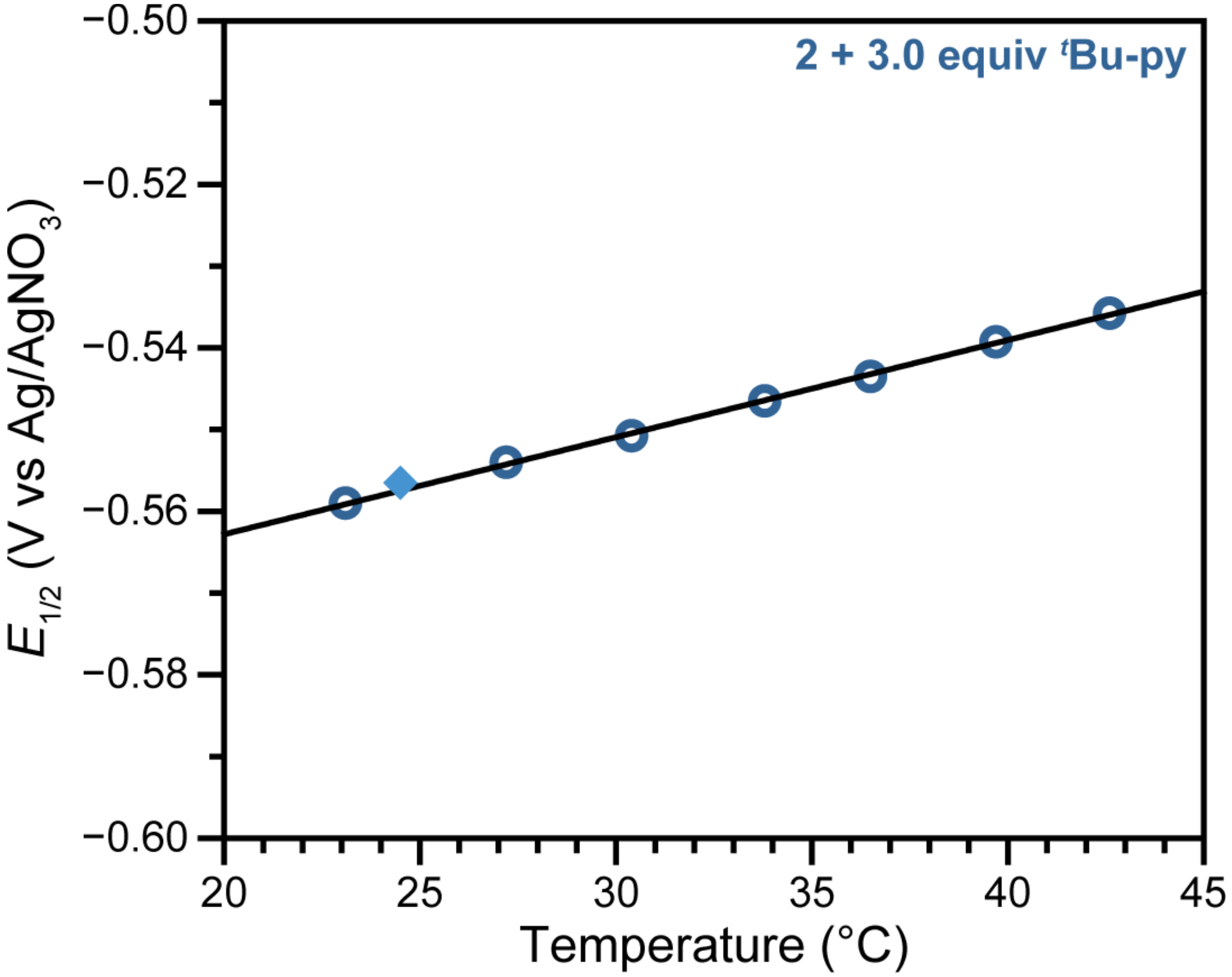

Figure S49. Plot of the temperature dependence of $E_{1/2}$ for the Cu^{II}/Cu^{I} redox couple of **2** in the presence of 3.0 equiv of ^{t}Bu-py in dry MeCN containing 0.1 M ($^{n}Bu_4N$)(PF_6) supporting electrolyte, as obtained from the variable-temperature cyclic voltammetry data shown in Figure S48. Colored circles denote experimental data from the variable-temperature experiment in heating mode, and the black line corresponds to a linear fit to the data. The average slope from three independent measurements after correcting for the temperature coefficient of the reference electrode potential (eqs S9 and S10), which represents the average temperature coefficient, is α = 1.67(6) mV °C^{-1} (Table 1). The error in the average slope corresponds to the standard deviation of individual measurements. The blue diamond corresponds to a data point measured after cooling the solution back down to close to room temperature.

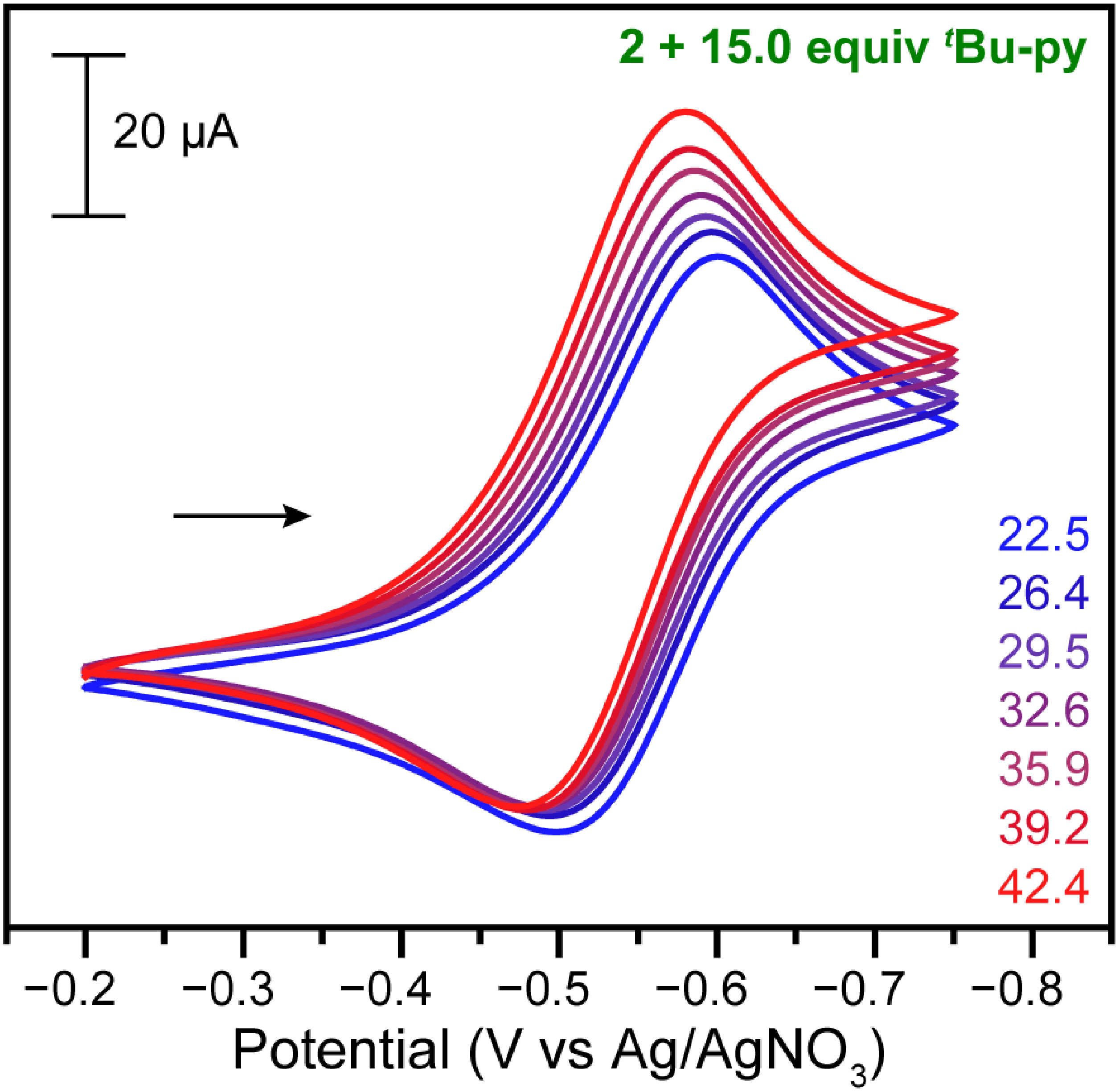

Figure S50. Cyclic voltammograms of 3 mM of **2** in the presence of 15.0 equiv of ^{t}Bu-py in dry MeCN containing 0.1 M (${}^{n}Bu_4N$)(PF_6) supporting electrolyte collected at variable temperatures (~23–42 °C) in the potential window containing the Cu^{II}/Cu^{I} redox couple using 100 mV s^{-1} scan rate. Colored numbers denote the temperature of the solution for each measurement in °C. Note that the E_{OCP} changes with temperature, thus, no vertical line denoting E_{OCP} is displayed. The black arrow indicates the scan direction. Glassy carbon, Ag/$AgNO_3$, and Pt mesh were used as working, reference, and counter electrodes, respectively.

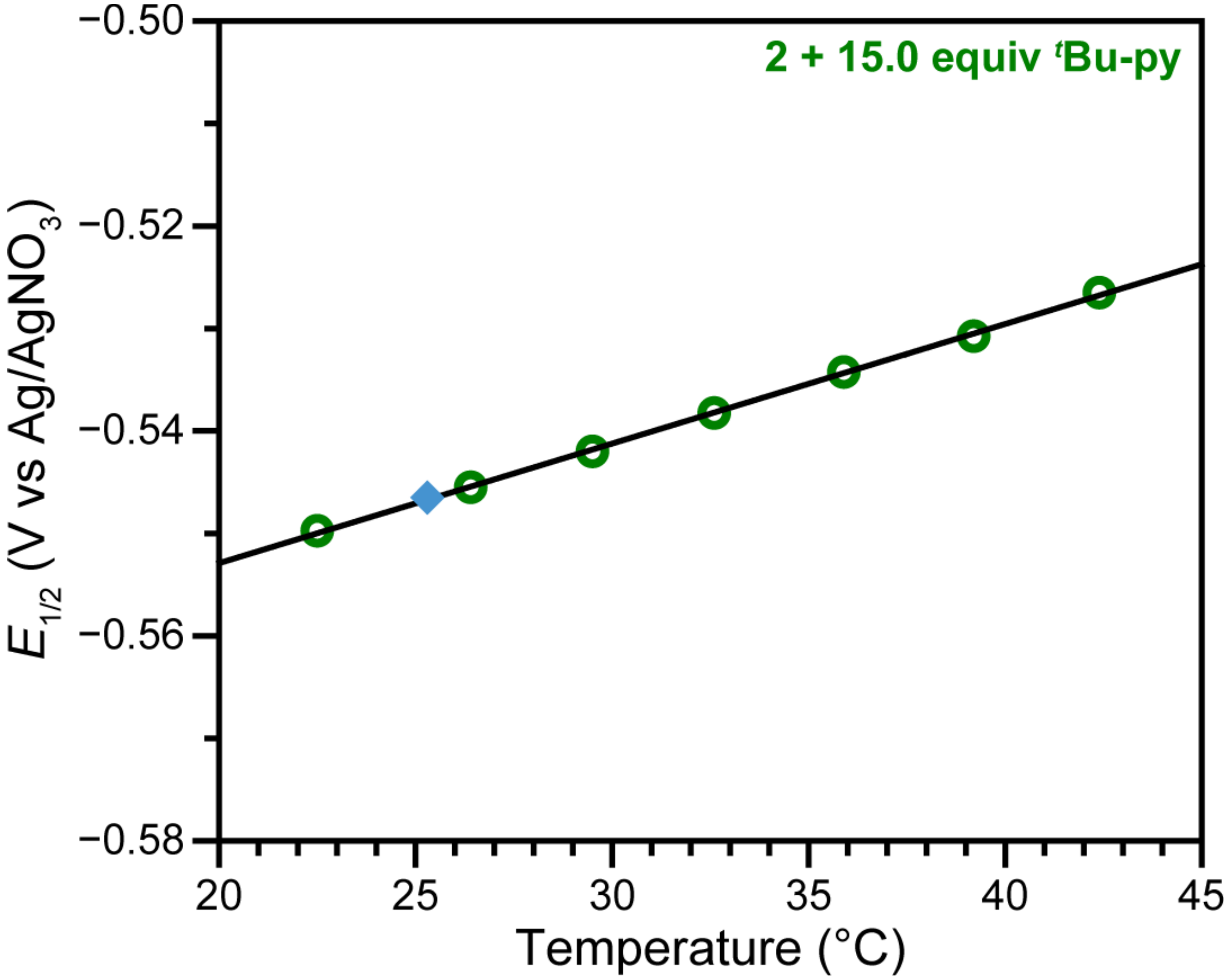

Figure S51. Plot of the temperature dependence of $E_{1/2}$ for the Cu^{II}/Cu^{I} redox couple of **2** in the presence of 15.0 equiv of ^{t}Bu-py in dry MeCN containing 0.1 M ($^{n}Bu_4N$)(PF_6) supporting electrolyte, as obtained from the variable-temperature cyclic voltammetry data shown in Figure S50. Colored circles denote experimental data from the variable-temperature experiment in heating mode, and the black line corresponds to a linear fit to the data. The average slope from three independent measurements after correcting for the temperature coefficient of the reference electrode potential (eqs S9 and S10), which represents the average temperature coefficient, is α = 1.6(1) mV °C^{-1} (Table 1). The error in the average slope corresponds to the standard deviation of individual measurements. The blue diamond corresponds to a data point measured after cooling the solution back down to close to room temperature.

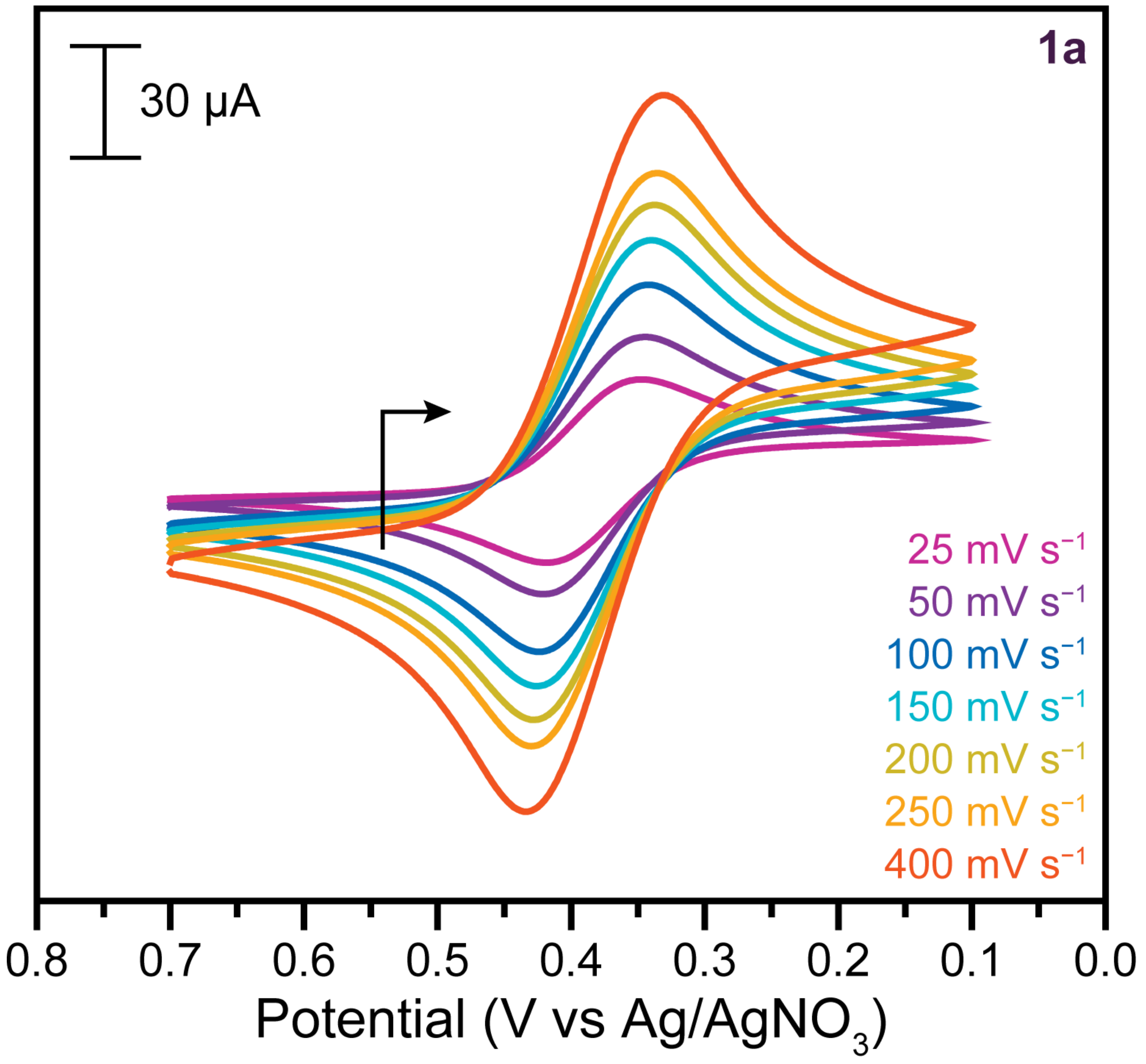

Figure S52. Cyclic voltammograms of 3.0 mM of **1a** in dry MeCN containing 0.1 M (^{n}Bu$_4$N)(PF$_6$) supporting electrolyte collected at room temperature (23–25 °C) in the potential window containing the Cu^{II}/Cu^{I} redox couple using variable scan rates (25–400 mV s^{-1}). Colored numbers denote the scan rate used for each measurement. The vertical black line and arrow denote the open-circuit potential and scan direction, respectively. Glassy carbon, Ag/AgNO$_3$, and Pt mesh were used as working, reference, and counter electrodes, respectively.

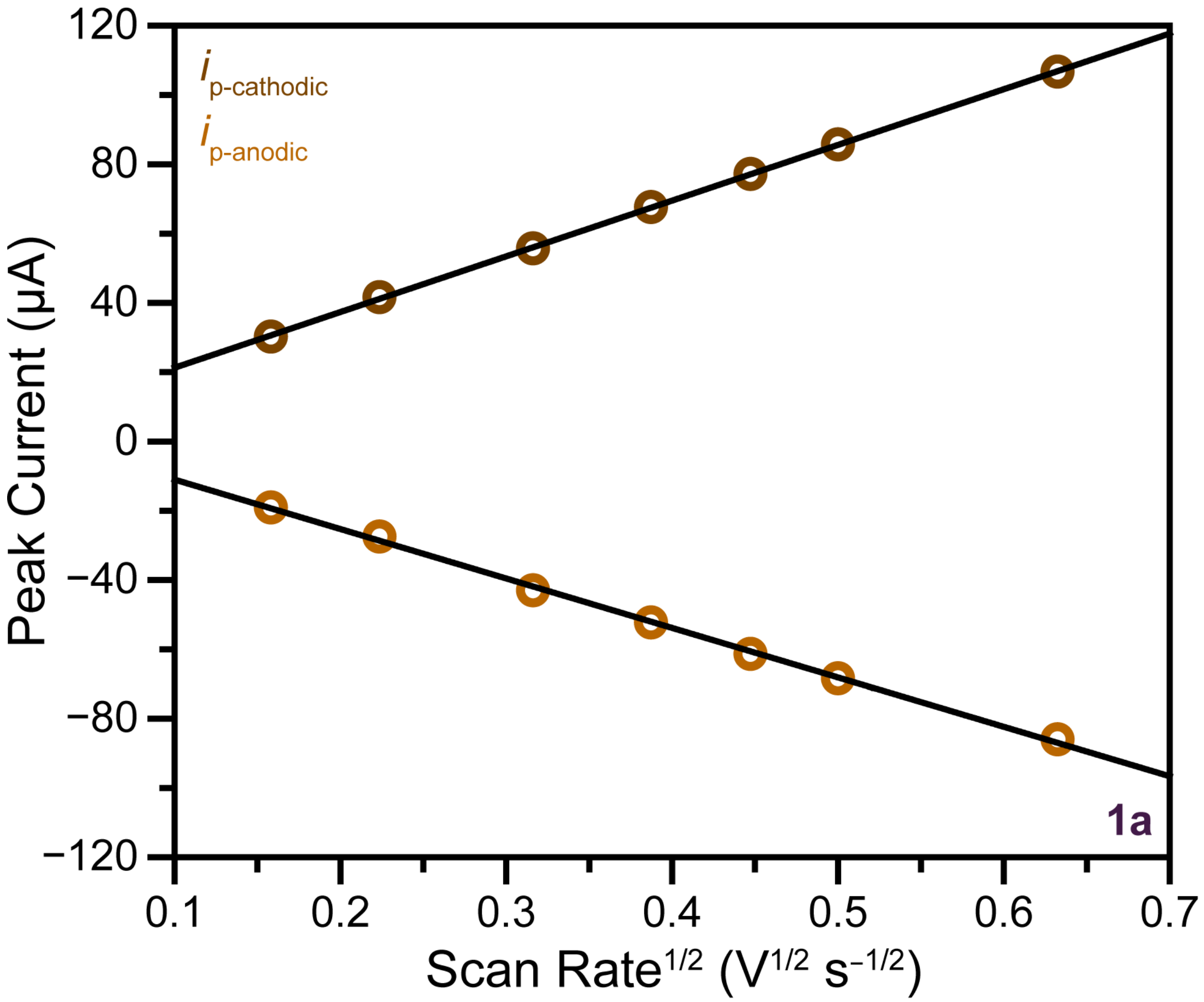

Figure S53. Randles–Ševčík plot for the Cu^{II}/Cu^{I} redox couple of **1a** obtained from the variable-scan-rate cyclic voltammetry data shown in Figure S52. Colored circles denote experimental data, and black lines correspond to linear fits to the data. $i_{p\text{-cathodic}}$ and $i_{p\text{-anodic}}$ denote the cathodic and anodic peak current, respectively.

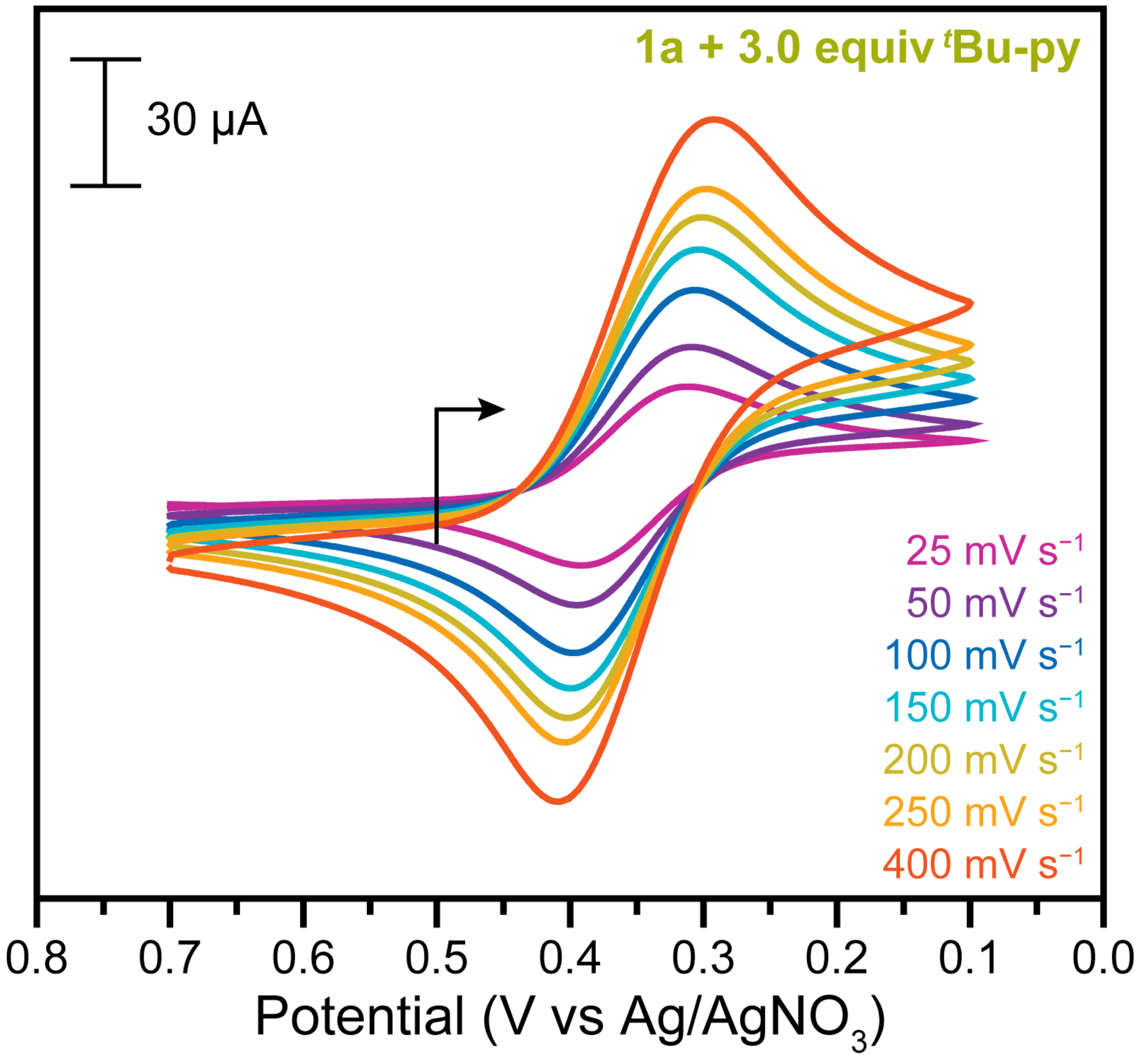

Figure S54. Cyclic voltammograms of 3.0 mM of **1a** in the presence of 3.0 equiv of *t*Bu-py in dry MeCN containing 0.1 M (^{n}Bu$_4$N)(PF$_6$) supporting electrolyte collected at room temperature (23–25 °C) in the potential window containing the Cu^{II}/Cu^{I} redox couple using variable scan rates (25–400 mV s^{-1}). Colored numbers denote the scan rate used for each measurement. The vertical black line and arrow denote the open-circuit potential and scan direction, respectively. Glassy carbon, Ag/AgNO$_3$, and Pt mesh were used as working, reference, and counter electrodes, respectively.

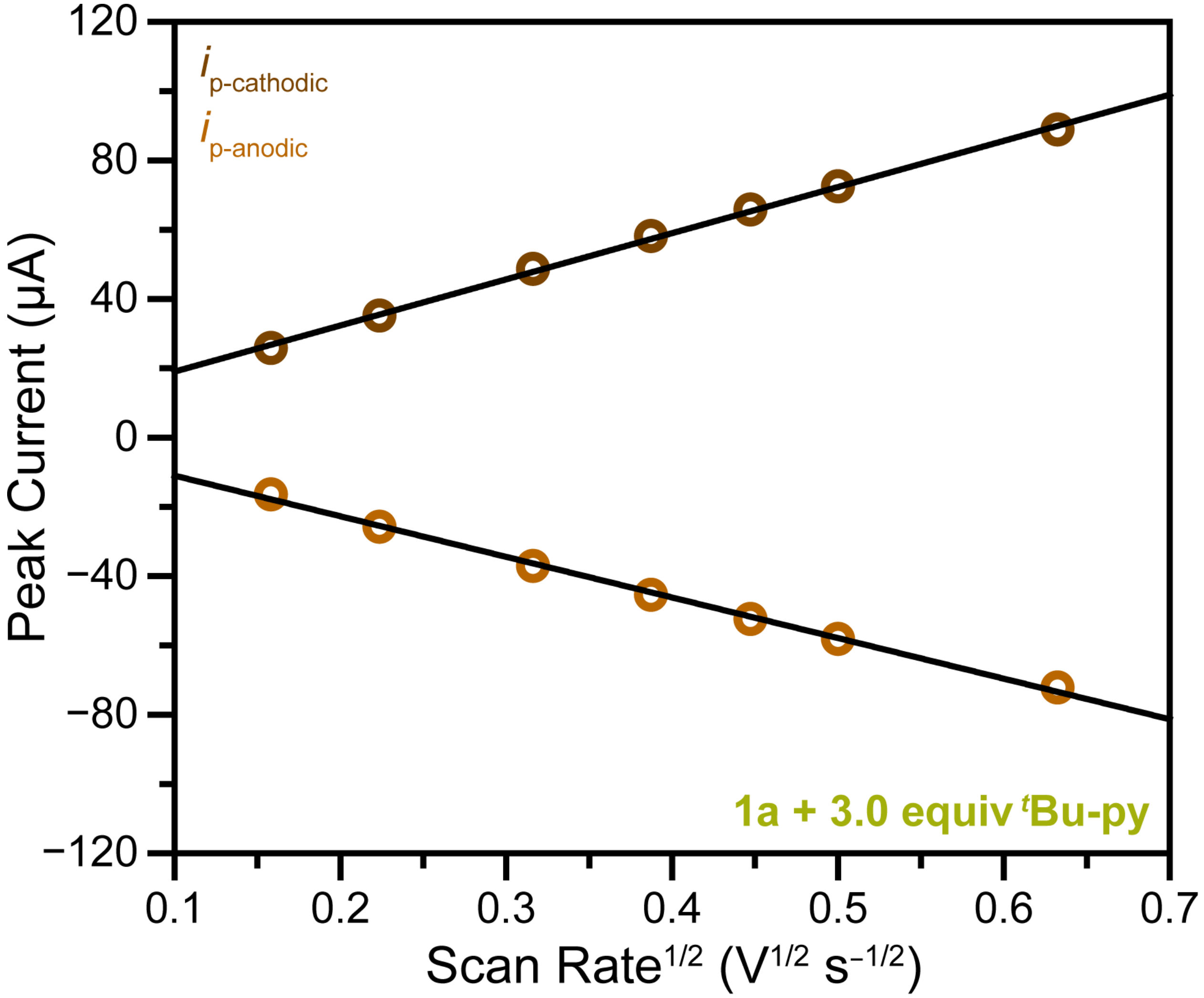

Figure S55. Randles–Ševčík plot for the Cu^{II}/Cu^{I} redox couple of **1a** in the presence of 3.0 equiv of ^{t}Bu-py obtained from the variable-scan-rate cyclic voltammetry data shown in Figure S54. Colored circles denote experimental data, and black lines correspond to linear fits to the data. $i_{p\text{-cathodic}}$ and $i_{p\text{-anodic}}$ denote the cathodic and anodic peak current, respectively.

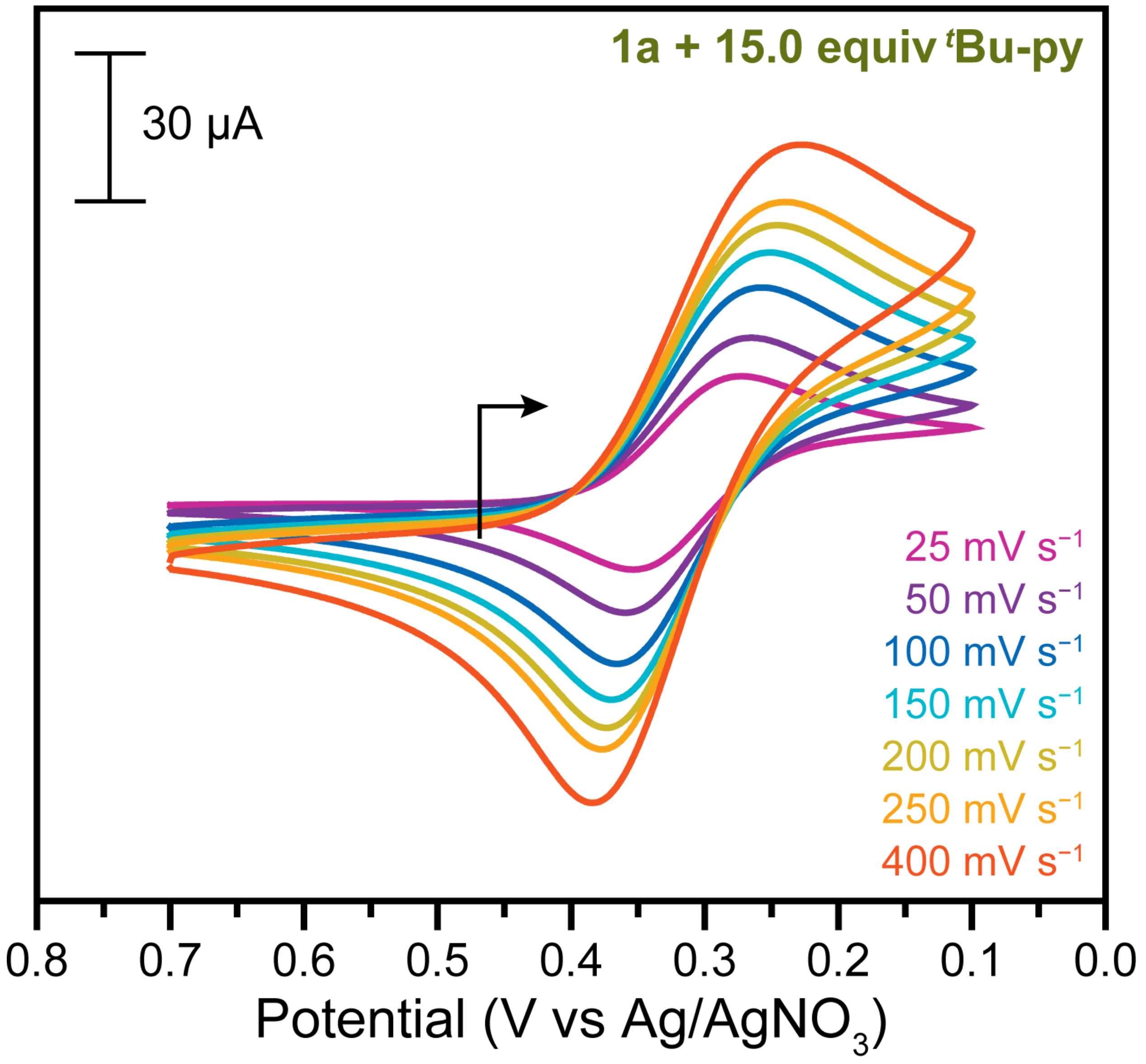

Figure S56. Cyclic voltammograms of 3.0 mM of **1a** in the presence of 15.0 equiv of ^{t}Bu-py in dry MeCN containing 0.1 M (^{n}Bu$_4$N)(PF$_6$) supporting electrolyte collected at room temperature (23–25 °C) in the potential window containing the Cu^{II}/Cu^{I} redox couple using variable scan rates (25–400 mV s^{-1}). Colored numbers denote the scan rate used for each measurement. The vertical black line and arrow denote the open-circuit potential and scan direction, respectively. Glassy carbon, Ag/AgNO$_3$, and Pt mesh were used as working, reference, and counter electrodes, respectively.

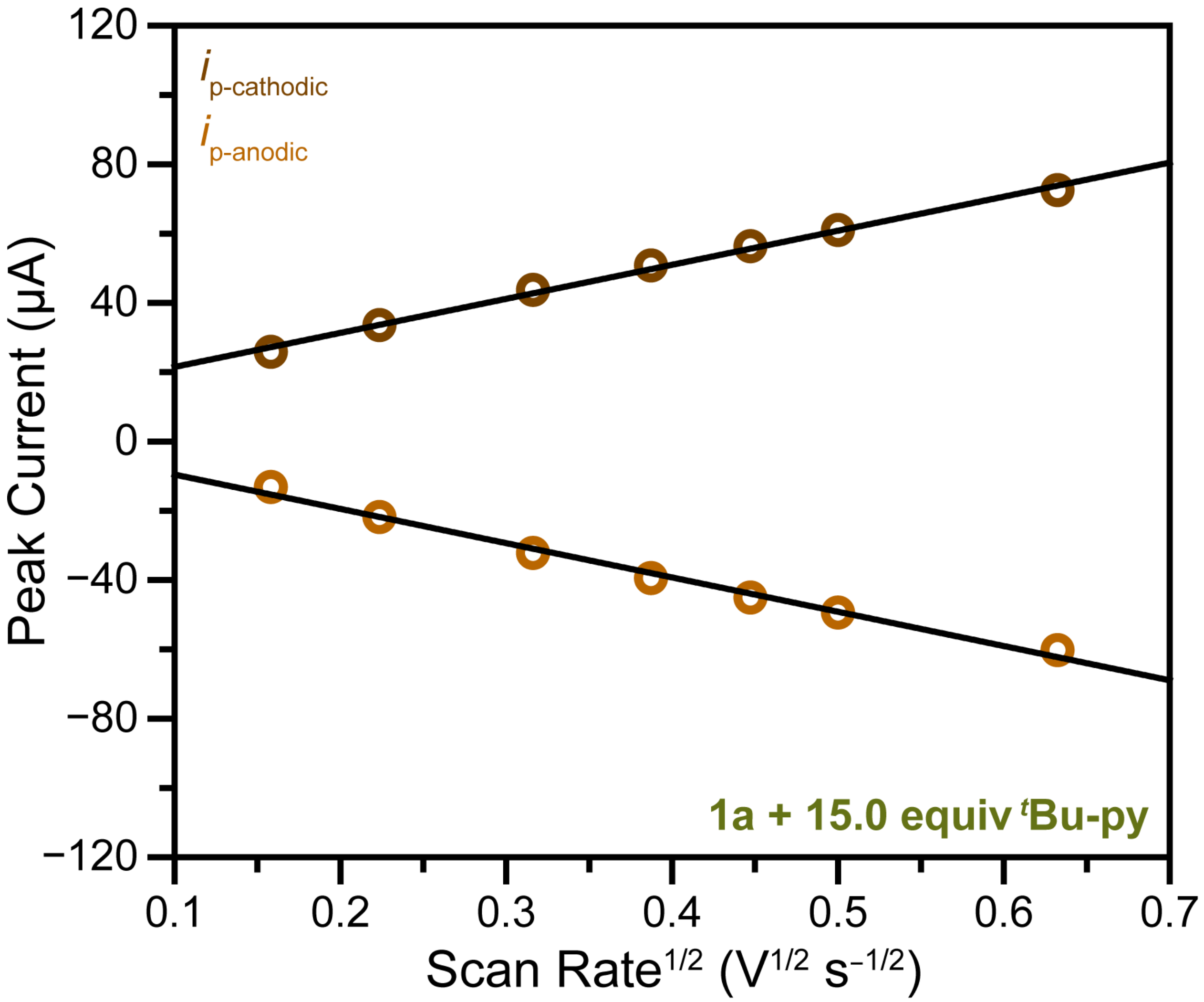

Figure S57. Randles–Ševčík plot for the Cu^{II}/Cu^{I} redox couple of **1a** in the presence of 15.0 equiv of ^{t}Bu-py obtained from the variable-scan-rate cyclic voltammetry data shown in Figure S56. Colored circles denote experimental data, and black lines correspond to linear fits to the data. $i_{p\text{-cathodic}}$ and $i_{p\text{-anodic}}$ denote the cathodic and anodic peak current, respectively.

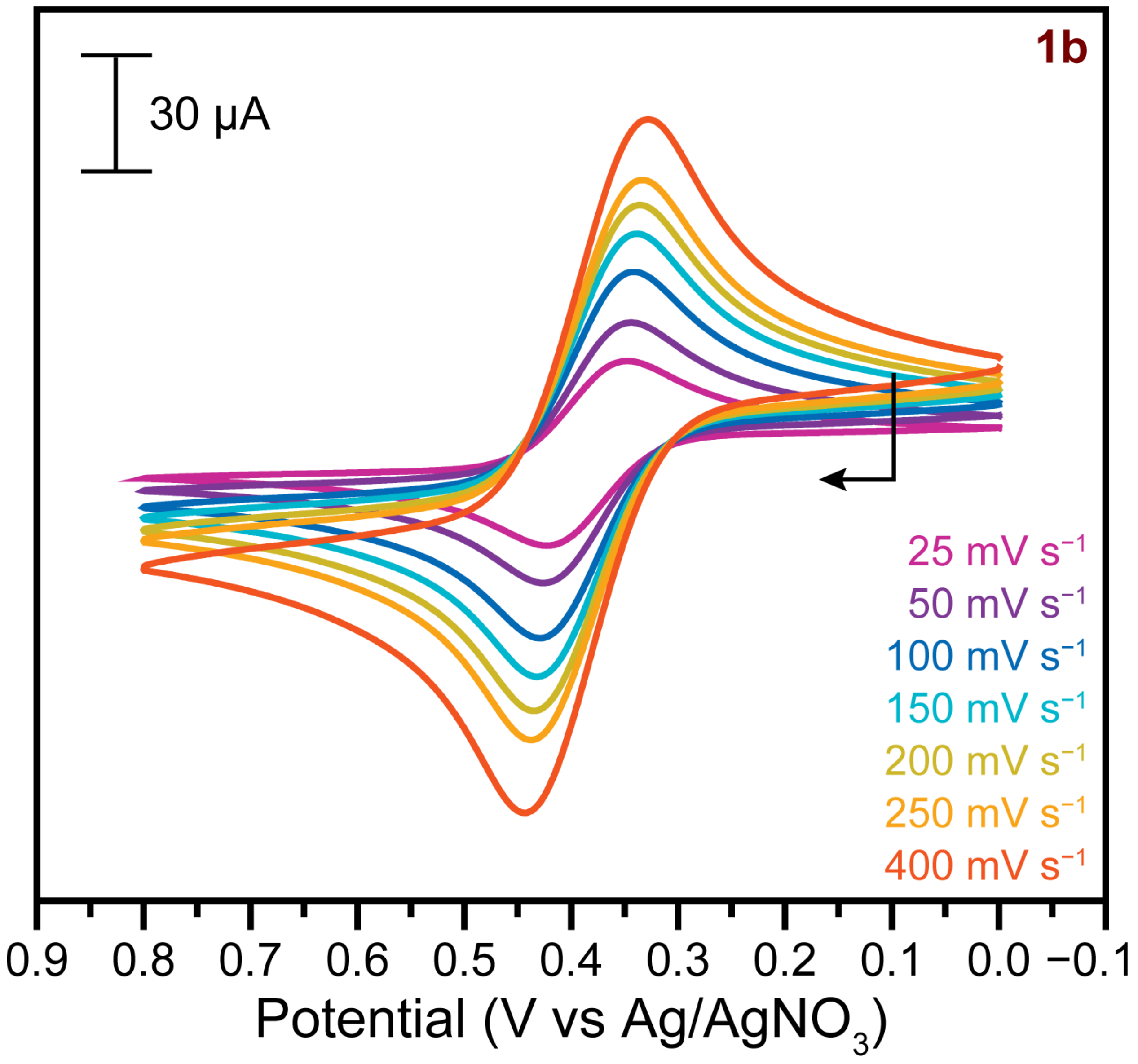

Figure S58. Cyclic voltammograms of 3.0 mM of **1b** in dry MeCN containing 0.1 M (^{n}Bu$_4$N)(PF$_6$) supporting electrolyte collected at room temperature (23–25 °C) in the potential window containing the Cu^{II}/Cu^{I} redox couple using variable scan rates (25–400 mV s^{-1}). Colored numbers denote the scan rate used for each measurement. The vertical black line and arrow denote the open-circuit potential and scan direction, respectively. Glassy carbon, Ag/AgNO$_3$, and Pt mesh were used as working, reference, and counter electrodes, respectively.

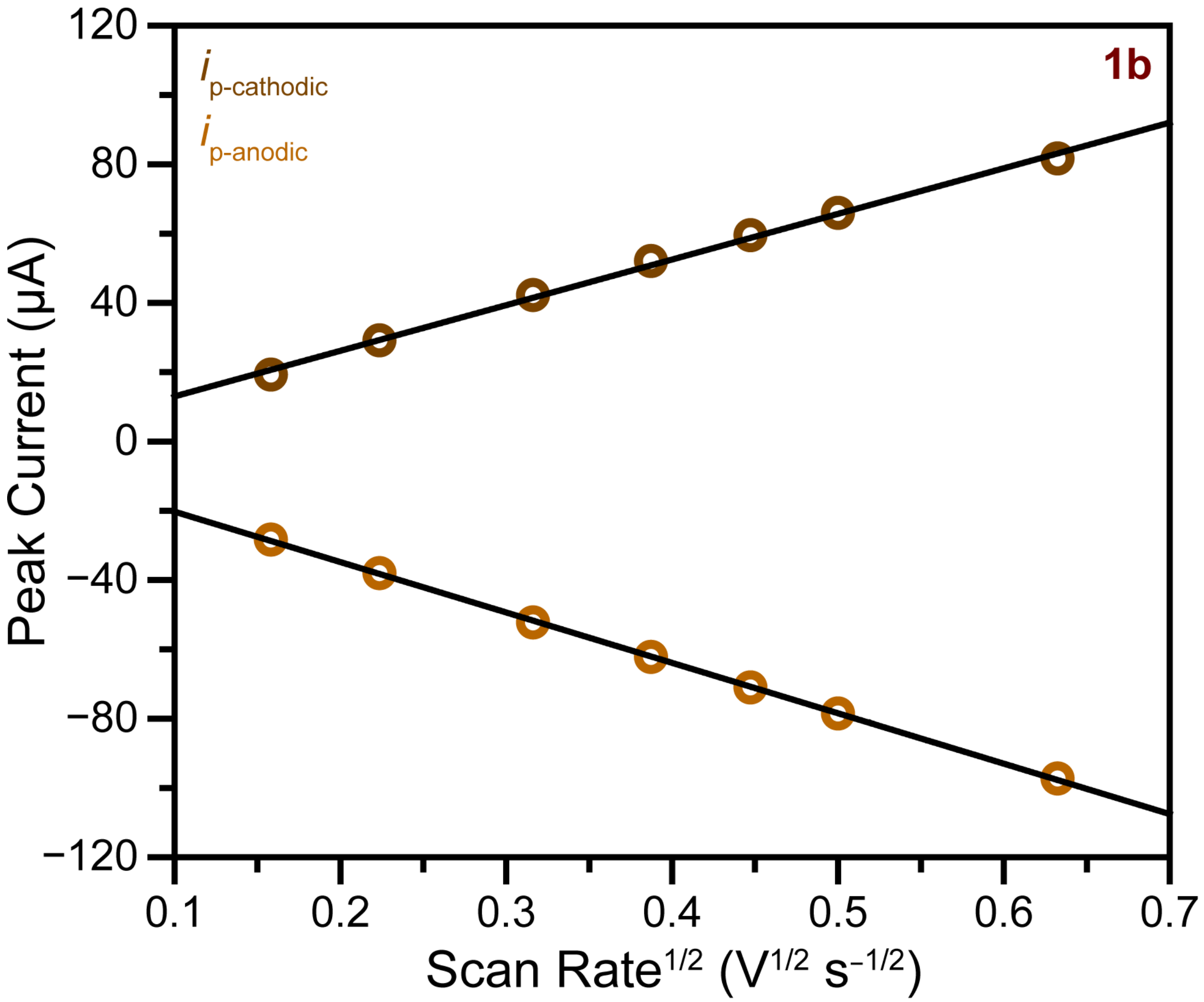

Figure S59. Randles–Ševčík plot for the Cu^{II}/Cu^{I} redox couple of **1b** obtained from the variable-scan-rate cyclic voltammetry data shown in Figure S58. Colored circles denote experimental data, and black lines correspond to linear fits to the data. $i_{p\text{-cathodic}}$ and $i_{p\text{-anodic}}$ denote the cathodic and anodic peak current, respectively.

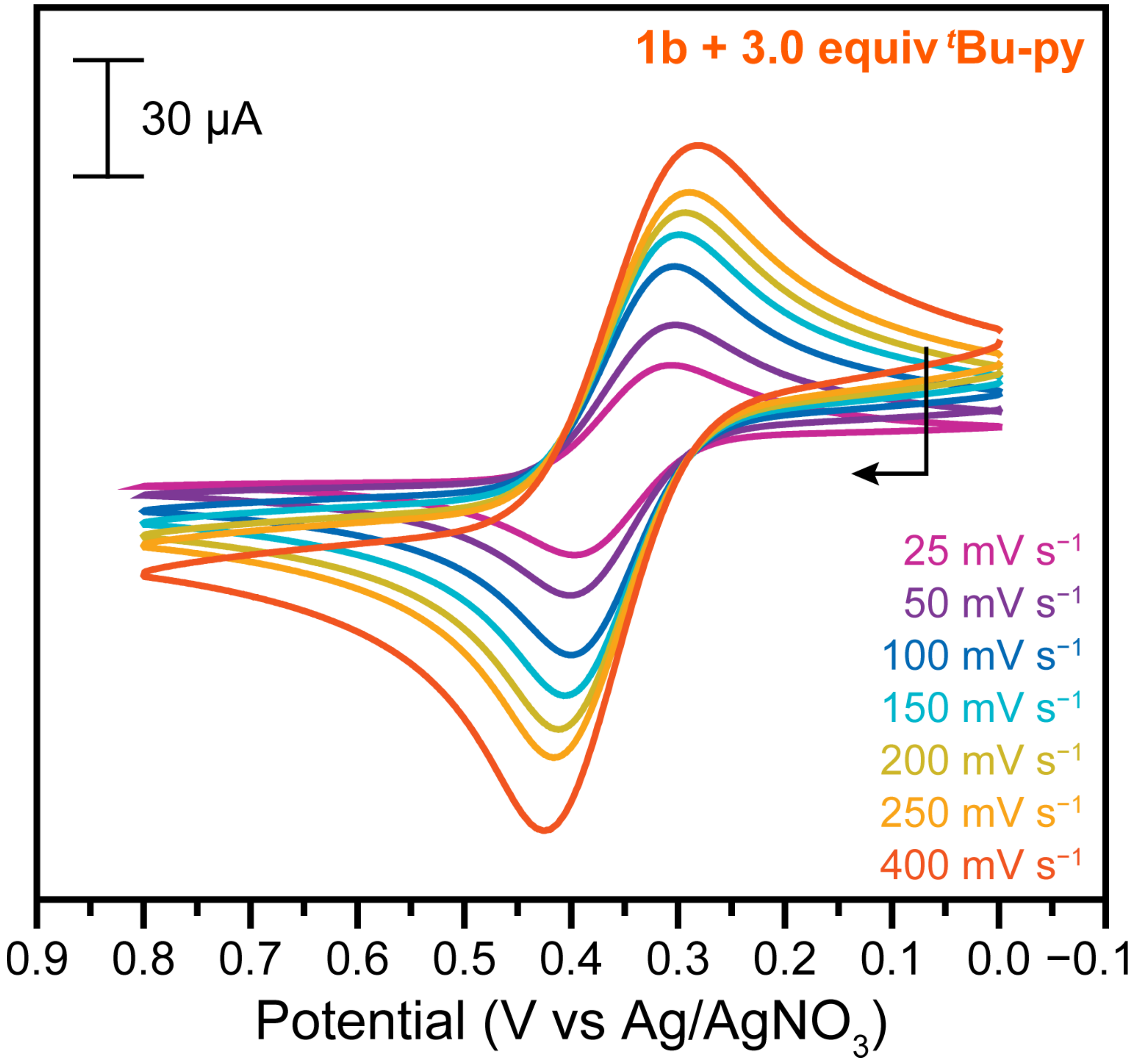

Figure S60. Cyclic voltammograms of 3.0 mM of **1b** in the presence of 3.0 equiv of ^{t}Bu-py in dry MeCN containing 0.1 M (^{n}Bu$_4$N)(PF$_6$) supporting electrolyte collected at room temperature (23–25 °C) in the potential window containing the Cu^{II}/Cu^{I} redox couple using variable scan rates (25–400 mV s^{-1}). Colored numbers denote the scan rate used for each measurement. The vertical black line and arrow denote the open-circuit potential and scan direction, respectively. Glassy carbon, Ag/AgNO$_3$, and Pt mesh were used as working, reference, and counter electrodes, respectively.

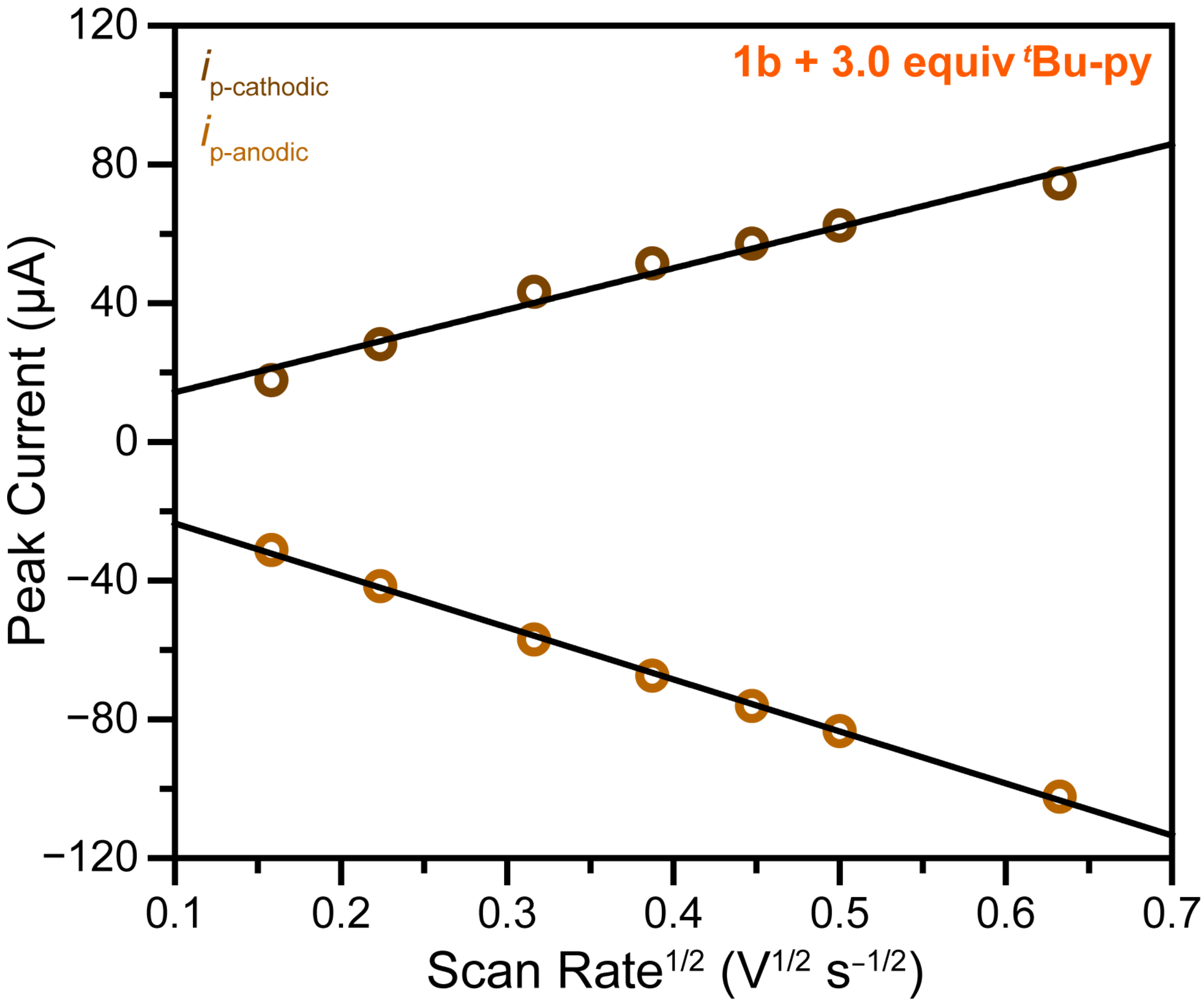

Figure S61. Randles–Ševčík plot for the Cu^{II}/Cu^{I} redox couple of **1b** in the presence of 3.0 equiv of ^{t}Bu-py obtained from the variable-scan-rate cyclic voltammetry data shown in Figure S60. Colored circles denote experimental data, and black lines correspond to linear fits to the data. $i_{p\text{-cathodic}}$ and $i_{p\text{-anodic}}$ denote the cathodic and anodic peak current, respectively.

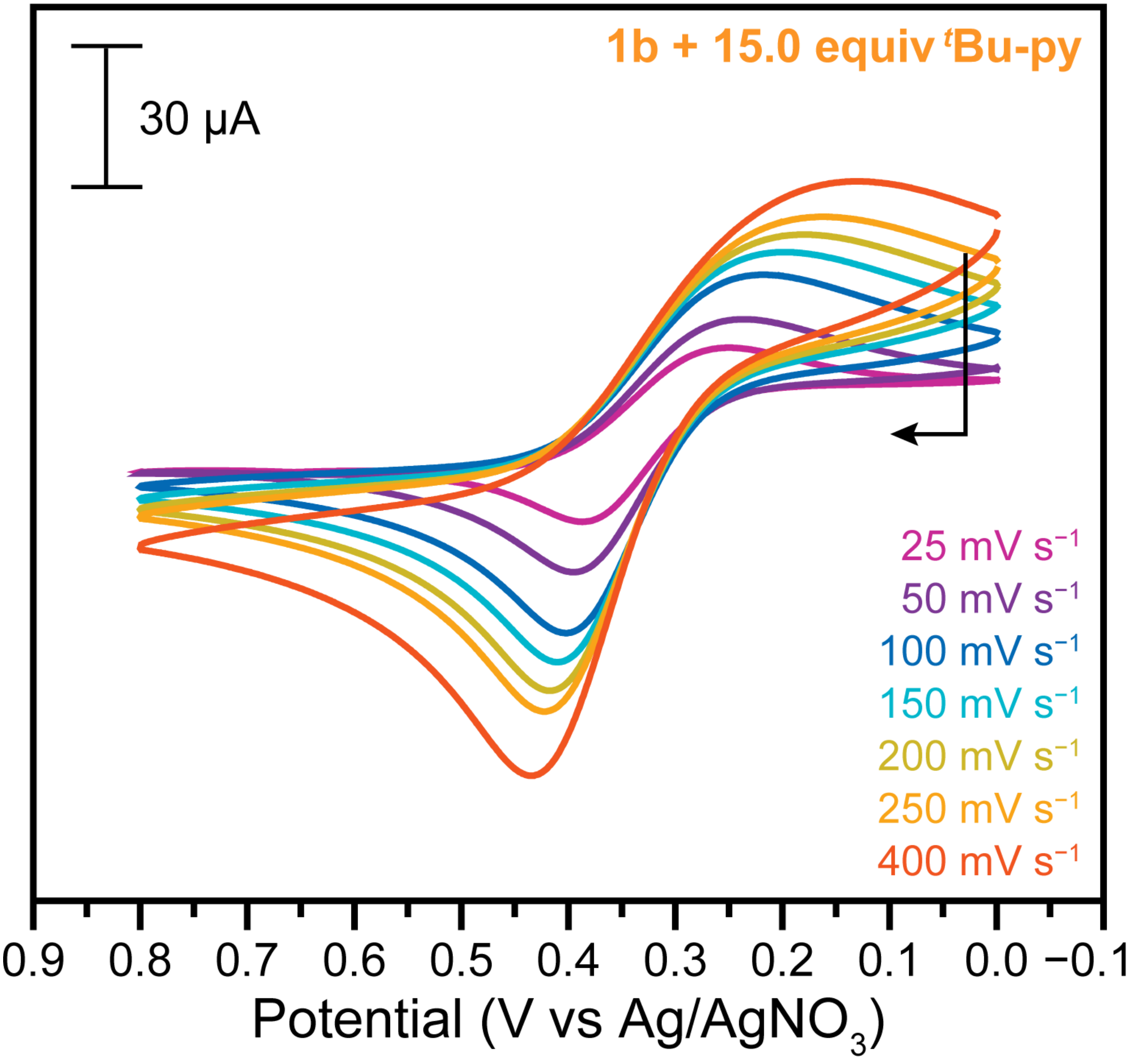

Figure S62. Cyclic voltammograms of 3.0 mM of **1b** in the presence of 15.0 equiv of ^{t}Bu-py in dry MeCN containing 0.1 M (^{n}Bu$_4$N)(PF$_6$) supporting electrolyte collected at room temperature (23–25 °C) in the potential window containing the Cu^{II}/Cu^{I} redox couple using variable scan rates (25–400 mV s^{-1}). Colored numbers denote the scan rate used for each measurement. The vertical black line and arrow denote the open-circuit potential and scan direction, respectively. Glassy carbon, Ag/AgNO$_3$, and Pt mesh were used as working, reference, and counter electrodes, respectively.

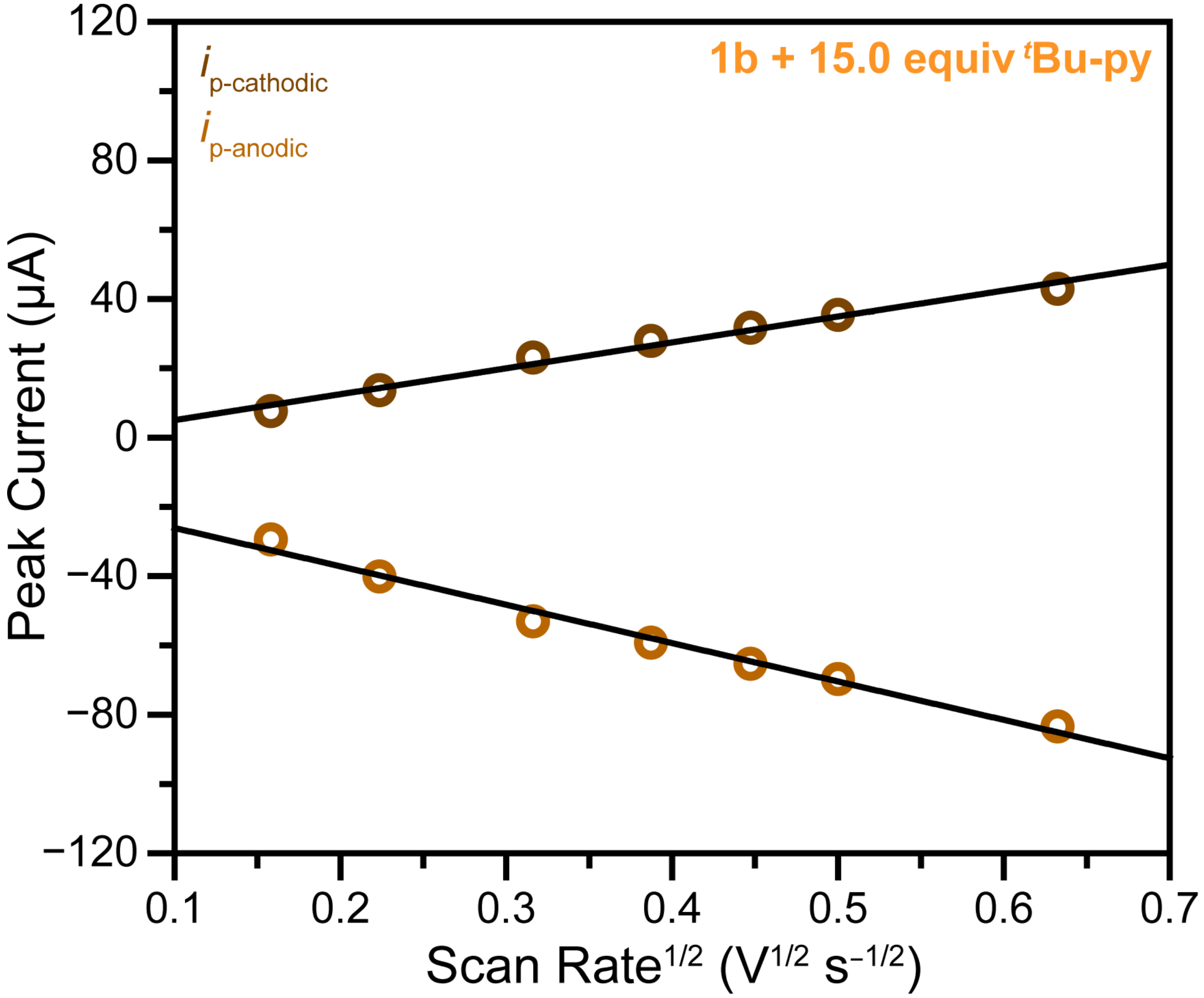

Figure S63. Randles–Ševčík plot for the Cu^{II}/Cu^{I} redox couple of **1b** in the presence of 15.0 equiv of ^{t}Bu-py obtained from the variable-scan-rate cyclic voltammetry data shown in Figure S62. Colored circles denote experimental data, and black lines correspond to linear fits to the data. $i_{\text{p-cathodic}}$ and $i_{\text{p-anodic}}$ denote the cathodic and anodic peak current, respectively.

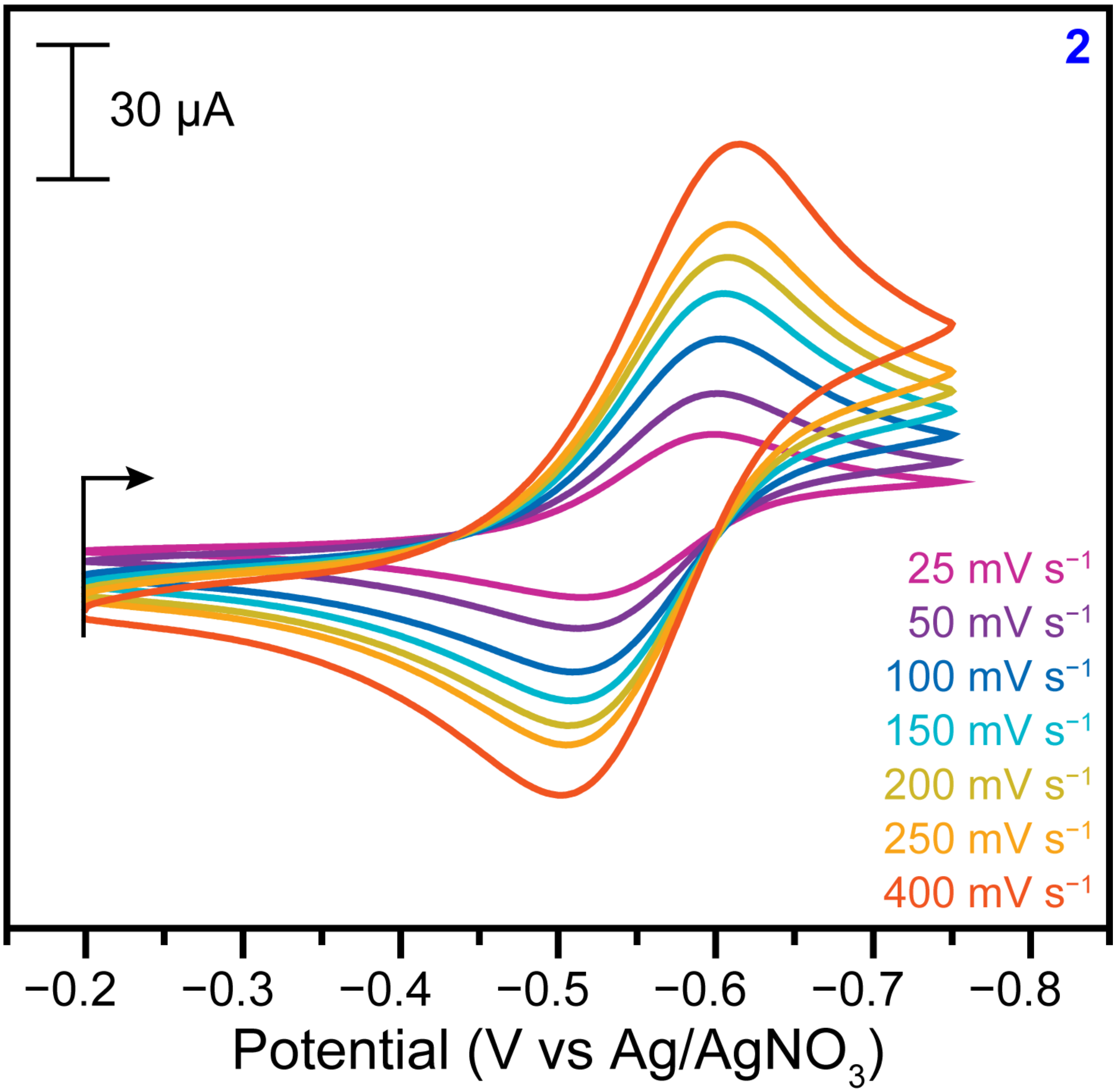

Figure S64. Cyclic voltammograms of 3.0 mM of **2** in dry MeCN containing 0.1 M ($^{n}Bu_4N$)(PF_6) supporting electrolyte collected at room temperature (23–25 °C) in the potential window containing the Cu^{II}/Cu^{I} redox couple using variable scan rates (25–400 mV s^{-1}). Colored numbers denote the scan rate used for each measurement. The vertical black line and arrow denote the open-circuit potential and scan direction, respectively. Glassy carbon, $Ag/AgNO_3$, and Pt mesh were used as working, reference, and counter electrodes, respectively.

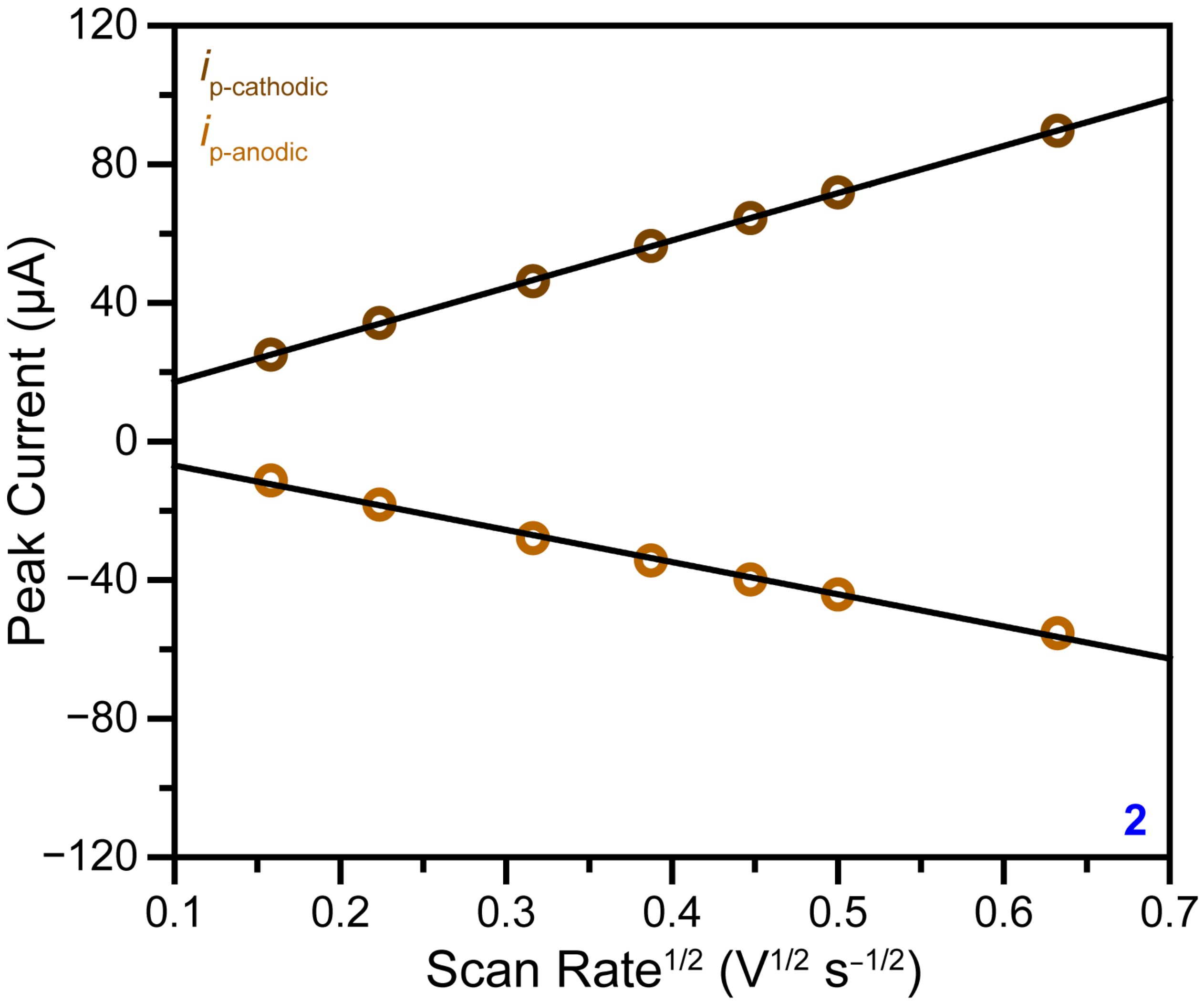

Figure S65. Randles–Ševčík plot for the Cu^{II}/Cu^{I} redox couple of **2** obtained from the variable-scan-rate cyclic voltammetry data shown in Figure S64. Colored circles denote experimental data, and black lines correspond to linear fits to the data. $i_{\text{p-cathodic}}$ and $i_{\text{p-anodic}}$ denote the cathodic and anodic peak current, respectively.

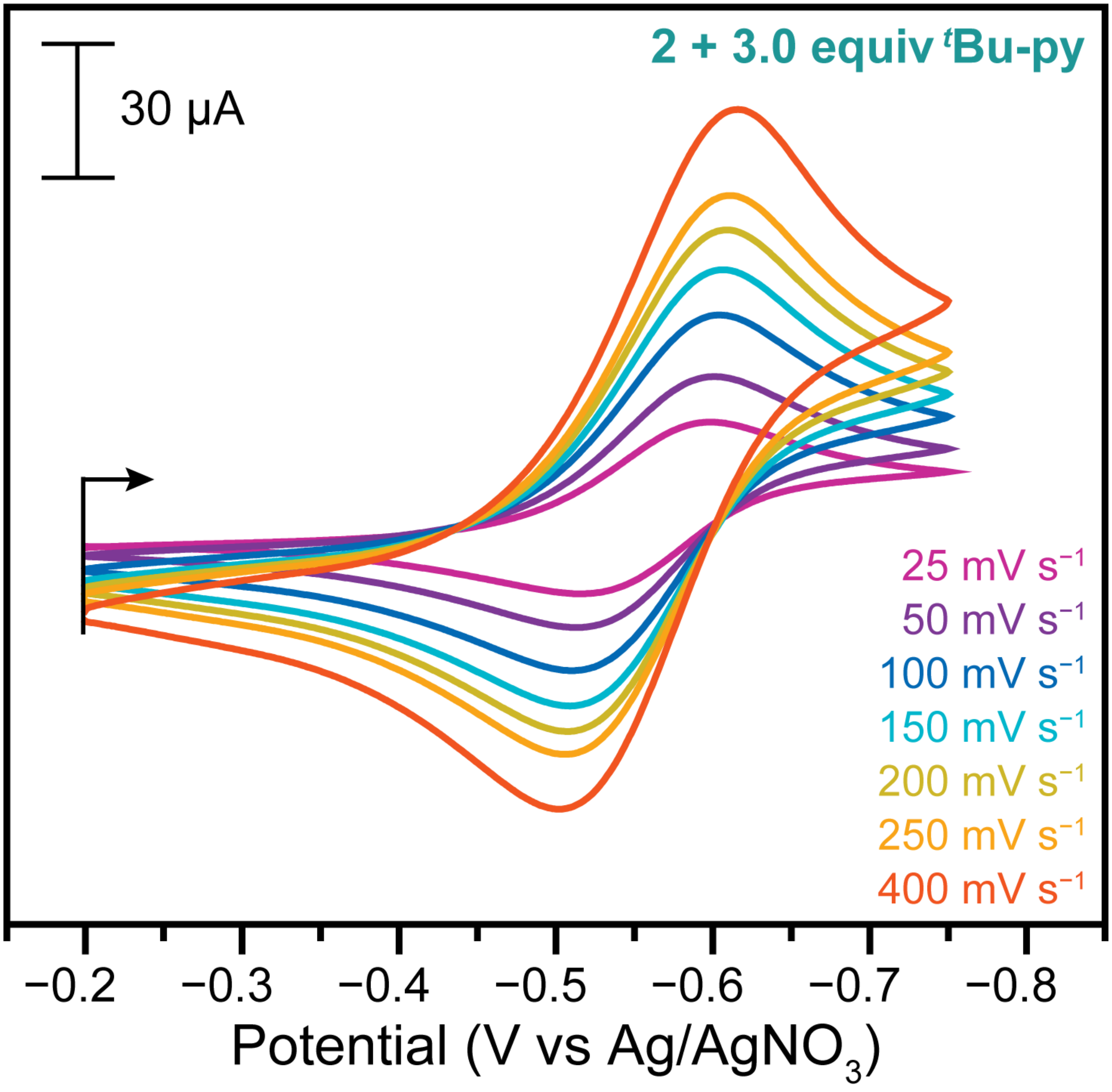

Figure S66. Cyclic voltammograms of 3.0 mM of **2** in the presence of 3.0 equiv of ^{t}Bu-py in dry MeCN containing 0.1 M (^{n}Bu$_4$N)(PF$_6$) supporting electrolyte collected at room temperature (23–25 °C) in the potential window containing the Cu^{II}/Cu^{I} redox couple using variable scan rates (25–400 mV s^{-1}). Colored numbers denote the scan rate used for each measurement. The vertical black line and arrow denote the open-circuit potential and scan direction, respectively. Glassy carbon, Ag/AgNO$_3$, and Pt mesh were used as working, reference, and counter electrodes, respectively.

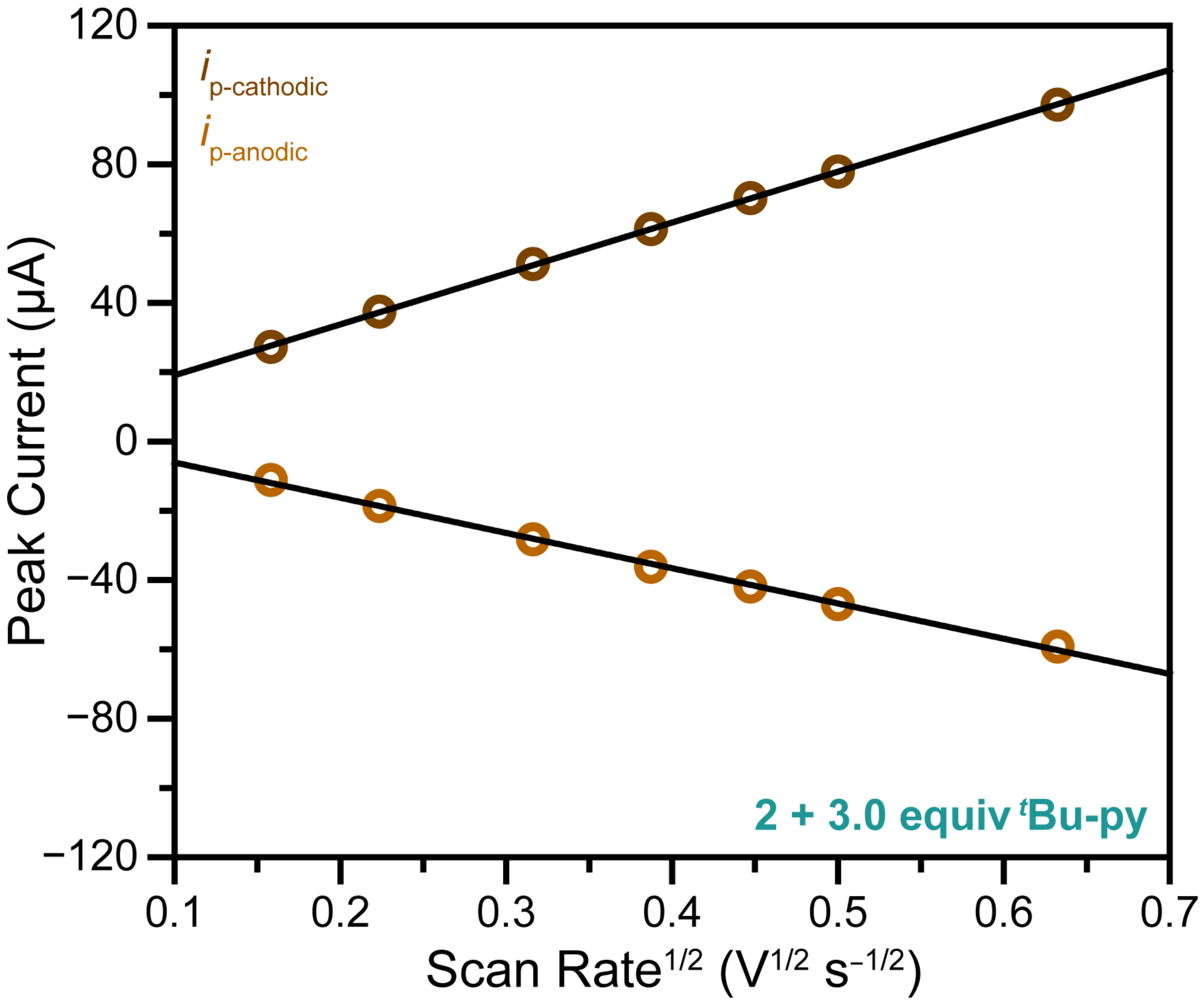

Figure S67. Randles–Ševčík plot for the Cu^{II}/Cu^{I} redox couple of **2** in the presence of 3.0 equiv of ^{t}Bu-py obtained from the variable-scan-rate cyclic voltammetry data shown in Figure S66. Colored circles denote experimental data, and black lines correspond to linear fits to the data. $i_{p\text{-cathodic}}$ and $i_{p\text{-anodic}}$ denote the cathodic and anodic peak current, respectively.

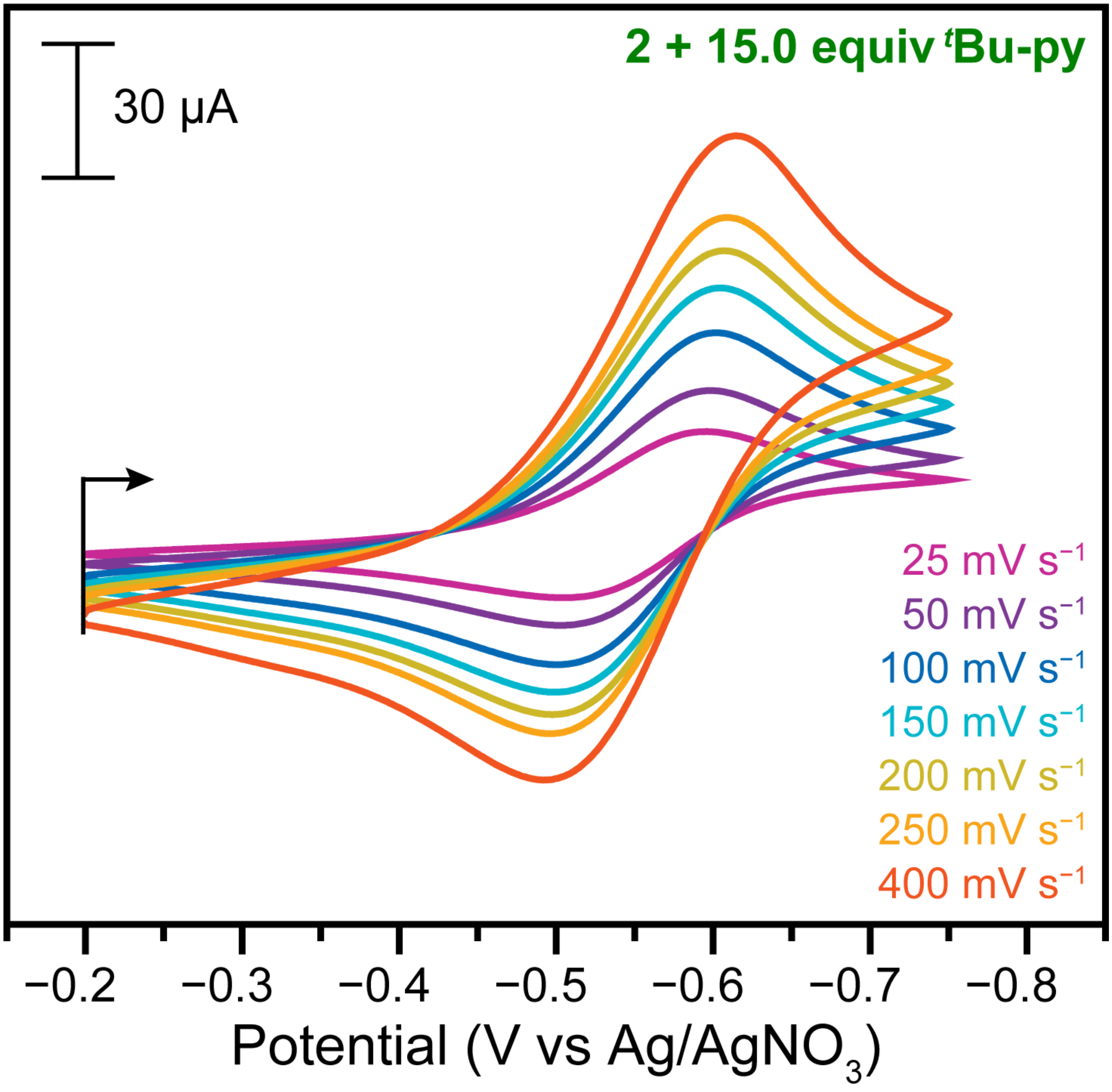

Figure S68. Cyclic voltammograms of 3.0 mM of **2** in the presence of 15.0 equiv of ^{t}Bu-py in dry MeCN containing 0.1 M ($^n Bu_4N$)(PF_6) supporting electrolyte collected at room temperature (23–25 °C) in the potential window containing the Cu^{II}/Cu^{I} redox couple using variable scan rates (25–400 mV s^{-1}). Colored numbers denote the scan rate used for each measurement. The vertical black line and arrow denote the open-circuit potential and scan direction, respectively. Glassy carbon, $Ag/AgNO_3$, and Pt mesh were used as working, reference, and counter electrodes, respectively.

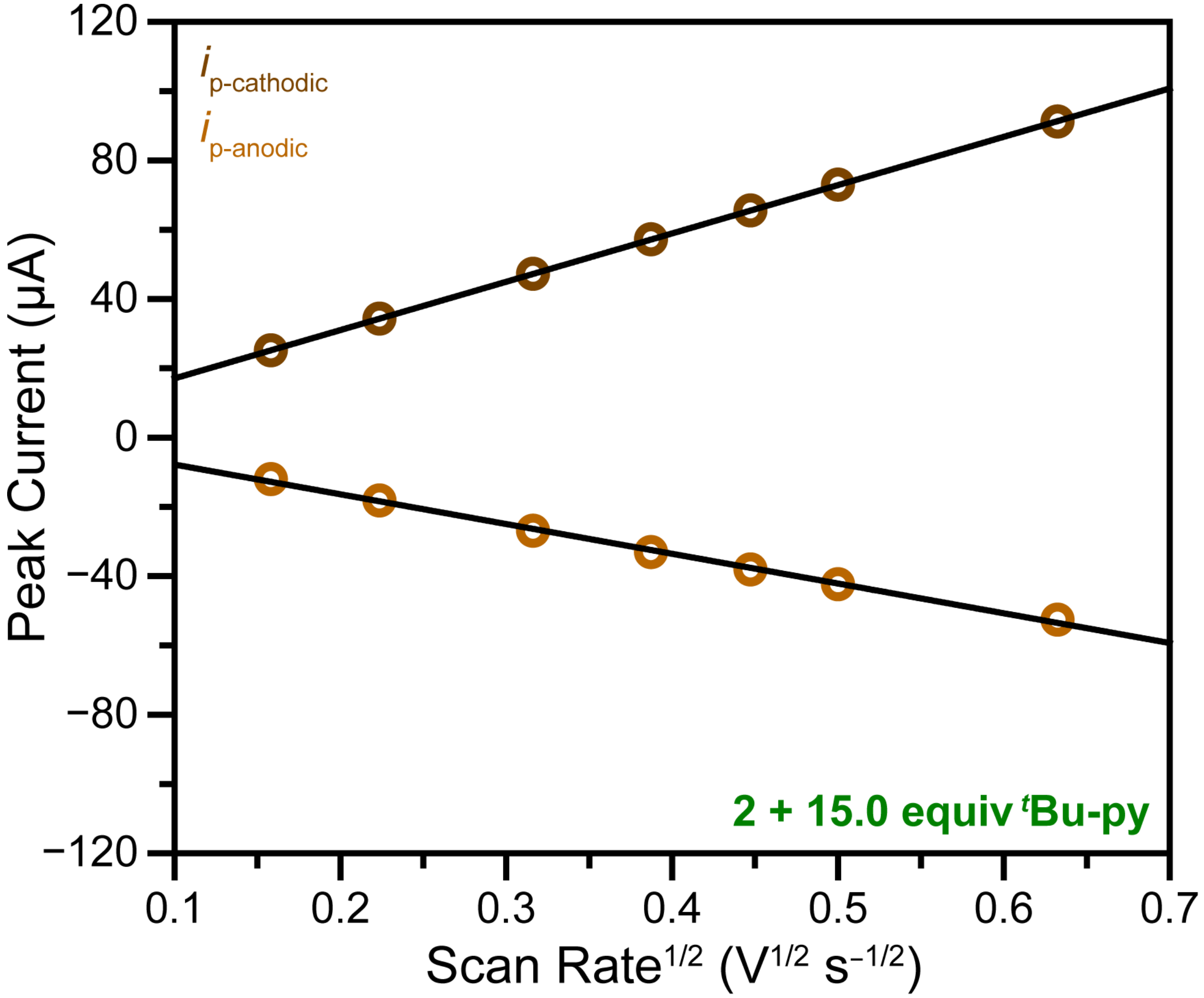

Figure S69. Randles–Ševčík plot for the Cu^{II}/Cu^{I} redox couple of **2** in the presence of 15.0 equiv of ^{t}Bu-py obtained from the variable-scan-rate cyclic voltammetry data shown in Figure S68. Colored circles denote experimental data, and black lines correspond to linear fits to the data. $i_{p\text{-cathodic}}$ and $i_{p\text{-anodic}}$ denote the cathodic and anodic peak current, respectively.

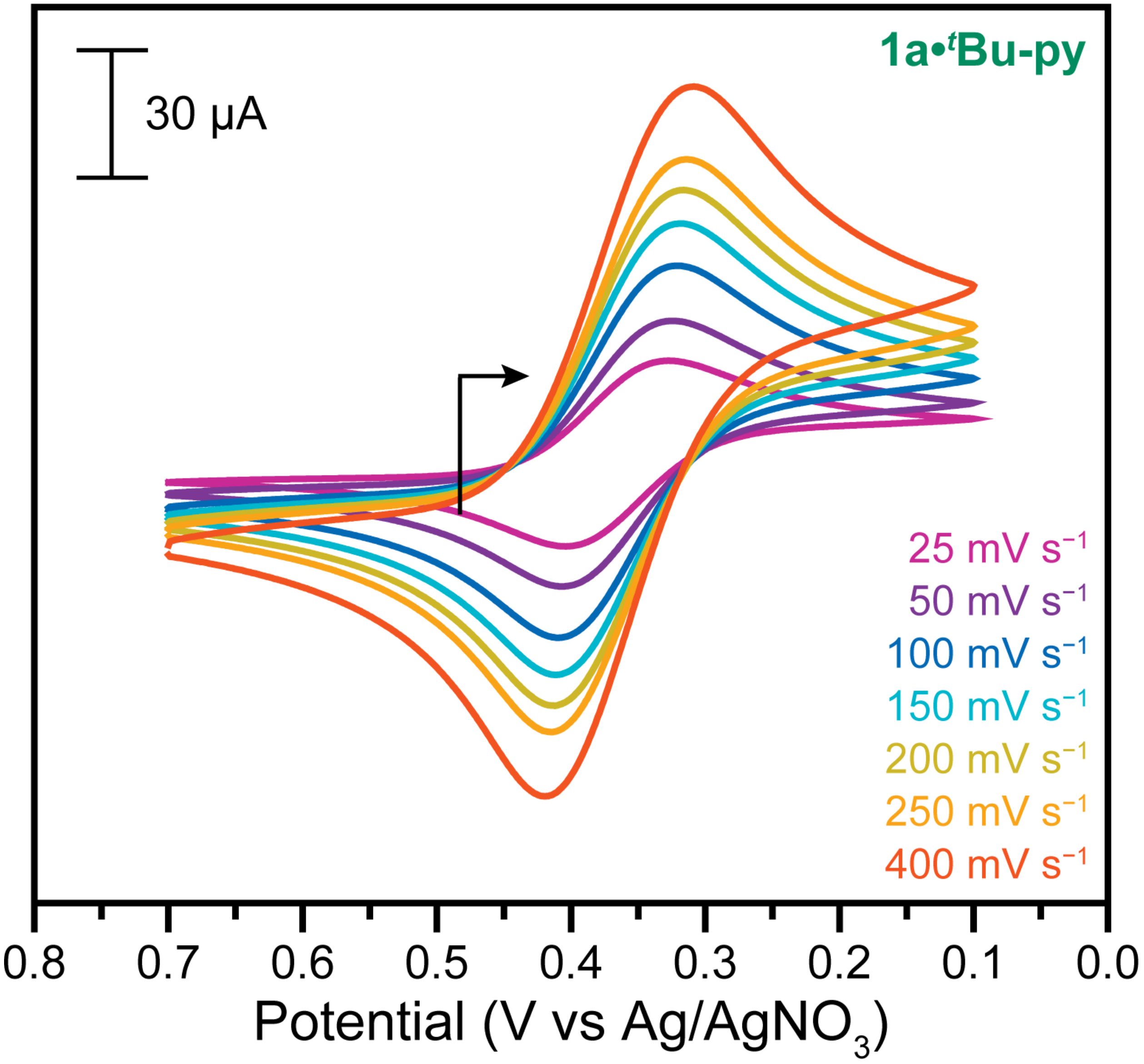

Figure S70. Cyclic voltammograms of 3.0 mM of **1a•^{t}Bu-py** in dry MeCN containing 0.1 M ($^n Bu_4N$)(PF_6) supporting electrolyte collected at room temperature (23–25 °C) in the potential window containing the Cu^{II}/Cu^{I} redox couple using variable scan rates (25–400 mV s^{-1}). Colored numbers denote the scan rate used for each measurement. The vertical black line and arrow denote the open-circuit potential and scan direction, respectively. Glassy carbon, Ag/$AgNO_3$, and Pt mesh were used as working, reference, and counter electrodes, respectively.

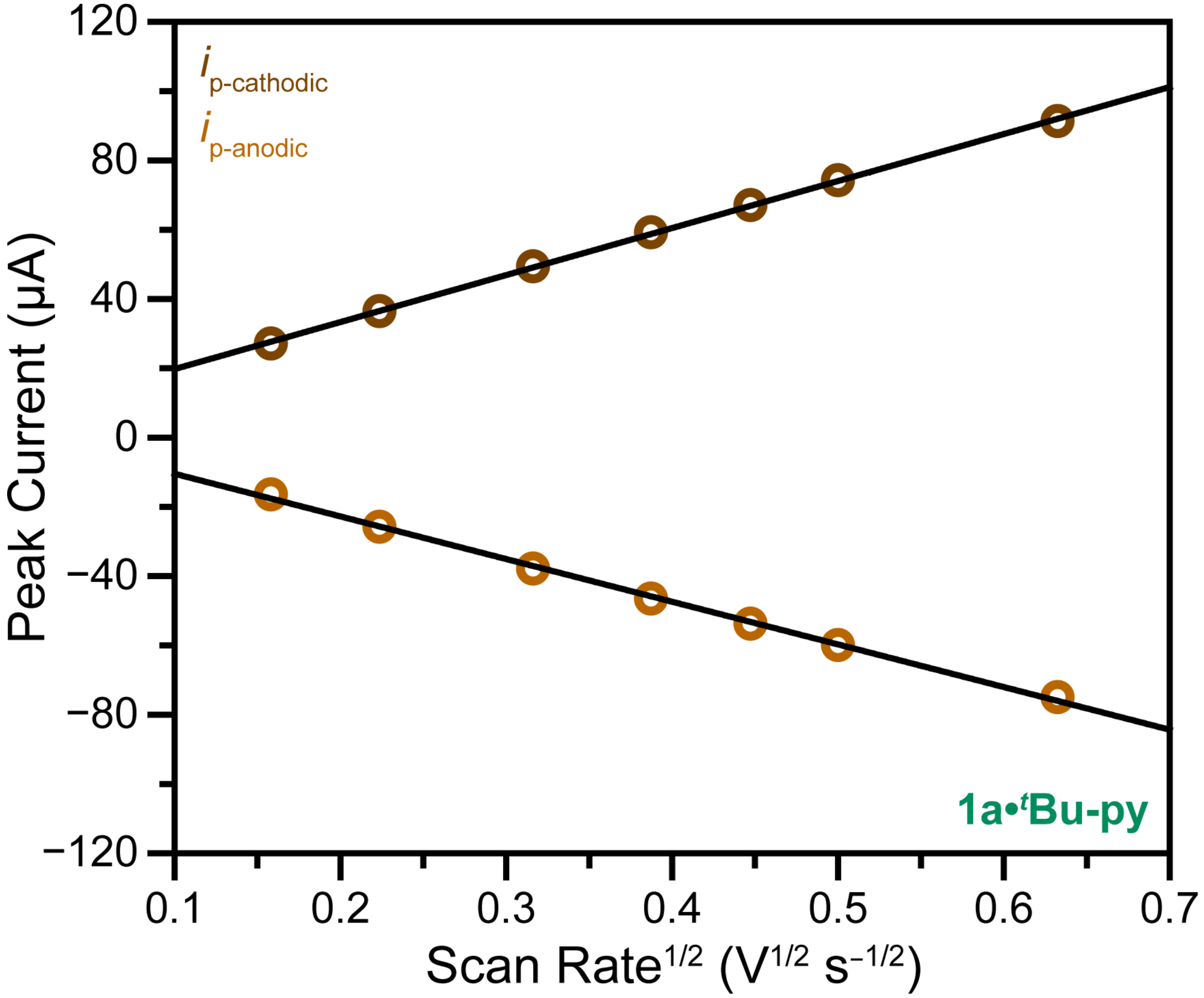

Figure S71. Randles–Ševčík plot for the Cu^{II}/Cu^{I} redox couple of **1a•*t*Bu-py** obtained from the variable-scan-rate cyclic voltammetry data shown in Figure S70. Colored circles denote experimental data, and black lines correspond to linear fits to the data. $i_{p\text{-cathodic}}$ and $i_{p\text{-anodic}}$ denote the cathodic and anodic peak current, respectively.

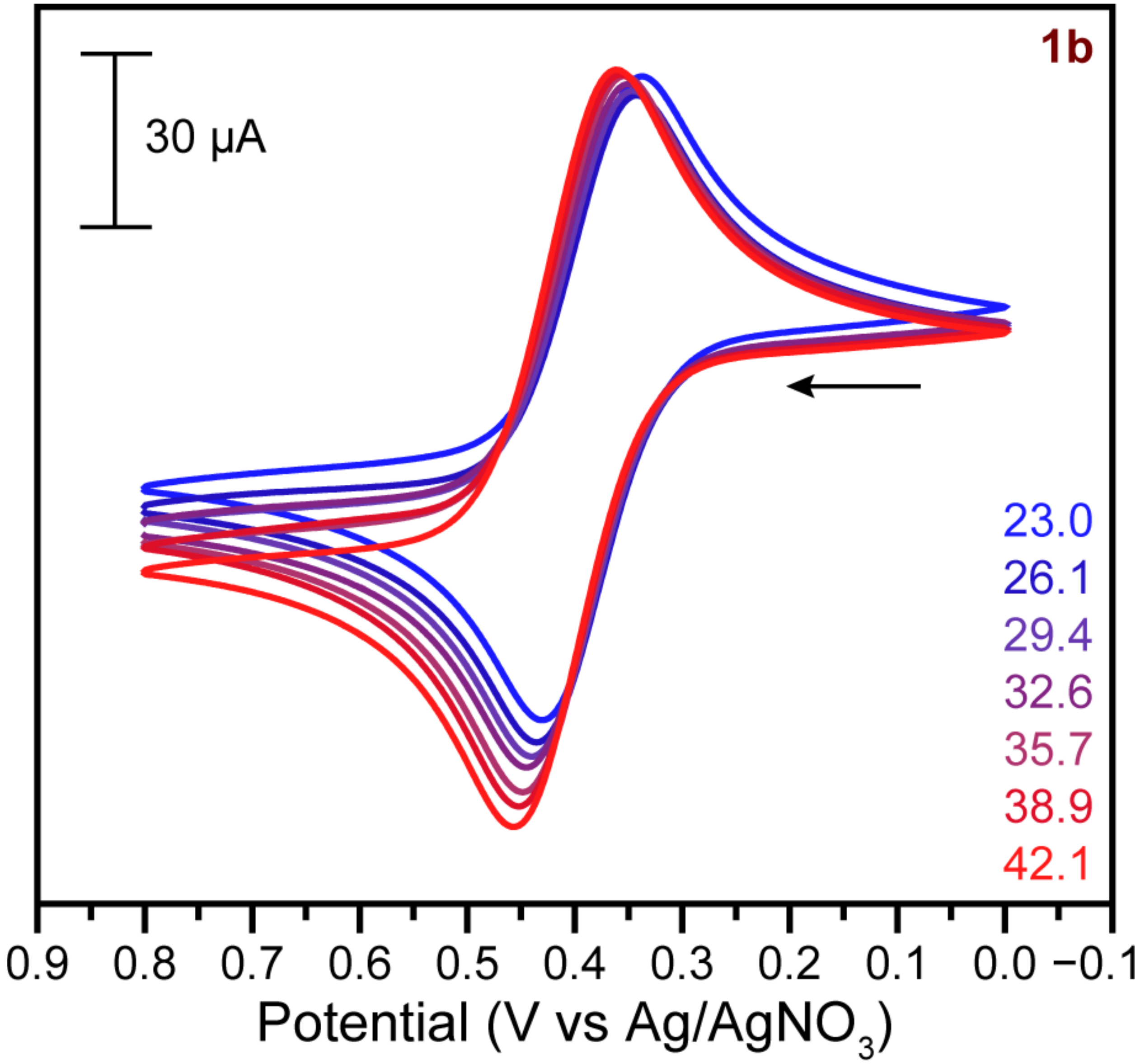

Figure S72. Cyclic voltammograms of 3 mM of **1b** in dry MeCN containing 0.1 M (^{n}Bu$_4$N)(PF$_6$) supporting electrolyte collected at variable temperatures (~23–42 °C) in the potential window containing the Cu^{II}/Cu^{I} redox couple using 100 mV s^{-1} scan rate. Colored numbers denote the temperature of the solution for each measurement in °C. Note that the E_{OCP} changes with temperature, thus, no vertical line denoting E_{OCP} is displayed. The black arrow indicates the scan direction. Glassy carbon, Ag/$AgNO_3$, and Pt mesh were used as working, reference, and counter electrodes, respectively.

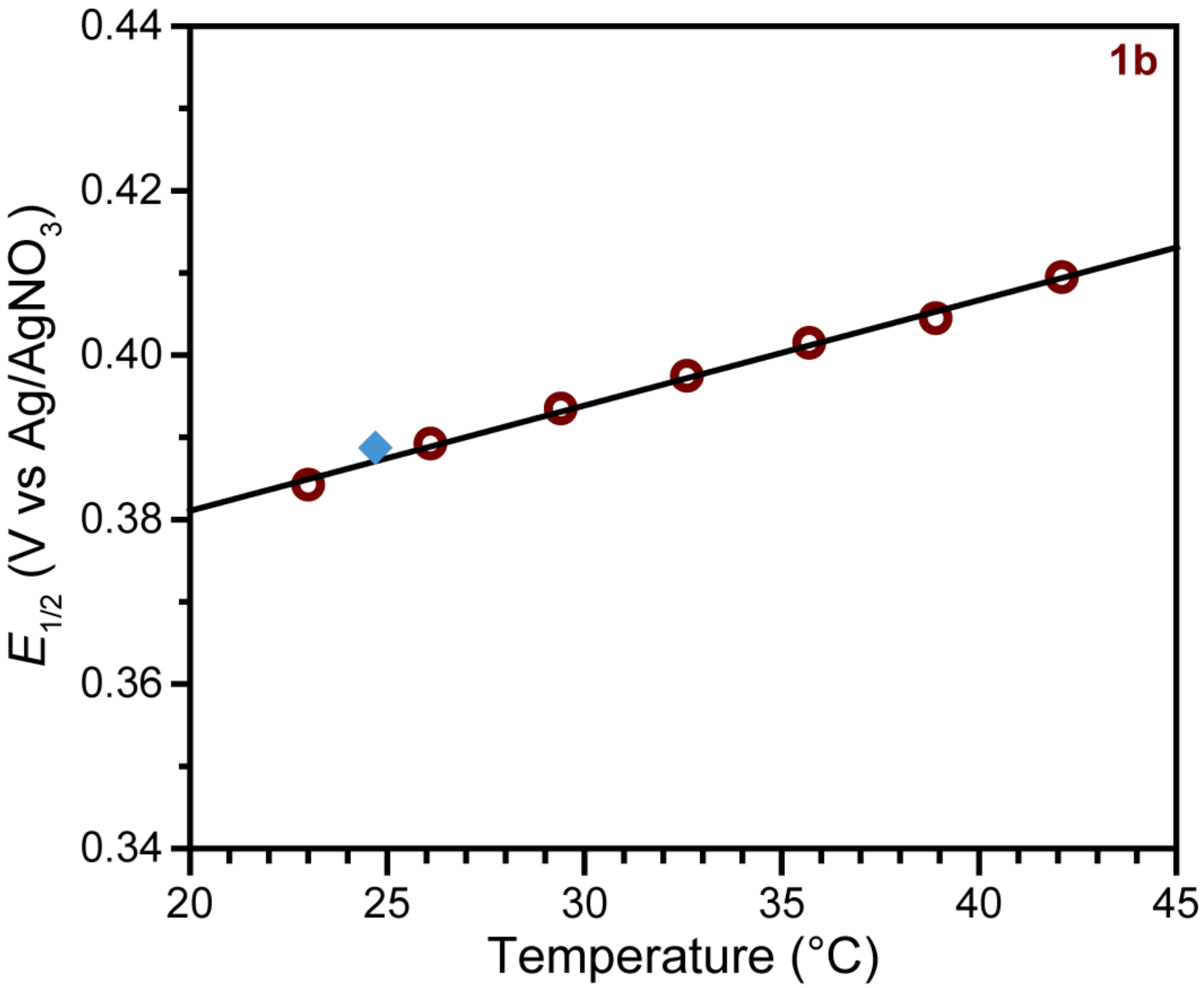

Figure S73. Plot of the temperature dependence of $E_{1/2}$ for the Cu^{II}/Cu^{I} redox couple of **1b** in dry MeCN containing 0.1 M ($^{n}Bu_4N$)(PF_6) supporting electrolyte, as obtained from the variable-temperature cyclic voltammetry data shown in Figure S72. Colored circles denote experimental data from the variable-temperature experiment in heating mode, and the black line corresponds to a linear fit to the data. The average slope from five independent measurements after correcting for the temperature coefficient of the reference electrode potential (eqs S9 and S10), which represents the average temperature coefficient, is α = 1.80(6) mV $°C^{-1}$ (Table 1). The error in the average slope corresponds to the standard deviation of individual measurements. The blue diamond corresponds to a data point measured after cooling the solution back down to close to room temperature.

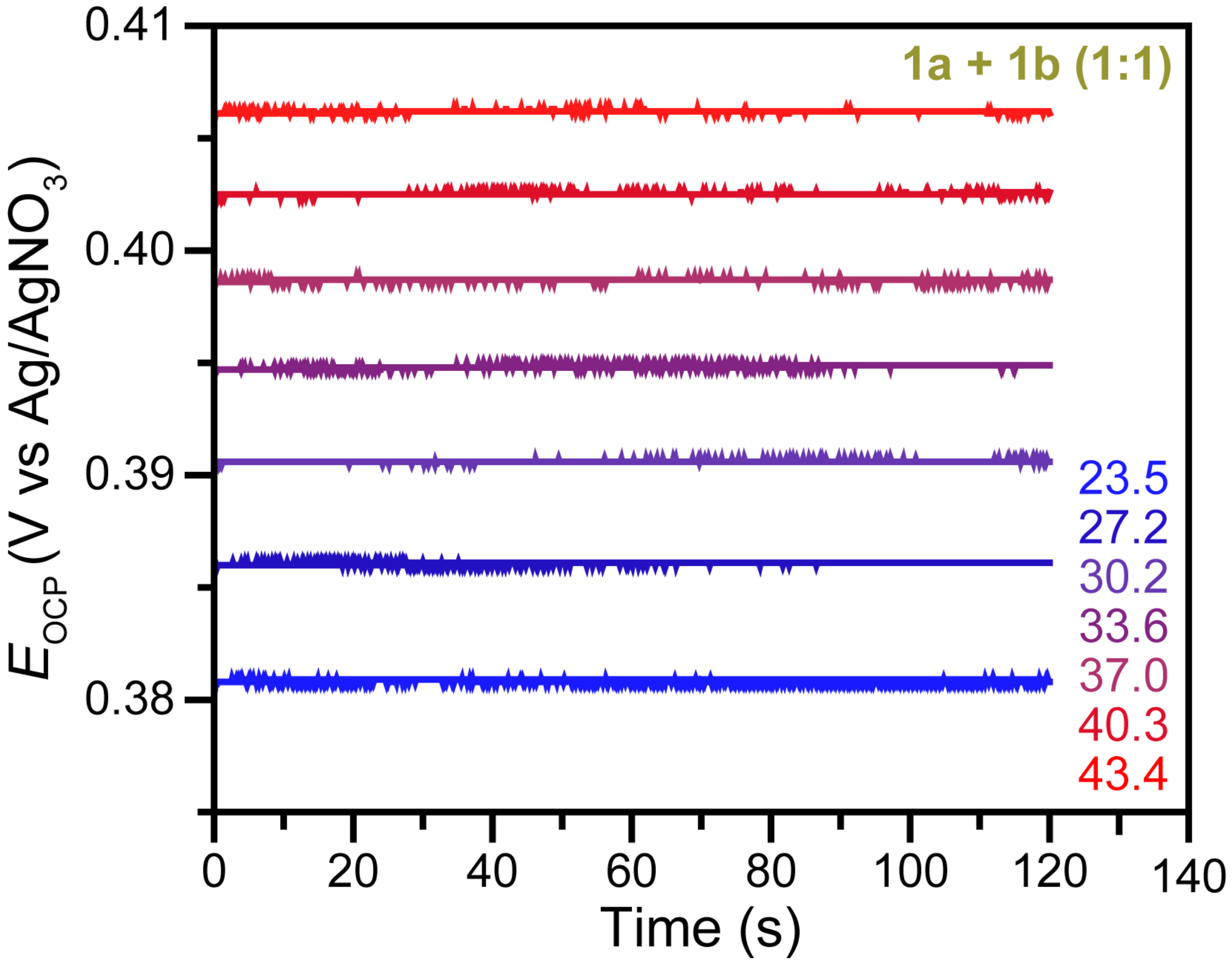

Figure S74. Plot of the time dependence of E_{OCP} for an equimolar solution of **1a** and **1b** (3 mM total) in dry MeCN containing 0.1 M (^{n}Bu$_4$N)(PF$_6$) supporting electrolyte collected at variable temperatures (~24–43 °C). The data were collected under isothermal conditions. Colored numbers denote the temperature of the solution for each measurement in °C. Glassy carbon, Ag/AgNO$_3$, and Pt mesh were used as working, reference, and counter electrodes, respectively.

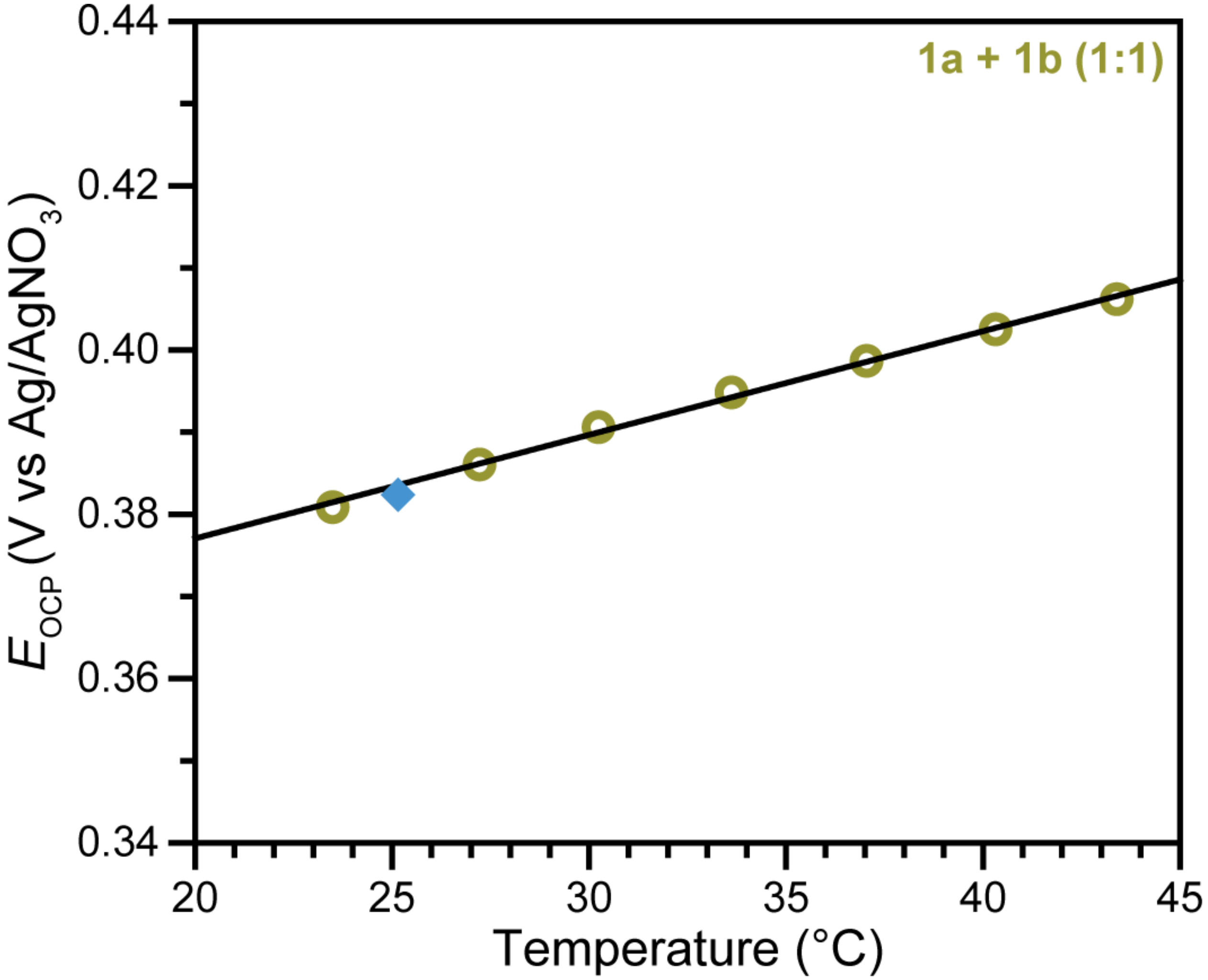

Figure S75. Plot of the temperature dependence of E_{OCP} for an equimolar solution of **1a** and **1b** (3 mM total) in dry MeCN containing 0.1 M (^{n}Bu$_4$N)(PF$_6$) supporting electrolyte, as obtained from the variable-temperature E_{OCP} data shown in Figure S74. Colored circles denote experimental data from the variable-temperature experiment in heating mode, and the black line corresponds to a linear fit to the data. The average slope from two independent measurements after correcting for the temperature coefficient of the reference electrode potential (eq S10), which may be estimated as the average temperature coefficient, is α = 1.76(5) mV °C^{-1}. The error in the average slope corresponds to the standard deviation of individual measurements. The blue diamond corresponds to a data point measured after cooling the solution back down to close to room temperature.

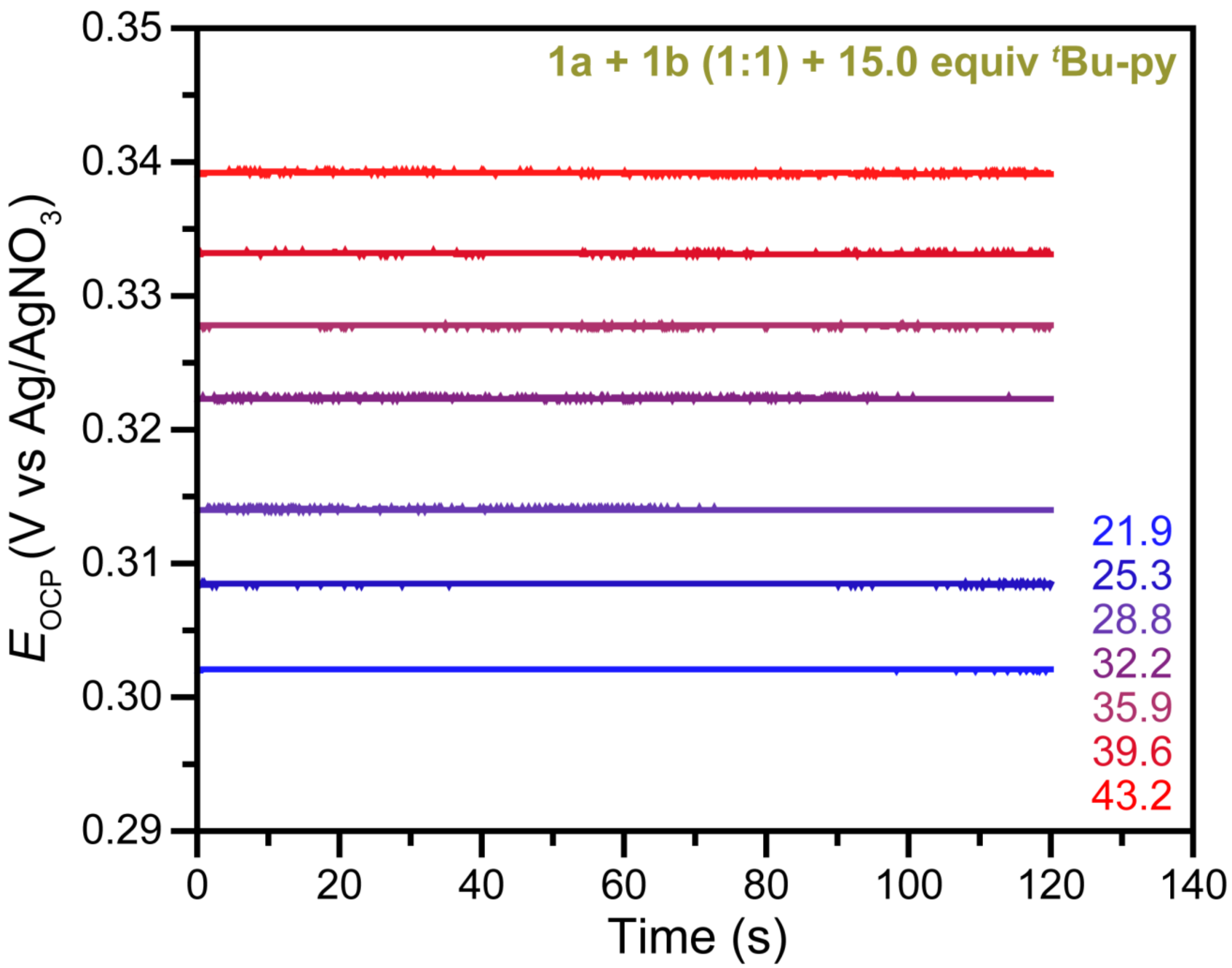

Figure S76. Plot of the time dependence of E_{OCP} for an equimolar solution of **1a** and **1b** (3 mM total) in the presence of 15.0 equiv of ^{t}Bu-py in dry MeCN containing 0.1 M ($^{n}Bu_4N$)(PF_6) supporting electrolyte collected at variable temperatures (~22–43 °C). The data were collected under isothermal conditions. Colored numbers denote the temperature of the solution for each measurement in °C. Glassy carbon, Ag/$AgNO_3$, and Pt mesh were used as working, reference, and counter electrodes, respectively.

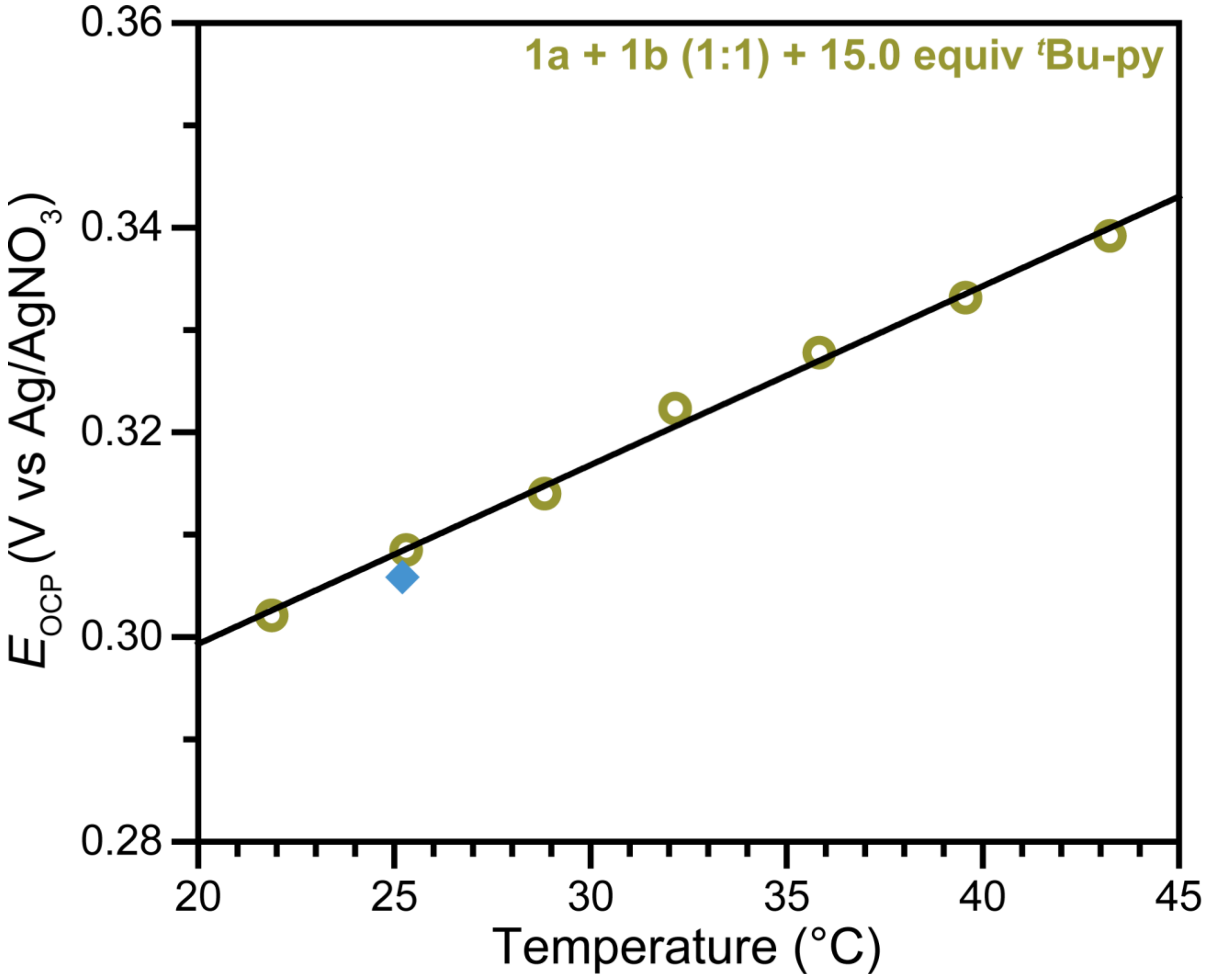

Figure S77. Plot of the temperature dependence of E_{OCP} for an equimolar solution of **1a** and **1b** (3 mM total) in the presence of 15.0 equiv of ^{t}Bu-py in dry MeCN containing 0.1 M (^{n}Bu$_4$N)(PF$_6$) supporting electrolyte, as obtained from the variable-temperature E_{OCP} data shown in Figure S76. Colored circles denote experimental data from the variable-temperature experiment in heating mode, and the black line corresponds to a linear fit to the data. The average slope from two independent measurements after correcting for the temperature coefficient of the reference electrode potential (eq S10), which may be estimated as the average temperature coefficient, is α = 2.26(6) mV °C^{-1}. The error in the average slope was obtained through error propagation of the standard error in the slope of the linear fit to individual E_{OCP} vs temperature data sets. The blue diamond corresponds to a data point measured after cooling the solution back down to close to room temperature.

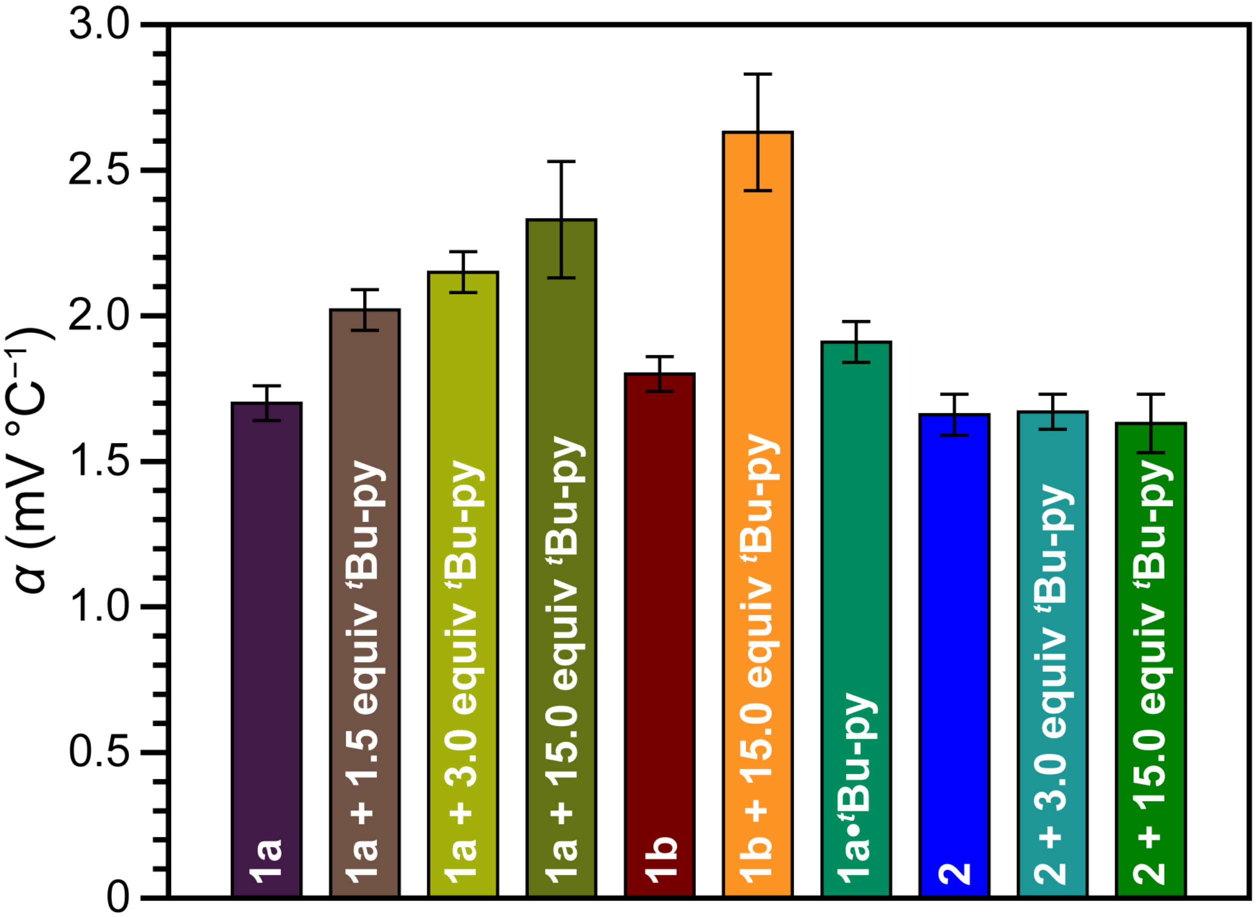

Figure S78. Comparison of the temperature coefficients (α) obtained from variable-temperature cyclic voltammetry experiments (~23–44 °C) for **1a**, **1b**, **1a•^{t}Bu-py**, and **2** in the absence and presence of 1.5, 3.0, and 15.0 equiv of ^{t}Bu-py in dry MeCN containing 0.1 M (^{n}Bu$_4$N)(PF$_6$) supporting electrolyte. Error bars denote the standard deviation of measurements of at least three independently prepared samples.

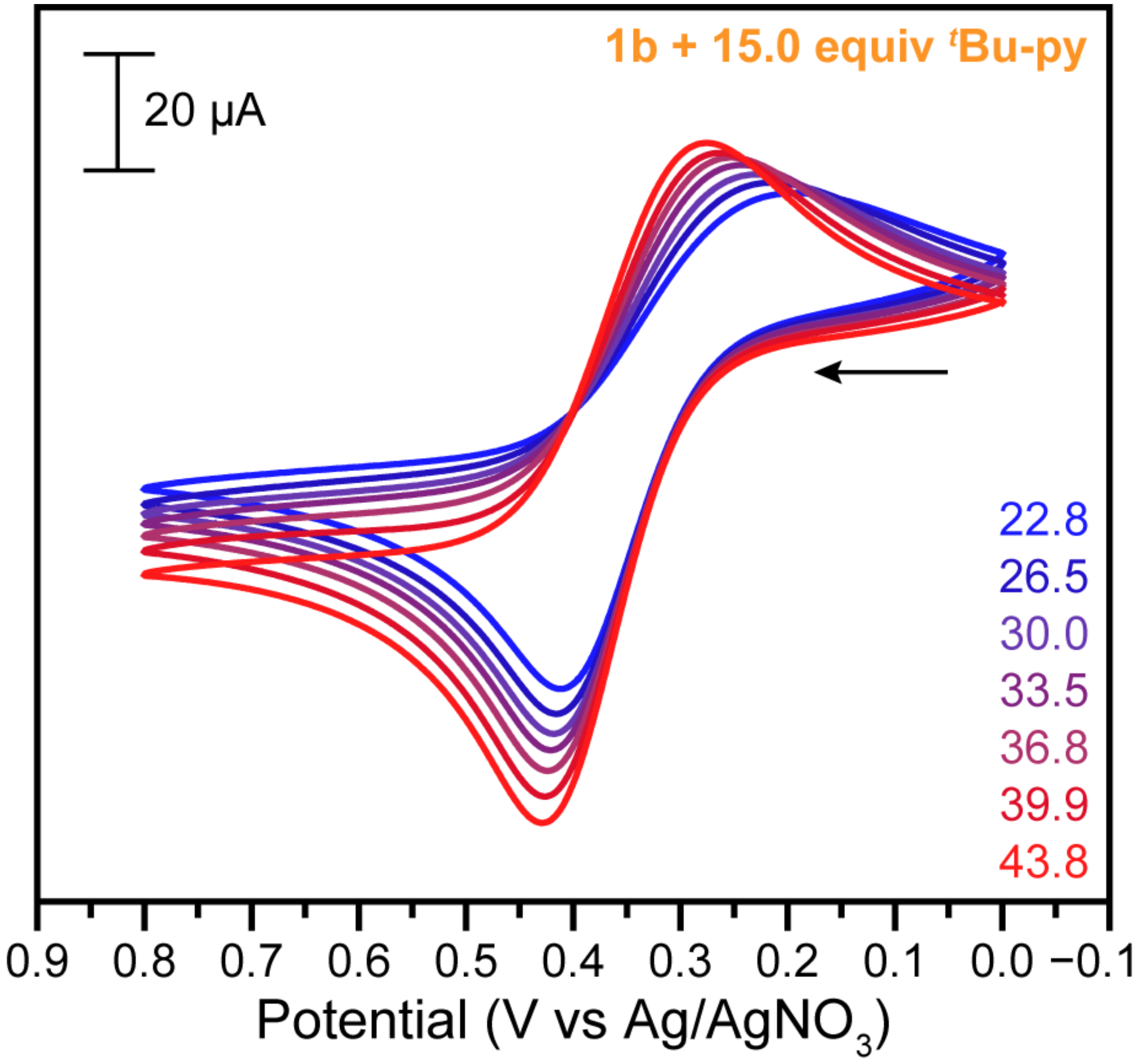

Figure S79. Cyclic voltammograms of 3 mM of **1b** in the presence of 15.0 equiv of ^{t}Bu-py in dry MeCN containing 0.1 M ($^n Bu_4N$)(PF_6) supporting electrolyte collected at variable temperatures (~23–44 °C) in the potential window containing the Cu^{II}/Cu^{I} redox couple using 100 mV s^{-1} scan rate. Colored numbers denote the temperature of the solution for each measurement in °C. Note that the data were collected after performing 40 CV cycles in the same potential range and using the same scan rate at room temperature. This preconditioning was performed to provide a stable $E_{1/2}$ prior to variable-temperature CV measurements. Further note that the E_{OCP} changes with temperature, thus, no vertical line denoting E_{OCP} is displayed. The black arrow indicates the scan direction. Glassy carbon, $Ag/AgNO_3$, and Pt mesh were used as working, reference, and counter electrodes, respectively.

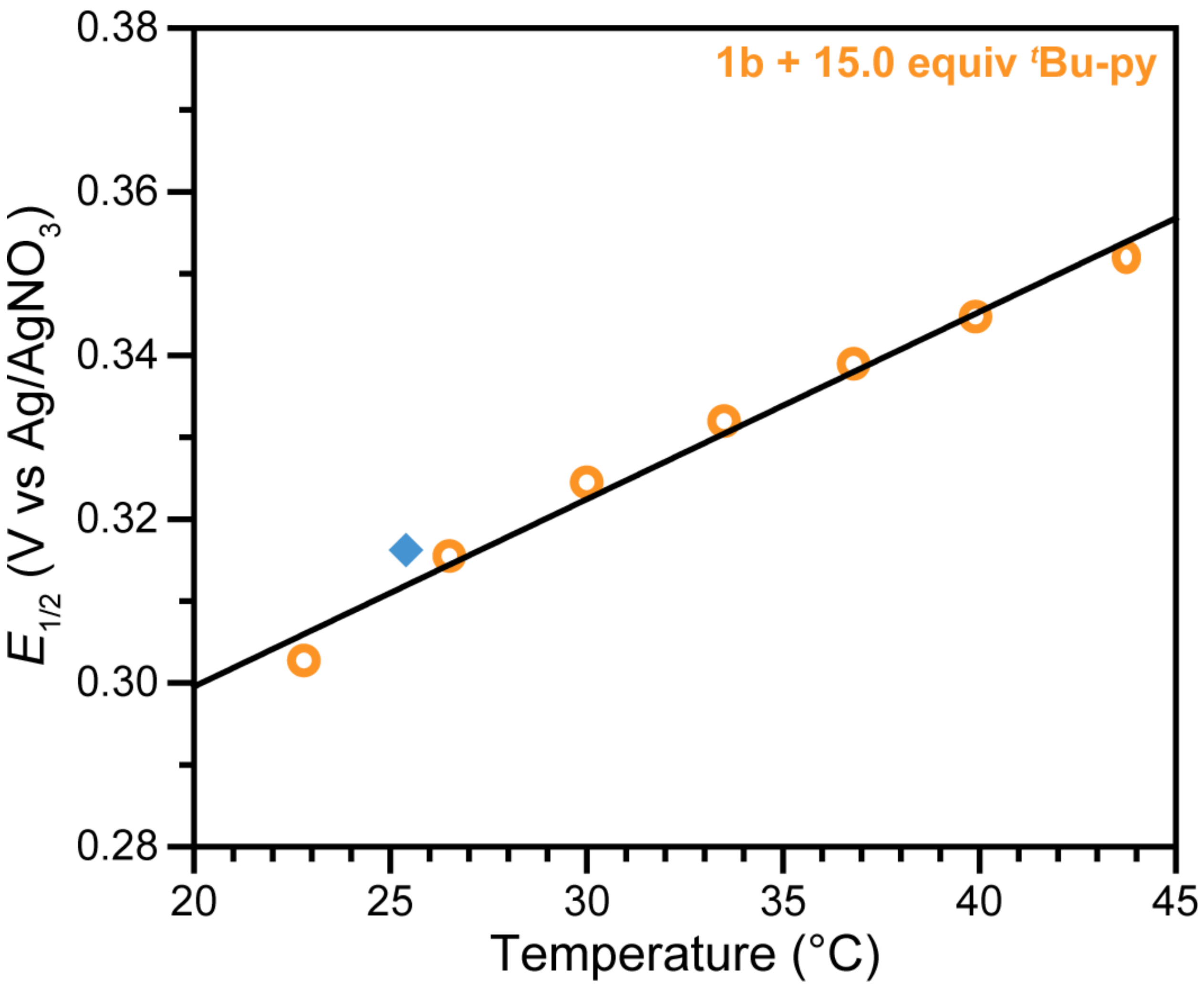

Figure S80. Plot of the temperature dependence of $E_{1/2}$ for the Cu^{II}/Cu^{I} redox couple of **1b** in the presence of 15.0 equiv of ^{t}Bu-py in dry MeCN containing 0.1 M (${}^{n}Bu_4N$)(PF_6) supporting electrolyte, as obtained from the variable-temperature cyclic voltammetry data shown in Figure S79. Colored circles denote experimental data from the variable-temperature experiment in heating mode, and the black line corresponds to a linear fit to the data. The average slope from four independent measurements after correcting for the temperature coefficient of the reference electrode potential (eqs S9 and S10), which represents the average temperature coefficient, is α = 2.6(2) mV °C^{-1}. The error in the average slope corresponds to the standard deviation of individual measurements. The blue diamond corresponds to a data point measured after cooling the solution back down to close to room temperature.

D. Supplementary Tables

Table S1. Summary of crystallographic data obtained for **1a′**, **1a•^{t}Bu-py**, and **3′**.

	**1a′**	**1a•^{t}Bu-py**	**3′**
Empirical formula	$C_{32}H_{30}CuF_{12}N_6P_2$	$C_{38}H_{39}Cl_2CuF_{12}N_5P_2$	$C_{44}H_{68}CuF_{12}N_6OP_2$
Formula wt (g mol^{-1})	852.10	990.12	1050.52
Crystal system	Monoclinic	Triclinic	Triclinic
Space group	$P2_1/n$	$P\bar{1}$	$P\bar{1}$
Wavelength (Å)	1.54184	1.54184	1.54184
Temperature (K)	100.00(10)	173.00(10)	100.00(10)
a (Å)	11.95830(10)	10.6152(3)	15.00440(10)
b (Å)	15.08310(10)	11.8173(3)	15.1642(2)
c (Å)	19.28260(10)	18.4779(3)	15.59870(10)
α (°)	90	96.074(2)	84.4800(10)
β (°)	104.5120(10)	104.745(2)	89.6440(10)
γ (°)	90	110.322(2)	63.8000(10)
V (Å^3)	3367.00(4)	2053.30(9)	3167.14(6)
Z	4	2	2
ρ_{calcd} (g cm^{-3})	1.681	1.601	1.102
μ (mm^{-1})	2.749	3.506	1.550
Reflns coll./unique	57370/7221	47173/8733	104036/13581
$R_{int}{}^a$	0.0426	0.0380	0.0574
R_1 ($I > 2\sigma(I)$)^b	0.0369	0.0437	0.0570
wR_2 (*all*)^c	0.0977	0.1259	0.1626
GoF	1.058	1.095	1.048

$^aR_{int} = \Sigma|F_0^2 - \langle F_0^2\rangle| / \Sigma|F_0^2|$.

$^bR_1 = \Sigma||F_0| - |F_c|| / \Sigma|F_0|$.

$^cwR_2 = [\Sigma[w(F_0^2 - F_c^2)^2] / \Sigma[w(F_0^2)^2]]^{1/2}$.

Table S2. Selected mean interatomic distances (Å) and angles (°), and structural parameters for the cationic complexes in **1a′**, **1a•^{t}Bu-py**, **1b**, and **2**.

	**1a′**	**1a•^{t}Bu-py**	**1b**^{f}	**2**^{h}
T (K)^a	100	173	90	295
Cu–N_{phen}	2.0851(8)	2.088(1)	2.048(1)	—
Cu–N_{ligand}^{b}	2.016(2)	2.049(2)	—	—
Cu–N^c	2.0713(7)	2.0803(9)	2.048(1)	2.1116(3)
N_{phen}–Cu–N_{phen}	107.48(3)	106.99(4)	110.28(4)	—
N_{phen}–Cu–N_{ligand}^{b}	106.94(3)	107.42(4)	—	—
N–Cu–N^d	107.26(2)	107.16(3)	110.28(4)	105.287(3)
τ / Σ_{sum}	0.19(2)^e	0.039(2)^e	0.71(1)^g	116.89(5)^i

^{a}Temperature of crystal structure data collection in K. ^{b}Ligand denotes MeCN or ^{t}Bu-py molecule. ^{c}Average Cu–N bond distance. ^{d}Average N–Cu–N bond angle. ^{e}τ_5 geometry index for 5-coordinate complexes: $\tau_5 = (\beta - \alpha)/60°$, where α and β are the two largest angles in [CuN_5] and $\beta > \alpha$.[34] ^{f}Data taken from literature, see ref 3. ^{g}τ_4 geometry index for 4-coordinate complexes: $\tau_4 = (360° - (\alpha + \beta))/141°$, where α and β are the two largest angles in [CuN_4].[35] ^{h}Data taken from literature, see ref 5. ^{i}Octahedral distortion parameter (Σ_{sum}) = sum of the absolute deviation from 90° of each 12 *cis* angle in [CuN_6].[36]

Table S3. Ratio of ^{t}Bu-py binding to **1a** as a function of equiv of ^{t}Bu-py in dry MeCN, as obtained from UV–visible–NIR titration experiments at 20 °C and monitoring of the absorbance at 725 nm and 847 nm wavelengths.[a]

Equiv of ^{t}Bu-py	Ratio (725 nm)	Ratio (847 nm)
0	0	0
0.2	0.03	0.05
0.4	0.11	0.11
0.6	0.18	0.21
0.8	0.25	0.26
1.0	0.32	0.31
1.25	0.39	0.44
1.5	0.45	0.49
2.0	0.55	0.57
2.5	0.63	0.67
3.0	0.69	0.69
4.0	0.78	0.78
5.0	0.84	0.86
7.5	0.92	0.93
10.0	0.96	0.99
12.5	1.00	0.99
15.0	1.01	1.02
17.5	1.01	1.00
20.0	1.01	1.01
25.0	1.01	1.01
30.0	1.00	0.97
40.0	0.97	1.00
50.0	0.92	0.92

[a]See UV–Visible–NIR Absorption Spectroscopy section for experimental details.

Table S4. Selected mean interatomic distances (Å) and angles (°), and structural parameters for the cationic complex in **3′** at 100 K.

	**3′**
Cu–$N_{t\text{Bu-py}}$	2.0249(9)
Cu–N_{MeCN}	2.526(2)
Cu–N[a]	2.1919(8)
cis-$N_{t\text{Bu-py}}$–Cu–$N_{t\text{Bu-py}}$	90.01(4)
trans-$N_{t\text{Bu-py}}$–Cu–$N_{t\text{Bu-py}}$	177.80(5)
$N_{t\text{Bu-py}}$–Cu–N_{MeCN}	90.02(3)
N_{MeCN}–Cu–N_{MeCN}	174.51(6)
N–Cu–N[b]	107.35(2)
τ_4[c]	0.031(3)
Σ_{sum}[d]	18.12(2)

[a]Average Cu–N bond distance. [b]Average N–Cu–N bond angle. [c]τ_4 geometry index for the equatorial [CuN_4] plane: $\tau_4 = (360° - (\alpha + \beta))/141°$, where α and β are the two largest angles in the [CuN_4] plane.[35] [d]Octahedral distortion parameter (Σ_{sum}) = sum of the absolute deviation from 90° of each 12 *cis* angle in [CuN_6].[36]

Table S5. Summary of anodic and cathodic diffusion coefficients of the Cu^{II}/Cu^{I} redox couple for **1a**, **1b**, **1a•^{t}Bu-py**, and **2** in the absence and presence of 3.0 and 15.0 equiv of ^{t}Bu-py in dry MeCN containing 0.1 M (^{n}Bu$_4$N)(PF$_6$) at room temperature (23–25 °C), as calculated from Randles–Ševčík analysis for reversible redox couples (eq S7; Figures S52–S71).[a]

Compound	Equiv of ^{t}Bu-py	$D_{0\text{-anodic}}$[b] (cm^2 s^{-1})	$D_{0\text{-cathodic}}$[b] (cm^2 s^{-1})	$D_{0\text{-average}}$[c] (cm^2 s^{-1})
**1a**	0	5.8(5) × 10^{-6}	7.0(9) × 10^{-6}	6.4(6) × 10^{-6}
	3.0	4.4(5) × 10^{-6}	5.3(2) × 10^{-6}	4.9(3) × 10^{-6}
	15.0	2.8(3) × 10^{-6}	2.7(2) × 10^{-6}	2.8(2) × 10^{-6}
**1b**	0	6.7(3) × 10^{-6}	5.8(5) × 10^{-6}	6.3(3) × 10^{-6}
	3.0	6.3(8) × 10^{-6}	4.2(5) × 10^{-6}	5.3(5) × 10^{-6}
	15.0	4.3(4) × 10^{-6}	1.9(2) × 10^{-6}	3.1(3) × 10^{-6}
**1a•^{t}Bu-py**	0	4.7(5) × 10^{-6}	5.5(6) × 10^{-6}	5.1(4) × 10^{-6}
**2**	0	2.9(2) × 10^{-6}	6.1(3) × 10^{-6}	4.5(2) × 10^{-6}
	3.0	3.0(3) × 10^{-6}	6.5(4) × 10^{-6}	4.8(3) × 10^{-6}
	15.0	2.3(1) × 10^{-6}	6.3(2) × 10^{-6}	4.3(2) × 10^{-6}

[a]A temperature of 25 °C and molar masses of copper complexes without MeCN and ^{t}Bu-py binding beyond the chemical formulas of isolated powder samples were used for the calculations. [b]The reported value for each oxidation or reduction peak is obtained from the anodic or cathodic peak current, respectively, using eq S7 (reversible redox couple). Error bars denote the standard deviation of measurements of three independently prepared samples. [c]Average value of diffusion coefficients obtained using anodic and cathodic peak currents. Error bars were obtained from error propagation.

Table S6. Summary of anodic and cathodic diffusion coefficients of the Cu^{II}/Cu^{I} redox couple for **1a**, **1b**, **1a•^{t}Bu-py**, and **2** in the absence and presence of 3.0 and 15.0 equiv of ^{t}Bu-py in dry MeCN containing 0.1 M (^{n}Bu$_4$N)(PF$_6$) at room temperature (23–25 °C), as calculated from Randles–Ševčík analysis for irreversible redox couples (eq S8; Figures S52–S71).[a]

Compound	Equiv of ^{t}Bu-py	$D_{0\text{-anodic}}$[b] (cm^2 s^{-1})	$D_{0\text{-cathodic}}$[b] (cm^2 s^{-1})	$D_{0\text{-average}}$[c] (cm^2 s^{-1})
**1a**	0	9.4(7) × 10^{-6}	1.1(2) × 10^{-5}	1.0(1) × 10^{-5}
	3.0	7.2(7) × 10^{-6}	8.5(3) × 10^{-6}	7.9(4) × 10^{-6}
	15.0	4.6(5) × 10^{-6}	4.3(3) × 10^{-6}	4.5(3) × 10^{-6}
**1b**	0	1.1(1) × 10^{-5}	9.4(8) × 10^{-6}	1.0(1) × 10^{-5}
	3.0	1.0(2) × 10^{-5}	6.8(8) × 10^{-6}	8.5(8) × 10^{-6}
	15.0	6.9(7) × 10^{-6}	3.1(3) × 10^{-6}	5.0(4) × 10^{-6}
**1a•^{t}Bu-py**	0	7.6(7) × 10^{-6}	8.9(9) × 10^{-6}	8.3(6) × 10^{-6}
**2**	0	4.7(4) × 10^{-6}	9.8(5) × 10^{-6}	7.3(4) × 10^{-6}
	3.0	4.8(4) × 10^{-6}	1.1(1) × 10^{-5}	7.7(4) × 10^{-6}
	15.0	3.8(1) × 10^{-6}	1.0(1) × 10^{-5}	7.0(2) × 10^{-6}

[a]A temperature of 25 °C and molar masses of copper complexes without MeCN and ^{t}Bu-py binding beyond the chemical formulas of isolated powder samples were used for the calculations. [b]The reported value for each oxidation or reduction peak is obtained from the anodic or cathodic peak current, respectively, using eq S8 (irreversible redox couple). Error bars denote the standard deviation of measurements of 2–3 independently prepared samples. [c]Average value of diffusion coefficients obtained using anodic and cathodic peak currents. Error bars were obtained from error propagation.

Table S7. Comparison of diffusion coefficients for **1b** obtained from different analyses.

Method	Equiv of ^{t}Bu-py	D_0 (cm^2 s^{-1})
Randles–Ševčík (eq S7)[a]	0	$6.3(3) \times 10^{-6}$
	15.0	$3.1(3) \times 10^{-6}$
Randles–Ševčík (eq S8)[b]	0	$1.0(1) \times 10^{-5}$
	15.0	$5.0(4) \times 10^{-6}$
Stokes–Einstein[c]	0	8.8×10^{-6}
DOSY NMR[d]	0	$1.3(2) \times 10^{-5}$
	15.0	$1.3(2) \times 10^{-5}$

[a]A temperature of 25 °C and molar mass of the copper complex without MeCN and ^{t}Bu-py binding beyond the chemical formula of the isolated powder sample were used for the calculations. Average value of diffusion coefficients obtained from anodic and cathodic peak currents using eq S7 (Table S5). [b]A temperature of 25 °C and molar mass of the copper complex without MeCN and ^{t}Bu-py binding beyond the chemical formula of the isolated powder sample were used for the calculations. Average value of diffusion coefficients obtained from anodic and cathodic peak currents using eq S8 (Table S6). [c]The Stokes–Einstein equation: $D = k_B T/(6\pi\eta r)$, with temperature (T) of 25 °C, dynamic viscosity (η) of 0.346 mPa·s (see ref 37 for the dynamic viscosity of MeCN), and radius of cationic complex (r) of 7.32 Å (see ref 3; obtained from reported crystal structure data of **1b** at 90 K) was used for the calculations. The diameter of the cationic complex was estimated as the longest interatomic distance (between two H atoms) plus the van der Waals radius of each H atom (1.20 Å). [d]Determined in CD_3CN solution at 19–22 °C (see Experimental Section for details of other experimental parameters and estimation of error bars).

Table S8. Summary of temperature coefficients reported in the literature for Cu-based redox couples and metal complexes with overall 2+/1+ charge-state change in homogeneous MeCN solution.[a]

Redox Couple	α (mV °C^{-1})	Temperature Range (°C)	Solvent	Reference
Cu^{II}/Cu^{0} $[Cu]^{2+/0}$ [b]	1.66	20–90	Water	38
Cu^{II}/Cu^{0} $[Cu]^{2+/0}$ [b]	2.12	25–70	Water	39
Fe^{III}/Fe^{II} $[Fe_2]^{2+/1+}$	1.07(4)	24–46	MeCN	26
Fe^{III}/Fe^{II} $[FeZn]^{2+/1+}$	1.07(4)	23–46	MeCN	26
Ru^{II}/Ru^{I} $[Ru(bpy)_3]^{2+/1+}$ [c]	0.73	Not reported	MeCN	33
Cr^{II}/Cr^{I} $[Cr(bpy)_3]^{2+/1+}$ [c]	0.67	Not reported	MeCN	33

[a]The provided list contains selected relevant examples reported in the literature for comparison to the work reported herein. [b]Corrosion of Cu electrode takes place. [c]The abbreviation bpy denotes 2,2′-bipyridine.

E. References

(1) Zhou, G.; Zhang, L.; Xia, Y.; Xu, H.; Yin, W.; Wang, S.; Yi, J.; Zhu, X.; Ning, X.; Wang, X. Highly Selective Photocatalytic CO_2 Reduction by Metal-N_4 Dynamically Generated from Atomically Dispersed Copper. *Chemical Engineering Journal* **2023**, *477*, No. 147040. DOI: 10.1016/j.cej.2023.147040

(2) Leandri, V.; Daniel, Q.; Chen, H.; Sun, L.; Gardner, J. M.; Kloo, L. Electronic and Structural Effects of Inner Sphere Coordination of Chloride to a Homoleptic Copper(II) Diimine Complex. *Inorganic Chemistry* **2018**, *57* (8), 4556–4562. DOI: 10.1021/acs.inorgchem.8b00225

(3) Kovalevsky, A. Y.; Gembicky, M.; Novozhilova, I. V.; Coppens, P. Solid-State Structure Dependence of the Molecular Distortion and Spectroscopic Properties of the Cu(I) Bis(2,9-dimethyl-1,10-phenanthroline) Ion. *Inorganic Chemistry* **2003**, *42* (26), 8794–8802. DOI: 10.1021/ic0348805

(4) Meyer, A.; Schnakenburg, G.; Glaum, R.; Schiemann, O. (Bis(terpyridine))copper(II) Tetraphenylborate: A Complex Example for the Jahn–Teller Effect. *Inorganic Chemistry* **2015**, *54* (17), 8456–8464. DOI: 10.1021/acs.inorgchem.5b01157

(5) Arriortua, M. I.; Rojo, T.; Amigó, J. M.; Germain, G.; Declercq, J. P. Bis(2,2′:6′,2″-terpyridine)copper(II) Hexafluorophosphate. *Acta Crystallographica Section B: Structural Science, Crystal Engineering and Materials* **1982**, *38* (4), 1323–1324. DOI: 10.1107/S0567740882005676

(6) CrysAlis^{Pro}, *version 171.43.118a;* Rigaku Corporation: Oxford 2024.

(7) Sheldrick, G. M. *SHELXT* – Integrated Space-Group and Crystal-Structure Determination. *Acta Crystallographica Section A: Foundations and Advances* **2015**, *71* (1), 3–8. DOI: 10.1107/S2053273314026370

(8) Sheldrick, G. M. Crystal Structure Refinement with *SHELXL*. *Acta Crystallographica Section C: Structural Chemistry* **2015**, *71* (1), 3–8. DOI: 10.1107/S2053229614024218

(9) Dolomanov, O. V.; Bourhis, L. J.; Gildea, R. J.; Howard, J. A. K.; Puschmann, H. *OLEX2* : A Complete Structure Solution, Refinement and Analysis Program. *Journal of Applied Crystallography* **2009**, *42* (2), 339–341. DOI: 10.1107/S0021889808042726

(10) Spek, A. L. *PLATON* SQUEEZE: A Tool for the Calculation of the Disordered Solvent Contribution to the Calculated Structure Factors. *Acta Crystallographica Section C: Structural Chemistry* **2015**, *71* (1), 9–18. DOI: 10.1107/S2053229614024929

(11) Hirose, K. A Practical Guide for the Determination of Binding Constants. *Journal of Inclusion Phenomena and Macrocyclic Chemistry* **2001**, *39* (3–4), 193–209. DOI: 10.1023/A:1011117412693

(12) Kannankutty, K.; Chen, C.-C.; Nguyen, V. S.; Lin, Y.-C.; Chou, H.-H.; Yeh, C.-Y.; Wei, T.-C. *Tert*-Butylpyridine Coordination with $[Cu(dmp)_2]^{2+/+}$ Redox Couple and Its Connection to the Stability of the Dye-Sensitized Solar Cell. *ACS Applied Materials & Interfaces* **2020**, *12* (5), 5812–5819. DOI: 10.1021/acsami.9b19119

(13) Boettcher, S. W.; Oener, S. Z.; Lonergan, M. C.; Surendranath, Y.; Ardo, S.; Brozek, C.; Kempler, P. A. Potentially Confusing: Potentials in Electrochemistry. *ACS Energy Letters* **2021**, *6* (1), 261–266. DOI: 10.1021/acsenergylett.0c02443

(14) Bard, A. J.; Faulkner, L. R. *Electrochemical Methods: Fundamentals and Applications,* 2nd ed.; John Wiley & Sons, 2001.

(15) De, A.; Dagar, M.; Kneer, B.; Kim, J.; Thorarinsdottir, A. E. Best Practices for Variable-Temperature Electrochemistry Experiments and Data Reporting. *ACS Energy Letters* **2025**, *10* (4), 1542–1549. DOI: 10.1021/acsenergylett.5c00308

(16) Quickenden, T. I.; Mua, Y. A Review of Power Generation in Aqueous Thermogalvanic Cells. *Journal of The Electrochemical Society* **1995**, *142* (11), 3985–3994. DOI: 10.1149/1.2048446

(17) Dupont, M. F.; MacFarlane, D. R.; Pringle, J. M. Thermo-Electrochemical Cells for Waste Heat Harvesting – Progress and Perspectives. *Chemical Communications* **2017**, *53* (47), 6288–6302. DOI: 10.1039/c7cc02160g

(18) Yee, E. L.; Cave, R. J.; Guyer, K. L.; Tyma, P. D.; Weaver, M. J. A Survey of Ligand Effects upon the Reaction Entropies of Some Transition Metal Redox Couples. *Journal of the American Chemical Society* **1979**, *101* (5), 1131–1137. DOI: 10.1021/ja00499a013

(19) Elgrishi, N.; Rountree, K. J.; McCarthy, B. D.; Rountree, E. S.; Eisenhart, T. T.; Dempsey, J. L. A Practical Beginner's Guide to Cyclic Voltammetry. *Journal of Chemical Education* **2018**, *95* (2), 197–206. DOI: 10.1021/acs.jchemed.7b00361

(20) Willery, M.; Julliard, P.-G.; Molton, F.; Thomas, F.; Fortage, J.; Costentin, C.; Collomb, M.-N. Mechanism of Electrochemical Proton Reduction Catalyzed by a Cobalt Tetraaza Schiff Base Macrocyclic Complex: Ligand Protonation and/or Influence of the Chloro Ligand. *ACS Catalysis* **2024**, *14* (15), 11352–11365. DOI: 10.1021/acscatal.4c03061

(21) Feldman, D. M.; Julliard, P.-G.; Madrigalejo, J.-C.; Fortage, J.; Collomb, M.-N.; Costentin, C. A Self-Moderation Mechanism in CO_2 Electroreduction Catalyzed by

a Cobalt Macrocyclic Complex. *Journal of the American Chemical Society* **2026**, *148* (2), 2769–2778. DOI: 10.1021/jacs.5c19804

(22) Fürer, S. O.; Milhuisen, R. A.; Kashif, M. K.; Raga, S. R.; Acharya, S. S.; Forsyth, C.; Liu, M.; Frazer, L.; Duffy, N. W.; Ohlin, C. A.; Funston, A. M.; Tachibana, Y.; Bach, U. The Performance-Determining Role of Lewis Bases in Dye-Sensitized Solar Cells Employing Copper-Bisphenanthroline Redox Mediators. *Advanced Energy Materials* **2020**, *10* (37), No. 2002067. DOI: 10.1002/aenm.202002067

(23) Randles, J. E. B. A Cathode Ray Polarograph. Part II.—The Current-Voltage Curves. *Transactions of the Faraday Society* **1948**, *44*, 327–338. DOI: 10.1039/TF9484400327

(24) Ševčík, A. Oscillographic Polarography with Periodical Triangular Voltage. *Collection of Czechoslovak Chemical Communications* **1948**, *13*, 349–377. DOI: 10.1135/cccc19480349

(25) Liu, Q.; Sleightholme, A. E. S.; Shinkle, A. A.; Li, Y.; Thompson, L. T. Non-Aqueous Vanadium Acetylacetonate Electrolyte for Redox Flow Batteries. *Electrochemistry Communications* **2009**, *11* (12), 2312–2315. DOI: 10.1016/j.elecom.2009.10.006

(26) Carmona-Pérez, D.; Gao, M.; Andes, S.; Brennessel, W. W.; Thorarinsdottir, A. E. Effect of Coordination Environment and Electronic Coupling on Redox Entropy in a Family of Dinuclear Complexes. *ACS Electrochemistry* **2025**, *1* (5), 741–753. DOI: 10.1021/acselectrochem.4c00186

(27) Liu, Y.; Cui, M.; Ling, W.; Cheng, L.; Lei, H.; Li, W.; Huang, Y. Thermo-Electrochemical Cells for Heat to Electricity Conversion: From Mechanisms, Materials, Strategies to Applications. *Energy & Environmental Science* **2022**, *15* (9), 3670–3687. DOI: 10.1039/D2EE01457B

(28) van Staveren, D. R.; Bothe, E.; Weyhermüller, T.; Metzler-Nolte, N. Variable Temperature Electrochemistry as a Powerful Method for Conformational Investigations on the Fluxional Organometallic Complex Mo(His-N_{ε}-$C_2H_4CO_2Me$)(η-allyl)$(CO)_2$ (His = N_{δ}, *N*,*O*-L-Histidinate). *Chemical Communications* **2001**, *2001* (1), 131–132. DOI: 10.1039/b007822k

(29) Kobayashi, T.; Yamada, T.; Tadokoro, M.; Kimizuka, N. A Novel Thermocell System Using Proton Solvation Entropy. *Chemistry – A European Journal* **2021**, *27* (13), 4287–4290. DOI: 10.1002/chem.202004562

(30) Kratochvil, B.; Lorah, E.; Garber, C. Silver–Silver Nitrate Couple as Reference Electrode in Acetonitrile. *Analytical Chemistry* **1969**, *41* (13), 1793–1796. DOI: 10.1021/ac60282a011

(31) Huang, B.; Muy, S.; Feng, S.; Katayama, Y.; Lu, Y.-C.; Chen, G.; Shao-Horn, Y. Non-Covalent Interactions in Electrochemical Reactions and Implications in Clean Energy Applications. *Physical Chemistry Chemical Physics* **2018**, *20* (23), 15680–15686. DOI: 10.1039/C8CP02512F

(32) Dagar, M.; De, A.; Lu, Z.; Matson, E. M.; Thorarinsdottir, A. E. Implications of Charge and Heteroatom Dopants on the Thermodynamics and Kinetics of Redox Reactions in Keggin-Type Polyoxometalates. *ACS Materials Au* **2025**, *5* (1), 200–210. DOI: 10.1021/acsmaterialsau.4c00136

(33) Hupp, J. T.; Weaver, M. J. Solvent, Ligand, and Ionic Charge Effects on Reaction Entropies for Simple Transition-Metal Redox Couples. *Inorganic Chemistry* **1984**, *23* (22), 3639–3644. DOI: 10.1021/ic00190a042

(34) Blackman, A. G.; Schenk, E. B.; Jelley, R. E.; Krenske, E. H.; Gahan, L. R. Five-Coordinate Transition Metal Complexes and the Value of τ_5: Observations and Caveats. *Dalton Transactions* **2020**, *49* (42), 14798–14806. DOI: 10.1039/D0DT02985H

(35) Yang, L.; Powell, D. R.; Houser, R. P. Structural Variation in Copper(I) Complexes with Pyridylmethylamide Ligands: Structural Analysis with a New Four-Coordinate Geometry Index, τ_4. *Dalton Transactions* **2007**, *2007* (9), 955–964. DOI: 10.1039/B617136B

(36) Drew, M. G. B.; Harding, C. J.; McKee, V.; Morgan, G. G.; Nelson, J. Geometric Control of Manganese Redox State. *Journal of the Chemical Society, Chemical Communications* **1995**, *1995* (10), 1035–1038. DOI: 10.1039/c39950001035

(37) Ansari, M.; Singh, S. P. Viscosities and Densities of Acetonitrile-Water Systems at 25 °C. *Research Journal of Chemical Sciences* **2022**, *12* (1), 67–69. ISSN 2231-606X

(38) Yu, B.; Xiao, H.; Zeng, Y.; Liu, S.; Wu, D.; Liu, P.; Guo, J.; Xie, W.; Duan, J.; Zhou, J. Cost-Effective n-Type Thermocells Enabled by Thermosensitive Crystallizations and 3D Multi-Structured Electrodes. *Nano Energy* **2022**, *93*, No. 106795. DOI: 10.1016/j.nanoen.2021.106795

(39) Wu, M.; Hao, S.; Qi, L.; Shi, Y.; Yang, W.; Bao, J.; Du, M.; Mo, Z.; Sun, L. An N-Type Thermogalvanic Cell with a High Temperature Coefficient Based on the $Cu/Cu(en)_2^{2+}$ Redox Couple. *ACS Applied Materials & Interfaces* **2025**, *17* (18), 26775–26783. DOI: 10.1021/acsami.5c03375
